# Replication Validity of Initial Association Studies: A Comparison between Psychiatry, Neurology and Four Somatic Diseases

**DOI:** 10.1371/journal.pone.0158064

**Published:** 2016-06-23

**Authors:** Estelle Dumas-Mallet, Katherine Button, Thomas Boraud, Marcus Munafo, François Gonon

**Affiliations:** 1 CNRS, UMR 5293, Institute of Neurodegenerative diseases, Bordeaux, France; 2 University of Bordeaux, UMR 5293, Institute of Neurodegenerative diseases, Bordeaux, France; 3 CNRS, UMR 5116, Centre Emile Durkheim, Bordeaux, France; 4 University of Bath, Department of Psychology, Bath, United Kingdom; 5 MRC Integrative Epidemiology Unit, University of Bristol, Bristol, United Kingdom; 6 CHU Bordeaux, Bordeaux, France; University of Vienna, School of Psychology, AUSTRIA

## Abstract

**Context:**

There are growing concerns about effect size inflation and replication validity of association studies, but few observational investigations have explored the extent of these problems.

**Objective:**

Using meta-analyses to measure the reliability of initial studies and explore whether this varies across biomedical domains and study types (cognitive/behavioral, brain imaging, genetic and “others”).

**Methods:**

We analyzed 663 meta-analyses describing associations between markers or risk factors and 12 pathologies within three biomedical domains (psychiatry, neurology and four somatic diseases). We collected the effect size, sample size, publication year and Impact Factor of initial studies, largest studies (i.e., with the largest sample size) and the corresponding meta-analyses. Initial studies were considered as replicated if they were in nominal agreement with meta-analyses and if their effect size inflation was below 100%.

**Results:**

Nominal agreement between initial studies and meta-analyses regarding the presence of a significant effect was not better than chance in psychiatry, whereas it was somewhat better in neurology and somatic diseases. Whereas effect sizes reported by largest studies and meta-analyses were similar, most of those reported by initial studies were inflated. Among the 256 initial studies reporting a significant effect (p<0.05) and paired with significant meta-analyses, 97 effect sizes were inflated by more than 100%. Nominal agreement and effect size inflation varied with the biomedical domain and study type. Indeed, the replication rate of initial studies reporting a significant effect ranged from 6.3% for genetic studies in psychiatry to 86.4% for cognitive/behavioral studies. Comparison between eight subgroups shows that replication rate decreases with sample size and “true” effect size. We observed no evidence of association between replication rate and publication year or Impact Factor.

**Conclusion:**

The differences in reliability between biological psychiatry, neurology and somatic diseases suggest that there is room for improvement, at least in some subdomains.

## Introduction

Many opinion and review articles have lamented the poor reproducibility of biomedical studies (e.g., [1–4]). However, relatively few empirical studies have quantitatively explored this issue across preclinical, clinical and association studies. The reproducibility of preclinical studies has been recently reviewed [5]; five studies estimated the prevalence of irreproducible results in preclinical studies to be between 51 and 89%. Regarding clinical trials of new treatments, two lines of evidence point to the low replication validity of initial studies. First, only 40% of phase II trials claiming a significant benefit of new drugs are confirmed when those drugs are subsequently tested in phase III trials [6, 7]. Second, most initial studies of a new treatment report a larger effect than subsequent studies or meta-analyses on the same topic [8–10, 11, 12]. Similarly, initial studies reporting a significant genetic association with various pathologies are often shown to report inflated effects when compared to the results of subsequent studies [13–15]. When the effect sizes associated with biomarkers reported in 35 highly cited studies were compared to those reported in the corresponding meta-analyses [16], 14 were initial studies reporting a significant association, of which one was disconfirmed by the corresponding meta-analysis and seven reported an effect size at least twice as large as that the one indicated in the corresponding meta-analysis.

The methods used in the four studies listed above were different. Two selected initial studies, and then searched for subsequent studies on the same topic [14, 16]. The other two exploited a database of 36 meta-analyses [13] that was extended to 55 meta-analyses [15]. Meta-analyses provide a unique way to compare initial findings with subsequent studies, and this approach has been used by Ioannidis and colleagues to estimate the replication validity of clinical trials [8–11]. Unfortunately, this approach cannot yet be widely used for preclinical studies because only a few meta-analyses exist in this area. In contrast, numerous recent meta-analyses have tested the association between markers or risk factors and various diseases. Because this type of biomedical research is crucial for improving the understanding and diagnosis of diseases, an estimate of the replication validity of these studies is timely. Since the four previously published estimates [13–16] mainly focused on genetic associations and were based on a relatively small number of meta-analyses, we decided to investigate this question on a larger scale.

According to Button and colleagues [17], “the average statistical power of neuroscience studies is very low. The consequences of this include overestimates of effect size and low reproducibility of the results.” Their analysis used a database of 49 meta-analyses published in 2011 in the field of neuroscience, most pertaining to psychiatric disorders or neurological diseases. This raises two questions. First, is the replication rate of initial studies in psychiatry similar to neurology? Second, how do these compare to a sample of somatic diseases? The present study was designed to address both questions. To assess the credibility of studies investigating the association of markers or risk factors with diseases, we collected all meta-analyses published during a five year period related to four psychiatric disorders (attention deficit hyperactivity disorder (ADHD), autism, unipolar depression, schizophrenia), four neurological diseases (Alzheimer and Parkinson diseases, epilepsy and multiple sclerosis) and four somatic diseases (breast cancer, glaucoma, psoriasis and rheumatoid arthritis). We aimed to test whether initial studies were in agreement with the corresponding meta-analyses and to calculate their effect size inflation. We then explored several factors that might influence discrepancies between initial studies and meta-analyses: the level of statistical significance of the corresponding meta-analyses and of the initial studies, the biomedical domain, the study type (e.g., genetic), the sample size, the journal Impact Factor and the publication year.

## Methods

### Selection of meta-analyses for Inclusion

We searched PubMed for relevant articles, with the search limited to articles referenced as "meta-analysis" and published in English between January 1st 2008 and December 31st 2012. Studies published online in 2012 but printed in 2013 or 2014 were also considered. We used the following key words for each disease: “attention deficit hyperactivity disorder”, “autism”, “major depressive disorder”, “epilepsy”, “Alzheimer disease”, “Parkinson disease”, “multiple sclerosis”, “breast cancer”, “glaucoma”, “psoriasis” and “rheumatoid arthritis”.

Meta-analyses captured by this search strategy were screened by two authors (EDM and FG) for eligibility. First, the title and the abstract were considered. Second, full texts were obtained for the remaining studies and screened by two authors (EDM or FG). Articles were excluded if: 1) they were related to the treatment, screening or diagnosis of the disease, 2) if they reported on voxel-based brain imaging studies, 3) the disease itself was the risk factor for another outcome, 4) no meta-analysis was conducted, or 5) the article was not related or relevant to the disease. Meta-analyses were included if: 1) they compiled results from at least 7 distinct datasets reported in at least 4 independent publications, 2) the effect size was expressed as mean difference, standardized mean difference (Cohen’s d), odds ratio (OR) or risk ratio (RR), and 3) parameters were measured and/or validated by professionals (i.e., psychological traits that were only self-assessed by subjects were not considered).

A number of articles reported several meta-analyses. We included all of them providing that they were dealing with distinct parameters or risk factors. If an article reported several meta-analyses on the same parameter and the same outcome, we selected the most comprehensive one (i.e., the one containing the most datasets) and the one reporting the fixed effects rather than the random effects model. When two or more articles dealt with the same parameter and the same outcome, the most recent one was used. For genetic association studies, we used the meta-analysis results for the allelic model when the data were available. However, when the corresponding initial study put forward another model we selected this one.

### Selection of initial studies and largest studies

From each meta-analysis we identified the initial study and we collected its full text article. We checked them to assert that no study on the same topic was previously published. We thus identified 110 initial studies that were not listed in the corresponding meta-analyses. These omissions were mainly due to two reasons. First, a meta-analysis article of 36 associations between brain imaging observations and schizophrenia explicitly mentioned that data published before 1998 were not taken into account. Therefore, we looked for the “true” initial studies. Second, initial studies were often replicated by the same group using an extended population and the second publication often included the initial data. Therefore, the corresponding meta-analyses did not include the initial study. Nevertheless, we used here the data of the “true” initial studies. From each meta-analysis we also identified the largest study as the one that included the largest number of patients.

### Data extraction

Data were extracted independently by two authors (EDM and FG), and discrepancies resolved by mutual consent. A third author (KB) conducted a further 10% data check, which resulted in a small number of errors being detected and corrected. The following data were extracted from initial, largest and meta-analysis articles: year of publication, first author, 2012 Impact Factor of the journal that published it, nominal statistical significance (based on p<0.05), effect size, its p-value for datasets reporting a significant effect or its confidence interval for non-significant ones, number of patients and of healthy controls. Moreover, regarding meta-analyses we also extracted the number of included datasets.

If the effect size was reported as a mean difference, it was converted to Cohen’s d using the software OpenMetaAnalyst [18]. Meta-analyses and their corresponding initial and largest studies were excluded if data were incomplete or if the reporting was unclear. In order to compare the effect sizes across all meta-analyses and diseases, effect sizes were natural logarithm transformed. The effect sizes expressed as Cohen’s d were converted to ln(OR) using the formula ln(OR) = d × π/√3. For each disease, we calculated the average effect size of the initial and largest studies as well as of the meta-analyses and the median of the number of patients. These raw data are given in Supporting Information (S1 File).

### Sensitivity and specificity analysis

In order to describe the nominal agreement between each initial study and its corresponding meta-analysis we used, as previous authors have done [15], two concepts borrowed from biomedical diagnostic testing: sensitivity and specificity. In the context of our study, sensitivity expresses the conditional probability that an initial study predicts a significant effect when a significant effect is actually confirmed by the corresponding meta-analysis. Specificity expresses the probability that an initial study reporting a non-significant effect corresponds to a meta-analysis supporting the same view.

This agreement was coded according to four possibilities: 1) initial study and meta-analysis both report a significant effect at p<0.05, 2) initial study and meta-analysis both report a non-significant effect, 3) the initial study reports a significant effect whereas its associated meta-analysis do not (false positive), and 4) the initial study reports a non-significant effect whereas the associated meta-analysis reports a significant one (false negative). In addition we observed that six initial studies reporting a significant effect corresponded to meta-analyses reporting a significant effect in the opposite direction. These six initial studies were coded as case 3 (false positive). The sensitivity and specificity were calculated using the Clinical Calculator 1 (vassarstats.net). Their confidence intervals at 95% were given by this online calculator according to Newcombe [19].

### Defining a small effect

We used our dataset of meta-analyses reporting a significant association to define a small effect in each of our four association types: cognitive/behavioral, brain imaging, genetic and other. For each category we sorted in quintiles the significant effect sizes. The largest effect size of the lowest quintile was defined as the maximal value of a small effect. All effects sizes of the 65 significant cognitive/behavioral studies were expressed as standardized mean difference and a small effect was estimated to be d ≤ 0.51. All but two effect sizes of the 69 significant brain imaging studies were expressed as standardized mean difference. The two others were expressed as odds ratio and were converted to standardized mean difference. This gave a small effect as d ≤ 0.29. All 136 significant genetic associations were expressed as odds ratio and a small effect was defined as OR ≤ 1.11. Finally, the 146 significant “other” studies were first devised into two categories: 60 were expressed as standardized mean difference and 96 as odds ratios. Regarding the former, a small effect was d ≤ 0.30 and the latter was OR ≤ 1.20.

### Statistical analyses

Effect sizes of initial or largest studies were compared to those of the corresponding meta-analyses using Wilcoxon’s test for paired observations, as others have done with similar data [20, 21]. Other comparisons were tested using the χ^2^ test. The relationship between effect size inflation and Impact factor was analyzed using linear regression and also Spearman’s rank correlation. These statistical analyses were done using SigmaPlot software.

## Results

### Characteristics of included studies

We included 663 meta-analyses reporting an association between a marker or a risk factor and a disease outcome in one of the three domains of interest (psychiatry, neurology and somatic diseases). Among these, 66 meta-analyses were related to cognitive test or behavioral observation and 97 to brain imaging studies. All but two meta-analyses were in the domain of psychiatry (see Table 1). Genetic studies were widely represented in all three domains (Table 1). Finally, associations classified as “other” corresponded to a wide variety of studies (e.g., epidemiology, blood tests, physical characteristics). Table 1 summarizes the number of articles selected at each step of the process, and the number of meta-analyses included for each pathology. The selection of studies is also shown in flow diagrams (see supporting information S1 Fig).

**Table 1:** Number of studies identified in three research domains.

	Psychiatric disorders				Neurological diseases				Somatic diseases			
	ADHD	ASD	MDD	SCZ	AD	Epi	MS	PD	BC	Glau	Pso	RA
PubMed search	118	71	553	454	197	147	137	139	811	75	81	285
Examined full texts	43	36	114	198	117	29	44	74	345	19	22	82
Included articles	20	13	28	59	35	12	18	20	89	9	8	22
Included meta-analyses	40	24	53	203	50	15	37	57	114	21	15	34
*Cognitive/behavioral*	14	0	9	43	1	0	0	1	0	0	0	0
*Brain imaging*	2	10	19	64	1	1	0	0	0	0	0	0
*Genetic studies*	12	4	8	56	33	3	17	30	61	17	12	31
*Other studies*	12	10	17	40	15	11	20	26	53	4	3	3

ADHD: attention deficit hyperactivity disorder. ASD: autism spectrum disorder. MDD: major depressive disorder. SCZ: schizophrenia. AD: Alzheimer disease. Epi: epilepsy. MS: multiple sclerosis. PD: Parkinson disease. BC: breast cancer. Glau: glaucoma. Pso: psoriasis. RA: rheumatoid arthritis.

### Do initial studies agree with subsequent meta-analyses?

**Fig 1:**
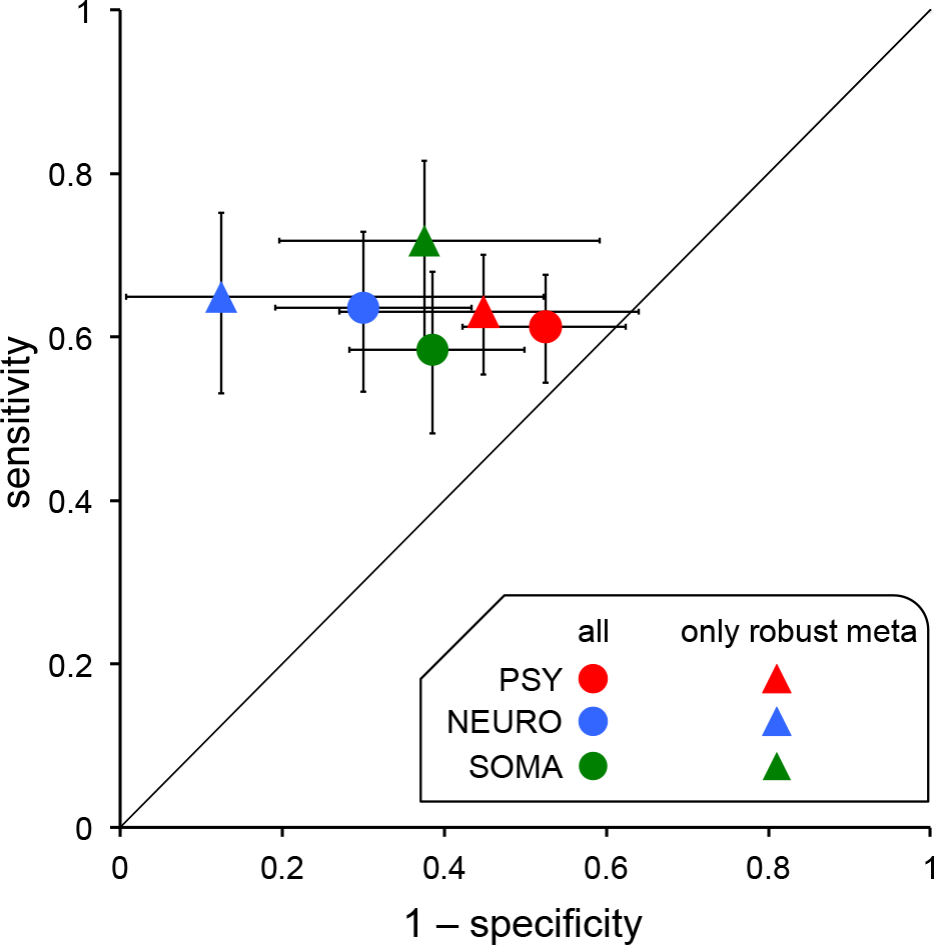
ROC diagram expressing the sensitivity and specificity of initial studies in agreeing with corresponding meta-analyses regarding the presence or absence of a nominally significant effect. For the three biomedical domains the diagram either considered all 663 pairs of initial studies and meta-analyses (circles) or only the 385 initial studies paired with stringent meta-analyses (triangles). Error bars represent the confidence intervals at 95%. The dashed diagonal line corresponds to no discrimination above chance. The fact that errors bars related to psychiatry cross this line suggests that the agreement between initial studies and corresponding meta-analyses might not be better than chance. PSY: psychiatry; NEURO: neurology; SOMA: somatic diseases.

As previous authors have done [15], we calculated the sensitivity and specificity of initial studies to predict the presence or absence of an association indicated by the corresponding meta-analysis (defined as statistically significant at p<0.05). The sensitivity of initial studies ranged from 0.58 to 0.64 and was very similar across the three biomedical domains (Table 2). In contrast, the specificity ranged from 0.47 to 0.70 (Table 2) and there was some evidence that this was lower for initial studies in psychiatry when compared to neurology (χ^2^ test: p = 0.0055) and somatic diseases (p = 0.059). We used a ROC diagram to plot the sensitivity as a function of (1-specificity). This indicated that the agreement between initial studies and corresponding meta-analyses may not be better than chance in psychiatry whereas it was somewhat better in neurology and somatic diseases (Fig 1).

**Table 2:** Diagnostic performance of initial studies against the statistical significance of the corresponding meta-analyses.

	PSY	NEURO	SOMA
**All meta-analyses**	320	159	184
significant meta-analyses	221 (69.1%)	100 (62.9%)	104 (56.5%)
sensitivity	0.612 (0.544 0.676)	0.636 (0.533 0.729)	0.584 (0.482 0.680)
specificity	0.475 (0.376 0.577)	0.700 (0.566 0.808)	0.614 (0.501 0.717)
**Stringent meta-analyses**	205	85	95
significant meta-analyses	178 (86.8%)	77 (90.6%)	71 (74.7%)
sensitivity	0.625 (0.549 0.696)	0.649 (0.531 0.752)	0.718 (0.597 0.816)
specificity	0.533 (0.346 0.712)	0.875 (0.477 0.993)	0.625 (0.408 0.804)

PSY: psychiatric disorders; NEURO: neurological diseases; SOMA: somatic diseases.

Confidence intervals at 95% are given in parentheses.

Previous studies have shown that meta-analyses reporting a significant effect at p<0.05 are sometimes disconfirmed by subsequent meta-analyses whereas those reporting stronger statistical evidence (p<0.005) were rarely disconfirmed [20, 22]. This higher level of statistical stringency may increase the credibility of scientific research [23]. Furthermore, a meta-analysis reporting a non-significant effect only means that its confidence interval contains the null value, but does not prove the absence of an association. However, the confidence interval can be used to reject an effect larger than a predefined threshold [24]. If the 95% confidence interval excludes anything other than a small effect, the study under consideration is able to reject a larger effect. However, there is no general rule that defines what is a small effect–it depends on the type of association [22]. Here we used our database of 425 significant associations reported by meta-analyses to define a small effect in each of our four association types: cognitive/behavioral, brain imaging, genetic and other (see Methods).

We therefore conducted a sensitivity analysis where we restricted our analysis to associations investigated by stringent meta-analyses. We defined a meta-analysis as stringent either if it reported an effect as significant at p<0.005 or, when non-significant, if its confidence interval excluded a threshold effect, as determined relatively to typical effect sizes in that field. Using these criteria we categorized 385 meta-analyses as stringent, of which 326 reported a significant effect (Table 2). When initial studies were compared to these corresponding stringent meta-analyses, sensitivity and specificity remained similar to those observed when all 663 associations were considered (Table 2). Again, the ROC diagram shows that the agreement between initial studies in psychiatry and their corresponding stringent meta-analyses might not be better than chance (Fig 1).

### Do initial studies report inflated effect sizes?

**Fig 2:**
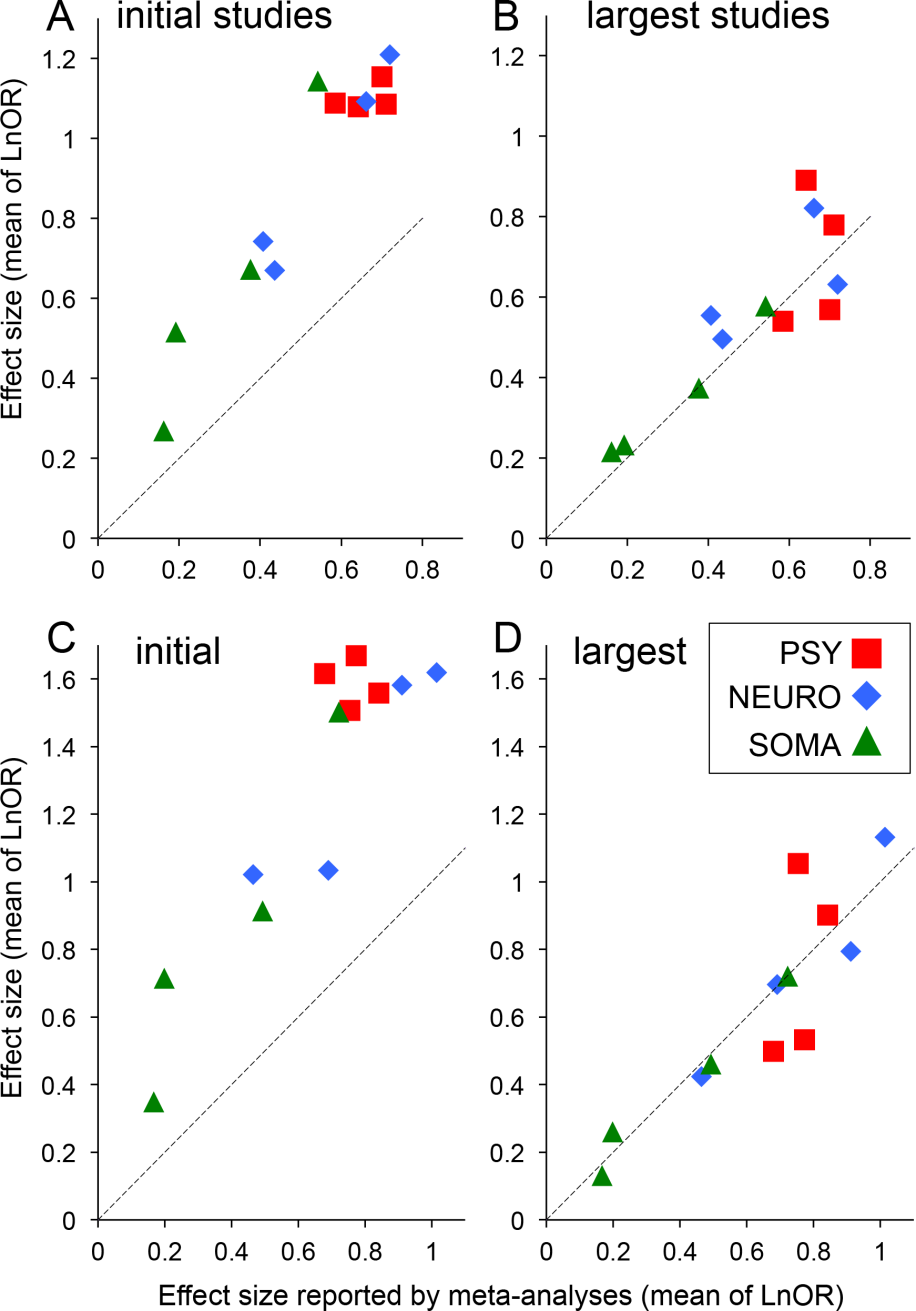
Comparison between effect sizes reported by initial or largest studies and their corresponding meta-analyses. All effect sizes were expressed as LnOR (standardized mean differences were converted to LnOR as described in the Methods). (A) and (C) Mean of the effect sizes reported by initial studies for each pathology. (B) and (D) Mean of the effect sizes reported by largest studies for each pathology. (A) and (B) All 663 trios were considered. (C) and (D) Only the 359 initial studies reporting a significant effect and their corresponding largest studies. The dashed lines correspond to equality between the effect sizes of the initial studies or of the largest studies and the effect sizes reported by the meta-analyses. PSY: psychiatry; NEURO: neurology; SOMA: somatic diseases.

Even if initial studies and corresponding meta-analyses both report a significant effect, initial studies might be misleading by reporting an inflated estimate of the effect size. Among the 256 initial studies reporting a significant effect and paired with a meta-analysis reporting a significant effect in the same direction, 150 initial studies (59%) reported an effect size inflated by more than 50%, of which 97 were inflated by more than 100%. When we analyzed the 12 pathologies under consideration separately, the average effect size reported by initial studies was always larger than that reported by subsequent meta-analyses (Fig 2A). In contrast the effect size reported by the corresponding largest studies were much more consistent with the meta-analyses (Fig 2B and 2D). Indeed, for all pathologies, the effect size distribution differed between those reported by initial studies and those reported by meta-analyses (Wilcoxon’s test for paired observations). There was clear statistical evidence for most of these differences (p<0.002) except for autism (p = 0.025), epilepsy (p = 0.02) and psoriasis (p = 0.009). A similar pattern was observed when considering only initial studies reporting a significant effect (Fig 2C).

### Are initial studies consistent with subsequent meta-analyses?

To summarize the consistency between initial studies and corresponding meta-analyses we classified as replicated: *i)* initial studies reporting a non-significant effect and corresponding to non-significant meta-analyses, and *ii)* initial studies reporting a significant effect and corresponding to significant meta-analyses providing that the inflation of the effect size did not exceed a certain percentage threshold. We tested two inflation thresholds: 100% and 50% (Table 3). Regardless of the threshold, initial studies dealing with psychiatric disorders were less often in agreement with the corresponding meta-analyses than those dealing with neurological diseases (Table 3). However, there was no evidence that replication rates differed between psychiatric disorders and somatic diseases (Table 3). We also analyzed initial studies reporting a significant effect only and we did not observe any evidence of a difference between the three biomedical domains (Table 3).

**Table 3:** Number of initial studies consistent with corresponding meta-analyses.

	number of studies						comparisons		
	PSY		NEURO		SOMA		PSY /	PSY /	NEURO /
	#	%	#	%	#	%	NEURO	SOMA	SOMA
**All initial studies**	320		159		184				
initial consistent (inflation ≤100%)	131	40.9	85	53.5	84	45.7	p = 0.0094	p = 0.30	p = 0.15
initial consistent (inflation ≤50%)	101	31.6	71	44.7	75	40.8	p = 0.0049	p = 0.037	p = 0.47
*Only initial reported as significant*									
number of studies	187		81		91				
initial consistent (inflation ≤100%)	83	44.4	43	53.1	33	36.3	p = 0.19	p = 0.20	p = 0.27
**Only highly significant initial**									
number of studies with p < 0.005	94		44		48				
initial consistent (inflation ≤100%)	42	44.7	23	52.3	23	47.9	p = 0.41	p = 0.71	p = 0.68
**Without cognitive/behavioral**	254		157		184				
initial consistent (inflation ≤100%)	90	35.4	84	53.5	84	45.7	p = 0.0003	p = 0.031	p = 0.15
initial consistent (inflation ≤50%)	70	27.6	71	45.2	75	40.8	p = 0.0003	p = 0.0039	p = 0.41
*Only initial reported as significant*									
number of studies	142		79		91				
initial consistent (inflation ≤100%)	45	31.7	42	53.2	33	36.3	p = 0.0019	p = 0.47	p = 0.028

PSY: psychiatric disorders; NEURO: neurological diseases; SOMA: somatic diseases.

When considering only initial studies paired with 385 stringent meta-analyses, these results did not change substantially (Table 4). However, when considering only initial studies reporting a significant effect, the percentage of initial studies in agreement with corresponding stringent meta-analyses was larger than those observed when considering all 663 pairs (see Table 4 vs Table 3). This is due to the fact that the percentage of meta-analyses reporting a non-significant effect was much smaller when we only considered stringent meta-analyses (Table 2). Therefore, the number of significant initial studies associated with non-significant stringent meta-analyses was reduced.

**Table 4:** Number of initial studies consistent with corresponding stringent meta-analyses.

	number of studies						comparisons		
	PSY		NEURO		SOMA		PSY / NEURO	PSY / SOMA	NEURO / SOMA
	#	%	#	%	#	%			
**All initial studies**	205		85		95				
initial consistent (inflation ≤100%)	93	45.4	44	51.8	47	49.5	p = 0.32	p = 0.51	p = 0.76
*Only initial reported as significant*									
number of studies	124		51		60				
initial consistent (inflation ≤100%)	77	62.1	37	72.5	32	53.3	p = 0.19	p = 0.26	p = 0.037

PSY: psychiatric disorders; NEURO: neurological diseases; SOMA: somatic diseases.

### Are highly significant initial studies more reliable?

We applied our criteria of a stringent study to initial studies by removing those reporting a significant effect with a p-value >0.005 and those that did not exclude a small effect on the basis of their confidence interval. On this basis only five of the 304 initial studies reporting a non-significant effect were able to exclude a small effect and only one was consistent with the corresponding meta-analysis. Of the 359 initial studies reporting a significant effect, only 186 reported a p-value <0.005. We compared their replication rate to that of all initial studies reporting a significant effect (Table 3). We observed no difference. Therefore, initial studies reporting a highly significant effect (p<0.005) are no more reliable than those reporting a p-value between 0.05 and 0.005.

### Does replication rate of initial studies differ by study type?

We tested whether the agreement of initial studies with their corresponding meta-analyses depends on the biomedical domain and on the type of study. In order to do so, we defined a replication rate as the percentage of initial studies that were in nominal agreement with meta-analyses and whose effect size inflation was ≤100%. In this subgroup analysis, we did not take into account four associations with neurological diseases (two brain imaging and two cognitive/behavioral studies) due to the small number of studies. We thus considered eight homogenous subgroups. Associations with psychiatric disorders were sorted into four subgroups: cognitive/behavioral, brain imaging, genetic and “other” studies. Associations with neurological and somatic diseases were both sorted into two subgroups: genetic and “other” studies. Table 5 gives the number and percentage of initial studies that were consistent with their corresponding meta-analyses in each subgroup, whether considering all 659 meta-analyses or only the 382 defined as stringent. Data restricted to initial studies reporting a significant effect are given in Table 6.

**Table 5:** Replication rate of initial studies in 8 subgroups.

	All meta-analyses			Stringent meta-analyses		
	# stud	# consist	%	# stud	# consist	%
**Psychiatry**						
Cognitive/behavioral	66	41	62.1	65	41	63.1
Brain imaging	95	36	37.9	55	23	41.8
Genetic	80	26	32.5	26	7	26.9
“Other” studies	79	28	35.4	59	22	37.3
**Neurology**						
Genetic	83	40	48.2	39	16	41.0
“Other” associations	72	42	58.3	43	26	60.5
**Somatic diseases**						
Genetic	121	60	49.6	57	32	56.1
“Other” associations	63	24	38.1	39	16	41.0

Initial studies are consistent if inflated by 100% or less.

**Table 6:** Replication rate of significant initial studies in 8 subgroups.

	All meta-analyses			Stringent meta-analyses		
	# stud	# consist	%	# stud	# consist	%
**Psychiatry**						
Cognitive/behavioral	45	38	84.4	44	38	86.4
Brain imaging	50	21	42.0	28	18	64.3
Genetic	46	3	6.5	16	1	6.3
“Other” studies	46	21	45.7	36	20	55.6
**Neurology**						
Genetic	37	14	37.8	19	11	57.9
“Other” associations	41	27	65.9	29	24	82.8
**Somatic diseases**						
Genetic	61	24	39.3	40	24	60.0
“Other” associations	30	9	30.0	20	8	40.0

Initial studies are consistent if inflated by 100% or less.

Tables 5 and 6 show that the replication rate of initial studies differs considerably between subgroups. Within psychiatry, initial studies of cognitive/behavioral associations exhibited the highest replication rate, whereas those regarding genetic associations were the least replicated. As all but two cognitive/behavioral meta-analyses were related to psychiatric disorders, we also compared the replication rate of initial studies in the three domains when restricting the analysis to biomarkers and biological risk factors (Table 3). This analysis shows that initial studies in biological psychiatry were less reliable than those of both other biomedical domains.

### Does replication rate depend on sample size and “true” effect size?

**Fig 3:**
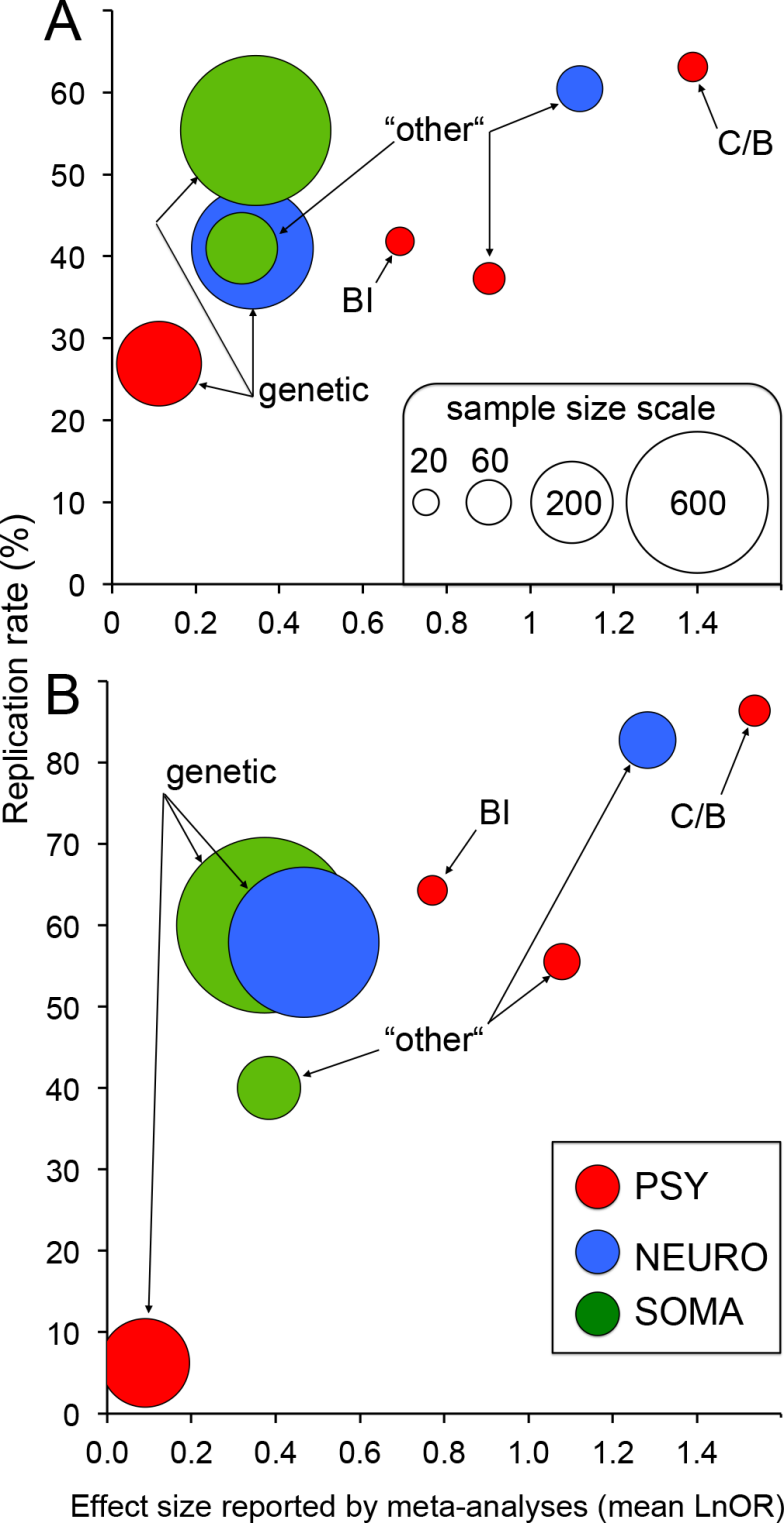
Replication rate of initial studies depending on the “true” effect and on the sample size. The replication rate was calculated for the eight subgroups as the percentage of initial studies that were consistent with meta-analyses and whose effect size inflation was ≤100% (raw data are given in Tables 5 and 6). The average of the “true” effect size was the mean of the summary effect size reported by each stringent meta-analysis for each subgroup. The sample size corresponded to the median of the number of patients of all initial studies in each subgroup and was represented as the area of the circles (same scale for (A) and (B)). (A) All initial studies paired with stringent meta-analyses (n = 382). (B) Only initial studies reporting a significant effect and paired with stringent meta-analyses (n = 232). BI: brain imaging studies; C/B: cognitive/behavioral studies; PSY: psychiatric disorders; NEURO: neurological diseases; SOMA: somatic diseases.

In order to elucidate the factors contributing to the replication rate of initial studies, we built three-dimensional graphs expressing for each subgroup how the replication rate was related to the “true’ effect size (i.e., the effect size reported by stringent meta-analyses) and to sample size (i.e., the number of patients in initial studies) (Fig 3). Whether considering all initial studies (Fig 3A) or only those reporting a significant effect (Fig 3B), subgroups exhibiting the lowest replication rates were associated with either a smaller “true” effect size or a lower sample size or both.

### Are largest studies more reliable?

We used the same procedures to evaluate the performance of largest studies against their corresponding meta-analyses. With respect to the nominal agreement with corresponding meta-analyses, the sensitivity of largest studies (Table 7) was similar to that of initial studies (Table 2), but their specificity was better. This was true either when all 663 associations were considered or when the analysis was restricted to stringent meta-analyses (Table 7). Moreover, there was no evidence that the performance of the largest studies differed between the three domains (Table 7).

**Table 7:** Diagnostic performance of largest studies against the statistical significance of the corresponding meta-analyses.

	PSY	NEURO	SOMA
**All meta-analyses**	320	159	184
sensitivity	0.586 (0.518 0.652)	0.646 (0.543 0.738)	0.538 (0.438 0.636)
specificity	0.82 (0.728 0.887)	0.817 (0.691 0.901)	0.85 (0.749 0.917)
**Stringent meta-analyses**	205	85	95
sensitivity	0.669 (0.594 0.736)	0.795 (0.686 0.875)	0.643 (0.519 0.751)
specificity	0.926 (0.742 0.987)	1 (0.598 1)	0.875 (0.665 0.967)

PSY: psychiatric disorders; NEURO: neurological diseases; SOMA: somatic diseases.

Confidence intervals at 95% are given in parentheses.

The replication rate of largest studies was much better than that of initial studies. It ranged from 62.5% to 67.9% when all largest studies were considered, and from 70.6% to 80.4% when only significant largest studies were taken into account. Again, we observed no significant difference between the three domains.

### Are studies published in high Impact Factor journals more reliable?

In our sample of 663 associations, initial studies dealing with psychiatric disorders were published in scientific journals exhibiting a higher Impact Factor than those dealing with neurological diseases or with our set of somatic diseases (Impact Factor median: 7.7 versus 4.8 versus 5.6, respectively). However, there was no statistical evidence that the Impact Factor distributions differed between the three domains.

To test the association of Impact Factor and the replication rate of initial studies, we calculated an inflation index for every initial study defined as the ratio of its effect size divided by the effect size reported by the corresponding meta-analysis [25]. We plotted this ratio against the Impact Factor of the journal that published the initial study. Data were analyzed using linear regression. This analysis was performed separately for each three domains and for the eight subgroups. No negative relationships where observed. We found no evidence of a positive relationship between inflation score and Impact Factor except in three subgroups: genetic studies related to psychiatry (r^2^ = 0.06, p = 0.026, n = 80) and to neurology (r^2^ = 0.06, p = 0.025, n = 83) as well as “other” studies related to somatic diseases (r^2^ = 0.11, p = 0.008, n = 63). Nevertheless, the strength of association in each case was modest. We also tested whether the inflation index was correlated with the Impact factor using Spearman’s rank correlation. None of the eleven tested associations reached statistical significance at p<0.05.

### Does publication year influence the reliability?

In our sample of 663 initial studies only 40 were published before 1980 and none in 2010 or later. Therefore, in order to test a possible influence of the publication year we sorted our sample into four groups, those published before 1980 and those published during the last three decades (1980s, 1990s and 2000s). There was no evidence that publication year was associated with the replication rate of initial studies whether analyzing all associations together or considering each three domains separately.

However, among genetic studies, the publication year of initial studies reporting a significant effect deserves to be examined in detail. Indeed, in our sample of initial studies, only 2/46 psychiatric studies were published between 2005 and 2009 whereas for neurological and somatic diseases 17/37 and 22/61 studies were published during this five-year period, respectively. Because for neurological and somatic subgroups the sample size increased more than six-fold during this period, due to the growth in large collaborative studies, the replication rate of their initial studies was three times larger than for those published before 2005. Thus, the fact that our sample of initial genetic studies related to psychiatry included only two studies published after 2004 partly contributes to the overall impression that they were less often replicated (Table 6).

### Are initial studies representative of later studies?

**Fig 4:**
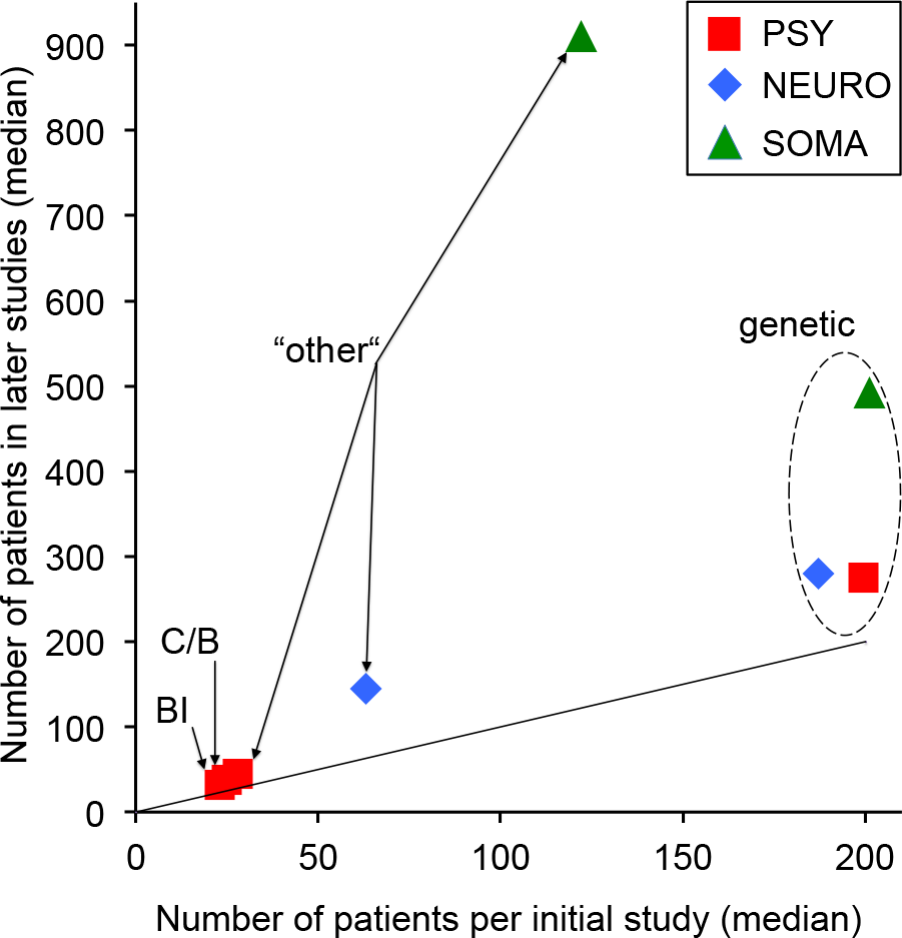
Relationship between the number of patients in initial studies and the average number of patients in subsequent studies. The average number of patients in subsequent studies was calculated by subtracting the number of patients in initial studies from the total number of patients included in each meta-analysis and by dividing it by the number of datasets minus one. The 659 averaged sample sizes of subsequent studies were sorted in eight subgroups and we calculated the median for each subgroup. These eight medians were plotted as a function of the medians of the number of patients in initial studies for each subgroups. The dashed line corresponds to equality between both median types. BI: brain imaging studies; C/B: cognitive/behavioral studies.

Fig 4 shows the relationship between the number of patients in initial studies and the average number of patients in subsequent ones. This average number of patients was calculated by subtracting the number of patients in initial studies from the total number of patients included in each meta-analysis and by dividing it by the number of datasets minus one. For the eight subgroups, the sample size of initial studies was always smaller than for the averaged subsequent studies, but with noticeable differences (Fig 4). The average sample size of subsequent studies was at least twice as large as for initial studies regarding three subgroups: somatic studies in genetics as well as neurological and somatic studies of the “other” type (ratio: 2.4, 2.3 and 7.5, respectively). Because in these three subgroups the subsequent studies included, on average, many more patients, many are expected to be more reliable than initial studies. In contrast, concerning the five other subgroups (i.e., the four subgroups related to psychiatry and the genetic neurological studies), the number of patients in subsequent studies was, on average, only 1.4 to 1.6 times larger than that of the corresponding initial studies. Therefore, the replication rate of these subsequent studies would be expected to be similar to that of the corresponding initial studies. However, this warrants further research.

## Comments

Our observations confirm that the replication validity of association studies is low. The agreement between initial studies and their corresponding meta-analyses regarding the presence or absence of a significant effect is not better or only poorly better than chance depending on the biomedical domain. Moreover, 43% of initial significant studies paired with a significant meta-analysis reported an effect size inflated by more than 100%. Our major finding is that lack of replication validity and effect size inflation differ across biomedical domains and study types. Among initial studies reporting a significant effect, the replication rate ranged from 6.3% for genetic studies of psychiatric disorders to 86.4% for cognitive/behavioral studies of psychiatric disorders. When comparing the three biomedical domains we observed no major differences, but when this comparison was restricted to biomarkers and biological risk factors, initial studies related to biological psychiatry appeared less reliable than those of both other domains.

Button and colleagues suggested that small sample size undermines the reliability of many neuroscience studies [17]. The present study reaches the same general conclusion and extends it. First, in our samples, initial neurological studies are at least as reliable as those related to a set of four somatic diseases. Second, cognitive/behavioral initial studies related to psychiatric disorders appeared much more reliable than other psychiatric studies. Third, initial studies related to biological psychiatry are less reliable than those related to neurological diseases.

Theoretical simulations show that small sample size undermines reproducibility and increases effect size inflation [1, 17, 26]. Previous observational studies [9, 11, 27] as well as the present study support this view. Moreover, the comparisons between eight subgroups illustrate that the relationship between sample size and effect size inflation is relative. When the “true” effect size is large, as in our sample of cognitive/behavioral studies, even initial studies with a small sample size are replicated. In contrast, initial genetic studies related to psychiatry were less often replicated than in neurology not because their sample size was smaller but because it was too small to accurately deal with a much smaller “true” effect size. The fact that the replication rate of largest studies is much better than that of initial studies further supports the view that inadequate sample size is a major cause of low replication validity.

Three previous studies have already highlighted that many brain imaging studies report inflated effect sizes [28–30]. In particular, Ioannidis evaluated the 461 primary datasets included in 41 meta-analyses on the associations between psychiatric disorders and brain volume abnormalities [28]. This demonstrated that the number of primary studies reporting a significant effect was about twice as large as what could be expected from the “true” effects reported by the meta-analyses. Our observations regarding the 95 brain imaging studies related to psychiatric disorders are consistent with this previous study.

The effect size inflation and the poor replication validity of candidate gene studies of complex diseases have been already described [13, 15, 31]. In particular Trikalinos and colleagues analyzed 55 meta-analyses and showed that initial studies poorly predicted the presence or absence of a nominally significant effect reported by the corresponding meta-analysis [15]. More surprisingly, but in agreement with our own observations, they showed that this predictive value was not improved when considering only initial studies reporting a highly significant effect (p<0.005).

In our sample, 38 out of 45 initial studies reporting a significant association between cognitive/behavioral observations and psychiatric disorders were in agreement with the corresponding meta-analysis. This represents a much better performance than expected. Indeed, collaborative attempts to replicate observations in cognitive psychology showed that only 21 out of 42 initial studies reporting a significant effect were replicated [21]. Moreover, psychological science suffers from the same reporting biases as biomedical science [32].

We observed weak positive relationships between the inflation score of initial studies and the Impact Factor of the journal that published them for three subgroups including genetic studies in psychiatry and neurology. No negative relationships where observed. Our observations are in agreement with the positive relationship previously reported regarding 81 primary genetic studies related to psychiatry, although our relationships are clearly weaker [25]. Nevertheless, both studies at least suggest that the reliability of initial studies does not increase with the journal Impact Factor and this conclusion has also been drawn about preclinical studies [33].

## Limitations

Our study is based on the view that the summary effect reported by a meta-analysis is a reliable estimate of the “true” effect. For this reason we only considered meta-analyses dealing with at least seven independent datasets published in at least 4 independent publications. Indeed, a comparison between pairs of meta-analyses published at a five-years interval about the same association showed that the change in summary effect size was inversely related to the number of datasets [20]. Despite this selection criterion, it is likely that some meta-analyses of our sample still reported an inflated effect or even a false positive effect. Indeed, publication bias (i.e., the preferential publication of positive results) and the inclusion of primary studies with low methodological quality are likely to inflate the effect size estimated by meta-analyses [34, 35]. However, without study pre-registration, these biases are difficult to detect. Several tests have been proposed to identify publication bias in meta-analyses and to correct for it [34, 35, 36, 37], but these methods suffer from limitations and often require meticulous explicit modeling of each potential source of bias [37, 38]. Ioannidis and colleagues propose two methods to probe the robustness of meta-analyses that report a statistically significant effect at p<0.05 [20, 22, 39]. Both methods require no assumption about the sources of potential biases, but are not easy to apply in the context of our large database. However, these studies inspired us to adopt a simpler approach to identify, among nominally significant meta-analyses, those likely to retain their statistical significance with the inclusion of further studies.

We hypothesized that meta-analyses reporting highly significant effects (p<0.005) are more reliable than those reporting a p value between 0.05 and 0.005. This hypothesis is supported by theoretical considerations and by observational studies. Indeed, this more stringent p-value roughly corresponds to a Bayes factor of 50, which means than no more than one in 50 meta-analyses would report a false positive effect [23]. Moreover, Pereira and colleagues (2011) reported on 80 nominally significant (p<0.05) meta-analyses of clinical trials published in 2005 that were updated in 2010 with additional trials. Among them, four lost their statistical significance in 2010 and all four had a p value between 0.005 and 0.05 in 2005. Likewise, using a Bayesian approach, Ioannidis (2008) tested the effect of formal statistical significance on the credibility of 50 meta-analyses reporting a significant genetic association at p<0.05 [22]. Among them, 27 had a p value below 0.005 of which 26 were analyzed as providing at least a strong support to the association. In contrast, none of the 23 genetic associations with a reported p value between 0.5 and 0.005 provided a strong support.

Ioannidis listed small sample size and small “true” effect size as two major causes of replication failure [1]. However, he also highlighted other causes related to the methodological quality of the studies and to the general characteristics of each research field (i.e., the number of associations tested, the influence of financial interests and the strength of the competition between researchers) [1]. However, because these other causes of replication failure are difficult to identify and quantify, especially when considering a large database, they were not investigated in the present study.

We collected and analyzed the data of 663 initial studies and their corresponding largest studies and meta-analyses. We did not individually consider each of the 10,154 primary datasets included in these 663 meta-analyses. Therefore, while we provide direct data about the replication rate of initial and largest studies, we have only indirect and limited evidence regarding that of other primary studies in each subgroup. Our emphasis on initial studies, and especially on those reporting a significant effect, is justified by their influence on subsequent research efforts [15], and by the fact that they are much more echoed by the media than subsequent studies [40].

Our selection of 12 pathologies is partly arbitrary. Regarding neurological diseases we excluded stroke because it is also a cardiovascular disease, traumatic brain injury because it is accidental and autism, which is rather a psychiatric disorder. Thus, our selection of neurological diseases includes all four severe diseases exhibiting the highest prevalence rate [41]. Our selection of four psychiatric disorders is less systematic: it included two severe disorders (autism and schizophrenia) and two less severe and more prevalent disorders (ADHD and unipolar depression). Finally, our selection of four somatic diseases is even more arbitrary. We selected them because their biological causes and risk factors are still poorly elucidated and because they cover a range of prevalence similar to that of both other domains.

Our sample of initial studies was extracted from meta-analyses built from at least seven primary datasets. We did not investigate initial studies not followed by meta-analyses or included in less extensive meta-analyses. Therefore, our sample represents a tiny fraction of all studies published during the last three decades about the association of a risk factor with one of our 12 pathologies. Indeed, among a random sample of 259 biomedical studies reporting observational data, only 6.2% were included in a systematic meta-analysis [42]. Whether our sample of initial studies was representative regarding replication validity remains to be elucidated.

## Conclusion

The poor replication validity of initial biomedical studies has been put forward to recommend potential solutions that might improve it [4, 32, 43–45]. However, some of these recommendations might have unintended negative consequences [46]. Beside these recommendations, we would like to emphasize the opinion voiced by the Open Science Collaboration [21]: “If initial ideas were always correct, then there would hardly be a reason to conduct research in the first place. A healthy discipline will have many false starts as it confronts the limits of the present understanding”. Nevertheless, the differences between biological psychiatry, neurology and somatic diseases suggest that there is room for improvement at least in some subdomains.

An unrecognized consequence of the low reliability of initial studies is not related to the scientific process of uncertainty reduction *per se*, but to the media coverage of scientific findings. We showed with the case of ADHD that newspapers preferentially echo initial findings and almost never inform the public when they are refuted or strongly attenuated although the majority of them are [40]. Therefore, the present estimates of the replication rates of initial studies in various domains and subdomains might be especially useful for journalists and for scientists when they interact with journalists. Initial findings should always be described as tentative, uncertain and requiring replication when reported in the scientific literature, the academic press releases and the media.

## Supporting Information

S1 Fig Twelve flow diagrams. (DOCX)

S1 File Raw data about initial studies, largest studies and meta-analyses for 12 pathologies. (XLSX)

S1 Text References of the 333 articles describing the 663 meta-analyses. (DOCX)

S2 Text References of the 485 articles describing the 663 initial observations. (DOCX)

S3 Text References of the 447 articles describing the 663 largest observations. (DOCX)
