# Supplementary material for: Replication Validity of Initial Association Studies: A Comparison between Psychiatry, Neurology and Four Somatic Diseases
Source: PLoS One. 2016 Jun 23;11(6):e0158064. doi: 10.1371/journal.pone.0158064 (PMC4919034; doi:10.1371/journal.pone.0158064)
Supplement: S1 Fig — (DOCX) [file pone.0158064.s001.docx]

**Fig S1**. Flow diagram ADHD

Records excluded

(N=75)

Intervention/treatment study (N=53)

Not about ADHD/relevant (N= 12)

Not enough datasets (N=1)

ADHD is the risk factor (N= 4)

Not expressed as d or OR/RR (N=5)

Records identified through PubMed search

(N=118)

Records screened

(N=118)

Full text articles excluded, with reasons

(N=23)

Effect size not reported as d or OR/RR (N=7)

Incomplete data (N=5)

<7 datasets (N=9)

See more recent article about the same topic (N=2)

Full text articles assessed for eligibility (N= 43)

INCLUDED

Full text articles (N=20)

Meta-analyses (N= 40)

**Fig S2**. Flow diagram Autism (ASD)

Records excluded

(N=35)

Intervention/treatment study (N=27)

Not about Autism/relevant (N= 5)

Autism is the risk factor (N= 3)

Records identified through PubMed search

(N=71)

Records screened

(N=71)

Full text articles excluded, with reasons

(N=23)

Effect size not reported as d or OR/RR (N=13)

Incomplete data (N=3)

<7 datasets (N=7)

Full text articles assessed for eligibility (N= 36)

INCLUDED

Full text articles (N=13)

Meta-analyses (N= 24)

**Fig S3**. Flow diagram Major Depressive Disorder (MDD)

Records excluded

(N=439)

Intervention/treatment study /diagnosis (N=355)

Not about MDD/relevant (N= 59)

Not enough datasets/incomplete data (N=6)

MDD is the risk factor (N= 14)

Not expressed as d or OR/RR (N=5)

Records identified through PubMed search

(N=553)

Records screened

(N=553)

Full text articles assessed for eligibility (N= 114)

Full text articles excluded, with reasons

(N=86)

Effect size not reported as d or OR/RR (N=19)

Incomplete data (N=15)

<7 datasets (N=27)

See more recent article about the same topic (N=13)

Not relevant (N=5)

No control group (N=4)

Not a meta-analysis (N=3)

INCLUDED

Full text articles (N=28)

Meta-analyses (N= 53)

**Fig S4**. Flow diagram Schizophrenia (SCZ)

Records excluded

(N=256)

Intervention/treatment study /diagnosis (N=171)

Not about SCH/relevant (N= 67)

SCH is the risk factor (N= 17)

Not expressed as d or OR/RR (N=1)

Records identified through PubMed search

(N=454)

Records screened

(N=454)

Full text articles excluded, with reasons

(N=139)

Effect size not reported as d or OR/RR (N=37)

Incomplete data (N=19)

<7 datasets (N=40)

See more recent article about the same topic (N=19)

Not relevant (N=8)

No control group (N=6)

Not a meta-analysis (N=4)

Qualitative measure (N=4)

Inconsistencies (N=2)

Full text articles assessed for eligibility (N= 198)

INCLUDED

Full text articles (N=59)

Meta-analyses (N= 203)

**Fig S5**. Flow diagram Alzheimer’s disease (AD)

Records excluded

(N=80)

Intervention/treatment study /diagnosis (N=67)

Not about AD/relevant (N= 11)

AD is the risk factor (N= 2)

Records identified through PubMed search

(N=197)

Records screened

(N=197)

Full text articles excluded, with reasons

(N=82)

Effect size not reported as d or OR/RR (N=12)

Incomplete data (N=15)

<7 datasets (N=30)

See more recent article about the same topic (N=7)

Not relevant (N=7)

No control group (N=4)

Not a meta-analysis (N=4)

Qualitative measure (N=1)

Inconsistencies (N=2)

Full text articles assessed for eligibility (N= 117)

INCLUDED

Full text articles (N=35)

Meta-analyses (N= 50)

**Fig S6**. Flow diagram Epilepsy (Epi)

Records identified through PubMed search

(N=147)

Records excluded

(N=118)

Intervention/treatment study (N=61)

Not about Epilepsy/relevant (N= 51)

Epilepsy is the risk factor (N= 6)

Records screened

(N=147)

Full text articles excluded, with reasons

(N=17)

Effect size not reported as d or OR/RR (N=6)

<7 datasets (N=9)

Not relevant (N=1)

Not a meta-analysis (N=1)

Full text articles assessed for eligibility (N= 29)

INCLUDED

Full text articles (N=12)

Meta-analyses (N= 15)

**Fig S7**. Flow diagram Multiple Sclerosis (MS)

Records identified through PubMed search

(N=137)

Records excluded

(N=93)

Intervention/treatment study /diagnosis (N=51)

Not about MS/relevant (N= 35)

MS is the risk factor (N= 7)

Records screened

(N=137)

Full text articles excluded, with reasons

(N=26)

Effect size not reported as d or OR/RR (N=3)

Incomplete data (N=3)

<7 datasets (N=11)

See more recent article about the same topic (N=4)

Not relevant (N=3)

Not a meta-analysis (N=1)

Inconsistencies (N=1)

Full text articles assessed for eligibility (N= 44)

INCLUDED

Full text articles (N=18)

Meta-analyses (N= 37)

**Fig S8**. Flow diagram Parkinson’s Disease (PD)

Records excluded

(N=65)

Intervention/treatment study (N=43)

Not about PD/relevant (N= 14)

PD is the risk factor (N= 8)

Records identified through PubMed search

(N=139)

Records screened

(N=139)

Full text articles excluded, with reasons

(N=54)

Effect size not reported as d or OR/RR (N=8)

Incomplete data (N=4)

<7 datasets (N=27)

See more recent article about the same topic (N=10)

Not a meta-analysis (N=5)

Full text articles assessed for eligibility (N= 74)

INCLUDED

Full text articles (N=20)

Meta-analyses (N= 57)

**Fig S9**. Flow diagram Breast Cancer (BC)

Records excluded

(N=466)

Intervention/treatment study /diagnosis (N=184)

Not about BC/relevant (N= 242)

BC is the risk factor (N= 38)

Not a meta-analysis (N=2)

Records identified through PubMed search

(N=811)

Records screened

(N=811)

Full text articles excluded, with reasons

(N=256)

Effect size not reported as d or OR/RR (N=2)

Incomplete data (N=94)

<7 datasets (N=95)

See more recent article about the same topic (N=55)

Not relevant (N=1)

No control group (N=2)

Not a meta-analysis (N=3)

Inconsistencies (N=4)

Full text articles assessed for eligibility (N= 345)

INCLUDED

Full text articles (N=89)

Meta-analyses (N= 114)

**Fig S10**. Flow diagram Glaucoma (GLAU)

Records excluded

(N=56)

Intervention/treatment study /diagnosis (N=50)

Not about GLAU/relevant (N= 4)

GLAU is the risk factor (N= 2)

Records identified through PubMed search

(N=75)

Records screened

(N=75)

Full text articles excluded, with reasons

(N=10)

<7 datasets (N=7)

See more recent article about the same topic (N=1)

Not relevant (N=1)

Not a meta-analysis (N=1)

Full text articles assessed for eligibility (N= 19)

INCLUDED

Full text articles (N=9)

Meta-analyses (N= 21)

**Fig S11**. Flow diagram Psoriasis (PSO)

Records excluded

(N=59)

Intervention/treatment study (N=40)

Not about PSO/relevant (N= 14)

PSO is the risk factor (N= 5)

Records identified through PubMed search

(N=81)

Records screened

(N=81)

Full text articles excluded, with reasons

(N=14)

Incomplete data (N=1)

<7 datasets (N=13)

Full text articles assessed for eligibility (N= 22)

INCLUDED

Full text articles (N=8)

Meta-analyses (N= 15)

**Fig S12**. Flow diagram Rheumatoid Arthritis (RA)

Records identified through PubMed search (N=285)

(N=285)

Records excluded

(N=203)

Intervention/treatment study /diagnosis (N=154)

Not about RA/relevant (N= 34)

RA is the risk factor (N= 15)

Records screened

(N=285)

Full text articles excluded, with reasons

(N=60)

Incomplete data (N=3)

<7 datasets (N=31)

See more recent article about the same topic (N=17)

Not relevant (N=9)

Full text articles assessed for eligibility (N= 82)

INCLUDED

Full text articles (N=22)

Meta-analyses (N= 34)
