# Supplementary material for: Replication Validity of Initial Association Studies: A Comparison between Psychiatry, Neurology and Four Somatic Diseases
Source: PLoS One. 2016 Jun 23;11(6):e0158064. doi: 10.1371/journal.pone.0158064 (PMC4919034; doi:10.1371/journal.pone.0158064)
Supplement: S1 Text — (DOCX) [file pone.0158064.s003.docx]

**References for meta-analysis**

**ADHD**

Arns M, Conners CK, Kraemer HC (2013) A decade of EEG Theta/Beta Ratio Research in ADHD: a meta-analysis. Journal of attention disorders 17:374-383.

Chamberlain SR, Robbins TW, Winder-Rhodes S, Muller U, Sahakian BJ, Blackwell AD, Barnett JH (2011) Translational approaches to frontostriatal dysfunction in attention-deficit/hyperactivity disorder using a computerized neuropsychological battery. Biological psychiatry 69:1192-1203.

Cortese b S, Angriman M, Lecendreux M, Konofal E (2012) Iron and attention deficit/hyperactivity disorder: What is the empirical evidence so far? A systematic review of the literature. Expert review of neurotherapeutics 12:1227-1240.

Cortese S, Faraone SV, Konofal E, Lecendreux M (2009) Sleep in children with attention-deficit/hyperactivity disorder: meta-analysis of subjective and objective studies. Journal of the American Academy of Child and Adolescent Psychiatry 48:894-908.

Forero DA, Arboleda GH, Vasquez R, Arboleda H (2009) Candidate genes involved in neural plasticity and the risk for attention-deficit hyperactivity disorder: a meta-analysis of 8 common variants. Journal of psychiatry & neuroscience : JPN 34:361-366.

Frodl T, Skokauskas N (2012) Meta-analysis of structural MRI studies in children and adults with attention deficit hyperactivity disorder indicates treatment effects. Acta psychiatrica Scandinavica 125:114-126.

Fusar-Poli P, Rubia K, Rossi G, Sartori G, Balottin U (2012) Striatal dopamine transporter alterations in ADHD: pathophysiology or adaptation to psychostimulants? A meta-analysis. The American journal of psychiatry 169:264-272.

Geburek AJ, Rist F, Gediga G, Stroux D, Pedersen A (2013) Electrophysiological indices of error monitoring in juvenile and adult attention deficit hyperactivity disorder (ADHD)--a meta-analytic appraisal. International journal of psychophysiology : official journal of the International Organization of Psychophysiology 87:349-362.

Gizer IR, Ficks C, Waldman ID (2009) Candidate gene studies of ADHD: a meta-analytic review. Human genetics 126:51-90.

Hasson R, Fine JG (2012) Gender differences among children with ADHD on continuous performance tests: a meta-analytic review. Journal of attention disorders 16:190-198.

Hemmi MH, Wolke D, Schneider S (2011) Associations between problems with crying, sleeping and/or feeding in infancy and long-term behavioural outcomes in childhood: a meta-analysis. Archives of disease in childhood 96:622-629.

Kasper LJ, Alderson RM, Hudec KL (2012) Moderators of working memory deficits in children with attention-deficit/hyperactivity disorder (ADHD): a meta-analytic review. Clinical psychology review 32:605-617.

Kofler MJ, Rapport MD, Alderson RM (2008) Quantifying ADHD classroom inattentiveness, its moderators, and variability: a meta-analytic review. Journal of child psychology and psychiatry, and allied disciplines 49:59-69.

Lipszyc J, Schachar R (2010) Inhibitory control and psychopathology: a meta-analysis of studies using the stop signal task. Journal of the International Neuropsychological Society : JINS 16:1064-1076.

Metin B, Roeyers H, Wiersema JR, van der Meere J, Sonuga-Barke E (2012) A meta-analytic study of event rate effects on Go/No-Go performance in attention-deficit/hyperactivity disorder. Biological psychiatry 72:990-996.

Nigg JT, Lewis K, Edinger T, Falk M (2012) Meta-analysis of attention-deficit/hyperactivity disorder or attention-deficit/hyperactivity disorder symptoms, restriction diet, and synthetic food color additives. Journal of the American Academy of Child and Adolescent Psychiatry 51:86-97 e88.

Pauli-Pott U, Becker K (2011) Neuropsychological basic deficits in preschoolers at risk for ADHD: a meta-analysis. Clinical psychology review 31:626-637.

Scassellati C, Bonvicini C, Faraone SV, Gennarelli M (2012) Biomarkers and attention-deficit/hyperactivity disorder: a systematic review and meta-analyses. Journal of the American Academy of Child and Adolescent Psychiatry 51:1003-1019 e1020.

Smith TF (2010) Meta-analysis of the heterogeneity in association of DRD4 7-repeat allele and AD/HD: stronger association with AD/HD combined type. American journal of medical genetics Part B, Neuropsychiatric genetics : the official publication of the International Society of Psychiatric Genetics 153B:1189-1199.

Wu J, Xiao H, Sun H, Zou L, Zhu LQ (2012) Role of dopamine receptors in ADHD: a systematic meta-analysis. Molecular neurobiology 45:605-620.

**Autism**

Amin SB, Smith T, Wang H (2011) Is neonatal jaundice associated with Autism Spectrum Disorders: a systematic review. Journal of autism and developmental disorders 41:1455-1463.

Aoki Y, Kasai K, Yamasue H (2012) Age-related change in brain metabolite abnormalities in autism: a meta-analysis of proton magnetic resonance spectroscopy studies. Translational psychiatry 2:e69.

Ben-Sasson A, Hen L, Fluss R, Cermak SA, Engel-Yeger B, Gal E (2009) A meta-analysis of sensory modulation symptoms in individuals with autism spectrum disorders. Journal of autism and developmental disorders 39:1-11.

Fournier KA, Hass CJ, Naik SK, Lodha N, Cauraugh JH (2010) Motor coordination in autism spectrum disorders: a synthesis and meta-analysis. Journal of autism and developmental disorders 40:1227-1240.

Frazier TW, Hardan AY (2009) A meta-analysis of the corpus callosum in autism. Biological psychiatry 66:935-941.

Frustaci A, Neri M, Cesario A, Adams JB, Domenici E, Dalla Bernardina B, Bonassi S (2012) Oxidative stress-related biomarkers in autism: systematic review and meta-analyses. Free radical biology & medicine 52:2128-2141.

Huang CH, Santangelo SL (2008) Autism and serotonin transporter gene polymorphisms: a systematic review and meta-analysis. American journal of medical genetics Part B, Neuropsychiatric genetics : the official publication of the International Society of Psychiatric Genetics 147B:903-913.

Hultman CM, Sandin S, Levine SZ, Lichtenstein P, Reichenberg A (2011) Advancing paternal age and risk of autism: new evidence from a population-based study and a meta-analysis of epidemiological studies. Molecular psychiatry 16:1203-1212.

Ipser JC, Syal S, Bentley J, Adnams CM, Steyn B, Stein DJ (2012) 1H-MRS in autism spectrum disorders: a systematic meta-analysis. Metabolic brain disease 27:275-287.

Ozgen HM, Hop JW, Hox JJ, Beemer FA, van Engeland H (2010) Minor physical anomalies in autism: a meta-analysis. Molecular psychiatry 15:300-307.

Sandin S, Hultman CM, Kolevzon A, Gross R, MacCabe JH, Reichenberg A (2012) Advancing maternal age is associated with increasing risk for autism: a review and meta-analysis. Journal of the American Academy of Child and Adolescent Psychiatry 51:477-486 e471.

Song RR, Zou L, Zhong R, Zheng XW, Zhu BB, Chen W, Liu L, Miao XP (2011) An integrated meta-analysis of two variants in HOXA1/HOXB1 and their effect on the risk of autism spectrum disorders. PloS one 6:e25603.

Stanfield AC, McIntosh AM, Spencer MD, Philip R, Gaur S, Lawrie SM (2008) Towards a neuroanatomy of autism: a systematic review and meta-analysis of structural magnetic resonance imaging studies. European psychiatry : the journal of the Association of European Psychiatrists 23:289-299.

**Major Depressive Disorder**

Almeida OP, McCaul K, Hankey GJ, Norman P, Jamrozik K, Flicker L (2008) Homocysteine and depression in later life. Archives of general psychiatry 65:1286-1294.

Arnone D, McIntosh AM, Ebmeier KP, Munafo MR, Anderson IM (2012) Magnetic resonance imaging studies in unipolar depression: systematic review and meta-regression analyses. European neuropsychopharmacology : the journal of the European College of Neuropsychopharmacology 22:1-16.

Bocchio-Chiavetto L, Bagnardi V, Zanardini R, Molteni R, Nielsen MG, Placentino A, Giovannini C, Rillosi L, Ventriglia M, Riva MA, Gennarelli M (2010) Serum and plasma BDNF levels in major depression: a replication study and meta-analyses. The world journal of biological psychiatry : the official journal of the World Federation of Societies of Biological Psychiatry 11:763-773.

Bora E, Harrison BJ, Davey CG, Yucel M, Pantelis C (2012) Meta-analysis of volumetric abnormalities in cortico-striatal-pallidal-thalamic circuits in major depressive disorder. Psychological medicine 42:671-681.

Bylsma LM, Morris BH, Rottenberg J (2008) A meta-analysis of emotional reactivity in major depressive disorder. Clinical psychology review 28:676-691.

Chang-Quan b H, Zheng-Rong W, Yong-Hong L, Yi-Zhou X, Qing-Xiu L (2010) Education and risk for late life depression: a meta-analysis of published literature. International journal of psychiatry in medicine 40:109-124.

Chang-Quan H, Xue-Mei Z, Bi-Rong D, Zhen-Chan L, Ji-Rong Y, Qing-Xiu L (2010) Health status and risk for depression among the elderly: a meta-analysis of published literature. Age and ageing 39:23-30.

Clarke H, Flint J, Attwood AS, Munafo MR (2010) Association of the 5- HTTLPR genotype and unipolar depression: a meta-analysis. Psychological medicine 40:1767-1778.

Cole J, Costafreda SG, McGuffin P, Fu CH (2011) Hippocampal atrophy in first episode depression: a meta-analysis of magnetic resonance imaging studies. Journal of affective disorders 134:483-487.

Demenescu LR, Kortekaas R, den Boer JA, Aleman A (2010) Impaired attribution of emotion to facial expressions in anxiety and major depression. PloS one 5:e15058.

Gyekis JP, Yu W, Dong S, Wang H, Qian J, Kota P, Yang J (2013) No association of genetic variants in BDNF with major depression: a meta- and gene-based analysis. American journal of medical genetics Part B, Neuropsychiatric genetics : the official publication of the International Society of Psychiatric Genetics 162B:61-70.

Hiles SA, Baker AL, de Malmanche T, Attia J (2012) A meta-analysis of differences in IL-6 and IL-10 between people with and without depression: exploring the causes of heterogeneity. Brain, behavior, and immunity 26:1180-1188.

Kempton MJ, Salvador Z, Munafo MR, Geddes JR, Simmons A, Frangou S, Williams SC (2011) Structural neuroimaging studies in major depressive disorder. Meta-analysis and comparison with bipolar disorder. Archives of general psychiatry 68:675-690.

Kishi T, Yoshimura R, Fukuo Y, Okochi T, Matsunaga S, Umene-Nakano W, Nakamura J, Serretti A, Correll CU, Kane JM, Iwata N (2013) The serotonin 1A receptor gene confer susceptibility to mood disorders: results from an extended meta-analysis of patients with major depression and bipolar disorder. European archives of psychiatry and clinical neuroscience 263:105-118.

Knorr U, Vinberg M, Kessing LV, Wetterslev J (2010) Salivary cortisol in depressed patients versus control persons: a systematic review and meta-analysis. Psychoneuroendocrinology 35:1275-1286.

Liu X, Li L, Xiao J, Yang J, Jiang X (2013) Abnormalities of autobiographical memory of patients with depressive disorders: a meta-analysis. Psychology and psychotherapy 86:353-373.

Liu Y, Ho RC, Mak A (2012) Interleukin (IL)-6, tumour necrosis factor alpha (TNF-alpha) and soluble interleukin-2 receptors (sIL-2R) are elevated in patients with major depressive disorder: a meta-analysis and meta-regression. Journal of affective disorders 139:230-239.

Lopez-Duran NL, Kovacs M, George CJ (2009) Hypothalamic-pituitary-adrenal axis dysregulation in depressed children and adolescents: a meta-analysis. Psychoneuroendocrinology 34:1272-1283.

Lopez-Leon S, Janssens AC, Gonzalez-Zuloeta Ladd AM, Del-Favero J, Claes SJ, Oostra BA, van Duijn CM (2008) Meta-analyses of genetic studies on major depressive disorder. Molecular psychiatry 13:772-785.

Luykx JJ, Laban KG, van den Heuvel MP, Boks MP, Mandl RC, Kahn RS, Bakker SC (2012) Region and state specific glutamate downregulation in major depressive disorder: a meta-analysis of (1)H-MRS findings. Neuroscience and biobehavioral reviews 36:198-205.

Micco JA, Henin A, Mick E, Kim S, Hopkins CA, Biederman J, Hirshfeld-Becker DR (2009) Anxiety and depressive disorders in offspring at high risk for anxiety: a meta-analysis. Journal of anxiety disorders 23:1158-1164.

Munafo MR, Brown SM, Hariri AR (2008) Serotonin transporter (5-HTTLPR) genotype and amygdala activation: a meta-analysis. Biological psychiatry 63:852-857.

Paulson JF, Bazemore SD (2010) Prenatal and postpartum depression in fathers and its association with maternal depression: a meta-analysis. JAMA : the journal of the American Medical Association 303:1961-1969.

Risch N, Herrell R, Lehner T, Liang KY, Eaves L, Hoh J, Griem A, Kovacs M, Ott J, Merikangas KR (2009) Interaction between the serotonin transporter gene (5-HTTLPR), stressful life events, and risk of depression: a meta-analysis. JAMA : the journal of the American Medical Association 301:2462-2471.

Wagner S, Doering B, Helmreich I, Lieb K, Tadic A (2012) A meta-analysis of executive dysfunctions in unipolar major depressive disorder without psychotic symptoms and their changes during antidepressant treatment. Acta psychiatrica Scandinavica 125:281-292.

Wojcik W, Lee W, Colman I, Hardy R, Hotopf M (2013) Foetal origins of depression? A systematic review and meta-analysis of low birth weight and later depression. Psychological medicine 43:1-12.

Wu Y, Wang X, Shen X, Tan Z, Yuan Y (2012) The I/D polymorphism of angiotensin-converting enzyme gene in major depressive disorder and therapeutic outcome: a case-control study and meta-analysis. Journal of affective disorders 136:971-978.

Wu YL, Ding XX, Sun YH, Yang HY, Chen J, Zhao X, Jiang YH, Lv XL, Wu ZQ (2013) Association between MTHFR C677T polymorphism and depression: An updated meta-analysis of 26 studies. Progress in neuro-psychopharmacology & biological psychiatry 46:78-85.

**Schizophrenia**

Adriano F, Caltagirone C, Spalletta G (2012) Hippocampal volume reduction in first-episode and chronic schizophrenia: a review and meta-analysis. The Neuroscientist : a review journal bringing neurobiology, neurology and psychiatry 18:180-200.

Allen NC, Bagade S, McQueen MB, Ioannidis JP, Kavvoura FK, Khoury MJ, Tanzi RE, Bertram L (2008) Systematic meta-analyses and field synopsis of genetic association studies in schizophrenia: the SzGene database. Nature genetics 40:827-834.

Arias I, Sorlozano A, Villegas E, de Dios Luna J, McKenney K, Cervilla J, Gutierrez B, Gutierrez J (2012) Infectious agents associated with schizophrenia: a meta-analysis. Schizophrenia research 136:128-136.

Bora b E, Yucel M, Pantelis C (2009) Theory of mind impairment in schizophrenia: meta-analysis. Schizophrenia research 109:1-9.

Bourque F, van der Ven E, Malla A (2011) A meta-analysis of the risk for psychotic disorders among first- and second-generation immigrants. Psychological medicine 41:897-910.

Chan RC, Li H, Cheung EF, Gong QY (2010) Impaired facial emotion perception in schizophrenia: a meta-analysis. Psychiatry research 178:381-390.

Cohen AS, Brown LA, Auster TL (2012) Olfaction, "olfiction," and the schizophrenia-spectrum: an updated meta-analysis on identification and acuity. Schizophrenia research 135:152-157.

De Peri L, Crescini A, Deste G, Fusar-Poli P, Sacchetti E, Vita A (2012) Brain structural abnormalities at the onset of schizophrenia and bipolar disorder: a meta-analysis of controlled magnetic resonance imaging studies. Current pharmaceutical design 18:486-494.

Dickson H, Laurens KR, Cullen AE, Hodgins S (2012) Meta-analyses of cognitive and motor function in youth aged 16 years and younger who subsequently develop schizophrenia. Psychological medicine 42:743-755.

Doughty OJ, Done DJ (2009) Is semantic memory impaired in schizophrenia? A systematic review and meta-analysis of 91 studies. Cognitive neuropsychiatry 14:473-509.

Esterberg ML, Trotman HD, Holtzman C, Compton MT, Walker EF (2010) The impact of a family history of psychosis on age-at-onset and positive and negative symptoms of schizophrenia: a meta-analysis. Schizophrenia research 120:121-130.

Ferreira-Santos F, Silveira C, Almeida PR, Palha A, Barbosa F, Marques-Teixeira J (2012) The auditory P200 is both increased and reduced in schizophrenia? A meta-analytic dissociation of the effect for standard and target stimuli in the oddball task. Clinical neurophysiology : official journal of the International Federation of Clinical Neurophysiology 123:1300-1308.

Fioravanti M, Bianchi V, Cinti ME (2012) Cognitive deficits in schizophrenia: an updated metanalysis of the scientific evidence. BMC psychiatry 12:64.

Forbes NF, Carrick LA, McIntosh AM, Lawrie SM (2009) Working memory in schizophrenia: a meta-analysis. Psychological medicine 39:889-905.

Fusar-Poli b P, Meyer-Lindenberg A (2013) Striatal presynaptic dopamine in schizophrenia, part II: meta-analysis of [(18)F/(11)C]-DOPA PET studies. Schizophrenia bulletin 39:33-42.

Fusar-Poli P, Meyer-Lindenberg A (2013) Striatal presynaptic dopamine in schizophrenia, Part I: meta-analysis of dopamine active transporter (DAT) density. Schizophrenia bulletin 39:22-32.

Golembo-Smith S, Walder DJ, Daly MP, Mittal VA, Kline E, Reeves G, Schiffman J (2012) The presentation of dermatoglyphic abnormalities in schizophrenia: a meta-analytic review. Schizophrenia research 142:1-11.

Green MJ, Matheson SL, Shepherd A, Weickert CS, Carr VJ (2011) Brain-derived neurotrophic factor levels in schizophrenia: a systematic review with meta-analysis. Molecular psychiatry 16:960-972.

Haijma SV, Van Haren N, Cahn W, Koolschijn PC, Hulshoff Pol HE, Kahn RS (2013) Brain volumes in schizophrenia: a meta-analysis in over 18 000 subjects. Schizophrenia bulletin 39:1129-1138.

Hoen WP, Lijmer JG, Duran M, Wanders RJ, van Beveren NJ, de Haan L (2013) Red blood cell polyunsaturated fatty acids measured in red blood cells and schizophrenia: a meta-analysis. Psychiatry research 207:1-12.

Howes OD, Kambeitz J, Kim E, Stahl D, Slifstein M, Abi-Dargham A, Kapur S (2012) The nature of dopamine dysfunction in schizophrenia and what this means for treatment. Archives of general psychiatry 69:776-786.

Huang J, Xu T, Chan RC (2011) Do patients with schizophrenia have a general or specific deficit in the perception of social threat? A meta-analytic study. Psychiatry research 185:1-8.

Kawashima K, Ikeda M, Kishi T, Kitajima T, Yamanouchi Y, Kinoshita Y, Okochi T, Aleksic B, Tomita M, Okada T, Kunugi H, Inada T, Ozaki N, Iwata N (2009) BDNF is not associated with schizophrenia: data from a Japanese population study and meta-analysis. Schizophrenia research 112:72-79.

Kim SG, Song JY, Joo EJ, Jeong SH, Kim SH, Lee KY, Lee NY, Ahn YM, Kim YS, Roh MS (2011) No association of functional polymorphisms in methlylenetetrahydrofolate reductase and the risk and minor physical anomalies of schizophrenia in Korean population. Journal of Korean medical science 26:1356-1363.

Kraguljac NV, Reid M, White D, Jones R, den Hollander J, Lowman D, Lahti AC (2012) Neurometabolites in schizophrenia and bipolar disorder - a systematic review and meta-analysis. Psychiatry research 203:111-125.

Liu ZW, Liu JL, An Y, Zhang L, Wang YM (2012) Association between Ser311Cys polymorphism in the dopamine D2 receptor gene and schizophrenia risk: a meta-analysis in Asian populations. Genetics and molecular research : GMR 11:261-270.

Maia-de-Oliveira JP, Trzesniak C, Oliveira IR, Kempton MJ, Rezende TM, Iego S, Baker GB, Dursun SM, Machado-de-Sousa JP, Hallak JE (2012) Nitric oxide plasma/serum levels in patients with schizophrenia: a systematic review and meta-analysis. Revista brasileira de psiquiatria (Sao Paulo, Brazil : 1999) 34 Suppl 2:S149-155.

Marsman A, van den Heuvel MP, Klomp DW, Kahn RS, Luijten PR, Hulshoff Pol HE (2013) Glutamate in schizophrenia: a focused review and meta-analysis of (1)H-MRS studies. Schizophrenia bulletin 39:120-129.

Matheson SL, Shepherd AM, Pinchbeck RM, Laurens KR, Carr VJ (2013) Childhood adversity in schizophrenia: a systematic meta-analysis. Psychological medicine 43:225-238.

Miller B, Messias E, Miettunen J, Alaraisanen A, Jarvelin MR, Koponen H, Rasanen P, Isohanni M, Kirkpatrick B (2011) Meta-analysis of paternal age and schizophrenia risk in male versus female offspring. Schizophrenia bulletin 37:1039-1047.

Molloy C, Conroy RM, Cotter DR, Cannon M (2011) Is traumatic brain injury a risk factor for schizophrenia? A meta-analysis of case-controlled population-based studies. Schizophrenia bulletin 37:1104-1110.

Munafo MR, Attwood AS, Flint J (2008) Neuregulin 1 genotype and schizophrenia. Schizophrenia bulletin 34:9-12.

Neelam K, Garg D, Marshall M (2011) A systematic review and meta-analysis of neurological soft signs in relatives of people with schizophrenia. BMC psychiatry 11:139.

Nieto RG, Castellanos FX (2011) A meta-analysis of neuropsychological functioning in patients with early onset schizophrenia and pediatric bipolar disorder. Journal of clinical child and adolescent psychology : the official journal for the Society of Clinical Child and Adolescent Psychology, American Psychological Association, Division 53 40:266-280.

Ohi K, Hashimoto R, Yasuda Y, Yoshida T, Takahashi H, Iike N, Iwase M, Kamino K, Ishii R, Kazui H, Fukumoto M, Takamura H, Yamamori H, Azechi M, Ikezawa K, Tanimukai H, Tagami S, Morihara T, Okochi M, Yamada K, Numata S, Ikeda M, Tanaka T, Kudo T, Ueno S, Yoshikawa T, Ohmori T, Iwata N, Ozaki N, Takeda M (2010) The chitinase 3-like 1 gene and schizophrenia: evidence from a multi-center case-control study and meta-analysis. Schizophrenia research 116:126-132.

Okochi T, Ikeda M, Kishi T, Kawashima K, Kinoshita Y, Kitajima T, Yamanouchi Y, Tomita M, Inada T, Ozaki N, Iwata N (2009) Meta-analysis of association between genetic variants in COMT and schizophrenia: an update. Schizophrenia research 110:140-148.

Olabi B, Ellison-Wright I, McIntosh AM, Wood SJ, Bullmore E, Lawrie SM (2011) Are there progressive brain changes in schizophrenia? A meta-analysis of structural magnetic resonance imaging studies. Biological psychiatry 70:88-96.

Patel S, Mahon K, Wellington R, Zhang J, Chaplin W, Szeszko PR (2011) A meta-analysis of diffusion tensor imaging studies of the corpus callosum in schizophrenia. Schizophrenia research 129:149-155.

Potvin b S, Marchand S (2008) Hypoalgesia in schizophrenia is independent of antipsychotic drugs: a systematic quantitative review of experimental studies. Pain 138:70-78.

Potvin c S, Stip E, Sepehry AA, Gendron A, Bah R, Kouassi E (2008) Inflammatory cytokine alterations in schizophrenia: a systematic quantitative review. Biological psychiatry 63:801-808.

Saetre P, Lundmark P, Wang A, Hansen T, Rasmussen HB, Djurovic S, Melle I, Andreassen OA, Werge T, Agartz I, Hall H, Terenius L, Jonsson EG (2010) The tryptophan hydroxylase 1 (TPH1) gene, schizophrenia susceptibility, and suicidal behavior: a multi-centre case-control study and meta-analysis. American journal of medical genetics Part B, Neuropsychiatric genetics : the official publication of the International Society of Psychiatric Genetics 153B:387-396.

Savla GN, Vella L, Armstrong CC, Penn DL, Twamley EW (2013) Deficits in domains of social cognition in schizophrenia: a meta-analysis of the empirical evidence. Schizophrenia bulletin 39:979-992.

Schroeter ML, Abdul-Khaliq H, Krebs M, Diefenbacher A, Blasig IE (2009) Neuron-specific enolase is unaltered whereas S100B is elevated in serum of patients with schizophrenia--original research and meta-analysis. Psychiatry research 167:66-72.

Schumacher J, Laje G, Abou Jamra R, Becker T, Muhleisen TW, Vasilescu C, Mattheisen M, Herms S, Hoffmann P, Hillmer AM, Georgi A, Herold C, Schulze TG, Propping P, Rietschel M, McMahon FJ, Nothen MM, Cichon S (2009) The DISC locus and schizophrenia: evidence from an association study in a central European sample and from a meta-analysis across different European populations. Human molecular genetics 18:2719-2727.

Shepherd AM, Matheson SL, Laurens KR, Carr VJ, Green MJ (2012) Systematic meta-analysis of insula volume in schizophrenia. Biological psychiatry 72:775-784.

Shi b J, Gershon ES, Liu C (2008) Genetic associations with schizophrenia: meta-analyses of 12 candidate genes. Schizophrenia research 104:96-107.

Shi J, Badner JA, Gershon ES, Liu C (2008) Allelic association of G72/G30 with schizophrenia and bipolar disorder: a comprehensive meta-analysis. Schizophrenia research 98:89-97.

Siegert RJ, Weatherall M, Bell EM (2008) Is implicit sequence learning impaired in schizophrenia? A meta-analysis. Brain and cognition 67:351-359.

Trzesniak C, Kempton MJ, Busatto GF, de Oliveira IR, Galvao-de Almeida A, Kambeitz J, Ferrari MC, Filho AS, Chagas MH, Zuardi AW, Hallak JE, McGuire PK, Crippa JA (2011) Adhesio interthalamica alterations in schizophrenia spectrum disorders: A systematic review and meta-analysis. Progress in neuro-psychopharmacology & biological psychiatry 35:877-886.

Utsunomiya K, Shinkai T, De Luca V, Hwang R, Sakata S, Fukunaka Y, Chen HI, Ohmori O, Nakamura J (2008) Genetic association between the dopamine D3 gene polymorphism (Ser9Gly) and schizophrenia in Japanese populations: evidence from a case-control study and meta-analysis. Neuroscience letters 444:161-165.

Wang K, Cheung EF, Gong QY, Chan RC (2011) Semantic processing disturbance in patients with schizophrenia: a meta-analysis of the N400 component. PloS one 6:e25435.

Watanabe Y, Nunokawa A, Kaneko N, Shibuya M, Egawa J, Someya T (2012) Supportive evidence for the association between the Gln2Pro polymorphism in the SIGMAR1 gene and schizophrenia in the Japanese population: a case-control study and an updated meta-analysis. Schizophrenia research 141:279-280.

Waters F, Woodward T, Allen P, Aleman A, Sommer I (2012) Self-recognition deficits in schizophrenia patients with auditory hallucinations: a meta-analysis of the literature. Schizophrenia bulletin 38:741-750.

Westerhausen R, Kompus K, Hugdahl K (2011) Impaired cognitive inhibition in schizophrenia: a meta-analysis of the Stroop interference effect. Schizophrenia research 133:172-181.

Westerhausen R, Kompus K, Hugdahl K (2013) Unaffected control of distractor interference in schizophrenia: a meta-analysis of incompatibility slowing in flanker tasks. Journal of psychiatric research 47:246-251.

Xu M, St Clair D, He L (2010) Testing for genetic association between the ZDHHC8 gene locus and susceptibility to schizophrenia: An integrated analysis of multiple datasets. American journal of medical genetics Part B, Neuropsychiatric genetics : the official publication of the International Society of Psychiatric Genetics 153B:1266-1275.

Xu T, Chan RC, Compton MT (2011) Minor physical anomalies in patients with schizophrenia, unaffected first-degree relatives, and healthy controls: a meta-analysis. PloS one 6:e24129.

Zhang b M, Zhao Z, He L, Wan C (2010) A meta-analysis of oxidative stress markers in schizophrenia. Science China Life sciences 53:112-124.

Zhang R, Yan JD, Valenzuela RK, Lu SM, Du XY, Zhong B, Ren J, Zhao SH, Gao CG, Wang L, Guo TW, Ma J (2012) Further evidence for the association of genetic variants of ZNF804A with schizophrenia and a meta-analysis for genome-wide significance variant rs1344706. Schizophrenia research 141:40-47.

**Alzheimer’s disease**

Antunez C, Boada M, Lopez-Arrieta J, Moreno-Rey C, Hernandez I, Marin J, Gayan J, Gonzalez-Perez A, Real LM, Alegret M, Tarraga L, Ramirez-Lorca R, Ruiz A (2011) Genetic association of complement receptor 1 polymorphism rs3818361 in Alzheimer's disease. Alzheimer's & dementia : the journal of the Alzheimer's Association 7:e124-129.

Belbin O, Carrasquillo MM, Crump M, Culley OJ, Hunter TA, Ma L, Bisceglio G, Zou F, Allen M, Dickson DW, Graff-Radford NR, Petersen RC, Morgan K, Younkin SG (2011) Investigation of 15 of the top candidate genes for late-onset Alzheimer's disease. Human genetics 129:273-282.

Cao L, Wang F, Ge H, Wu PC, Qu P, Chen GH, Gao ZL (2013) PIN1-842G/C and -667T/C polymorphisms are not associated with the susceptibility of Alzheimer's disease: pooled analysis of epidemiologic studies. Neuroscience letters 535:100-103.

Chang WW, Zhang L, Jin YL, Yao YS (2013) Meta-analysis of the transforming growth factor-beta1 polymorphisms and susceptibility to Alzheimer's disease. Journal of neural transmission (Vienna, Austria : 1996) 120:353-360.

Chang-Quan H, Hui W, Chao-Min W, Zheng-Rong W, Jun-Wen G, Yong-Hong L, Yan-You L, Qing-Xiu L (2011) The association of antihypertensive medication use with risk of cognitive decline and dementia: a meta-analysis of longitudinal studies. International journal of clinical practice 65:1295-1305.

Di Bona D, Candore G, Franceschi C, Licastro F, Colonna-Romano G, Camma C, Lio D, Caruso C (2009) Systematic review by meta-analyses on the possible role of TNF-alpha polymorphisms in association with Alzheimer's disease. Brain research reviews 61:60-68.

Di Bona D, Rizzo C, Bonaventura G, Candore G, Caruso C (2012) Association between interleukin-10 polymorphisms and Alzheimer's disease: a systematic review and meta-analysis. Journal of Alzheimer's disease : JAD 29:751-759.

Farrall AJ, Wardlaw JM (2009) Blood-brain barrier: ageing and microvascular disease--systematic review and meta-analysis. Neurobiology of aging 30:337-352.

Fukumoto N, Fujii T, Combarros O, Kamboh MI, Tsai SJ, Matsushita S, Nacmias B, Comings DE, Arboleda H, Ingelsson M, Hyman BT, Akatsu H, Grupe A, Nishimura AL, Zatz M, Mattila KM, Rinne J, Goto Y, Asada T, Nakamura S, Kunugi H (2010) Sexually dimorphic effect of the Val66Met polymorphism of BDNF on susceptibility to Alzheimer's disease: New data and meta-analysis. American journal of medical genetics Part B, Neuropsychiatric genetics : the official publication of the International Society of Psychiatric Genetics 153B:235-242.

Guan F, Gu J, Hu F, Zhu Y, Wang W (2012) Association between alpha1-antichymotrypsin signal peptide -15A/T polymorphism and the risk of Alzheimer's disease: a meta-analysis. Molecular biology reports 39:6661-6669.

Ho RC, Cheung MW, Fu E, Win HH, Zaw MH, Ng A, Mak A (2011) Is high homocysteine level a risk factor for cognitive decline in elderly? A systematic review, meta-analysis, and meta-regression. The American journal of geriatric psychiatry : official journal of the American Association for Geriatric Psychiatry 19:607-617.

Hua b Y, Zhao H, Lu X, Kong Y, Jin H (2012) Meta-analysis of the cystatin C(CST3) gene G73A polymorphism and susceptibility to Alzheimer's disease. The International journal of neuroscience 122:431-438.

Hua Y, Zhao H, Kong Y, Ye M (2011) Association between the MTHFR gene and Alzheimer's disease: a meta-analysis. The International journal of neuroscience 121:462-471.

Jiang M, Lv L, Wang H, Yang X, Ji H, Zhou F, Zhu W, Cai L, Gu X, Sun J, Dong Q (2012) Meta-analysis on association between the ATP-binding cassette transporter A1 gene (ABCA1) and Alzheimer's disease. Gene 510:147-153.

Koyama A, O'Brien J, Weuve J, Blacker D, Metti AL, Yaffe K (2013) The role of peripheral inflammatory markers in dementia and Alzheimer's disease: a meta-analysis. The journals of gerontology Series A, Biological sciences and medical sciences 68:433-440.

Li b L, Yin Z, Liu J, Li G, Wang Y, Yan J, Zhou H (2013) CYP46A1 T/C polymorphism associated with the APOE epsilon4 allele increases the risk of Alzheimer's disease. Journal of neurology 260:1701-1708.

Li BH, Zhang LL, Yin YW, Pi Y, Guo L, Yang QW, Gao CY, Fang CQ, Wang JZ, Xiang J, Li JC (2013) Association between interleukin-1alpha C(-889)T polymorphism and Alzheimer's disease: a meta-analysis including 12,817 subjects. Journal of neural transmission (Vienna, Austria : 1996) 120:497-506.

Lin M, Zhao L, Fan J, Lian XG, Ye JX, Wu L, Lin H (2012) Association between HFE polymorphisms and susceptibility to Alzheimer's disease: a meta-analysis of 22 studies including 4,365 cases and 8,652 controls. Molecular biology reports 39:3089-3095.

Llorca J, Rodriguez-Rodriguez E, Dierssen-Sotos T, Delgado-Rodriguez M, Berciano J, Combarros O (2008) Meta-analysis of genetic variability in the beta-amyloid production, aggregation and degradation metabolic pathways and the risk of Alzheimer's disease. Acta neurologica Scandinavica 117:1-14.

Millet X, Le Goff M, Bouisson J, Dartigues JF, Amieva H (2010) Encoding processes influence word-stem completion priming in Alzheimer's disease: a meta-analysis. Journal of clinical and experimental neuropsychology 32:494-504.

Pi Y, Zhang L, Chang K, Li B, Guo L, Fang C, Gao C, Wang J, Xiang J, Li J (2012) Lack of an association between Paraoxonase 1 gene polymorphisms (Q192R, L55M) and Alzheimer's disease: a meta-analysis. Neuroscience letters 523:174-179.

Power MC, Weuve J, Gagne JJ, McQueen MB, Viswanathan A, Blacker D (2011) The association between blood pressure and incident Alzheimer disease: a systematic review and meta-analysis. Epidemiology (Cambridge, Mass) 22:646-659.

Qi HP, Qu ZY, Duan SR, Wei SQ, Wen SR, Bi S (2012) IL-6-174 G/C and -572 C/G polymorphisms and risk of Alzheimer's disease. PloS one 7:e37858.

Rahayel S, Frasnelli J, Joubert S (2012) The effect of Alzheimer's disease and Parkinson's disease on olfaction: a meta-analysis. Behavioural brain research 231:60-74.

Reynolds CA, Hong MG, Eriksson UK, Blennow K, Johansson B, Malmberg B, Berg S, Gatz M, Pedersen NL, Bennet AM, Prince JA (2010) Sequence variation in SORL1 and dementia risk in Swedes. Neurogenetics 11:139-142.

Sabayan B, Jansen S, Oleksik AM, van Osch MJ, van Buchem MA, van Vliet P, de Craen AJ, Westendorp RG (2012) Cerebrovascular hemodynamics in Alzheimer's disease and vascular dementia: a meta-analysis of transcranial Doppler studies. Ageing research reviews 11:271-277.

Sadigh-Eteghad S, Talebi M, Farhoudi M (2012) Association of apolipoprotein E epsilon 4 allele with sporadic late onset Alzheimer`s disease. A meta-analysis. Neurosciences (Riyadh, Saudi Arabia) 17:321-326.

Schrag M, Mueller C, Oyoyo U, Smith MA, Kirsch WM (2011) Iron, zinc and copper in the Alzheimer's disease brain: a quantitative meta-analysis. Some insight on the influence of citation bias on scientific opinion. Progress in neurobiology 94:296-306.

Schuur M, Ikram MA, van Swieten JC, Isaacs A, Vergeer-Drop JM, Hofman A, Oostra BA, Breteler MM, van Duijn CM (2011) Cathepsin D gene and the risk of Alzheimer's disease: a population-based study and meta-analysis. Neurobiology of aging 32:1607-1614.

Seitz DP, Shah PS, Herrmann N, Beyene J, Siddiqui N (2011) Exposure to general anesthesia and risk of Alzheimer's disease: a systematic review and meta-analysis. BMC geriatrics 11:83.

Song F, Poljak A, Valenzuela M, Mayeux R, Smythe GA, Sachdev PS (2011) Meta-analysis of plasma amyloid-beta levels in Alzheimer's disease. Journal of Alzheimer's disease : JAD 26:365-375.

Ventriglia M, Bucossi S, Panetta V, Squitti R (2012) Copper in Alzheimer's disease: a meta-analysis of serum, plasma, and cerebrospinal fluid studies. Journal of Alzheimer's disease : JAD 30:981-984.

Wang b Y, Bi L, Wang H, Li Y, Di Q, Xu W, Qian Y (2012) NEDD9 rs760678 polymorphism and the risk of Alzheimer's disease: a meta-analysis. Neuroscience letters 527:121-125.

Xin XY, Ding JQ, Chen SD (2010) Apolipoprotein E promoter polymorphisms and risk of Alzheimer's disease: evidence from meta-analysis. Journal of Alzheimer's disease : JAD 19:1283-1294.

Yuan H, Xia Q, Ge P, Wu S (2013) Genetic polymorphism of interleukin 1beta -511C/T and susceptibility to sporadic Alzheimer's disease: a meta-analysis. Molecular biology reports 40:1827-1834.

**Epilepsy**

Otte WM, van Eijsden P, Sander JW, Duncan JS, Dijkhuizen RM, Braun KP (2012) A meta-analysis of white matter changes in temporal lobe epilepsy as studied with diffusion tensor imaging. Epilepsia 53:659-667.

Quattrocchi G, Nicoletti A, Marin B, Bruno E, Druet-Cabanac M, Preux PM (2012) Toxocariasis and epilepsy: systematic review and meta-analysis. PLoS neglected tropical diseases 6:e1775.

Haerian BS, Baum L (2013) GABRG2 rs211037 polymorphism and epilepsy: a systematic review and meta-analysis. Seizure : the journal of the British Epilepsy Association 22:53-58.

Kauffman MA, Consalvo D, Moron DG, Lereis VP, Kochen S (2010) ApoE epsilon4 genotype and the age at onset of temporal lobe epilepsy: a case-control study and meta-analysis. Epilepsy research 90:234-239.

Yu N, Di Q, Hu Y, Zhang YF, Su LY, Liu XH, Li LC (2012) A meta-analysis of pro-inflammatory cytokines in the plasma of epileptic patients with recent seizure. Neuroscience letters 514:110-115.

Nurmohamed L, Garcia-Bournissen F, Buono RJ, Shannon MW, Finkelstein Y (2010) Predisposition to epilepsy--does the ABCB1 gene play a role? Epilepsia 51:1882-1885.

Kauffman b MA, Moron DG, Consalvo D, Bello R, Kochen S (2008) Association study between interleukin 1 beta gene and epileptic disorders: a HuGe review and meta-analysis. Genetics in medicine : official journal of the American College of Medical Genetics 10:83-88.

Brigo F, Storti M, Benedetti MD, Rossini F, Nardone R, Tezzon F, Fiaschi A, Bongiovanni LG, Manganotti P (2012) Resting motor threshold in idiopathic generalized epilepsies: a systematic review with meta-analysis. Epilepsy research 101:3-13.

Quet F, Guerchet M, Pion SD, Ngoungou EB, Nicoletti A, Preux PM (2010) Meta-analysis of the association between cysticercosis and epilepsy in Africa. Epilepsia 51:830-837.

Samokhvalov AV, Irving H, Mohapatra S, Rehm J (2010) Alcohol consumption, unprovoked seizures, and epilepsy: a systematic review and meta-analysis. Epilepsia 51:1177-1184.

Amiet C, Gourfinkel-An I, Bouzamondo A, Tordjman S, Baulac M, Lechat P, Mottron L, Cohen D (2008) Epilepsy in autism is associated with intellectual disability and gender: evidence from a meta-analysis. Biological psychiatry 64:577-582.

Lotufo PA, Valiengo L, Bensenor IM, Brunoni AR (2012) A systematic review and meta-analysis of heart rate variability in epilepsy and antiepileptic drugs. Epilepsia 53:272-282.

**Multiple sclerosis**

Barragan-Martinez C, Speck-Hernandez CA, Montoya-Ortiz G, Mantilla RD, Anaya JM, Rojas-Villarraga A (2012) Organic solvents as risk factor for autoimmune diseases: a systematic review and meta-analysis. PloS one 7:e51506.

Farez MF, Correale J (2011) Immunizations and risk of multiple sclerosis: systematic review and meta-analysis. Journal of neurology 258:1197-1206.

Handel AE, Williamson AJ, Disanto G, Dobson R, Giovannoni G, Ramagopalan SV (2011) Smoking and multiple sclerosis: an updated meta-analysis. PloS one 6:e16149.

Handel AE, Williamson AJ, Disanto G, Handunnetthi L, Giovannoni G, Ramagopalan SV (2010) An updated meta-analysis of risk of multiple sclerosis following infectious mononucleosis. PloS one 5.

Huang J, Xie ZF (2012) Polymorphisms in the vitamin D receptor gene and multiple sclerosis risk: a meta-analysis of case-control studies. Journal of the neurological sciences 313:79-85.

Huang J, Xie ZK, Lu RB, Xie ZF (2013) Association of interleukin-1 gene polymorphisms with multiple sclerosis: a meta-analysis. Inflammation research : official journal of the European Histamine Research Society [et al] 62:97-106.

Kudryavtseva EA, Rozhdestvenskii AS, Kakulya AV, Khanokh EV, Delov RA, Malkova NA, Korobko DS, Platonov FA, Aref Eva EG, Zagorskaya NN, Aliferova VM, Titova MA, Babenko SA, Smagina IV, El Chaninova SA, Zolovkina AG, Lifshits GI, Puzyrev VP, Filipenko ML (2011) Polymorphic locus rs10492972 of the KIF1B gene association with multiple sclerosis in Russia: case control study. Molecular genetics and metabolism 104:390-394.

Monteiro L, Souza-Machado A, Menezes C, Melo A (2011) Association between allergies and multiple sclerosis: a systematic review and meta-analysis. Acta neurologica Scandinavica 123:1-7.

Nikolopoulos GK, Masgala A, Tsiara C, Limitsiou OK, Karnaouri AC, Dimou NL, Bagos PG (2011) Cytokine gene polymorphisms in multiple sclerosis: a meta-analysis of 45 studies including 7379 cases and 8131 controls. European journal of neurology : the official journal of the European Federation of Neurological Societies 18:944-951.

O'Gorman C, Lin R, Stankovich J, Broadley SA (2013) Modelling genetic susceptibility to multiple sclerosis with family data. Neuroepidemiology 40:1-12.

Pakpoor J, Handel AE, Giovannoni G, Dobson R, Ramagopalan SV (2012) Meta-analysis of the relationship between multiple sclerosis and migraine. PloS one 7:e45295.

Qiu W, James I, Carroll WM, Mastaglia FL, Kermode AG (2011) HLA-DR allele polymorphism and multiple sclerosis in Chinese populations: a meta-analysis. Multiple sclerosis (Houndmills, Basingstoke, England) 17:382-388.

Santiago O, Gutierrez J, Sorlozano A, de Dios Luna J, Villegas E, Fernandez O (2010) Relation between Epstein-Barr virus and multiple sclerosis: analytic study of scientific production. European journal of clinical microbiology & infectious diseases : official publication of the European Society of Clinical Microbiology 29:857-866.

Wang LM, Zhang DM, Xu YM, Sun SL (2011) Interleukin 2 receptor alpha gene polymorphism and risk of multiple sclerosis: a meta-analysis. The Journal of international medical research 39:1625-1635.

Yin YW, Zhang YD, Wang JZ, Li BH, Yang QW, Fang CQ, Gao CY, Li JC, Zhang LL (2012) Association between apolipoprotein E gene polymorphism and the risk of multiple sclerosis: a meta-analysis of 6977 subjects. Gene 511:12-17.

Zhang b Q, Lin CY, Dong Q, Wang J, Wang W (2011) Relationship between HLA-DRB1 polymorphism and susceptibility or resistance to multiple sclerosis in Caucasians: a meta-analysis of non-family-based studies. Autoimmunity reviews 10:474-481.

Zhang R, Duan L, Jiang Y, Zhang X, Sun P, Li J, Zhang M, Tang G, Wang X, Li X (2011) Association between the IL7R T244I polymorphism and multiple sclerosis: a meta-analysis. Molecular biology reports 38:5079-5084.

Zhu Y, He ZY, Liu HN (2011) Meta-analysis of the relationship between homocysteine, vitamin B(1)(2), folate, and multiple sclerosis. Journal of clinical neuroscience : official journal of the Neurosurgical Society of Australasia 18:933-938.

**Parkinson’s Disease**

Agundez JA, Lorenzo-Betancor O, Pastor P, Garcia-Martin E, Luengo A, Alonso-Navarro H, Jimenez-Jimenez FJ (2012) LINGO1 rs9652490 and rs11856808 are not associated with the risk of Parkinson's disease: results of a meta-analysis. Parkinsonism & related disorders 18:657-659.

Balafkan N, Tzoulis C, Muller B, Haugarvoll K, Tysnes OB, Larsen JP, Bindoff LA (2012) Number of CAG repeats in POLG1 and its association with Parkinson disease in the Norwegian population. Mitochondrion 12:640-643.

Chu K, Zhou X, Luo BY (2012) Cytokine gene polymorphisms and Parkinson's disease: a meta-analysis. The Canadian journal of neurological sciences Le journal canadien des sciences neurologiques 39:58-64.

Gray HM, Tickle-Degnen L (2010) A meta-analysis of performance on emotion recognition tasks in Parkinson's disease. Neuropsychology 24:176-191.

Li FJ, Ji HF, Shen L (2012) A meta-analysis of tea drinking and risk of Parkinson's disease. TheScientificWorldJournal 2012:923464.

Lill CM, Roehr JT, McQueen MB, Kavvoura FK, Bagade S, Schjeide BM, Schjeide LM, Meissner E, Zauft U, Allen NC, Liu T, Schilling M, Anderson KJ, Beecham G, Berg D, Biernacka JM, Brice A, DeStefano AL, Do CB, Eriksson N, Factor SA, Farrer MJ, Foroud T, Gasser T, Hamza T, Hardy JA, Heutink P, Hill-Burns EM, Klein C, Latourelle JC, Maraganore DM, Martin ER, Martinez M, Myers RH, Nalls MA, Pankratz N, Payami H, Satake W, Scott WK, Sharma M, Singleton AB, Stefansson K, Toda T, Tung JY, Vance J, Wood NW, Zabetian CP, Young P, Tanzi RE, Khoury MJ, Zipp F, Lehrach H, Ioannidis JP, Bertram L (2012) Comprehensive research synopsis and systematic meta-analyses in Parkinson's disease genetics: The PDGene database. PLoS genetics 8:e1002548.

Liu b YL, Yang J, Zheng J, Liu DW, Liu T, Wang JM, Wang CN, Wang MW, Tian QB (2012) Paraoxonase 1 polymorphisms L55M and Q192R were not risk factors for Parkinson's disease: a HuGE review and meta-analysis. Gene 501:188-192.

Mariani S, Ventriglia M, Simonelli I, Donno S, Bucossi S, Vernieri F, Melgari JM, Pasqualetti P, Rossini PM, Squitti R (2013) Fe and Cu do not differ in Parkinson's disease: a replication study plus meta-analysis. Neurobiology of aging 34:632-633.

Noyce AJ, Bestwick JP, Silveira-Moriyama L, Hawkes CH, Giovannoni G, Lees AJ, Schrag A (2012) Meta-analysis of early nonmotor features and risk factors for Parkinson disease. Annals of neurology 72:893-901.

Ragland M, Hutter C, Zabetian C, Edwards K (2009) Association between the ubiquitin carboxyl-terminal esterase L1 gene (UCHL1) S18Y variant and Parkinson's Disease: a HuGE review and meta-analysis. American journal of epidemiology 170:1344-1357.

Rahayel S, Frasnelli J, Joubert S (2012) The effect of Alzheimer's disease and Parkinson's disease on olfaction: a meta-analysis. Behavioural brain research 231:60-74.

Tang L, Zhao S, Wang M, Sheth A, Zhao Z, Chen L, Fan X, Chen L (2012) Meta-analysis of association between PITX3 gene polymorphism and Parkinson's disease. Journal of the neurological sciences 317:80-86.

Undela K, Gudala K, Malla S, Bansal D (2013) Statin use and risk of Parkinson's disease: a meta-analysis of observational studies. Journal of neurology 260:158-165.

van der Hoorn A, Burger H, Leenders KL, de Jong BM (2012) Handedness correlates with the dominant Parkinson side: a systematic review and meta-analysis. Movement disorders : official journal of the Movement Disorder Society 27:206-210.

van der Mark M, Brouwer M, Kromhout H, Nijssen P, Huss A, Vermeulen R (2012) Is pesticide use related to Parkinson disease? Some clues to heterogeneity in study results. Environmental health perspectives 120:340-347.

Wang Y, Yang X (2012) Association of catechol-o-methyltransferase polymorphism (Val108/158Met) with Parkinson's disease: a meta-analysis. Journal of motor behavior 44:365-372.

Williams-Gray CH, Goris A, Saiki M, Foltynie T, Compston DA, Sawcer SJ, Barker RA (2009) Apolipoprotein E genotype as a risk factor for susceptibility to and dementia in Parkinson's disease. Journal of neurology 256:493-498.

Wu X, Tang KF, Li Y, Xiong YY, Shen L, Wei ZY, Zhou KJ, Niu JM, Han X, Yang L, Feng GY, He L, Qin SY (2012) Quantitative assessment of the effect of LRRK2 exonic variants on the risk of Parkinson's disease: a meta-analysis. Parkinsonism & related disorders 18:722-730.

Zhang b Y, Wang ZZ, Sun HM (2012) Lack of association between p.Ser167Asn variant of Parkin and Parkinson's disease: a meta-analysis of 15 studies involving 2,280 cases and 2,459 controls. American journal of medical genetics Part B, Neuropsychiatric genetics : the official publication of the International Society of Psychiatric Genetics 159B:38-47.

Zhang Y, Wang ZZ, Sun HM (2012) Meta-analysis of the influence of Parkin p.Asp394Asn variant on the susceptibility of Parkinson's disease. Neuroscience letters 524:60-64.

**Breast cancer**

Ambrosone CB, Kropp S, Yang J, Yao S, Shields PG, Chang-Claude J (2008) Cigarette smoking, N-acetyltransferase 2 genotypes, and breast cancer risk: pooled analysis and meta-analysis. Cancer epidemiology, biomarkers & prevention : a publication of the American Association for Cancer Research, cosponsored by the American Society of Preventive Oncology 17:15-26.

Angelousi AG, Anagnostou VK, Stamatakos MK, Georgiopoulos GA, Kontzoglou KC (2012) Mechanisms in endocrinology: primary HT and risk for breast cancer: a systematic review and meta-analysis. European journal of endocrinology / European Federation of Endocrine Societies 166:373-381.

Aune b D, Chan DS, Vieira AR, Navarro Rosenblatt DA, Vieira R, Greenwood DC, Norat T (2012) Dietary compared with blood concentrations of carotenoids and breast cancer risk: a systematic review and meta-analysis of prospective studies. The American journal of clinical nutrition 96:356-373.

Aune c D, Chan DS, Vieira AR, Rosenblatt DA, Vieira R, Greenwood DC, Norat T (2012) Fruits, vegetables and breast cancer risk: a systematic review and meta-analysis of prospective studies. Breast cancer research and treatment 134:479-493.

Aune D, Chan DS, Greenwood DC, Vieira AR, Rosenblatt DA, Vieira R, Norat T (2012) Dietary fiber and breast cancer risk: a systematic review and meta-analysis of prospective studies. Annals of oncology : official journal of the European Society for Medical Oncology / ESMO 23:1394-1402.

Bosetti C, Rosato V, Gallus S, Cuzick J, La Vecchia C (2012) Aspirin and cancer risk: a quantitative review to 2011. Annals of oncology : official journal of the European Society for Medical Oncology / ESMO 23:1403-1415.

Boyle P, Koechlin A, Pizot C, Boniol M, Robertson C, Mullie P, Bolli G, Rosenstock J, Autier P (2013) Blood glucose concentrations and breast cancer risk in women without diabetes: a meta-analysis. European journal of nutrition 52:1533-1540.

Buck K, Zaineddin AK, Vrieling A, Linseisen J, Chang-Claude J (2010) Meta-analyses of lignans and enterolignans in relation to breast cancer risk. The American journal of clinical nutrition 92:141-153.

Chen b P, Hu P, Xie D, Qin Y, Wang F, Wang H (2010) Meta-analysis of vitamin D, calcium and the prevention of breast cancer. Breast cancer research and treatment 121:469-477.

Chen b W, Zhong R, Ming J, Zou L, Zhu B, Lu X, Ke J, Zhang Y, Liu L, Miao X, Huang T (2012) The SLC4A7 variant rs4973768 is associated with breast cancer risk: evidence from a case-control study and a meta-analysis. Breast cancer research and treatment 136:847-857.

Chen C, Ma X, Zhong M, Yu Z (2010) Extremely low-frequency electromagnetic fields exposure and female breast cancer risk: a meta-analysis based on 24,338 cases and 60,628 controls. Breast cancer research and treatment 123:569-576.

Chen c L, Zhou WB, Zhao Y, Liu XA, Ding Q, Zha XM, Wang S (2012) Bloody nipple discharge is a predictor of breast cancer risk: a meta-analysis. Breast cancer research and treatment 132:9-14.

Chen c MB, Li C, Shen WX, Guo YJ, Shen W, Lu PH (2011) Association of a LSP1 gene rs3817198T>C polymorphism with breast cancer risk: evidence from 33,920 cases and 35,671 controls. Molecular biology reports 38:4687-4695.

Chen d Y, Pei J (2010) Factors influencing the association between CYP17 T34C polymorphism and the risk of breast cancer: meta-regression and subgroup analysis. Breast cancer research and treatment 122:471-481.

Chen X, Guan J, Song Y, Chen P, Zheng H, Tang C, Wu Q (2008) IGF-I (CA) repeat polymorphisms and risk of cancer: a meta-analysis. Journal of human genetics 53:227-238.

Chen Y, Pei J (2011) Possible risk modifications in the association between MnSOD Ala-9Val polymorphism and breast cancer risk: subgroup analysis and evidence-based sample size calculation for a future trial. Breast cancer research and treatment 125:495-504.

Choi Y, Giovannucci E, Lee JE (2012) Glycaemic index and glycaemic load in relation to risk of diabetes-related cancers: a meta-analysis. The British journal of nutrition 108:1934-1947.

Colleran G, McInerney N, Rowan A, Barclay E, Jones AM, Curran C, Miller N, Kerin M, Tomlinson I, Sawyer E (2010) The TGFBR1*6A/9A polymorphism is not associated with differential risk of breast cancer. Breast cancer research and treatment 119:437-442.

Dahabreh IJ, Murray S (2011) Lack of replication for the association between HER2 I655V polymorphism and breast cancer risk: a systematic review and meta-analysis. Cancer epidemiology 35:503-509.

Dong c JY, Qin LQ (2011) Soy isoflavones consumption and risk of breast cancer incidence or recurrence: a meta-analysis of prospective studies. Breast cancer research and treatment 125:315-323.

Dong d JY, Zhang L, He K, Qin LQ (2011) Dairy consumption and risk of breast cancer: a meta-analysis of prospective cohort studies. Breast cancer research and treatment 127:23-31.

Eom CS, Park SM, Cho KH (2012) Use of antidepressants and the risk of breast cancer: a meta-analysis. Breast cancer research and treatment 136:635-645.

Esposito K, Chiodini P, Colao A, Lenzi A, Giugliano D (2012) Metabolic syndrome and risk of cancer: a systematic review and meta-analysis. Diabetes care 35:2402-2411.

Francisco G, Menezes PR, Eluf-Neto J, Chammas R (2008) XPC polymorphisms play a role in tissue-specific carcinogenesis: a meta-analysis. European journal of human genetics : EJHG 16:724-734.

Fulan H, Changxing J, Baina WY, Wencui Z, Chunqing L, Fan W, Dandan L, Dianjun S, Tong W, Da P, Yashuang Z (2011) Retinol, vitamins A, C, and E and breast cancer risk: a meta-analysis and meta-regression. Cancer causes & control : CCC 22:1383-1396.

Gao b LB, Pan XM, Li LJ, Liang WB, Zhu Y, Zhang LS, Wei YG, Tang M, Zhang L (2011) RAD51 135G/C polymorphism and breast cancer risk: a meta-analysis from 21 studies. Breast cancer research and treatment 125:827-835.

Guo H, Ming J, Liu C, Li Z, Zhang N, Cheng H, Wang W, Shi W, Shen N, Zhao Q, Li D, Yi P, Wang L, Wang R, Xin Y, Zhao X, Nie X, Huang T (2012) A common polymorphism near the ESR1 gene is associated with risk of breast cancer: evidence from a case-control study and a meta-analysis. PloS one 7:e52445.

Hao b Y, Montiel R, Li B, Huang E, Zeng L, Huang Y (2010) Association between androgen receptor gene CAG repeat polymorphism and breast cancer risk: a meta-analysis. Breast cancer research and treatment 124:815-820.

Hao Y, Montiel R, Huang Y (2010) Endothelial nitric oxide synthase (eNOS) 894 G>T polymorphism is associated with breast cancer risk: a meta-analysis. Breast cancer research and treatment 124:809-813.

Hardefeldt c PJ, Eslick GD, Edirimanne S (2012) Benign thyroid disease is associated with breast cancer: a meta-analysis. Breast cancer research and treatment 133:1169-1177.

He c XF, Wei W, Su J, Yang ZX, Liu Y, Zhang Y, Ding DP, Wang W (2012) Association between the XRCC3 polymorphisms and breast cancer risk: meta-analysis based on case-control studies. Molecular biology reports 39:5125-5134.

Hu c Z, Li X, Qu X, He Y, Ring BZ, Song E, Su L (2010) Intron 3 16 bp duplication polymorphism of TP53 contributes to cancer susceptibility: a meta-analysis. Carcinogenesis 31:643-647.

Hu d Z, Li X, Yuan R, Ring BZ, Su L (2010) Three common TP53 polymorphisms in susceptibility to breast cancer, evidence from meta-analysis. Breast cancer research and treatment 120:705-714.

Huang Y, Li B, Qian J, Xie J, Yu L (2010) TGF-beta1 29T/C polymorphism and breast cancer risk: a meta-analysis involving 25,996 subjects. Breast cancer research and treatment 123:863-868.

Huo Q, Zhang N, Yang Q (2012) Epstein-Barr virus infection and sporadic breast cancer risk: a meta-analysis. PloS one 7:e31656.

Jiang b Z, Li C, Xu Y, Cai S, Wang X (2010) Associations between XPD polymorphisms and risk of breast cancer: a meta-analysis. Breast cancer research and treatment 123:203-212.

Kim JS, Kang EJ, Woo OH, Park KH, Woo SU, Yang DS, Kim AR, Lee JB, Kim YH, Kim JS, Seo JH (2013) The relationship between preeclampsia, pregnancy-induced hypertension and maternal risk of breast cancer: a meta-analysis. Acta oncologica (Stockholm, Sweden) 52:1643-1648.

Lee H, Wang Q, Yang F, Tao P, Li H, Huang Y, Li JY (2012) SULT1A1 Arg213His polymorphism, smoked meat, and breast cancer risk: a case-control study and meta-analysis. DNA and cell biology 31:688-699.

Li b L, Huang X, Huo K (2010) IGFBP3 polymorphisms and risk of cancer: a meta-analysis. Molecular biology reports 37:127-140.

Li b N, Bi X, Zhang Y, Zhao P, Zheng T, Dai M (2011) Human papillomavirus infection and sporadic breast carcinoma risk: a meta-analysis. Breast cancer research and treatment 126:515-520.

Li c N, Dong J, Hu Z, Shen H, Dai M (2010) Potentially functional polymorphisms in ESR1 and breast cancer risk: a meta-analysis. Breast cancer research and treatment 121:177-184.

Liu b X, Lv K (2013) Cruciferous vegetables intake is inversely associated with risk of breast cancer: a meta-analysis. Breast (Edinburgh, Scotland) 22:309-313.

Liu C, Liu L (2011) Polymorphisms in three obesity-related genes (LEP, LEPR, and PON1) and breast cancer risk: a meta-analysis. Tumour biology : the journal of the International Society for Oncodevelopmental Biology and Medicine 32:1233-1240.

Liu C, Wang Y, Wang QS, Wang YJ (2012) The CHEK2 I157T variant and breast cancer susceptibility: a systematic review and meta-analysis. Asian Pacific journal of cancer prevention : APJCP 13:1355-1360.

Lu b PH, Yang J, Li C, Wei MX, Shen W, Shi LP, Jiang ZY, Zhou N, Tao GQ (2011) Association between mitogen-activated protein kinase kinase kinase 1 rs889312 polymorphism and breast cancer risk: evidence from 59,977 subjects. Breast cancer research and treatment 126:663-670.

Lu C, Dong J, Ma H, Jin G, Hu Z, Peng Y, Guo X, Wang X, Shen H (2009) CCND1 G870A polymorphism contributes to breast cancer susceptibility: a meta-analysis. Breast cancer research and treatment 116:571-575.

Lu c S, Wang Z, Cui D, Liu H, Hao X (2011) Glutathione S-transferase P1 Ile105Val polymorphism and breast cancer risk: a meta-analysis involving 34,658 subjects. Breast cancer research and treatment 125:253-259.

Mao C, Chung VC, He BF, Luo RC, Tang JL (2012) Association between ATM 5557G>A polymorphism and breast cancer risk: a meta-analysis. Molecular biology reports 39:1113-1118.

Ni XJ, Xia TS, Zhao YC, Ma JJ, Zhao J, Liu XA, Ding Q, Zha XM, Wang S (2012) Postmenopausal hormone therapy is associated with in situ breast cancer risk. Asian Pacific journal of cancer prevention : APJCP 13:3917-3925.

Niu W, Qi Y, Gao P, Zhu D (2010) Association of TGFB1 -509 C>T polymorphism with breast cancer: evidence from a meta-analysis involving 23,579 subjects. Breast cancer research and treatment 124:243-249.

Pei XH, Li HX (2012) Insertion/deletion (I/D) in the angiotensin-converting enzyme gene and breast cancer risk: lack of association in a meta- analysis. Asian Pacific journal of cancer prevention : APJCP 13:5633-5636.

Pei YL, Zhang HL, Han HG (2013) Polymorphism of 8q24 rsl3281615 and breast cancer risk : a meta-analysis. Tumour biology : the journal of the International Society for Oncodevelopmental Biology and Medicine 34:421-428.

Qin X, Peng Q, Qin A, Chen Z, Lin L, Deng Y, Xie L, Xu J, Li H, Li T, Li S, Zhao J (2012) Association of COMT Val158Met polymorphism and breast cancer risk: an updated meta-analysis. Diagnostic pathology 7:136.

Qiu c LX, Zhang J, Li WH, Zhang QL, Yu H, Wang BY, Wang LP, Wang JL, Wang HJ, Liu XJ, Luo ZG, Wu XH (2011) Lack of association between methylenetetrahydrofolate reductase gene A1298C polymorphism and breast cancer susceptibility. Molecular biology reports 38:2295-2299.

Qiu g LX, Yao L, Zhang J, Zhu XD, Zhao XM, Xue K, Mao C, Chen B, Zhan P, Yuan H, Hu XC (2010) XPD Lys751Gln polymorphism and breast cancer susceptibility: a meta-analysis involving 28,709 subjects. Breast cancer research and treatment 124:229-235.

Raimondi S, Johansson H, Maisonneuve P, Gandini S (2009) Review and meta-analysis on vitamin D receptor polymorphisms and cancer risk. Carcinogenesis 30:1170-1180.

Renehan AG, Tyson M, Egger M, Heller RF, Zwahlen M (2008) Body-mass index and incidence of cancer: a systematic review and meta-analysis of prospective observational studies. Lancet 371:569-578.

Shen C, Sun H, Sun D, Xu L, Zhang X, Liu A, Jia X, Bai J, Chen F, Yu Y, Jin Y, Yu J, Fu S (2011) Polymorphisms of tumor necrosis factor-alpha and breast cancer risk: a meta-analysis. Breast cancer research and treatment 126:763-770.

Sheng X, Zhang L, Tong N, Luo D, Wang M, Xu M, Zhang Z (2012) MDR1 C3435T polymorphism and cancer risk: a meta-analysis based on 39 case-control studies. Molecular biology reports 39:7237-7249.

Smitten AL, Simon TA, Hochberg MC, Suissa S (2008) A meta-analysis of the incidence of malignancy in adult patients with rheumatoid arthritis. Arthritis research & therapy 10:R45.

Suzuki R, Orsini N, Mignone L, Saji S, Wolk A (2008) Alcohol intake and risk of breast cancer defined by estrogen and progesterone receptor status--a meta-analysis of epidemiological studies. International journal of cancer Journal international du cancer 122:1832-1841.

Suzuki R, Orsini N, Saji S, Key TJ, Wolk A (2009) Body weight and incidence of breast cancer defined by estrogen and progesterone receptor status--a meta-analysis. International journal of cancer Journal international du cancer 124:698-712.

Takkouche B, Regueira-Mendez C, Etminan M (2008) Breast cancer and use of nonsteroidal anti-inflammatory drugs: a meta-analysis. Journal of the National Cancer Institute 100:1439-1447.

Tang b N, Zhou B, Wang B, Yu R (2009) Coffee consumption and risk of breast cancer: a metaanalysis. American journal of obstetrics and gynecology 200:290 e291-299.

Taylor VH, Misra M, Mukherjee SD (2009) Is red meat intake a risk factor for breast cancer among premenopausal women? Breast cancer research and treatment 117:1-8.

Undela K, Srikanth V, Bansal D (2012) Statin use and risk of breast cancer: a meta-analysis of observational studies. Breast cancer research and treatment 135:261-269.

Wang c K, Liu L, Zhu ZM, Shao JH, Xin L (2011) Five polymorphisms of vascular endothelial growth factor (VEGF) and risk of breast cancer: a meta-analysis involving 16,703 individuals. Cytokine 56:167-173.

Wang H, Yang Z, Zhang H (2013) Assessing interactions between the associations of fibroblast growth factor receptor 2 common genetic variants and hormone receptor status with breast cancer risk. Breast cancer research and treatment 137:511-522.

Wang j L, Tang L, Xie R, Nie W, Chen L, Guan X (2012) p16 promoter hypermethylation is associated with increased breast cancer risk. Molecular medicine reports 6:904-908.

Wang k L, Zhang Y, Ding D, He X, Zhu Z (2012) Lack of association of ADH1C genotype with breast cancer susceptibility in Caucasian population: a pooled analysis of case-control studies. Breast (Edinburgh, Scotland) 21:435-439.

Wang Z, Cui D, Lu W (2010) NBS1 8360G > C polymorphism is associated with breast cancer risk: a meta-analysis. Breast cancer research and treatment 123:557-561.

Wei B, Zhou Y, Xu Z, Xi B, Cheng H, Ruan J, Zhu M, Hu Q, Wang Q, Wang Z, Yan Z, Jin K, Zhou D, Xuan F, Huang X, Shao J, Lu P (2011) The effect of hOGG1 Ser326Cys polymorphism on cancer risk: evidence from a meta-analysis. PloS one 6:e27545.

Weiner AS, Boyarskikh UA, Voronina EN, Selezneva IA, Sinkina TV, Lazarev AF, Petrova VD, Filipenko ML (2012) Polymorphisms in the folate-metabolizing genes MTR, MTRR, and CBS and breast cancer risk. Cancer epidemiology 36:e95-e100.

Weischer M, Bojesen SE, Ellervik C, Tybjaerg-Hansen A, Nordestgaard BG (2008) CHEK2*1100delC genotyping for clinical assessment of breast cancer risk: meta-analyses of 26,000 patient cases and 27,000 controls. Journal of clinical oncology : official journal of the American Society of Clinical Oncology 26:542-548.

Wu K, Su D, Lin K, Luo J, Au WW (2011) XRCC1 Arg399Gln gene polymorphism and breast cancer risk: a meta-analysis based on case-control studies. Asian Pacific journal of cancer prevention : APJCP 12:2237-2243.

Wu Y, Zhang D, Kang S (2013) Physical activity and risk of breast cancer: a meta-analysis of prospective studies. Breast cancer research and treatment 137:869-882.

Xu X, Dailey AB, Peoples-Sheps M, Talbott EO, Li N, Roth J (2009) Birth weight as a risk factor for breast cancer: a meta-analysis of 18 epidemiological studies. Journal of women's health (2002) 18:1169-1178.

Yu b KD, Chen AX, Shao ZM (2010) No association between a progesterone receptor gene promoter polymorphism (+331G>A) and breast cancer risk in Caucasian women: evidence from a literature-based meta-analysis. Breast cancer research and treatment 122:853-858.

Yu b L, Chen J (2012) Association of MTHFR Ala222Val (rs1801133) polymorphism and breast cancer susceptibility: An update meta-analysis based on 51 research studies. Diagnostic pathology 7:171.

Yu KD, Chen AX, Qiu LX, Fan L, Yang C, Shao ZM (2010) XRCC2 Arg188His polymorphism is not directly associated with breast cancer risk: evidence from 37,369 subjects. Breast cancer research and treatment 123:219-225.

Yu KD, Di GH, Fan L, Wu J, Hu Z, Shen ZZ, Huang W, Shao ZM (2009) A functional polymorphism in the promoter region of GSTM1 implies a complex role for GSTM1 in breast cancer. FASEB journal : official publication of the Federation of American Societies for Experimental Biology 23:2274-2287.

Yuan W, Xu L, Chen W, Wang L, Fu Z, Pang D, Li D (2011) Evidence on the association between NQO1 Pro187Ser polymorphism and breast cancer risk in the current studies: a meta-analysis. Breast cancer research and treatment 125:467-472.

Zhang b C, Lv GQ, Yu XM, Gu YL, Li JP, Du LF, Zhou P (2011) Current evidence on the relationship between HRAS1 polymorphism and breast cancer risk: a meta-analysis. Breast cancer research and treatment 128:467-472.

Zhang b J, Qiu LX, Wang ZH, Wang JL, He SS, Hu XC (2010) NAT2 polymorphisms combining with smoking associated with breast cancer susceptibility: a meta-analysis. Breast cancer research and treatment 123:877-883.

Zhang d Z, Wang M, Wu D, Wang M, Tong N, Tian Y, Zhang Z (2010) P53 codon 72 polymorphism contributes to breast cancer risk: a meta-analysis based on 39 case-control studies. Breast cancer research and treatment 120:509-517.

Zhang f ZH, Yang LS, Huang F, Hao JH, Su PY, Sun YH (2012) Current evidence on the relationship between two polymorphisms in the NBS1 gene and breast cancer risk: a meta-analysis. Asian Pacific journal of cancer prevention : APJCP 13:5375-5379.

Zhao E, Cui D, Yuan L, Lu W (2012) MDM2 SNP309 polymorphism and breast cancer risk: a meta-analysis. Molecular biology reports 39:3471-3477.

Zhou e P, Huang W, Chu X, Du LF, Li JP, Zhang C (2012) The lymphotoxin-alpha 252A>G polymorphism and breast cancer: a meta-analysis. Asian Pacific journal of cancer prevention : APJCP 13:1949-1952.

Zhu H, Lei X, Feng J, Wang Y (2012) Oral contraceptive use and risk of breast cancer: a meta-analysis of prospective cohort studies. The European journal of contraception & reproductive health care : the official journal of the European Society of Contraception 17:402-414.

**Glaucoma**

Chen H, Chen LJ, Zhang M, Gong W, Tam PO, Lam DS, Pang CP (2010) Ethnicity-based subgroup meta-analysis of the association of LOXL1 polymorphisms with glaucoma. Molecular vision 16:167-177.

Cheng JW, Cheng SW, Ma XY, Cai JP, Li Y, Lu GC, Wei RL (2012) Myocilin polymorphisms and primary open-angle glaucoma: a systematic review and meta-analysis. PloS one 7:e46632.

Cheng JW, Li P, Wei RL (2010) Meta-analysis of association between optineurin gene and primary open-angle glaucoma. Medical science monitor : international medical journal of experimental and clinical research 16:Cr369-377.

Guo b Y, Zhang H, Chen X, Yang X, Cheng W, Zhao K (2012) Association of TP53 polymorphisms with primary open-angle glaucoma: a meta-analysis. Investigative ophthalmology & visual science 53:3756-3763.

Guo Y, Chen X, Zhang H, Li N, Yang X, Cheng W, Zhao K (2012) Association of OPA1 polymorphisms with NTG and HTG: a meta-analysis. PloS one 7:e42387.

Huo Y, Zou H, Lang M, Ji SX, Yin XL, Zheng Z, Liu W, Chen CL, Yuan RD, Ye J (2013) Association between MTHFR C677T polymorphism and primary open-angle glaucoma: a meta-analysis. Gene 512:179-184.

Marcus MW, de Vries MM, Junoy Montolio FG, Jansonius NM (2011) Myopia as a risk factor for open-angle glaucoma: a systematic review and meta-analysis. Ophthalmology 118:1989-1994.e1982.

Xin X, Gao L, Wu T, Sun F (2013) Roles of tumor necrosis factor alpha gene polymorphisms, tumor necrosis factor alpha level in aqueous humor, and the risks of open angle glaucoma: a meta-analysis. Molecular vision 19:526-535.

Xu F, Zhao X, Zeng SM, Li L, Zhong HB, Li M (2012) Homocysteine, B vitamins, methylenetetrahydrofolate reductase gene, and risk of primary open-angle glaucoma: a meta-analysis. Ophthalmology 119:2493-2499.

**Psoriasis**

Chen YF, Chang JS (2012) PTPN22 C1858T and the risk of psoriasis: a meta-analysis. Molecular biology reports 39:7861-7870.

Han Y, Liu T, Lu L (2013) Apolipoprotein E gene polymorphism in psoriasis: a meta-analysis. Archives of medical research 44:46-53.

Liu JL, Zhang SQ, Zeng HM (2013) ApaI, BsmI, FokI and TaqI polymorphisms in the vitamin D receptor (VDR) gene and the risk of psoriasis: a meta-analysis. Journal of the European Academy of Dermatology and Venereology : JEADV 27:739-746.

Wu D, Wu Y, Liu JL, Wang B, Zhang XD (2011) Association between HLA-Cw*0602 polymorphism and psoriasis risk: a meta-analysis. Genetics and molecular research : GMR 10:3109-3120.

Wu J, Ren X, Zhang X, Li C, Li Y, Ma H, Zhou Y, Jin Y, Chen F, Bai J, Fu S (2010) The vascular endothelial growth factor +405 G/C polymorphism in psoriasis. Journal of dermatological science 57:62-63.

Zhu b KJ, Zhu CY, Fan YM (2012) Alcohol consumption and psoriatic risk: a meta-analysis of case-control studies. The Journal of dermatology 39:770-773.

Zhu c KJ, Zhu CY, Shi G, Fan YM (2012) Association of IL23R polymorphisms with psoriasis and psoriatic arthritis: a meta-analysis. Inflammation research : official journal of the European Histamine Research Society [et al] 61:1149-1154.

Zhu KJ, He SM, Sun LD, Hu D, Cheng H, Zhang Z, Li Y, Lv YM, Zhang FY, Yang S, Zhang XJ (2011) Smoking and psoriasis: A meta-analysis of case-control studies. Journal of dermatological science 63:126-128.

**Rheumatoid Arthritis**

Bronson PG, Criswell LA, Barcellos LF (2008) The MHC2TA -168A/G polymorphism and risk for rheumatoid arthritis: a meta-analysis of 6861 patients and 9270 controls reveals no evidence for association. Annals of the rheumatic diseases 67:933-936.

Chang WW, Su H, He L, Zhao KF, Wu JL, Xu ZW (2010) Association between transforming growth factor-beta1 T869C polymorphism and rheumatoid arthritis: a meta-analysis. Rheumatology (Oxford, England) 49:652-656.

Chen b S, Jiang F, Ren J, Liu J, Meng W (2012) Association of IL-18 polymorphisms with rheumatoid arthritis and systemic lupus erythematosus in Asian populations: a meta-analysis. BMC medical genetics 13:107.

Chen J, Huang F, Liu M, Duan X, Xiang Z (2012) Genetic polymorphism of glutathione S-transferase T1 and the risk of rheumatoid arthritis: a meta-analysis. Clinical and experimental rheumatology 30:741-747.

Graf SW, Lester S, Nossent JC, Hill CL, Proudman SM, Lee A, Rischmueller M (2012) Low copy number of the FCGR3B gene and rheumatoid arthritis: a case-control study and meta-analysis. Arthritis research & therapy 14:R28.

Harrison P, Pointon JJ, Chapman K, Roddam A, Wordsworth BP (2008) Interleukin-1 promoter region polymorphism role in rheumatoid arthritis: a meta-analysis of IL-1B-511A/G variant reveals association with rheumatoid arthritis. Rheumatology (Oxford, England) 47:1768-1770.

Hou S, Gao GP, Zhang XJ, Sun L, Peng WJ, Wang HF, Ge XJ, Huang W, Sun YH (2013) PADI4 polymorphisms and susceptibility to rheumatoid arthritis: a meta-analysis. Modern rheumatology / the Japan Rheumatism Association 23:50-60.

Lee c YH, Bae SC, Song GG (2013) Association between the chemokine receptor 5 delta32 polymorphism and rheumatoid arthritis: a meta-analysis. Modern rheumatology / the Japan Rheumatism Association 23:304-310.

Lee d YH, Woo JH, Choi SJ, Ji JD, Song GG (2010) Fc receptor-like 3 -169 C/T polymorphism and RA susceptibility: a meta-analysis. Rheumatology international 30:947-953.

Lee e YH, Bae SC, Choi SJ, Ji JD, Song GG (2012) Associations between TNFAIP3 gene polymorphisms and rheumatoid arthritis: a meta-analysis. Inflammation research : official journal of the European Histamine Research Society [et al] 61:635-641.

Lee g YH, Bae SC, Choi SJ, Ji JD, Song GG (2012) The association between the PTPN22 C1858T polymorphism and rheumatoid arthritis: a meta-analysis update. Molecular biology reports 39:3453-3460.

Lee h YH, Bae SC, Choi SJ, Ji JD, Song GG (2012) Associations between interleukin-10 polymorphisms and susceptibility to rheumatoid arthritis: a meta-analysis. Molecular biology reports 39:81-87.

Lee YH, Bae SC, Choi SJ, Ji JD, Song GG (2013) Associations between interferon regulatory factor 5 polymorphisms and rheumatoid arthritis: a meta-analysis. Molecular biology reports 40:1791-1799.

Li X, Zhang C, Zhang J, Zhang Y, Wu Z, Yang L, Xiang Z, Qi Z, Zhang X, Xiao X (2012) Polymorphisms in the CTLA-4 gene and rheumatoid arthritis susceptibility: a meta-analysis. Journal of clinical immunology 32:530-539.

Liang YL, Wu H, Shen X, Li PQ, Yang XQ, Liang L, Tian WH, Zhang LF, Xie XD (2012) Association of STAT4 rs7574865 polymorphism with autoimmune diseases: a meta-analysis. Molecular biology reports 39:8873-8882.

Okada Y, Mori M, Yamada R, Suzuki A, Kobayashi K, Kubo M, Nakamura Y, Yamamoto K (2008) SLC22A4 polymorphism and rheumatoid arthritis susceptibility: a replication study in a Japanese population and a metaanalysis. The Journal of rheumatology 35:1723-1728.

Patsopoulos NA, Ioannidis JP (2010) Susceptibility variants for rheumatoid arthritis in the TRAF1-C5 and 6q23 loci: a meta-analysis. Annals of the rheumatic diseases 69:561-566.

Robinson JI, Barrett JH, Taylor JC, Naven M, Corscadden D, Barton A, Wilson AG, Emery P, Isaacs JD, Morgan AW (2010) Dissection of the FCGR3A association with RA: increased association in men and with autoantibody positive disease. Annals of the rheumatic diseases 69:1054-1057.

Song b GG, Bae SC, Choi SJ, Ji JD, Lee YH (2012) Associations between interleukin-23 receptor polymorphisms and susceptibility to rheumatoid arthritis: a meta-analysis. Molecular biology reports 39:10655-10663.

Song c GG, Bae SC, Lee YH (2012) The glutathione S-transferase M1 and P1 polymorphisms and rheumatoid arthritis: a meta-analysis. Molecular biology reports 39:10739-10745.

Sugiyama D, Nishimura K, Tamaki K, Tsuji G, Nakazawa T, Morinobu A, Kumagai S (2010) Impact of smoking as a risk factor for developing rheumatoid arthritis: a meta-analysis of observational studies. Annals of the rheumatic diseases 69:70-81.

Xue J, Mou L, Lu X, Wu H (2010) No association of interleukin-1 receptor antagonist VNTR polymorphism and rheumatoid arthritis susceptibility: a meta analysis. Clinical and experimental rheumatology 28:654-660.
