# Supplementary material for: Replication Validity of Initial Association Studies: A Comparison between Psychiatry, Neurology and Four Somatic Diseases
Source: PLoS One. 2016 Jun 23;11(6):e0158064. doi: 10.1371/journal.pone.0158064 (PMC4919034; doi:10.1371/journal.pone.0158064)
Supplement: S2 Text — (DOCX) [file pone.0158064.s004.docx]

**References for Initial studies**

**ADHD**

Barr CL, Feng Y, Wigg K, Bloom S, Roberts W, Malone M, Schachar R, Tannock R, Kennedy JL (2000) Identification of DNA variants in the SNAP-25 gene and linkage study of these polymorphisms and attention-deficit hyperactivity disorder. Mol Psychiatry 5:405-409.

Barr CL, Feng Y, Wigg KG, Schachar R, Tannock R, Roberts W, Malone M, Kennedy JL (2001) 5'-untranslated region of the dopamine D4 receptor gene and attention-deficit hyperactivity disorder. American journal of medical genetics 105:84-90.

Borger N, van der Meere J (2000) Motor control and state regulation in children with ADHD: a cardiac response study. Biological psychology 51:247-267.

Busby K, Firestone P, Pivik RT (1981) Sleep patterns in hyperkinetic and normal children. Sleep 4:366-383.

Castellanos FX, Giedd JN, Eckburg P, Marsh WL, Vaituzis AC, Kaysen D, Hamburger SD, Rapoport JL (1994) Quantitative morphology of the caudate nucleus in attention deficit hyperactivity disorder. The American journal of psychiatry 151:1791-1796.

Comings DE, Comings BG, Muhleman D, Dietz G, Shahbahrami B, Tast D, Knell E, Kocsis P, Baumgarten R, Kovacs BW, et al. (1991) The dopamine D2 receptor locus as a modifying gene in neuropsychiatric disorders. JAMA 266:1793-1800.

Cook EH, Jr., Stein MA, Krasowski MD, Cox NJ, Olkon DM, Kieffer JE, Leventhal BL (1995) Association of attention-deficit disorder and the dopamine transporter gene. American journal of human genetics 56:993-998.

Daly G, Hawi Z, Fitzgerald M, Gill M (1999) Mapping susceptibility loci in attention deficit hyperactivity disorder: preferential transmission of parental alleles at DAT1, DBH and DRD5 to affected children. Mol Psychiatry 4:192-196.

Dougherty DD, Bonab AA, Spencer TJ, Rauch SL, Madras BK, Fischman AJ (1999) Dopamine transporter density in patients with attention deficit hyperactivity disorder. Lancet 354:2132-2133.

Eisenberg J, Mei-Tal G, Steinberg A, Tartakovsky E, Zohar A, Gritsenko I, Nemanov L, Ebstein RP (1999) Haplotype relative risk study of catechol-O-methyltransferase (COMT) and attention deficit hyperactivity disorder (ADHD): association of the high-enzyme activity Val allele with ADHD impulsive-hyperactive phenotype. American journal of medical genetics 88:497-502.

Friedel S, Horro FF, Wermter AK, Geller F, Dempfle A, Reichwald K, Smidt J, Bronner G, Konrad K, Herpertz-Dahlmann B, Warnke A, Hemminger U, Linder M, Kiefl H, Goldschmidt HP, Siegfried W, Remschmidt H, Hinney A, Hebebrand J (2005) Mutation screen of the brain derived neurotrophic factor gene (BDNF): identification of several genetic variants and association studies in patients with obesity, eating disorders, and attention-deficit/hyperactivity disorder. American journal of medical genetics 132B:96-99.

Gorenstein EE, Mammato CA, Sandy JM (1989) Performance of inattentive-overactive children on selected measures of prefrontal-type function. Journal of clinical psychology 45:619-632.

Greenhill L, Puig-Antich J, Goetz R, Hanlon C, Davies M (1983) Sleep architecture and REM sleep measures in prepubertal children with attention deficit disorder with hyperactivity. Sleep 6:91-101.

Harper GW, Ottinger DR (1992) The performance of hyperactive and control preschoolers on a new computerized measure of visual vigilance: the Preschool Vigilance Task. Journal of child psychology and psychiatry, and allied disciplines 33:1365-1372.

Hawi Z, Dring M, Kirley A, Foley D, Kent L, Craddock N, Asherson P, Curran S, Gould A, Richards S, Lawson D, Pay H, Turic D, Langley K, Owen M, O'Donovan M, Thapar A, Fitzgerald M, Gill M (2002) Serotonergic system and attention deficit hyperactivity disorder (ADHD): a potential susceptibility locus at the 5-HT(1B) receptor gene in 273 nuclear families from a multi-centre sample. Mol Psychiatry 7:718-725.

Horn WF, Wagner AE, Ialongo N (1989) Sex differences in school-aged children with pervasive attention deficit hyperactivity disorder. Journal of abnormal child psychology 17:109-125.

Hughes C, Dunn J, White A (1998) Trick or treat?: uneven understanding of mind and emotion and executive dysfunction in "hard-to-manage" preschoolers. Journal of child psychology and psychiatry, and allied disciplines 39:981-994.

Jansen LM, Gispen-de Wied CC, Jansen MA, van der Gaag RJ, Matthys W, van Engeland H (1999) Pituitary-adrenal reactivity in a child psychiatric population: salivary cortisol response to stressors. European neuropsychopharmacology : the journal of the European College of Neuropsychopharmacology 9:67-75.

Kempton S, Vance A, Maruff P, Luk E, Costin J, Pantelis C (1999) Executive function and attention deficit hyperactivity disorder: stimulant medication and better executive function performance in children. Psychological medicine 29:527-538.

Konofal E, Lecendreux M, Arnulf I, Mouren MC (2004) Iron deficiency in children with attention-deficit/hyperactivity disorder. Archives of pediatrics & adolescent medicine 158:1113-1115.

LaHoste GJ, Swanson JM, Wigal SB, Glabe C, Wigal T, King N, Kennedy JL (1996) Dopamine D4 receptor gene polymorphism is associated with attention deficit hyperactivity disorder. Mol Psychiatry 1:121-124.

Manor I, Eisenberg J, Tyano S, Sever Y, Cohen H, Ebstein RP, Kotler M (2001) Family-based association study of the serotonin transporter promoter region polymorphism (5-HTTLPR) in attention deficit hyperactivity disorder. American journal of medical genetics 105:91-95.

Mariani MA, Barkley RA (1997) Neuropsychological and academic functioning in preschool boys with attention deficit hyperactivity disorder. Developmental neuropsychology 13:111-129.

Monastra VJ, Lubar JF, Linden M, VanDeusen P, Green G, Wing W, Phillips A, Fenger TN (1999) Assessing attention deficit hyperactivity disorder via quantitative electroencephalography: an initial validation study. Neuropsychology 13:424-433.

Rapoport JL, Lott IT, Alexander DF, Abramson AU (1970) Urinary noradrenaline and playroom behaviour in hyperactive boys. Lancet 296:1141.

Schachar R, Logan GD (1990) Impulsivity and inhibitory control in normal development and childhood psychopathology. Developmental Psychology 26:710-720.

Shue KL, Douglas VI (1992) Attention deficit hyperactivity disorder and the frontal lobe syndrome. Brain and cognition 20:104-124.

Sonuga-Barke EJ, Dalen L, Daley D, Remington B (2002) Are planning, working memory, and inhibition associated with individual differences in preschool ADHD symptoms? Developmental neuropsychology 21:255-272.

Wender PH, Epstein RS, Kopin IJ, Gordon EK (1971) Urinary monoamine metabolites in children with minimal brain dysfunction. The American journal of psychiatry 127:1411-1415.

Werry JS, Quay HC (1969) Observing the classroom behavior of elementary school children. Exceptional children 35:461-470.

Wiersema JR, van der Meere JJ, Roeyers H (2009) ERP correlates of error monitoring in adult ADHD. Journal of neural transmission (Vienna, Austria : 1996) 116:371-379.

Williams JI, Cram DM, Tausig FT, Webster E (1978) Relative effects of drugs and diet on hyperactive behaviors: an experimental study. Pediatrics 61:811-817.

Xu C, Schachar R, Tannock R, Roberts W, Malone M, Kennedy JL, Barr CL (2001) Linkage study of the alpha2A adrenergic receptor in attention-deficit hyperactivity disorder families. American journal of medical genetics 105:159-162.

Zoroglu SS, Erdal ME, Alasehirli B, Erdal N, Sivasli E, Tutkun H, Savas HA, Herken H (2002) Significance of serotonin transporter gene 5-HTTLPR and variable number of tandem repeat polymorphism in attention deficit hyperactivity disorder. Neuropsychobiology 45:176-181.

Zuckerman B, Stevenson J, Bailey V (1987) Sleep problems in early childhood: continuities, predictive factors, and behavioral correlates. Pediatrics 80:664-671.

**Autism**

Cook EH, Jr., Courchesne R, Lord C, Cox NJ, Yan S, Lincoln A, Haas R, Courchesne E, Leventhal BL (1997) Evidence of linkage between the serotonin transporter and autistic disorder. Molecular psychiatry 2:247-250.

Courchesne E, Karns CM, Davis HR, Ziccardi R, Carper RA, Tigue ZD, Chisum HJ, Moses P, Pierce K, Lord C, Lincoln AJ, Pizzo S, Schreibman L, Haas RH, Akshoomoff NA, Courchesne RY (2001) Unusual brain growth patterns in early life in patients with autistic disorder: an MRI study. Neurology 57:245-254.

Courchesne E, Yeung-Courchesne R, Press GA, Hesselink JR, Jernigan TL (1988) Hypoplasia of cerebellar vermal lobules VI and VII in autism. The New England journal of medicine 318:1349-1354.

D'Eufemia P, Finocchiaro R, Celli M, Viozzi L, Monteleone D, Giardini O (1995) Low serum tryptophan to large neutral amino acids ratio in idiopathic infantile autism. Biomedicine & pharmacotherapy = Biomedecine & pharmacotherapie 49:288-292.

Ermer J, Dunn W (1998) The sensory profile: a discriminant analysis of children with and without disabilities. The American journal of occupational therapy : official publication of the American Occupational Therapy Association 52:283-290.

Finegan JA, Quarrington B (1979) Pre-, peri-, and neonatal factors and infantile autism. Journal of child psychology and psychiatry, and allied disciplines 20:119-128.

Gaffney GR, Kuperman S, Tsai LY, Minchin S, Hassanein KM (1987) Midsagittal magnetic resonance imaging of autism. The British journal of psychiatry : the journal of mental science 151:831-833.

Glasson EJ, Bower C, Petterson B, de Klerk N, Chaney G, Hallmayer JF (2004) Perinatal factors and the development of autism: a population study. Archives of general psychiatry 61:618-627.

Hisaoka S, Harada M, Nishitani H, Mori K (2001) Regional magnetic resonance spectroscopy of the brain in autistic individuals. Neuroradiology 43:496-498.

Ingram JL, Stodgell CJ, Hyman SL, Figlewicz DA, Weitkamp LR, Rodier PM (2000) Discovery of allelic variants of HOXA1 and HOXB1: genetic susceptibility to autism spectrum disorders. Teratology 62:393-405.

James SJ, Cutler P, Melnyk S, Jernigan S, Janak L, Gaylor DW, Neubrander JA (2004) Metabolic biomarkers of increased oxidative stress and impaired methylation capacity in children with autism. The American journal of clinical nutrition 80:1611-1617.

Kleiman MD, Neff S, Rosman NP (1992) The brain in infantile autism: are posterior fossa structures abnormal? Neurology 42:753-760.

Larsson HJ, Eaton WW, Madsen KM, Vestergaard M, Olesen AV, Agerbo E, Schendel D, Thorsen P, Mortensen PB (2005) Risk factors for autism: perinatal factors, parental psychiatric history, and socioeconomic status. American journal of epidemiology 161:916-925; discussion 926-918.

Murphy DG, Critchley HD, Schmitz N, McAlonan G, Van Amelsvoort T, Robertson D, Daly E, Rowe A, Russell A, Simmons A, Murphy KC, Howlin P (2002) Asperger syndrome: a proton magnetic resonance spectroscopy study of brain. Archives of general psychiatry 59:885-891.

Otsuka H, Harada M, Mori K, Hisaoka S, Nishitani H (1999) Brain metabolites in the hippocampus-amygdala region and cerebellum in autism: an 1H-MR spectroscopy study. Neuroradiology 41:517-519.

Piven J, Arndt S, Bailey J, Havercamp S, Andreasen NC, Palmer P (1995) An MRI study of brain size in autism. The American journal of psychiatry 152:1145-1149.

Steg JP, Rapoport JL (1975) Minor physical anomalies in normal, neurotic, learning disabled, and severely disturbed children. Journal of autism and childhood schizophrenia 5:299-307.

Vilensky JA, Damasio AR, Maurer RG (1981) Gait disturbances in patients with autistic behavior: a preliminary study. Archives of neurology 38:646-649.

**Major Depressive Disorder**

Arias B, Arranz MJ, Gasto C, Catalan R, Pintor L, Gutierrez B, Kerwin RW, Fananas L (2002) Analysis of structural polymorphisms and C-1018G promoter variant of the 5-HT(1A) receptor gene as putative risk factors in major depression. Molecular psychiatry 7:930-932.

Arinami T, Li L, Mitsushio H, Itokawa M, Hamaguchi H, Toru M (1996) An insertion/deletion polymorphism in the angiotensin converting enzyme gene is associated with both brain substance P contents and affective disorders. Biological psychiatry 40:1122-1127.

Arinami T, Yamada N, Yamakawa-Kobayashi K, Hamaguchi H, Toru M (1997) Methylenetetrahydrofolate reductase variant and schizophrenia/depression. American journal of medical genetics 74:526-528.

Atkinson AK, Rickel AU (1984) Postpartum depression in primiparous parents. Journal of abnormal psychology 93:115-119.

Auer DP, Putz B, Kraft E, Lipinski B, Schill J, Holsboer F (2000) Reduced glutamate in the anterior cingulate cortex in depression: an in vivo proton magnetic resonance spectroscopy study. Biological psychiatry 47:305-313.

Ballmaier M, Toga AW, Blanton RE, Sowell ER, Lavretsky H, Peterson J, Pham D, Kumar A (2004) Anterior cingulate, gyrus rectus, and orbitofrontal abnormalities in elderly depressed patients: an MRI-based parcellation of the prefrontal cortex. The American journal of psychiatry 161:99-108.

Blazer D, Williams CD (1980) Epidemiology of dysphoria and depression in an elderly population. The American journal of psychiatry 137:439-444.

Brambilla F, Maggioni M (1998) Blood levels of cytokines in elderly patients with major depressive disorder. Acta psychiatrica Scandinavica 97:309-313.

Breslau N, Davis GC, Prabucki K (1987) Searching for evidence on the validity of generalized anxiety disorder: psychopathology in children of anxious mothers. Psychiatry research 20:285-297.

Caspi A, Sugden K, Moffitt TE, Taylor A, Craig IW, Harrington H, McClay J, Mill J, Martin J, Braithwaite A, Poulton R (2003) Influence of life stress on depression: moderation by a polymorphism in the 5-HTT gene. Science 301:386-389.

Coffey CE, Figiel GS, Djang WT, Weiner RD (1990) Subcortical hyperintensity on magnetic resonance imaging: a comparison of normal and depressed elderly subjects. The American journal of psychiatry 147:187-189.

Coffey CE, Wilkinson WE, Weiner RD, Parashos IA, Djang WT, Webb MC, Figiel GS, Spritzer CE (1993) Quantitative cerebral anatomy in depression. A controlled magnetic resonance imaging study. Archives of general psychiatry 50:7-16.

Collier DA, Stober G, Li T, Heils A, Catalano M, Di Bella D, Arranz MJ, Murray RM, Vallada HP, Bengel D, Muller CR, Roberts GW, Smeraldi E, Kirov G, Sham P, Lesch KP (1996) A novel functional polymorphism within the promoter of the serotonin transporter gene: possible role in susceptibility to affective disorders. Molecular psychiatry 1:453-460.

Dawson ME, Schell AM, Catania JJ (1977) Autonomic correlates of depression and clinical improvement following electroconvulsive shock therapy. Psychophysiology 14:569-578.

Drevets WC, Price JL, Simpson JR, Jr., Todd RD, Reich T, Vannier M, Raichle ME (1997) Subgenual prefrontal cortex abnormalities in mood disorders. Nature 386:824-827.

Extein I, Rosenberg G, Pottash AL, Gold MS (1982) The dexamethasone suppression test in depressed adolescents. The American journal of psychiatry 139:1617-1619.

Fan AP, Eaton WW (2001) Longitudinal study assessing the joint effects of socio-economic status and birth risks on adult emotional and nervous conditions. The British journal of psychiatry Supplement 40:s78-83.

Fawcett J, York R (1986) Spouses' physical and psychological symptoms during pregnancy and the postpartum. Nursing research 35:144-148.

Galard R, Gallart JM, Catalan R, Schwartz S, Arguello JM, Castellanos JM (1991) Salivary cortisol levels and their correlation with plasma ACTH levels in depressed patients before and after the DST. The American journal of psychiatry 148:505-508.

Greden JF, Genero N, Price HL, Feinberg M, Levine S (1986) Facial electromyography in depression. Subgroup differences. Archives of general psychiatry 43:269-274.

Hariri AR, Mattay VS, Tessitore A, Kolachana B, Fera F, Goldman D, Egan MF, Weinberger DR (2002) Serotonin transporter genetic variation and the response of the human amygdala. Science (New York, NY) 297:400-403.

Hickie I, Scott E, Naismith S, Ward PB, Turner K, Parker G, Mitchell P, Wilhelm K (2001) Late-onset depression: genetic, vascular and clinical contributions. Psychological medicine 31:1403-1412.

Husain MM, McDonald WM, Doraiswamy PM, Figiel GS, Na C, Escalona PR, Boyko OB, Nemeroff CB, Krishnan KR (1991) A magnetic resonance imaging study of putamen nuclei in major depression. Psychiatry research 40:95-99.

Kaney S, Bowen-Jones K, Bentall RP (1999) Persecutory delusions and autobiographical memory. The British journal of clinical psychology / the British Psychological Society 38 ( Pt 1):97-102.

Karege F, Perret G, Bondolfi G, Schwald M, Bertschy G, Aubry JM (2002) Decreased serum brain-derived neurotrophic factor levels in major depressed patients. Psychiatry research 109:143-148.

Krishnan KR, Doraiswamy PM, Lurie SN, Figiel GS, Husain MM, Boyko OB, Ellinwood EH, Jr., Nemeroff CB (1991) Pituitary size in depression. The Journal of clinical endocrinology and metabolism 72:256-259.

Krishnan KR, McDonald WM, Doraiswamy PM, Tupler LA, Husain M, Boyko OB, Figiel GS, Ellinwood EH, Jr. (1993) Neuroanatomical substrates of depression in the elderly. European archives of psychiatry and clinical neuroscience 243:41-46.

Krishnan KR, McDonald WM, Escalona PR, Doraiswamy PM, Na C, Husain MM, Figiel GS, Boyko OB, Ellinwood EH, Nemeroff CB (1992) Magnetic resonance imaging of the caudate nuclei in depression. Preliminary observations. Archives of general psychiatry 49:553-557.

Kubera M, Kenis G, Bosmans E, Zieba A, Dudek D, Nowak G, Maes M (2000) Plasma levels of interleukin-6, interleukin-10, and interleukin-1 receptor antagonist in depression: comparison between the acute state and after remission. Polish journal of pharmacology 52:237-241.

Kumar A, Miller D, Ewbank D, Yousem D, Newberg A, Samuels S, Cowell P, Gottlieb G (1997) Quantitative anatomic measures and comorbid medical illness in late-life major depression. The American journal of geriatric psychiatry : official journal of the American Association for Geriatric Psychiatry 5:15-25.

Kuyken W, Dalgleish T (1995) Autobiographical memory and depression. The British journal of clinical psychology / the British Psychological Society 34 ( Pt 1):89-92.

Lafont V, Medecin I, Robert PH, Beaulieu FE, Kazes M, Danion JM, Pringuey D, Darcourt G (1998) Initiation and supervisory processes in schizophrenia and depression. Schizophrenia research 34:49-57.

MacQueen GM, Campbell S, McEwen BS, Macdonald K, Amano S, Joffe RT, Nahmias C, Young LT (2003) Course of illness, hippocampal function, and hippocampal volume in major depression. Proceedings of the National Academy of Sciences of the United States of America 100:1387-1392.

Maes b M, Meltzer HY, Buckley P, Bosmans E (1995) Plasma-soluble interleukin-2 and transferrin receptor in schizophrenia and major depression. European archives of psychiatry and clinical neuroscience 244:325-329.

Parashos IA, Tupler LA, Blitchington T, Krishnan KR (1998) Magnetic-resonance morphometry in patients with major depression. Psychiatry research 84:7-15.

Penninx BW, Guralnik JM, Ferrucci L, Fried LP, Allen RH, Stabler SP (2000) Vitamin B(12) deficiency and depression in physically disabled older women: epidemiologic evidence from the Women's Health and Aging Study. The American journal of psychiatry 157:715-721.

Phifer JF, Murrell SA (1986) Etiologic factors in the onset of depressive symptoms in older adults. Journal of abnormal psychology 95:282-291.

Ravnkilde B, Videbech P, Clemmensen K, Egander A, Rasmussen NA, Rosenberg R (2002) Cognitive deficits in major depression. Scandinavian journal of psychology 43:239-251.

Roberts RE, Shema SJ, Kaplan GA, Strawbridge WJ (2000) Sleep complaints and depression in an aging cohort: A prospective perspective. The American journal of psychiatry 157:81-88.

Scott ML, Golden CJ, Ruedrich SL, Bishop RJ (1983) Ventricular enlargement in major depression. Psychiatry research 8:91-93.

Sheline YI, Gado MH, Price JL (1998) Amygdala core nuclei volumes are decreased in recurrent major depression. Neuroreport 9:2023-2028.

Sheline YI, Wang PW, Gado MH, Csernansky JG, Vannier MW (1996) Hippocampal atrophy in recurrent major depression. Proceedings of the National Academy of Sciences of the United States of America 93:3908-3913.

Smith MJ, Brebion G, Banquet JP, Allilaire JF (1994) Experimental evidence for two dimensions of cognitive disorders in depressives. Journal of psychiatric research 28:401-411.

Thorpe KJ, Dragonas T, Golding J (1992) The effects of psychosocial factors on the mother’s emotional well- being during early parenthood: a cross-cultural study of Britian and Greece. J Reprod Infant Psychol 10:205-217.

Tsai SJ, Cheng CY, Yu YW, Chen TJ, Hong CJ (2003) Association study of a brain-derived neurotrophic-factor genetic polymorphism and major depressive disorders, symptomatology, and antidepressant response. American journal of medical genetics Part B, Neuropsychiatric genetics : the official publication of the International Society of Psychiatric Genetics 123B:19-22.

Zubenko GS, Henderson R, Stiffler JS, Stabler S, Rosen J, Kaplan BB (1996) Association of the APOE epsilon 4 allele with clinical subtypes of late life depression. Biological psychiatry 40:1008-1016.

Zubenko GS, Sullivan P, Nelson JP, Belle SH, Huff FJ, Wolf GL (1990) Brain imaging abnormalities in mental disorders of late life. Archives of neurology 47:1107-1111.

Zuroff DC, Colussy SA (1986) Emotion recognition in schizophrenic and depressed inpatients. Journal of clinical psychology 42:411-417.

**Schizophrenia**

Abdalla DS, Monteiro HP, Oliveira JA, Bechara EJ (1986) Activities of superoxide dismutase and glutathione peroxidase in schizophrenic and manic-depressive patients. Clinical chemistry 32:805-807.

Akyol O, Herken H, Uz E, Fadillioglu E, Unal S, Sogut S, Ozyurt H, Savas HA (2002) The indices of endogenous oxidative and antioxidative processes in plasma from schizophrenic patients. The possible role of oxidant/antioxidant imbalance. Progress in neuro-psychopharmacology & biological psychiatry 26:995-1005.

Albee GW, Lane EA, Reuter JM (1964) CHILDHOOD INTELLIGENCE OF FUTURE SCHIZOPHRENICS AND NEIGHBORHOOD PEERS. The Journal of psychology 58:141-144.

Albrecht P, Torrey EF, Boone E, Hicks JT, Daniel N (1980) Raised cytomegalovirus-antibody level in cerebrospinal fluid of schizophrenic patients. Lancet 2:769-772.

Andreasen NC, Ehrhardt JC, Swayze VW, 2nd, Alliger RJ, Yuh WT, Cohen G, Ziebell S (1990) Magnetic resonance imaging of the brain in schizophrenia. The pathophysiologic significance of structural abnormalities. Archives of general psychiatry 47:35-44.

Andreasen NC, Flashman L, Flaum M, Arndt S, Swayze V, 2nd, O'Leary DS, Ehrhardt JC, Yuh WT (1994) Regional brain abnormalities in schizophrenia measured with magnetic resonance imaging. JAMA : the journal of the American Medical Association 272:1763-1769.

Arinami a T, Gao M, Hamaguchi H, Toru M (1997) A functional polymorphism in the promoter region of the dopamine D2 receptor gene is associated with schizophrenia. Human molecular genetics 6:577-582.

Arinami b T, Yamada N, Yamakawa-Kobayashi K, Hamaguchi H, Toru M (1997) Methylenetetrahydrofolate reductase variant and schizophrenia/depression. American journal of medical genetics 74:526-528.

Arinami T, Itokawa M, Enguchi H, Tagaya H, Yano S, Shimizu H, Hamaguchi H, Toru M (1994) Association of dopamine D2 receptor molecular variant with schizophrenia. Lancet 343:703-704.

Baron M (1982) Genetic models of schizophrenia. Acta psychiatrica Scandinavica 65:263-275.

Barta PE, Pearlson GD, Powers RE, Richards SS, Tune LE (1990) Auditory hallucinations and smaller superior temporal gyral volume in schizophrenia. The American journal of psychiatry 147:1457-1462.

Bartha R, Williamson PC, Drost DJ, Malla A, Carr TJ, Cortese L, Canaran G, Rylett RJ, Neufeld RW (1997) Measurement of glutamate and glutamine in the medial prefrontal cortex of never-treated schizophrenic patients and healthy controls by proton magnetic resonance spectroscopy. Archives of general psychiatry 54:959-965.

Blackwood DH, Whalley LJ, Christie JE, Blackburn IM, St Clair DM, McInnes A (1987) Changes in auditory P3 event-related potential in schizophrenia and depression. The British journal of psychiatry : the journal of mental science 150:154-160.

Bullen JG, Hemsley DR (1987) Schizophrenia: a failure to control the contents of consciousness? The British journal of clinical psychology / the British Psychological Society 26 ( Pt 1):25-33.

Carter CS, Robertson LC, Nordahl TE, O'Shora-Celaya LJ, Chaderjian MC (1993) Abnormal processing of irrelevant information in schizophrenia: the role of illness subtype. Psychiatry research 48:17-26.

Choe BY, Kim KT, Suh TS, Lee C, Paik IH, Bahk YW, Shinn KS, Lenkinski RE (1994) 1H magnetic resonance spectroscopy characterization of neuronal dysfunction in drug-naive, chronic schizophrenia. Academic radiology 1:211-216.

Chumakov I, Blumenfeld M, Guerassimenko O, Cavarec L, Palicio M, Abderrahim H, Bougueleret L, Barry C, Tanaka H, La Rosa P, Puech A, Tahri N, Cohen-Akenine A, Delabrosse S, Lissarrague S, Picard FP, Maurice K, Essioux L, Millasseau P, Grel P, Debailleul V, Simon AM, Caterina D, Dufaure I, Malekzadeh K, Belova M, Luan JJ, Bouillot M, Sambucy JL, Primas G, Saumier M, Boubkiri N, Martin-Saumier S, Nasroune M, Peixoto H, Delaye A, Pinchot V, Bastucci M, Guillou S, Chevillon M, Sainz-Fuertes R, Meguenni S, Aurich-Costa J, Cherif D, Gimalac A, Van Duijn C, Gauvreau D, Ouellette G, Fortier I, Raelson J, Sherbatich T, Riazanskaia N, Rogaev E, Raeymaekers P, Aerssens J, Konings F, Luyten W, Macciardi F, Sham PC, Straub RE, Weinberger DR, Cohen N, Cohen D (2002) Genetic and physiological data implicating the new human gene G72 and the gene for D-amino acid oxidase in schizophrenia. Proceedings of the National Academy of Sciences of the United States of America 99:13675-13680.

Collier DA, Arranz MJ, Sham P, Battersby S, Vallada H, Gill P, Aitchison KJ, Sodhi M, Li T, Roberts GW, Smith B, Morton J, Murray RM, Smith D, Kirov G (1996) The serotonin transporter is a potential susceptibility factor for bipolar affective disorder. Neuroreport 7:1675-1679.

Collins L, AL S (1966) Pain sensitivity, age and activity level in chronic schizophrenics and normals. The British journal of psychiatry : the journal of mental science 112:33-35.

Comings DE, Comings BG, Muhleman D, Dietz G, Shahbahrami B, Tast D, Knell E, Kocsis P, Baumgarten R, Kovacs BW, et al. (1991) The dopamine D2 receptor locus as a modifying gene in neuropsychiatric disorders. JAMA : the journal of the American Medical Association 266:1793-1800.

Corcoran R, Mercer G, Frith CD (1995) Schizophrenia, symptomatology and social inference: investigating "theory of mind" in people with schizophrenia. Schizophrenia research 17:5-13.

Corrigan PW, Addis IB (1995) The effects of cognitive complexity on a social sequencing task in schizophrenia. Schizophrenia research 16:137-144.

Corrigan PW, Green MF (1993) Schizophrenic patients' sensitivity to social cues: the role of abstraction. The American journal of psychiatry 150:589-594.

Crawley JC, Crow TJ, Johnstone EC, Oldland SR, Owen F, Owens DG, Smith T, Veall N, Zanelli GD (1986) Uptake of 77Br-spiperone in the striata of schizophrenic patients and controls. Nuclear medicine communications 7:599-607.

Crespo-Facorro B, Kim J, Andreasen NC, O'Leary DS, Bockholt HJ, Magnotta V (2000) Insular cortex abnormalities in schizophrenia: a structural magnetic resonance imaging study of first-episode patients. Schizophrenia research 46:35-43.

Crocq MA, Mant R, Asherson P, Williams J, Hode Y, Mayerova A, Collier D, Lannfelt L, Sokoloff P, Schwartz JC, et al. (1992) Association between schizophrenia and homozygosity at the dopamine D3 receptor gene. Journal of medical genetics 29:858-860.

Cutting J, David A, Murphy D (1987) The nature of overinclusive thinking in schizophrenia. Psychopathology 20:213-219.

Das I, Ramchand CN, Gliddon A, Hirsch SR (1998) Nitric oxide, free radicals and polyamines may have a role in the membrane pathology of schizophrenia. Neuropsychobiology 37:65-67.

Deicken RF, Johnson C, Eliaz Y, Schuff N (2000) Reduced concentrations of thalamic N-acetylaspartate in male patients with schizophrenia. The American journal of psychiatry 157:644-647.

Deicken RF, Zhou L, Schuff N, Weiner MW (1997) Proton magnetic resonance spectroscopy of the anterior cingulate region in schizophrenia. Schizophrenia research 27:65-71.

DeLisi LE, Hoff AL, Schwartz JE, Shields GW, Halthore SN, Gupta SM, Henn FA, Anand AK (1991) Brain morphology in first-episode schizophrenic-like psychotic patients: a quantitative magnetic resonance imaging study. Biological psychiatry 29:159-175.

DeLisi LE, Tew W, Xie S, Hoff AL, Sakuma M, Kushner M, Lee G, Shedlack K, Smith AM, Grimson R (1995) A prospective follow-up study of brain morphology and cognition in first-episode schizophrenic patients: preliminary findings. Biological psychiatry 38:349-360.

Edwards J, Pattison PE, Jackson HJ, Wales RJ (2001) Facial affect and affective prosody recognition in first-episode schizophrenia. Schizophrenia research 48:235-253.

Egan MF, Kojima M, Callicott JH, Goldberg TE, Kolachana BS, Bertolino A, Zaitsev E, Gold B, Goldman D, Dean M, Lu B, Weinberger DR (2003) The BDNF val66met polymorphism affects activity-dependent secretion of BDNF and human memory and hippocampal function. Cell 112:257-269.

Elkashef AM, Buchanan RW, Gellad F, Munson RC, Breier A (1994) Basal ganglia pathology in schizophrenia and tardive dyskinesia: an MRI quantitative study. The American journal of psychiatry 151:752-755.

Elkins IJ, Cromwell RL (1994) Priming effects in schizophrenia: associative interference and facilitation as a function of visual context. Journal of abnormal psychology 103:791-800.

Emamian ES, Hall D, Birnbaum MJ, Karayiorgou M, Gogos JA (2004) Convergent evidence for impaired AKT1-GSK3beta signaling in schizophrenia. Nature genetics 36:131-137.

Faber R, Reichstein MB (1981) Language dysfunction in schizophrenia. The British journal of psychiatry : the journal of mental science 139:519-522.

Feinberg TE, Rifkin A, Schaffer C, Walker E (1986) Facial discrimination and emotional recognition in schizophrenia and affective disorders. Archives of general psychiatry 43:276-279.

Foong J, Maier M, Clark CA, Barker GJ, Miller DH, Ron MA (2000) Neuropathological abnormalities of the corpus callosum in schizophrenia: a diffusion tensor imaging study. Journal of neurology, neurosurgery, and psychiatry 68:242-244.

Friedman S, Harrison G (1984) Sexual histories, attitudes, and behavior of schizophrenic and "normal" women. Archives of sexual behavior 13:555-567.

Frith CD, Leary J, Cahill C, Johnstone EC (1991) Performance on psychological tests. Demographic and clinical correlates of the results of these tests. The British journal of psychiatry Supplement 26-29, 44-26.

Gejman PV, Ram A, Gelernter J, Friedman E, Cao Q, Pickar D, Blum K, Noble EP, Kranzler HR, O'Malley S, et al. (1994) No structural mutation in the dopamine D2 receptor gene in alcoholism or schizophrenia. Analysis using denaturing gradient gel electrophoresis. JAMA : the journal of the American Medical Association 271:204-208.

Gerber DJ, Hall D, Miyakawa T, Demars S, Gogos JA, Karayiorgou M, Tonegawa S (2003) Evidence for association of schizophrenia with genetic variation in the 8p21.3 gene, PPP3CC, encoding the calcineurin gamma subunit. Proceedings of the National Academy of Sciences of the United States of America 100:8993-8998.

Gold JM, Carpenter C, Randolph C, Goldberg TE, Weinberger DR (1997) Auditory working memory and Wisconsin Card Sorting Test performance in schizophrenia. Archives of general psychiatry 54:159-165.

Goldberg TE, Ragland JD, Torrey EF, Gold JM, Bigelow LB, Weinberger DR (1990) Neuropsychological assessment of monozygotic twins discordant for schizophrenia. Archives of general psychiatry 47:1066-1072.

Golden CJ (1976) Identification of brain disorders by the Stroop Color and Word Test. Journal of clinical psychology 32:654-658.

Goldstein JM, Goodman JM, Seidman LJ, Kennedy DN, Makris N, Lee H, Tourville J, Caviness VS, Jr., Faraone SV, Tsuang MT (1999) Cortical abnormalities in schizophrenia identified by structural magnetic resonance imaging. Archives of general psychiatry 56:537-547.

Gotlieb-Stematsky T, Zonis J, Arlazoroff A, Mozes T, Sigal M, Szekely AG (1981) Antibodies to Epstein-Barr virus, herpes simplex type 1, cytomegalovirus and measles virus in psychiatric patients. Archives of virology 67:333-339.

Green M, Walker E (1985) Neuropsychological performance and positive and negative symptoms in schizophrenia. Journal of abnormal psychology 94:460-469.

Green MF, Kern RS, Williams O, Mcgurk S, Kee K (1997) Procedural Learning in Schizophrenia: Evidence from Serial Reaction Time. Cognitive neuropsychiatry 2:123-134.

Grillon C, Ameli R, Glazer WM (1991) N400 and semantic categorization in schizophrenia. Biological psychiatry 29:467-480.

Gualtieri CT, Adams A, Shen CD, Loiselle D (1982) Minor physical anomalies in alcoholic and schizophrenic adults and hyperactive and autistic children. The American journal of psychiatry 139:640-643.

Gunduz H, Wu H, Ashtari M, Bogerts B, Crandall D, Robinson DG, Alvir J, Lieberman J, Kane J, Bilder R (2002) Basal ganglia volumes in first-episode schizophrenia and healthy comparison subjects. Biological psychiatry 51:801-808.

Gur RE, Cowell P, Turetsky BI, Gallacher F, Cannon T, Bilker W, Gur RC (1998) A follow-up magnetic resonance imaging study of schizophrenia. Relationship of neuroanatomical changes to clinical and neurobehavioral measures. Archives of general psychiatry 55:145-152.

Gur RE, Mozley PD, Resnick SM, Shtasel D, Kohn M, Zimmerman R, Herman G, Atlas S, Grossman R, Erwin R, et al. (1991) Magnetic resonance imaging in schizophrenia. I. Volumetric analysis of brain and cerebrospinal fluid. Archives of general psychiatry 48:407-412.

Harrison G, Owens D, Holton A, Neilson D, Boot D (1988) A prospective study of severe mental disorder in Afro-Caribbean patients. Psychological medicine 18:643-657.

Harvey PD (1985) Reality monitoring in mania and schizophrenia. The association of thought disorder and performance. The Journal of nervous and mental disease 173:67-73.

Harvey PD, Serper MR (1990) Linguistic and cognitive failures in schizophrenia. A multivariate analysis. The Journal of nervous and mental disease 178:487-493.

Hegerl U, Gaebel W, Gutzman H, Ulrich G (1988) Auditory evoked potentials as possible predictors of outcome in schizophrenic outpatients. International journal of psychophysiology : official journal of the International Organization of Psychophysiology 6:207-214.

Hodgkinson CA, Goldman D, Jaeger J, Persaud S, Kane JM, Lipsky RH, Malhotra AK (2004) Disrupted in schizophrenia 1 (DISC1): association with schizophrenia, schizoaffective disorder, and bipolar disorder. American journal of human genetics 75:862-872.

Hurwitz T, Kopala L, Clark C, Jones B (1988) Olfactory deficits in schizophrenia. Biological psychiatry 23:123-128.

Ishiguro H, Ohtsuki T, Toru M, Itokawa M, Aoki J, Shibuya H, Kurumaji A, Okubo Y, Iwawaki A, Ota K, Shimizu H, Hamaguchi H, Arinami T (1998) Association between polymorphisms in the type 1 sigma receptor gene and schizophrenia. Neuroscience letters 257:45-48.

Jernigan TL, Zisook S, Heaton RK, Moranville JT, Hesselink JR, Braff DL (1991) Magnetic resonance imaging abnormalities in lenticular nuclei and cerebral cortex in schizophrenia. Archives of general psychiatry 48:881-890.

Johanson E (1958) A study of schizophrenia in the male: a psychiatric and social study based on 138 cases with follow up. Acta psychiatrica et neurologica Scandinavica Supplementum 125:1-132.

Johnstone EC, Crow TJ, Frith CD, Stevens M, Kreel L, Husband J (1978) The dementia of dementia praecox. Acta psychiatrica Scandinavica 57:305-324.

Kasai K, Shenton ME, Salisbury DF, Onitsuka T, Toner SK, Yurgelun-Todd D, Kikinis R, Jolesz FA, McCarley RW (2003) Differences and similarities in insular and temporal pole MRI gray matter volume abnormalities in first-episode schizophrenia and affective psychosis. Archives of general psychiatry 60:1069-1077.

Kelsoe JR, Jr., Cadet JL, Pickar D, Weinberger DR (1988) Quantitative neuroanatomy in schizophrenia. A controlled magnetic resonance imaging study. Archives of general psychiatry 45:533-541.

Kendler KS, Hays P (1982) Familial and sporadic schizophrenia: a symptomatic, prognostic, and EEG comparison. The American journal of psychiatry 139:1557-1562.

Kenny JT, Friedman L, Findling RL, Swales TP, Strauss ME, Jesberger JA, Schulz SC (1997) Cognitive impairment in adolescents with schizophrenia. The American journal of psychiatry 154:1613-1615.

Killian GA, Holzman PS, Davis JM, Gibbons R (1984) Effects of psychotropic medication on selected cognitive and perceptual measures. Journal of abnormal psychology 93:58-70.

Kurachi M, Matsui M, Kiba K, Suzuki M, Tsunoda M, Yamaguchi N (1994) Limited visual search on the WAIS Picture Completion test in patients with schizophrenia. Schizophrenia research 12:75-80.

Laakso A, Vilkman H, Alakare B, Haaparanta M, Bergman J, Solin O, Peurasaari J, Rakkolainen V, Syvalahti E, Hietala J (2000) Striatal dopamine transporter binding in neuroleptic-naive patients with schizophrenia studied with positron emission tomography. The American journal of psychiatry 157:269-271.

Langdon R, Michie PT, Ward PB, McConaghy N, Catts SV, Coltheart M (1997) Defective self and/or other mentalising in schizophrenia: A cognitive neuropsychological approach. Cognitive Neuropsychiatry 2:167-193.

Li T, Sham PC, Vallada H, Xie T, Tang X, Murray RM, Liu X, Collier DA (1996) Preferential transmission of the high activity allele of COMT in schizophrenia. Psychiatric genetics 6:131-133.

Lim KO, Tew W, Kushner M, Chow K, Matsumoto B, DeLisi LE (1996) Cortical gray matter volume deficit in patients with first-episode schizophrenia. The American journal of psychiatry 153:1548-1553.

Liu H, Heath SC, Sobin C, Roos JL, Galke BL, Blundell ML, Lenane M, Robertson B, Wijsman EM, Rapoport JL, Gogos JA, Karayiorgou M (2002) Genetic variation at the 22q11 PRODH2/DGCR6 locus presents an unusual pattern and increases susceptibility to schizophrenia. Proceedings of the National Academy of Sciences of the United States of America 99:3717-3722.

Lo WS, Lau CF, Xuan Z, Chan CF, Feng GY, He L, Cao ZC, Liu H, Luan QM, Xue H (2004) Association of SNPs and haplotypes in GABAA receptor beta2 gene with schizophrenia. Molecular psychiatry 9:603-608.

Maes M, Bosmans E, Ranjan R, Vandoolaeghe E, Meltzer HY, De Ley M, Berghmans R, Stans G, Desnyder R (1996) Lower plasma CC16, a natural anti-inflammatory protein, and increased plasma interleukin-1 receptor antagonist in schizophrenia: effects of antipsychotic drugs. Schizophrenia research 21:39-50.

Maier M, Ron MA (1996) Hippocampal age-related changes in schizophrenia: a proton magnetic resonance spectroscopy study. Schizophrenia research 22:5-17.

Martinoli MG, Trojanowski JQ, Schmidt ML, Arnold SE, Fujiwara TM, Lee VM, Hurtig H, Julien JP, Clark C (1995) Association of apolipoprotein epsilon 4 allele and neuropathologic findings in patients with dementia. Acta neuropathologica 90:239-243.

McCarley RW, Shenton ME, O'Donnell BF, Faux SF, Kikinis R, Nestor PG, Jolesz FA (1993) Auditory P300 abnormalities and left posterior superior temporal gyrus volume reduction in schizophrenia. Archives of general psychiatry 50:190-197.

Morice R (1990) Cognitive inflexibility and pre-frontal dysfunction in schizophrenia and mania. The British journal of psychiatry : the journal of mental science 157:50-54.

Nanko S, Kunugi H, Hirasawa H, Kato N, Nabika T, Kobayashi S (2003) Brain-derived neurotrophic factor gene and schizophrenia: polymorphism screening and association analysis. Schizophrenia research 62:281-283.

Neufeld RW (1978) The nature of deficit among paranoid and nonparanoid schizophrenics in the interpretation of sentences: an information processing approach. Journal of clinical psychology 34:333-339.

Noga JT, Aylward E, Barta PE, Pearlson GD (1995) Cingulate gyrus in schizophrenic patients and normal volunteers. Psychiatry research 61:201-208.

O'Donovan MC, Craddock N, Norton N, Williams H, Peirce T, Moskvina V, Nikolov I, Hamshere M, Carroll L, Georgieva L, Dwyer S, Holmans P, Marchini JL, Spencer CC, Howie B, Leung HT, Hartmann AM, Moller HJ, Morris DW, Shi Y, Feng G, Hoffmann P, Propping P, Vasilescu C, Maier W, Rietschel M, Zammit S, Schumacher J, Quinn EM, Schulze TG, Williams NM, Giegling I, Iwata N, Ikeda M, Darvasi A, Shifman S, He L, Duan J, Sanders AR, Levinson DF, Gejman PV, Cichon S, Nothen MM, Gill M, Corvin A, Rujescu D, Kirov G, Owen MJ, Buccola NG, Mowry BJ, Freedman R, Amin F, Black DW, Silverman JM, Byerley WF, Cloninger CR (2008) Identification of loci associated with schizophrenia by genome-wide association and follow-up. Nature genetics 40:1053-1055.

Ohtsuki T, Inada T, Arinami T (2004) Failure to confirm association between AKT1 haplotype and schizophrenia in a Japanese case-control population. Molecular psychiatry 9:981-983.

Oie M, Rund BR (1999) Neuropsychological deficits in adolescent-onset schizophrenia compared with attention deficit hyperactivity disorder. The American journal of psychiatry 156:1216-1222.

Okuyama Y, Ishiguro H, Toru M, Arinami T (1999) A genetic polymorphism in the promoter region of DRD4 associated with expression and schizophrenia. Biochemical and biophysical research communications 258:292-295.

Paik I, Toh K, Kim J, Lee C (2000) TPH gene may be associated with suicidal behavior, but not with schizophrenia in the Korean population. Human heredity 50:365-369.

Pickup GJ, Frith CD (2001) Theory of mind impairments in schizophrenia: symptomatology, severity and specificity. Psychological medicine 31:207-220.

Reddy R, Sahebarao MP, Mukherjee S, Murthy JN (1991) Enzymes of the antioxidant defense system in chronic schizophrenic patients. Biological psychiatry 30:409-412.

Reith J, Benkelfat C, Sherwin A, Yasuhara Y, Kuwabara H, Andermann F, Bachneff S, Cumming P, Diksic M, Dyve SE, Etienne P, Evans AC, Lal S, Shevell M, Savard G, Wong DF, Chouinard G, Gjedde A (1994) Elevated dopa decarboxylase activity in living brain of patients with psychosis. Proceedings of the National Academy of Sciences of the United States of America 91:11651-11654.

Rhinewine JP, Lencz T, Thaden EP, Cervellione KL, Burdick KE, Henderson I, Bhaskar S, Keehlisen L, Kane J, Kohn N, Fisch GS, Bilder RM, Kumra S (2005) Neurocognitive profile in adolescents with early-onset schizophrenia: clinical correlates. Biological psychiatry 58:705-712.

Rossi A, De Cataldo S, Di Michele V, Manna V, Ceccoli S, Stratta P, Casacchia M (1990) Neurological soft signs in schizophrenia. The British journal of psychiatry : the journal of mental science 157:735-739.

Rossi A, Stratta P, Mattei P, Cupillari M, Bozzao A, Gallucci M, Casacchia M (1992) Planum temporale in schizophrenia: a magnetic resonance study. Schizophrenia research 7:19-22.

Rothhammer F, Pereira G, Camousseight A, Benado M (1971) Dermatoglyphics in schizophrenic patients. Human heredity 21:198-202.

Rwegellera GG (1977) Psychiatric morbidity among West Africans and West Indians living in London. Psychological medicine 7:317-329.

Sachs G, Steger-Wuchse D, Kryspin-Exner I, Gur RC, Katschnig H (2004) Facial recognition deficits and cognition in schizophrenia. Schizophrenia research 68:27-35.

Sazci A, Ergul E, Guzelhan Y, Kaya G, Kara I (2003) Methylenetetrahydrofolate reductase gene polymorphisms in patients with schizophrenia. Brain research Molecular brain research 117:104-107.

Shenton ME, Kikinis R, Jolesz FA, Pollak SD, LeMay M, Wible CG, Hokama H, Martin J, Metcalf D, Coleman M, et al. (1992) Abnormalities of the left temporal lobe and thought disorder in schizophrenia. A quantitative magnetic resonance imaging study. The New England journal of medicine 327:604-612.

Shifman S, Bronstein M, Sternfeld M, Pisante-Shalom A, Lev-Lehman E, Weizman A, Reznik I, Spivak B, Grisaru N, Karp L, Schiffer R, Kotler M, Strous RD, Swartz-Vanetik M, Knobler HY, Shinar E, Beckmann JS, Yakir B, Risch N, Zak NB, Darvasi A (2002) A highly significant association between a COMT haplotype and schizophrenia. American journal of human genetics 71:1296-1302.

Shintani F, Kanba S, Maruo N, Nakaki T, Nibuya M, Suzuki E, Kinoshita N, Yagi G (1991) Serum interleukin-6 in schizophrenic patients. Life sciences 49:661-664.

Sierra-Honigmann AM, Carbone KM, Yolken RH (1995) Polymerase chain reaction (PCR) search for viral nucleic acid sequences in schizophrenia. The British journal of psychiatry : the journal of mental science 166:55-60.

Snyder PJ, Bogerts B, Wu H, Bilder RM, Deoras KS, Lieberman JA (1998) Absence of the adhesio interthalamica as a marker of early developmental neuropathology in schizophrenia: an MRI and postmortem histologic study. Journal of neuroimaging : official journal of the American Society of Neuroimaging 8:159-163.

Stefansson H, Sigurdsson E, Steinthorsdottir V, Bjornsdottir S, Sigmundsson T, Ghosh S, Brynjolfsson J, Gunnarsdottir S, Ivarsson O, Chou TT, Hjaltason O, Birgisdottir B, Jonsson H, Gudnadottir VG, Gudmundsdottir E, Bjornsson A, Ingvarsson B, Ingason A, Sigfusson S, Hardardottir H, Harvey RP, Lai D, Zhou M, Brunner D, Mutel V, Gonzalo A, Lemke G, Sainz J, Johannesson G, Andresson T, Gudbjartsson D, Manolescu A, Frigge ML, Gurney ME, Kong A, Gulcher JR, Petursson H, Stefansson K (2002) Neuregulin 1 and susceptibility to schizophrenia. American journal of human genetics 71:877-892.

Straub RE, Jiang Y, MacLean CJ, Ma Y, Webb BT, Myakishev MV, Harris-Kerr C, Wormley B, Sadek H, Kadambi B, Cesare AJ, Gibberman A, Wang X, O'Neill FA, Walsh D, Kendler KS (2002) Genetic variation in the 6p22.3 gene DTNBP1, the human ortholog of the mouse dysbindin gene, is associated with schizophrenia. American journal of human genetics 71:337-348.

Suddath RL, Casanova MF, Goldberg TE, Daniel DG, Kelsoe JR, Jr., Weinberger DR (1989) Temporal lobe pathology in schizophrenia: a quantitative magnetic resonance imaging study. The American journal of psychiatry 146:464-472.

Suddath RL, Christison GW, Torrey EF, Casanova MF, Weinberger DR (1990) Anatomical abnormalities in the brains of monozygotic twins discordant for schizophrenia. The New England journal of medicine 322:789-794.

Swayze VW, 2nd, Andreasen NC, Alliger RJ, Yuh WT, Ehrhardt JC (1992) Subcortical and temporal structures in affective disorder and schizophrenia: a magnetic resonance imaging study. Biological psychiatry 31:221-240.

Tatsumi M, Sasaki T, Sakai T, Kamijima K, Fukuda R, Kunugi H, Hattori M, Nanko S (1997) Genes for interleukin-2 receptor beta chain, interleukin-1 beta, and schizophrenia: no evidence for the association or linkage. American journal of medical genetics 74:338-341.

Toyooka K, Asama K, Watanabe Y, Muratake T, Takahashi M, Someya T, Nawa H (2002) Decreased levels of brain-derived neurotrophic factor in serum of chronic schizophrenic patients. Psychiatry research 110:249-257.

Vaddadi KS, Gilleard CJ, Mindham RH, Butler R (1986) A controlled trial of prostaglandin E1 precursor in chronic neuroleptic resistant schizophrenic patients. Psychopharmacology 88:362-367.

Walker E, McGuire M, Bettes B (1984) Recognition and identification of facial stimuli by schizophrenics and patients with affective disorders. The British journal of clinical psychology / the British Psychological Society 23 ( Pt 1):37-44.

Whitworth AB, Honeder M, Kremser C, Kemmler G, Felber S, Hausmann A, Wanko C, Wechdorn H, Aichner F, Stuppaeck CH, Fleischhacker WW (1998) Hippocampal volume reduction in male schizophrenic patients. Schizophrenia research 31:73-81.

Wiesmann M, Wandinger KP, Missler U, Eckhoff D, Rothermundt M, Arolt V, Kirchner H (1999) Elevated plasma levels of S-100b protein in schizophrenic patients. Biological psychiatry 45:1508-1511.

Wilcox JA, Nasrallah HA (1987) Childhood head trauma and psychosis. Psychiatry research 21:303-306.

Wood LS, Pickering EH, Dechairo BM (2007) Significant support for DAO as a schizophrenia susceptibility locus: examination of five genes putatively associated with schizophrenia. Biological psychiatry 61:1195-1199.

Wu J, Buchsbaum MS, Moy K, Denlea N, Kesslak P, Tseng H, Plosnaj D, Hetu M, Potkin S, Bracha S, et al. (1993) Olfactory memory in unmedicated schizophrenics. Schizophrenia research 9:41-47.

Yolken RH, Bachmann S, Ruslanova I, Lillehoj E, Ford G, Torrey EF, Schroeder J (2001) Antibodies to Toxoplasma gondii in individuals with first-episode schizophrenia. Clinical infectious diseases : an official publication of the Infectious Diseases Society of America 32:842-844.

Zhao X, Tang R, Gao B, Shi Y, Zhou J, Guo S, Zhang J, Wang Y, Tang W, Meng J, Li S, Wang H, Ma G, Lin C, Xiao Y, Feng G, Lin Z, Zhu S, Xing Y, Sang H, St Clair D, He L (2007) Functional variants in the promoter region of Chitinase 3-like 1 (CHI3L1) and susceptibility to schizophrenia. American journal of human genetics 80:12-18.

Zipursky RB, Lim KO, Sullivan EV, Brown BW, Pfefferbaum A (1992) Widespread cerebral gray matter volume deficits in schizophrenia. Archives of general psychiatry 49:195-205.

**Alzheimer’s disease**

Alafuzoff I, Adolfsson R, Bucht G, Winblad B (1983) Albumin and immunoglobulin in plasma and cerebrospinal fluid, and blood-cerebrospinal fluid barrier function in patients with dementia of Alzheimer type and multi-infarct dementia. Journal of the neurological sciences 60:465-472.

Bagli M, Papassotiropoulos A, Knapp M, Jessen F, Luise Rao M, Maier W, Heun R (2000) Association between an interleukin-6 promoter and 3' flanking region haplotype and reduced Alzheimer's disease risk in a German population. Neuroscience letters 283:109-112.

Bullido MJ, Artiga MJ, Recuero M, Sastre I, Garcia MA, Aldudo J, Lendon C, Han SW, Morris JC, Frank A, Vazquez J, Goate A, Valdivieso F (1998) A polymorphism in the regulatory region of APOE associated with risk for Alzheimer's dementia. Nature genetics 18:69-71.

Clarke R, Smith AD, Jobst KA, Refsum H, Sutton L, Ueland PM (1998) Folate, vitamin B12, and serum total homocysteine levels in confirmed Alzheimer disease. Archives of neurology 55:1449-1455.

Crawford FC, Freeman MJ, Schinka JA, Abdullah LI, Gold M, Hartman R, Krivian K, Morris MD, Richards D, Duara R, Anand R, Mullan MJ (2000) A polymorphism in the cystatin C gene is a novel risk factor for late-onset Alzheimer's disease. Neurology 55:763-768.

Desai P, DeKosky ST, Kamboh MI (2002) Genetic variation in the cholesterol 24-hydroxylase (CYP46) gene and the risk of Alzheimer's disease. Neuroscience letters 328:9-12.

Foerstl H, Biedert S, Hewer W (1989) Multiinfarct and Alzheimer-type dementia investigated by transcranial Doppler sonography. Biological psychiatry 26:590-594.

Forette F, Seux ML, Staessen JA, Thijs L, Birkenhager WH, Babarskiene MR, Babeanu S, Bossini A, Gil-Extremera B, Girerd X, Laks T, Lilov E, Moisseyev V, Tuomilehto J, Vanhanen H, Webster J, Yodfat Y, Fagard R (1998) Prevention of dementia in randomised double-blind placebo-controlled Systolic Hypertension in Europe (Syst-Eur) trial. Lancet 352:1347-1351.

Grimaldi LM, Casadei VM, Ferri C, Veglia F, Licastro F, Annoni G, Biunno I, De Bellis G, Sorbi S, Mariani C, Canal N, Griffin WS, Franceschi M (2000) Association of early-onset Alzheimer's disease with an interleukin-1alpha gene polymorphism. Annals of neurology 47:361-365.

Grupe A, Abraham R, Li Y, Rowland C, Hollingworth P, Morgan A, Jehu L, Segurado R, Stone D, Schadt E, Karnoub M, Nowotny P, Tacey K, Catanese J, Sninsky J, Brayne C, Rubinsztein D, Gill M, Lawlor B, Lovestone S, Holmans P, O'Donovan M, Morris JC, Thal L, Goate A, Owen MJ, Williams J (2007) Evidence for novel susceptibility genes for late-onset Alzheimer's disease from a genome-wide association study of putative functional variants. Human molecular genetics 16:865-873.

Hamaguchi T, Okino S, Sodeyama N, Itoh Y, Takahashi A, Otomo E, Matsushita M, Mizusawa H, Yamada M (2005) Association of a polymorphism of the transforming growth factor-beta1 gene with cerebral amyloid angiopathy. Journal of neurology, neurosurgery, and psychiatry 76:696-699.

Heyman A, Wilkinson WE, Stafford JA, Helms MJ, Sigmon AH, Weinberg T (1984) Alzheimer's disease: a study of epidemiological aspects. Annals of neurology 15:335-341.

Katzov H, Chalmers K, Palmgren J, Andreasen N, Johansson B, Cairns NJ, Gatz M, Wilcock GK, Love S, Pedersen NL, Brookes AJ, Blennow K, Kehoe PG, Prince JA (2004) Genetic variants of ABCA1 modify Alzheimer disease risk and quantitative traits related to beta-amyloid metabolism. Human mutation 23:358-367.

Kunugi H, Ueki A, Otsuka M, Isse K, Hirasawa H, Kato N, Nabika T, Kobayashi S, Nanko S (2001) A novel polymorphism of the brain-derived neurotrophic factor (BDNF) gene associated with late-onset Alzheimer's disease. Molecular psychiatry 6:83-86.

Lambert JC, Bensemain F, Chapuis J, Cottel D, Amouyel P (2006) Association study of the PIN1 gene with Alzheimer's disease. Neuroscience letters 402:259-261.

Lambert JC, Berr C, Pasquier F, Delacourte A, Frigard B, Cottel D, Perez-Tur J, Mouroux V, Mohr M, Cecyre D, Galasko D, Lendon C, Poirier J, Hardy J, Mann D, Amouyel P, Chartier-Harlin MC (1998) Pronounced impact of Th1/E47cs mutation compared with -491 AT mutation on neural APOE gene expression and risk of developing Alzheimer's disease. Human molecular genetics 7:1511-1516.

Li Y, Grupe A, Rowland C, Holmans P, Segurado R, Abraham R, Jones L, Catanese J, Ross D, Mayo K, Martinez M, Hollingworth P, Goate A, Cairns NJ, Racette BA, Perlmutter JS, O'Donovan MC, Morris JC, Brayne C, Rubinsztein DC, Lovestone S, Thal LJ, Owen MJ, Williams J (2008) Evidence that common variation in NEDD9 is associated with susceptibility to late-onset Alzheimer's and Parkinson's disease. Human molecular genetics 17:759-767.

Lio D, Licastro F, Scola L, Chiappelli M, Grimaldi LM, Crivello A, Colonna-Romano G, Candore G, Franceschi C, Caruso C (2003) Interleukin-10 promoter polymorphism in sporadic Alzheimer's disease. Genes and immunity 4:234-238.

Luedecking EK, DeKosky ST, Mehdi H, Ganguli M, Kamboh MI (2000) Analysis of genetic polymorphisms in the transforming growth factor-beta1 gene and the risk of Alzheimer's disease. Human genetics 106:565-569.

Moalem S, Percy ME, Andrews DF, Kruck TP, Wong S, Dalton AJ, Mehta P, Fedor B, Warren AC (2000) Are hereditary hemochromatosis mutations involved in Alzheimer disease? American journal of medical genetics 93:58-66.

Mueller SG, Weiner MW, Thal LJ, Petersen RC, Jack CR, Jagust W, Trojanowski JQ, Toga AW, Beckett L (2005) Ways toward an early diagnosis in Alzheimer's disease: the Alzheimer's Disease Neuroimaging Initiative (ADNI). Alzheimer's & dementia : the journal of the Alzheimer's Association 1:55-66.

Muramatsu T, Matsushita S, Arai H, Sasaki H, Higuchi S (1996) Alpha 1-antichymotrypsin gene polymorphism and risk for Alzheimer's disease. Journal of neural transmission (Vienna, Austria : 1996) 103:1205-1210.

Murphy T, Yip A, Brayne C, Easton D, Evans JG, Xuereb J, Cairns N, Esiri MM, Rubinsztein DC (2001) The BACE gene: genomic structure and candidate gene study in late-onset Alzheimer's disease. Neuroreport 12:631-634.

Nishiyama M, Kato Y, Hashimoto M, Yukawa S, Omori K (2000) Apolipoprotein E, methylenetetrahydrofolate reductase (MTHFR) mutation and the risk of senile dementia--an epidemiological study using the polymerase chain reaction (PCR) method. Journal of epidemiology / Japan Epidemiological Association 10:163-172.

Papassotiropoulos A, Bagli M, Feder O, Jessen F, Maier W, Rao ML, Ludwig M, Schwab SG, Heun R (1999) Genetic polymorphism of cathepsin D is strongly associated with the risk for developing sporadic Alzheimer's disease. Neuroscience letters 262:171-174.

Reiman EM, Webster JA, Myers AJ, Hardy J, Dunckley T, Zismann VL, Joshipura KD, Pearson JV, Hu-Lince D, Huentelman MJ, Craig DW, Coon KD, Liang WS, Herbert RH, Beach T, Rohrer KC, Zhao AS, Leung D, Bryden L, Marlowe L, Kaleem M, Mastroeni D, Grover A, Heward CB, Ravid R, Rogers J, Hutton ML, Melquist S, Petersen RC, Alexander GE, Caselli RJ, Kukull W, Papassotiropoulos A, Stephan DA (2007) GAB2 alleles modify Alzheimer's risk in APOE epsilon4 carriers. Neuron 54:713-720.

Rezek DL (1987) Olfactory deficits as a neurologic sign in dementia of the Alzheimer type. Archives of neurology 44:1030-1032.

Rogaeva E, Meng Y, Lee JH, Gu Y, Kawarai T, Zou F, Katayama T, Baldwin CT, Cheng R, Hasegawa H, Chen F, Shibata N, Lunetta KL, Pardossi-Piquard R, Bohm C, Wakutani Y, Cupples LA, Cuenco KT, Green RC, Pinessi L, Rainero I, Sorbi S, Bruni A, Duara R, Friedland RP, Inzelberg R, Hampe W, Bujo H, Song YQ, Andersen OM, Willnow TE, Graff-Radford N, Petersen RC, Dickson D, Der SD, Fraser PE, Schmitt-Ulms G, Younkin S, Mayeux R, Farrer LA, St George-Hyslop P (2007) The neuronal sortilin-related receptor SORL1 is genetically associated with Alzheimer disease. Nature genetics 39:168-177.

Salmon DP, Shimamura AP, Butters N, Smith S (1988) Lexical and semantic priming deficits in patients with Alzheimer's disease. Journal of clinical and experimental neuropsychology 10:477-494.

Schmidt R, Schmidt H, Curb JD, Masaki K, White LR, Launer LJ (2002) Early inflammation and dementia: a 25-year follow-up of the Honolulu-Asia Aging Study. Annals of neurology 52:168-174.

Shore D, Henkin RI, Nelson NR, Agarwal RP, Wyatt RJ (1984) Hair and serum copper, zinc, calcium, and magnesium concentrations in Alzheimer-type dementia. Journal of the American Geriatrics Society 32:892-895.

Strittmatter WJ, Saunders AM, Schmechel D, Pericak-Vance M, Enghild J, Salvesen GS, Roses AD (1993) Apolipoprotein E: high-avidity binding to beta-amyloid and increased frequency of type 4 allele in late-onset familial Alzheimer disease. Proceedings of the National Academy of Sciences of the United States of America 90:1977-1981.

Talbot C, Houlden H, Craddock N, Crook R, Hutton M, Lendon C, Prihar G, Morris JC, Hardy J, Goate A (1996) Polymorphism in AACT gene may lower age of onset of Alzheimer's disease. Neuroreport 7:534-536.

Tamaoka A, Fukushima T, Sawamura N, Ishikawa K, Oguni E, Komatsuzaki Y, Shoji S (1996) Amyloid beta protein in plasma from patients with sporadic Alzheimer's disease. Journal of the neurological sciences 141:65-68.

Tarkowski E, Liljeroth AM, Nilsson A, Ricksten A, Davidsson P, Minthon L, Blennow K (2000) TNF gene polymorphism and its relation to intracerebral production of TNFalpha and TNFbeta in AD. Neurology 54:2077-2081.

Ventriglia M, Bocchio Chiavetto L, Benussi L, Binetti G, Zanetti O, Riva MA, Gennarelli M (2002) Association between the BDNF 196 A/G polymorphism and sporadic Alzheimer's disease. Molecular psychiatry 7:136-137.

Ward NI, Mason JA (1987) Neutron activation analysis techniques for identifying elemental status in Alzheimer's disease Journal of radioanalytical and nuclear chemistry 113:515-526.

Warner MD, Peabody CA, Flattery JJ, Tinklenberg JR (1986) Olfactory deficits and Alzheimer's disease. Biological psychiatry 21:116-118.

Wollmer MA, Streffer JR, Lutjohann D, Tsolaki M, Iakovidou V, Hegi T, Pasch T, Jung HH, Bergmann K, Nitsch RM, Hock C, Papassotiropoulos A (2003) ABCA1 modulates CSF cholesterol levels and influences the age at onset of Alzheimer's disease. Neurobiology of aging 24:421-426.

Zuliani G, Ble A, Zanca R, Munari MR, Zurlo A, Vavalle C, Atti AR, Fellin R (2001) Genetic polymorphisms in older subjects with vascular or Alzheimer's dementia. Acta neurologica Scandinavica 103:304-308.

**Epilepsy**

Bartak L, Rutter M (1976) Differences between mentally retarded and normally intelligent autistic children. Journal of autism and childhood schizophrenia 6:109-120.

Briellmann RS, Torn-Broers Y, Busuttil BE, Major BJ, Kalnins RM, Olsen M, Jackson GD, Frauman AG, Berkovic SF (2000) APOE epsilon4 genotype is associated with an earlier onset of chronic temporal lobe epilepsy. Neurology 55:435-437.

Glickman LT, Cypess RH, Crumrine PK, Gitlin DA (1979) Toxocara infection and epilepsy in children. The Journal of pediatrics 94:75-78.

Kananura C, Haug K, Sander T, Runge U, Gu W, Hallmann K, Rebstock J, Heils A, Steinlein OK (2002) A splice-site mutation in GABRG2 associated with childhood absence epilepsy and febrile convulsions. Archives of neurology 59:1137-1141.

Kanemoto K, Kawasaki J, Miyamoto T, Obayashi H, Nishimura M (2000) Interleukin (IL)1beta, IL-1alpha, and IL-1 receptor antagonist gene polymorphisms in patients with temporal lobe epilepsy. Annals of neurology 47:571-574.

Massetani R, Strata G, Galli R, Gori S, Gneri C, Limbruno U, Di Santo D, Mariani M, Murri L (1997) Alteration of cardiac function in patients with temporal lobe epilepsy: different roles of EEG-ECG monitoring and spectral analysis of RR variability. Epilepsia 38:363-369.

Mignard C, Mignard D, Dandelot JB, Polydor JP, Laporte JP, Bousquet C, Choucair Y, Michault A (1986) [Epidemiologic survey of the cysticercosis endemic on Reunion Island]. Revue neurologique 142:635-637.

Ng SK, Hauser WA, Brust JC, Susser M (1988) Alcohol consumption and withdrawal in new-onset seizures. The New England journal of medicine 319:666-673.

Ogunniyi A, Osuntokun BO, Bademosi O, Adeuja AO, Schoenberg BS (1987) Risk factors for epilepsy: case-control study in Nigerians. Epilepsia 28:280-285.

Peltola J, Hurme M, Miettinen A, Keranen T (1998) Elevated levels of interleukin-6 may occur in cerebrospinal fluid from patients with recent epileptic seizures. Epilepsy research 31:129-133.

Reutens DC, Berkovic SF (1992) Increased cortical excitability in generalised epilepsy demonstrated with transcranial magnetic stimulation. Lancet 339:362-363.

Rodrigo S, Oppenheim C, Chassoux F, Golestani N, Cointepas Y, Poupon C, Semah F, Mangin JF, Le Bihan D, Meder JF (2007) Uncinate fasciculus fiber tracking in mesial temporal lobe epilepsy. Initial findings. European radiology 17:1663-1668.

Siddiqui A, Kerb R, Weale ME, Brinkmann U, Smith A, Goldstein DB, Wood NW, Sisodiya SM (2003) Association of multidrug resistance in epilepsy with a polymorphism in the drug-transporter gene ABCB1. The New England journal of medicine 348:1442-1448.

Tsai L, Stewart MA, August G (1981) Implication of sex differences in the familial transmission of infantile autism. Journal of autism and developmental disorders 11:165-173.

**Multiple sclerosis**

Alter M, Speer J (1968) Clinical evaluation of possible etiologic factors in multiple sclerosis. Neurology 18:109-116.

Amaducci L, Arfaioli C, Inzitari D, Marchi M (1982) Multiple sclerosis among shoe and leather workers: an epidemiological survey in Florence. Acta neurologica Scandinavica 65:94-103.

Antonovsky A, Leibowitz U, Smith HA, Medalie JM, Balogh M, Kats R, Halpern L, Alter M (1965) EPIDEMIOLOGIC STUDY OF MULTIPLE SCLEROSIS IN ISRAEL. I. AN OVERALL REVIEW OF METHODS AND FINDINGS. Archives of neurology 13:183-193.

Aulchenko YS, Hoppenbrouwers IA, Ramagopalan SV, Broer L, Jafari N, Hillert J, Link J, Lundstrom W, Greiner E, Dessa Sadovnick A, Goossens D, Van Broeckhoven C, Del-Favero J, Ebers GC, Oostra BA, van Duijn CM, Hintzen RQ (2008) Genetic variation in the KIF1B locus influences susceptibility to multiple sclerosis. Nature genetics 40:1402-1403.

Berr C, Puel J, Clanet M, Ruidavets JB, Mas JL, Alperovitch A (1989) Risk factors in multiple sclerosis: a population-based case-control study in Hautes-Pyrenees, France. Acta neurologica Scandinavica 80:46-50.

Besler HT, Comoglu S (2003) Lipoprotein oxidation, plasma total antioxidant capacity and homocysteine level in patients with multiple sclerosis. Nutritional neuroscience 6:189-196.

Bray PF, Luka J, Bray PF, Culp KW, Schlight JP (1992) Antibodies against Epstein-Barr nuclear antigen (EBNA) in multiple sclerosis CSF, and two pentapeptide sequence identities between EBNA and myelin basic protein. Neurology 42:1798-1804.

Coraddu F, Reyes-Yanez MP, Parra A, Gray J, Smith SI, Taylor CJ, Compston DA (1998) HLA associations with multiple sclerosis in the Canary Islands. Journal of neuroimmunology 87:130-135.

Crusius JB, Pena AS, Van Oosten BW, Bioque G, Garcia A, Dijkstra CD, Polman CH (1995) Interleukin-1 receptor antagonist gene polymorphism and multiple sclerosis. Lancet 346:979.

Ferri C, Sciacca FL, Grimaldi LE, Veglia F, Magnani G, Santuccio G, Comi G, Canal N, Grimaldi LM (2000) Lack of association between IL-1A and IL-1B promoter polymorphisms and multiple sclerosis. Journal of neurology, neurosurgery, and psychiatry 69:564-565.

Fugger L, Morling N, Sandberg-Wollheim M, Ryder LP, Svejgaard A (1990) Tumor necrosis factor alpha gene polymorphism in multiple sclerosis and optic neuritis. Journal of neuroimmunology 27:85-88.

Ghabanbasani MZ, Gu XX, Spaepen M, Vandevyver C, Raus J, Marynen P, Carton H, Cassiman JJ (1995) Importance of HLA-DRB1 and DQA1 genes and of the amino acid polymorphisms in the functional domain of DR beta 1 chain in multiple sclerosis. Journal of neuroimmunology 59:77-82.

Hafler DA, Compston A, Sawcer S, Lander ES, Daly MJ, De Jager PL, de Bakker PI, Gabriel SB, Mirel DB, Ivinson AJ, Pericak-Vance MA, Gregory SG, Rioux JD, McCauley JL, Haines JL, Barcellos LF, Cree B, Oksenberg JR, Hauser SL (2007) Risk alleles for multiple sclerosis identified by a genomewide study. The New England journal of medicine 357:851-862.

Huizinga TW, Westendorp RG, Bollen EL, Keijsers V, Brinkman BM, Langermans JA, Breedveld FC, Verweij CL, van de Gaer L, Dams L, Crusius JB, Garcia-Gonzalez A, van Oosten BW, Polman CH, Pena AS (1997) TNF-alpha promoter polymorphisms, production and susceptibility to multiple sclerosis in different groups of patients. Journal of neuroimmunology 72:149-153.

Neukirch F, Lyon-Caen O, Clanet M, Bousquet J, Feingold J, Druet P (1997) Asthma, nasal allergies, and multiple sclerosis. The Journal of allergy and clinical immunology 99:270-271.

Nijst TQ, Wevers RA, Schoonderwaldt HC, Hommes OR, de Haan AF (1990) Vitamin B12 and folate concentrations in serum and cerebrospinal fluid of neurological patients with special reference to multiple sclerosis and dementia. Journal of neurology, neurosurgery, and psychiatry 53:951-954.

Operskalski EA, Visscher BR, Malmgren RM, Detels R (1989) A case-control study of multiple sclerosis. Neurology 39:825-829.

Oro AS, Guarino TJ, Driver R, Steinman L, Umetsu DT (1996) Regulation of disease susceptibility: decreased prevalence of IgE-mediated allergic disease in patients with multiple sclerosis. The Journal of allergy and clinical immunology 97:1402-1408.

Partridge JM, Weatherby SJ, Woolmore JA, Highland DJ, Fryer AA, Mann CL, Boggild MD, Ollier WE, Strange RC, Hawkins CP (2004) Susceptibility and outcome in MS: associations with polymorphisms in pigmentation-related genes. Neurology 62:2323-2325.

Pei J, Xu WZ, Sun SX, Mi XY, Hou ZC, Wang LH (1987) HLA and multiple sclerosis in Chinese. Proceedings of the Chinese Academy of Medical Sciences and the Peking Union Medical College = Chung-kuo i hsueh k'o hsueh yuan, Chung-kuo hsieh ho i k'o ta hsueh hsueh pao 2:111-115.

Pickard C, Mann C, Sinnott P, Boggild M, Hawkins C, Strange RC, Hutchinson IV, Ollier WE, Donn RP (1999) Interleukin-10 (IL10) promoter polymorphisms and multiple sclerosis. Journal of neuroimmunology 101:207-210.

Rubinsztein DC, Hanlon CS, Irving RM, Goodburn S, Evans DG, Kellar-Wood H, Xuereb JH, Bandmann O, Harding AE (1994) Apo E genotypes in multiple sclerosis, Parkinson's disease, schwannomas and late-onset Alzheimer's disease. Molecular and cellular probes 8:519-525.

Sadovnick AD, Baird PA (1988) The familial nature of multiple sclerosis: age-corrected empiric recurrence risks for children and siblings of patients. Neurology 38:990-991.

Sanders VJ, Felisan S, Waddell A, Tourtellotte WW (1996) Detection of herpesviridae in postmortem multiple sclerosis brain tissue and controls by polymerase chain reaction. Journal of neurovirology 2:249-258.

Schrijver HM, Crusius JB, Uitdehaag BM, Garcia Gonzalez MA, Kostense PJ, Polman CH, Pena AS (1999) Association of interleukin-1beta and interleukin-1 receptor antagonist genes with disease severity in MS. Neurology 52:595-599.

Sumaya CV, Myers L, Ellison GW (1976) Epstein-Barr virus antibodies in multiple sclerosis. Transactions of the American Neurological Association 101:300-302.

Watkins SM, Espir M (1969) Migraine and multiple sclerosis. Journal of neurology, neurosurgery, and psychiatry 32:35-37.

Wikström J, Kinnunen E, Porras J (1984) The age-specific prevalence ratio of familial multiple sclerosis. Neuroepidemiology 3:74-81.

Zilber N, Kahana E (1996) Risk factors for multiple sclerosis: a case-control study in Israel. Acta neurologica Scandinavica 94:395-403.

Zipp F, Weil JG, Einhaupl KM (1999) No increase in demyelinating diseases after hepatitis B vaccination. Nature medicine 5:964-965.

**Parkinson’s Disease**

Butterfield PG, Valanis BG, Spencer PS, Lindeman CA, Nutt JG (1993) Environmental antecedents of young-onset Parkinson's disease. Neurology 43:1150-1158.

Cabrera-Valdivia F, Jimenez-Jimenez FJ, Molina JA, Fernandez-Calle P, Vazquez A, Canizares-Liebana F, Larumbe-Lobalde S, Ayuso-Peralta L, Rabasa M, Codoceo R (1994) Peripheral iron metabolism in patients with Parkinson's disease. Journal of the neurological sciences 125:82-86.

Chen H, Zhang SM, Hernan MA, Schwarzschild MA, Willett WC, Colditz GA, Speizer FE, Ascherio A (2003) Nonsteroidal anti-inflammatory drugs and the risk of Parkinson disease. Archives of neurology 60:1059-1064.

Di Fonzo A, Wu-Chou YH, Lu CS, van Doeselaar M, Simons EJ, Rohe CF, Chang HC, Chen RS, Weng YH, Vanacore N, Breedveld GJ, Oostra BA, Bonifati V (2006) A common missense variant in the LRRK2 gene, Gly2385Arg, associated with Parkinson's disease risk in Taiwan. Neurogenetics 7:133-138.

Fuchs J, Mueller JC, Lichtner P, Schulte C, Munz M, Berg D, Wullner U, Illig T, Sharma M, Gasser T (2009) The transcription factor PITX3 is associated with sporadic Parkinson's disease. Neurobiology of aging 30:731-738.

Haack DG, Baumann RJ, McKean HE, Jameson HD, Turbek JA (1981) Nicotine exposure and Parkinson disease. American journal of epidemiology 114:191-200.

Haubenberger D, Hotzy C, Pirker W, Katzenschlager R, Brucke T, Zimprich F, Auff E, Zimprich A (2009) Role of LINGO1 polymorphisms in Parkinson's disease. Movement disorders : official journal of the Movement Disorder Society 24:2404-2407.

Ho SC, Woo J, Lee CM (1989) Epidemiologic study of Parkinson's disease in Hong Kong. Neurology 39:1314-1318.

Hoda F, Nicholl D, Bennett P, Arranz M, Aitchison KJ, al-Chalabi A, Kunugi H, Vallada H, Leigh PN, Chaudhuri KR, Collier DA (1996) No association between Parkinson's disease and low-activity alleles of catechol O-methyltransferase. Biochemical and biophysical research communications 228:780-784.

Huang X, Chen H, Miller WC, Mailman RB, Woodard JL, Chen PC, Xiang D, Murrow RW, Wang YZ, Poole C (2007) Lower low-density lipoprotein cholesterol levels are associated with Parkinson's disease. Movement disorders : official journal of the Movement Disorder Society 22:377-381.

Hubble JP, Cao T, Hassanein RE, Neuberger JS, Koller WC (1993) Risk factors for Parkinson's disease. Neurology 43:1693-1697.

Jimenez-Jimenez FJ, Fernandez-Calle P, Martinez-Vanaclocha M, Herrero E, Molina JA, Vazquez A, Codoceo R (1992) Serum levels of zinc and copper in patients with Parkinson's disease. Journal of the neurological sciences 112:30-33.

Kessler, II (1972) Epidemiologic studies of Parkinson's disease. 3. A community-based survey. American journal of epidemiology 96:242-254.

Kondo I, Yamamoto M (1998) Genetic polymorphism of paraoxonase 1 (PON1) and susceptibility to Parkinson's disease. Brain research 806:271-273.

Lang AE, Kierans C, Blair RD (1987) Family history of tremor in Parkinson's disease compared with those of controls and patients with idiopathic dystonia. Advances in neurology 45:313-316.

Lwin A, Orvisky E, Goker-Alpan O, LaMarca ME, Sidransky E (2004) Glucocerebrosidase mutations in subjects with parkinsonism. Molecular genetics and metabolism 81:70-73.

Maraganore DM, de Andrade M, Lesnick TG, Strain KJ, Farrer MJ, Rocca WA, Pant PV, Frazer KA, Cox DR, Ballinger DG (2005) High-resolution whole-genome association study of Parkinson disease. American journal of human genetics 77:685-693.

Maraganore DM, Farrer MJ, Hardy JA, Lincoln SJ, McDonnell SK, Rocca WA (1999) Case-control study of the ubiquitin carboxy-terminal hydrolase L1 gene in Parkinson's disease. Neurology 53:1858-1860.

Maraganore DM, Hernandez DG, Singleton AB, Farrer MJ, McDonnell SK, Hutton ML, Hardy JA, Rocca WA (2001) Case-Control study of the extended tau gene haplotype in Parkinson's disease. Annals of neurology 50:658-661.

Marder K, Maestre G, Cote L, Mejia H, Alfaro B, Halim A, Tang M, Tycko B, Mayeux R (1994) The apolipoprotein epsilon 4 allele in Parkinson's disease with and without dementia. Neurology 44:1330-1331.

Marder K, Tang MX, Alfaro B, Mejia H, Cote L, Jacobs D, Stern Y, Sano M, Mayeux R (1998) Postmenopausal estrogen use and Parkinson's disease with and without dementia. Neurology 50:1141-1143.

Martin WE, Young WI, Anderson VE (1973) Parkinson's disease. A genetic study. Brain : a journal of neurology 96:495-506.

Mueller JC, Fuchs J, Hofer A, Zimprich A, Lichtner P, Illig T, Berg D, Wullner U, Meitinger T, Gasser T (2005) Multiple regions of alpha-synuclein are associated with Parkinson's disease. Annals of neurology 57:535-541.

Nefzger MD, Quadfasel FA, Karl VC (1968) A retrospective study of smoking in Parkinson's disease. American journal of epidemiology 88:149-158.

Nichols WC, Pankratz N, Hernandez D, Paisan-Ruiz C, Jain S, Halter CA, Michaels VE, Reed T, Rudolph A, Shults CW, Singleton A, Foroud T (2005) Genetic screening for a single common LRRK2 mutation in familial Parkinson's disease. Lancet 365:410-412.

Nishimura M, Mizuta I, Mizuta E, Yamasaki S, Ohta M, Kuno S (2000) Influence of interleukin-1beta gene polymorphisms on age-at-onset of sporadic Parkinson's disease. Neuroscience letters 284:73-76.

Oliveri RL, Zappia M, Annesi G, Bosco D, Annesi F, Spadafora P, Pasqua AA, Tomaino C, Nicoletti G, Pirritano D, Labate A, Gambardella A, Logroscino G, Manobianca G, Epifanio A, Morgante L, Savettieri G, Quattrone A (2001) The parkin gene is not involved in late-onset Parkinson's disease. Neurology 57:359-362.

Pankratz N, Wilk JB, Latourelle JC, DeStefano AL, Halter C, Pugh EW, Doheny KF, Gusella JF, Nichols WC, Foroud T, Myers RH (2009) Genomewide association study for susceptibility genes contributing to familial Parkinson disease. Human genetics 124:593-605.

Reynolds LM, Locke S (1971) Relation between handedness and side of onset of Parkinsonism. Lancet 2:714.

Ross OA, Wu YR, Lee MC, Funayama M, Chen ML, Soto AI, Mata IF, Lee-Chen GJ, Chen CM, Tang M, Zhao Y, Hattori N, Farrer MJ, Tan EK, Wu RM (2008) Analysis of Lrrk2 R1628P as a risk factor for Parkinson's disease. Annals of neurology 64:88-92.

Satoh J, Kuroda Y (1999) Association of codon 167 Ser/Asn heterozygosity in the parkin gene with sporadic Parkinson's disease. Neuroreport 10:2735-2739.

Scott S, Caird FI, Williams BO (1984) Evidence for an apparent sensory speech disorder in Parkinson's disease. Journal of neurology, neurosurgery, and psychiatry 47:840-843.

Semchuk KM, Love EJ, Lee RG (1992) Parkinson's disease and exposure to agricultural work and pesticide chemicals. Neurology 42:1328-1335.

Semchuk KM, Love EJ, Lee RG (1993) Parkinson's disease: a test of the multifactorial etiologic hypothesis. Neurology 43:1173-1180.

Taanman JW, Schapira AH (2005) Analysis of the trinucleotide CAG repeat from the DNA polymerase gamma gene (POLG) in patients with Parkinson's disease. Neuroscience letters 376:56-59.

Tanner CM, Chen B, Wang W, Peng M, Liu Z, Liang X, Kao LC, Gilley DW, Goetz CG, Schoenberg BS (1989) Environmental factors and Parkinson's disease: a case-control study in China. Neurology 39:660-664.

Wang J, Liu Z (2000) No association between paraoxonase 1 (PON1) gene polymorphisms and susceptibility to Parkinson's disease in a Chinese population. Movement disorders : official journal of the Movement Disorder Society 15:1265-1267.

Ward CD, Hess WA, Calne DB (1983) Olfactory impairment in Parkinson's disease. Neurology 33:943-946.

**Breast cancer**

Ahmed S, Thomas G, Ghoussaini M, Healey CS, Humphreys MK, Platte R, Morrison J, Maranian M, Pooley KA, Luben R, Eccles D, Evans DG, Fletcher O, Johnson N, dos Santos Silva I, Peto J, Stratton MR, Rahman N, Jacobs K, Prentice R, Anderson GL, Rajkovic A, Curb JD, Ziegler RG, Berg CD, Buys SS, McCarty CA, Feigelson HS, Calle EE, Thun MJ, Diver WR, Bojesen S, Nordestgaard BG, Flyger H, Dork T, Schurmann P, Hillemanns P, Karstens JH, Bogdanova NV, Antonenkova NN, Zalutsky IV, Bermisheva M, Fedorova S, Khusnutdinova E, Kang D, Yoo KY, Noh DY, Ahn SH, Devilee P, van Asperen CJ, Tollenaar RA, Seynaeve C, Garcia-Closas M, Lissowska J, Brinton L, Peplonska B, Nevanlinna H, Heikkinen T, Aittomaki K, Blomqvist C, Hopper JL, Southey MC, Smith L, Spurdle AB, Schmidt MK, Broeks A, van Hien RR, Cornelissen S, Milne RL, Ribas G, Gonzalez-Neira A, Benitez J, Schmutzler RK, Burwinkel B, Bartram CR, Meindl A, Brauch H, Justenhoven C, Hamann U, Chang-Claude J, Hein R, Wang-Gohrke S, Lindblom A, Margolin S, Mannermaa A, Kosma VM, Kataja V, Olson JE, Wang X, Fredericksen Z, Giles GG, Severi G, Baglietto L, English DR, Hankinson SE, Cox DG, Kraft P, Vatten LJ, Hveem K, Kumle M, Sigurdson A, Doody M, Bhatti P, Alexander BH, Hooning MJ, van den Ouweland AM, Oldenburg RA, Schutte M, Hall P, Czene K, Liu J, Li Y, Cox A, Elliott G, Brock I, Reed MW, Shen CY, Yu JC, Hsu GC, Chen ST, Anton-Culver H, Ziogas A, Andrulis IL, Knight JA, Beesley J, Goode EL, Couch F, Chenevix-Trench G, Hoover RN, Ponder BA, Hunter DJ, Pharoah PD, Dunning AM, Chanock SJ, Easton DF (2009) Newly discovered breast cancer susceptibility loci on 3p24 and 17q23.2. Nature genetics 41:585-590.

Albanes D, Blair A, Taylor PR (1989) Physical activity and risk of cancer in the NHANES I population. American journal of public health 79:744-750.

Allinen M, Huusko P, Mantyniemi S, Launonen V, Winqvist R (2001) Mutation analysis of the CHK2 gene in families with hereditary breast cancer. British journal of cancer 85:209-212.

Ambrosone CB, Freudenheim JL, Graham S, Marshall JR, Vena JE, Brasure JR, Michalek AM, Laughlin R, Nemoto T, Gillenwater KA, Shields PG (1996) Cigarette smoking, N-acetyltransferase 2 genetic polymorphisms, and breast cancer risk. JAMA : the journal of the American Medical Association 276:1494-1501.

Ambrosone CB, Freudenheim JL, Thompson PA, Bowman E, Vena JE, Marshall JR, Graham S, Laughlin R, Nemoto T, Shields PG (1999) Manganese superoxide dismutase (MnSOD) genetic polymorphisms, dietary antioxidants, and risk of breast cancer. Cancer research 59:602-606.

Angele S, Romestaing P, Moullan N, Vuillaume M, Chapot B, Friesen M, Jongmans W, Cox DG, Pisani P, Gerard JP, Hall J (2003) ATM haplotypes and cellular response to DNA damage: association with breast cancer risk and clinical radiosensitivity. Cancer research 63:8717-8725.

Augustin LS, Dal Maso L, La Vecchia C, Parpinel M, Negri E, Vaccarella S, Kendall CW, Jenkins DJ, Francesch S (2001) Dietary glycemic index and glycemic load, and breast cancer risk: a case-control study. Annals of oncology : official journal of the European Society for Medical Oncology / ESMO 12:1533-1538.

Beck P, Wysowski DK, Downey W, Butler-Jones D (2003) Statin use and the risk of breast cancer. Journal of clinical epidemiology 56:280-285.

Bulovskaya LN, Krupkin RG, Bochina TA, Shipkova AA, Pavlova MV (1978) Acetylator phenotype in patients with breast cancer. Oncology 35:185-188.

Carlomagno F, Chang-Claude J, Dunning AM, Ponder BA (1999) Determination of the frequency of the common 657Del5 Nijmegen breakage syndrome mutation in the German population: no association with risk of breast cancer. Genes, chromosomes & cancer 25:393-395.

Chouchane L, Ahmed SB, Baccouche S, Remadi S (1997) Polymorphism in the tumor necrosis factor-alpha promotor region and in the heat shock protein 70 genes associated with malignant tumors. Cancer 80:1489-1496.

Curran JE, Vaughan T, Lea RA, Weinstein SR, Morrison NA, Griffiths LR (1999) Association of A vitamin D receptor polymorphism with sporadic breast cancer development. International journal of cancer Journal international du cancer 83:723-726.

De Vivo I, Hankinson SE, Colditz GA, Hunter DJ (2003) A functional polymorphism in the progesterone receptor gene is associated with an increase in breast cancer risk. Cancer research 63:5236-5238.

Dorgan JF, Sowell A, Swanson CA, Potischman N, Miller R, Schussler N, Stephenson HE, Jr. (1998) Relationships of serum carotenoids, retinol, alpha-tocopherol, and selenium with breast cancer risk: results from a prospective study in Columbia, Missouri (United States). Cancer causes & control : CCC 9:89-97.

Duell EJ, Millikan RC, Pittman GS, Winkel S, Lunn RM, Tse CK, Eaton A, Mohrenweiser HW, Newman B, Bell DA (2001) Polymorphisms in the DNA repair gene XRCC1 and breast cancer. Cancer epidemiology, biomarkers & prevention : a publication of the American Association for Cancer Research, cosponsored by the American Society of Preventive Oncology 10:217-222.

Dunning AM, Ellis PD, McBride S, Kirschenlohr HL, Healey CS, Kemp PR, Luben RN, Chang-Claude J, Mannermaa A, Kataja V, Pharoah PD, Easton DF, Ponder BA, Metcalfe JC (2003) A transforming growth factorbeta1 signal peptide variant increases secretion in vitro and is associated with increased incidence of invasive breast cancer. Cancer research 63:2610-2615.

Easton DF, Pooley KA, Dunning AM, Pharoah PD, Thompson D, Ballinger DG, Struewing JP, Morrison J, Field H, Luben R, Wareham N, Ahmed S, Healey CS, Bowman R, Meyer KB, Haiman CA, Kolonel LK, Henderson BE, Le Marchand L, Brennan P, Sangrajrang S, Gaborieau V, Odefrey F, Shen CY, Wu PE, Wang HC, Eccles D, Evans DG, Peto J, Fletcher O, Johnson N, Seal S, Stratton MR, Rahman N, Chenevix-Trench G, Bojesen SE, Nordestgaard BG, Axelsson CK, Garcia-Closas M, Brinton L, Chanock S, Lissowska J, Peplonska B, Nevanlinna H, Fagerholm R, Eerola H, Kang D, Yoo KY, Noh DY, Ahn SH, Hunter DJ, Hankinson SE, Cox DG, Hall P, Wedren S, Liu J, Low YL, Bogdanova N, Schurmann P, Dork T, Tollenaar RA, Jacobi CE, Devilee P, Klijn JG, Sigurdson AJ, Doody MM, Alexander BH, Zhang J, Cox A, Brock IW, MacPherson G, Reed MW, Couch FJ, Goode EL, Olson JE, Meijers-Heijboer H, van den Ouweland A, Uitterlinden A, Rivadeneira F, Milne RL, Ribas G, Gonzalez-Neira A, Benitez J, Hopper JL, McCredie M, Southey M, Giles GG, Schroen C, Justenhoven C, Brauch H, Hamann U, Ko YD, Spurdle AB, Beesley J, Chen X, Mannermaa A, Kosma VM, Kataja V, Hartikainen J, Day NE, Cox DR, Ponder BA (2007) Genome-wide association study identifies novel breast cancer susceptibility loci. Nature 447:1087-1093.

Feigelson HS, Coetzee GA, Kolonel LN, Ross RK, Henderson BE (1997) A polymorphism in the CYP17 gene increases the risk of breast cancer. Cancer research 57:1063-1065.

Forsti A, Angelini S, Festa F, Sanyal S, Zhang Z, Grzybowska E, Pamula J, Pekala W, Zientek H, Hemminki K, Kumar R (2004) Single nucleotide polymorphisms in breast cancer. Oncology reports 11:917-922.

Freudenheim JL, Ambrosone CB, Moysich KB, Vena JE, Graham S, Marshall JR, Muti P, Laughlin R, Nemoto T, Harty LC, Crits GA, Chan AW, Shields PG (1999) Alcohol dehydrogenase 3 genotype modification of the association of alcohol consumption with breast cancer risk. Cancer causes & control : CCC 10:369-377.

Freudenheim JL, Marshall JR, Vena JE, Laughlin R, Brasure JR, Swanson MK, Nemoto T, Graham S (1996) Premenopausal breast cancer risk and intake of vegetables, fruits, and related nutrients. Journal of the National Cancer Institute 88:340-348.

Friedman GD, Ury HK (1980) Initial screening for carcinogenicity of commonly used drugs. Journal of the National Cancer Institute 65:723-733.

Ghilardi G, Biondi ML, Cecchini F, DeMonti M, Guagnellini E, Scorza R (2003) Vascular invasion in human breast cancer is correlated to T-->786C polymorphism of NOS3 gene. Nitric oxide : biology and chemistry / official journal of the Nitric Oxide Society 9:118-122.

Graham S, Marshall J, Mettlin C, Rzepka T, Nemoto T, Byers T (1982) Diet in the epidemiology of breast cancer. American journal of epidemiology 116:68-75.

Gridley G, McLaughlin JK, Ekbom A, Klareskog L, Adami HO, Hacker DG, Hoover R, Fraumeni JF, Jr. (1993) Incidence of cancer among patients with rheumatoid arthritis. Journal of the National Cancer Institute 85:307-311.

Grieu F, Malaney S, Ward R, Joseph D, Iacopetta B (2003) Lack of association between CCND1 G870A polymorphism and the risk of breast and colorectal cancers. Anticancer research 23:4257-4259.

Harries LW, Stubbins MJ, Forman D, Howard GC, Wolf CR (1997) Identification of genetic polymorphisms at the glutathione S-transferase Pi locus and association with susceptibility to bladder, testicular and prostate cancer. Carcinogenesis 18:641-644.

Hou MF, Huang TJ, Liu GC (2001) The diagnostic value of galactography in patients with nipple discharge. Clinical imaging 25:75-81.

Hunter DJ, Kraft P, Jacobs KB, Cox DG, Yeager M, Hankinson SE, Wacholder S, Wang Z, Welch R, Hutchinson A, Wang J, Yu K, Chatterjee N, Orr N, Willett WC, Colditz GA, Ziegler RG, Berg CD, Buys SS, McCarty CA, Feigelson HS, Calle EE, Thun MJ, Hayes RB, Tucker M, Gerhard DS, Fraumeni JF, Jr., Hoover RN, Thomas G, Chanock SJ (2007) A genome-wide association study identifies alleles in FGFR2 associated with risk of sporadic postmenopausal breast cancer. Nature genetics 39:870-874.

Ingram D, Sanders K, Kolybaba M, Lopez D (1997) Case-control study of phyto-oestrogens and breast cancer. Lancet 350:990-994.

Iwase H, Greenman JM, Barnes DM, Hodgson S, Bobrow L, Mathew CG (1996) Sequence variants of the estrogen receptor (ER) gene found in breast cancer patients with ER negative and progesterone receptor positive tumors. Cancer letters 108:179-184.

Jick H, Walker AM, Watkins RN, D'Ewart DC, Hunter JR, Danford A, Madsen S, Dinan BJ, Rothman KJ (1980) Replacement estrogens and breast cancer. American journal of epidemiology 112:586-594.

Justenhoven C, Hamann U, Pierl CB, Rabstein S, Pesch B, Harth V, Baisch C, Vollmert C, Illig T, Bruning T, Ko Y, Brauch H (2005) One-carbon metabolism and breast cancer risk: no association of MTHFR, MTR, and TYMS polymorphisms in the GENICA study from Germany. Cancer epidemiology, biomarkers & prevention : a publication of the American Association for Cancer Research, cosponsored by the American Society of Preventive Oncology 14:3015-3018.

Kapdi CC, Wolfe JN (1976) Breast cancer. Relationship to thyroid supplements for hypothyroidism. JAMA : the journal of the American Medical Association 236:1124-1127.

Katsouyanni K, Willett W, Trichopoulos D, Boyle P, Trichopoulou A, Vasilaros S, Papadiamantis J, MacMahon B (1988) Risk of breast cancer among Greek women in relation to nutrient intake. Cancer 61:181-185.

Kawajiri K, Nakachi K, Imai K, Watanabe J, Hayashi S (1993) Germ line polymorphisms of p53 and CYP1A1 genes involved in human lung cancer. Carcinogenesis 14:1085-1089.

Koh WP, Yuan JM, Sun CL, van den Berg D, Seow A, Lee HP, Yu MC (2003) Angiotensin I-converting enzyme (ACE) gene polymorphism and breast cancer risk among Chinese women in Singapore. Cancer research 63:573-578.

Krippl P, Langsenlehner U, Renner W, Yazdani-Biuki B, Wolf G, Wascher TC, Paulweber B, Haas J, Samonigg H (2003) A common 936 C/T gene polymorphism of vascular endothelial growth factor is associated with decreased breast cancer risk. International journal of cancer Journal international du cancer 106:468-471.

Kuschel B, Auranen A, McBride S, Novik KL, Antoniou A, Lipscombe JM, Day NE, Easton DF, Ponder BA, Pharoah PD, Dunning A (2002) Variants in DNA double-strand break repair genes and breast cancer susceptibility. Human molecular genetics 11:1399-1407.

Labrecque LG, Barnes DM, Fentiman IS, Griffin BE (1995) Epstein-Barr virus in epithelial cell tumors: a breast cancer study. Cancer research 55:39-45.

Lavigne JA, Helzlsouer KJ, Huang HY, Strickland PT, Bell DA, Selmin O, Watson MA, Hoffman S, Comstock GW, Yager JD (1997) An association between the allele coding for a low activity variant of catechol-O-methyltransferase and the risk for breast cancer. Cancer research 57:5493-5497.

Le Marchand L, Kolonel LN, Myers BC, Mi MP (1988) Birth characteristics of premenopausal women with breast cancer. British journal of cancer 57:437-439.

Lee HP, Gourley L, Duffy SW, Esteve J, Lee J, Day NE (1991) Dietary effects on breast-cancer risk in Singapore. Lancet 337:1197-1200.

Lidereau R, Escot C, Theillet C, Champeme MH, Brunet M, Gest J, Callahan R (1986) High frequency of rare alleles of the human c-Ha-ras-1 proto-oncogene in breast cancer patients. Journal of the National Cancer Institute 77:697-701.

Lowe LC, Guy M, Mansi JL, Peckitt C, Bliss J, Wilson RG, Colston KW (2005) Plasma 25-hydroxy vitamin D concentrations, vitamin D receptor genotype and breast cancer risk in a UK Caucasian population. European journal of cancer (Oxford, England : 1990) 41:1164-1169.

Lubin F, Wax Y, Modan B (1986) Role of fat, animal protein, and dietary fiber in breast cancer etiology: a case-control study. Journal of the National Cancer Institute 77:605-612.

Manjer J, Kaaks R, Riboli E, Berglund G (2001) Risk of breast cancer in relation to anthropometry, blood pressure, blood lipids and glucose metabolism: a prospective study within the Malmo Preventive Project. European journal of cancer prevention : the official journal of the European Cancer Prevention Organisation (ECP) 10:33-42.

Meijers-Heijboer H, van den Ouweland A, Klijn J, Wasielewski M, de Snoo A, Oldenburg R, Hollestelle A, Houben M, Crepin E, van Veghel-Plandsoen M, Elstrodt F, van Duijn C, Bartels C, Meijers C, Schutte M, McGuffog L, Thompson D, Easton D, Sodha N, Seal S, Barfoot R, Mangion J, Chang-Claude J, Eccles D, Eeles R, Evans DG, Houlston R, Murday V, Narod S, Peretz T, Peto J, Phelan C, Zhang HX, Szabo C, Devilee P, Goldgar D, Futreal PA, Nathanson KL, Weber B, Rahman N, Stratton MR (2002) Low-penetrance susceptibility to breast cancer due to CHEK2(*)1100delC in noncarriers of BRCA1 or BRCA2 mutations. Nature genetics 31:55-59.

Mills b PK, Beeson WL, Phillips RL, Fraser GE (1989) Dietary habits and breast cancer incidence among Seventh-day Adventists. Cancer 64:582-590.

Mills PK, Beeson WL, Phillips RL, Fraser GE (1989) Prospective study of exogenous hormone use and breast cancer in Seventh-day Adventists. Cancer 64:591-597.

Mittra I, Perrin J, Kumaoka S (1976) Thyroid and other autoantibodies in British and Japanese women: an epidemiological study of breast cancer. British medical journal 1:257-259.

Nordgard SH, Ritchie MD, Jensrud SD, Motsinger AA, Alnaes GI, Lemmon G, Berg M, Geisler S, Moore JH, Lonning PE, Borresen-Dale AL, Kristensen VN (2007) ABCB1 and GST polymorphisms associated with TP53 status in breast cancer. Pharmacogenetics and genomics 17:127-136.

Paffenbarger RS, Jr., Fasal E, Simmons ME, Kampert JB (1977) Cancer risk as related to use of oral contraceptives during fertile years. Cancer 39:1887-1891.

Park KS, Mok JW, Ko HE, Tokunaga K, Lee MH (2002) Polymorphisms of tumour necrosis factors A and B in breast cancer. European journal of immunogenetics : official journal of the British Society for Histocompatibility and Immunogenetics 29:7-10.

Pasche B, Kolachana P, Nafa K, Satagopan J, Chen YG, Lo RS, Brener D, Yang D, Kirstein L, Oddoux C, Ostrer H, Vineis P, Varesco L, Jhanwar S, Luzzatto L, Massague J, Offit K (1999) TbetaR-I(6A) is a candidate tumor susceptibility allele. Cancer research 59:5678-5682.

Polednak AP, Janerich DT (1983) Characteristics of first pregnancy in relation to early breast cancer. A case-control study. The Journal of reproductive medicine 28:314-318.

Potter JD, Cerhan JR, Sellers TA, McGovern PG, Drinkard C, Kushi LR, Folsom AR (1995) Progesterone and estrogen receptors and mammary neoplasia in the Iowa Women's Health Study: how many kinds of breast cancer are there? Cancer epidemiology, biomarkers & prevention : a publication of the American Association for Cancer Research, cosponsored by the American Society of Preventive Oncology 4:319-326.

Rafii S, O'Regan P, Xinarianos G, Azmy I, Stephenson T, Reed M, Meuth M, Thacker J, Cox A (2002) A potential role for the XRCC2 R188H polymorphic site in DNA-damage repair and breast cancer. Human molecular genetics 11:1433-1438.

Ren Z, Cai Q, Shu XO, Cai H, Li C, Yu H, Gao YT, Zheng W (2004) Genetic polymorphisms in the IGFBP3 gene: association with breast cancer risk and blood IGFBP-3 protein levels among Chinese women. Cancer epidemiology, biomarkers & prevention : a publication of the American Association for Cancer Research, cosponsored by the American Society of Preventive Oncology 13:1290-1295.

Ruggiero M, Pacini S, Aterini S, Fallai C, Ruggiero C, Pacini P (1998) Vitamin D receptor gene polymorphism is associated with metastatic breast cancer. Oncology research 10:43-46.

Russo A, Autelitano M, Bisanti L (2008) Metabolic syndrome and cancer risk. European journal of cancer (Oxford, England : 1990) 44:293-297.

Sharp L, Little J, Schofield AC, Pavlidou E, Cotton SC, Miedzybrodzka Z, Baird JO, Haites NE, Heys SD, Grubb DA (2002) Folate and breast cancer: the role of polymorphisms in methylenetetrahydrofolate reductase (MTHFR). Cancer letters 181:65-71.

Shibata A, Paganini-Hill A, Ross RK, Henderson BE (1992) Intake of vegetables, fruits, beta-carotene, vitamin C and vitamin supplements and cancer incidence among the elderly: a prospective study. British journal of cancer 66:673-679.

Shrubsole MJ, Gao YT, Cai Q, Shu XO, Dai Q, Jin F, Zheng W (2006) MTR and MTRR polymorphisms, dietary intake, and breast cancer risk. Cancer epidemiology, biomarkers & prevention : a publication of the American Association for Cancer Research, cosponsored by the American Society of Preventive Oncology 15:586-588.

Siegelmann-Danieli N, Buetow KH (2002) Significance of genetic variation at the glutathione S-transferase M1 and NAD(P)H:quinone oxidoreductase 1 detoxification genes in breast cancer development. Oncology 62:39-45.

Simard A, Vobecky J, Vobecky JS (1991) Vitamin D deficiency and cancer of the breast: an unprovocative ecological hypothesis. Canadian journal of public health = Revue canadienne de sante publique 82:300-303.

Sjalander A, Birgander R, Hallmans G, Cajander S, Lenner P, Athlin L, Beckman G, Beckman L (1996) p53 polymorphisms and haplotypes in breast cancer. Carcinogenesis 17:1313-1316.

Snowdon DA, Phillips RL (1984) Coffee consumption and risk of fatal cancers. American journal of public health 74:820-823.

Spurdle AB, Dite GS, Chen X, Mayne CJ, Southey MC, Batten LE, Chy H, Trute L, McCredie MR, Giles GG, Armes J, Venter DJ, Hopper JL, Chenevix-Trench G (1999) Androgen receptor exon 1 CAG repeat length and breast cancer in women before age forty years. Journal of the National Cancer Institute 91:961-966.

Tang D, Cho S, Rundle A, Chen S, Phillips D, Zhou J, Hsu Y, Schnabel F, Estabrook A, Perera FP (2002) Polymorphisms in the DNA repair enzyme XPD are associated with increased levels of PAH-DNA adducts in a case-control study of breast cancer. Breast cancer research and treatment 75:159-166.

Thompson WD, Jacobson HI, Negrini B, Janerich DT (1989) Hypertension, pregnancy, and risk of breast cancer. Journal of the National Cancer Institute 81:1571-1574.

Toniolo P, Riboli E, Shore RE, Pasternack BS (1994) Consumption of meat, animal products, protein, and fat and risk of breast cancer: a prospective cohort study in New York. Epidemiology (Cambridge, Mass) 5:391-397.

Toniolo P, Van Kappel AL, Akhmedkhanov A, Ferrari P, Kato I, Shore RE, Riboli E (2001) Serum carotenoids and breast cancer. American journal of epidemiology 153:1142-1147.

Van Zee KJ, Calvano JE, Bisogna M (1998) Hypomethylation and increased gene expression of p16INK4a in primary and metastatic breast carcinoma as compared to normal breast tissue. Oncogene 16:2723-2727.

Vatten LJ, Kvinnsland S (1992) Prospective study of height, body mass index and risk of breast cancer. Acta oncologica (Stockholm, Sweden) 31:195-200.

Vogel U, Nexo BA, Olsen A, Thomsen B, Jacobsen NR, Wallin H, Overvad K, Tjonneland A (2003) No association between OGG1 Ser326Cys polymorphism and breast cancer risk. Cancer epidemiology, biomarkers & prevention : a publication of the American Association for Cancer Research, cosponsored by the American Society of Preventive Oncology 12:170-171.

Wald NJ, Boreham J, Hayward JL, Bulbrook RD (1984) Plasma retinol, beta-carotene and vitamin E levels in relation to the future risk of breast cancer. British journal of cancer 49:321-324.

Wallace RB, Sherman BM, Bean JA (1982) A case-control study of breast cancer and psychotropic drug use. Oncology 39:279-283.

Wilkening S, Bermejo JL, Burwinkel B, Klaes R, Bartram CR, Meindl A, Bugert P, Schmutzler RK, Wappenschmidt B, Untch M, Hemminki K, Forsti A (2006) The single nucleotide polymorphism IVS1+309 in mouse double minute 2 does not affect risk of familial breast cancer. Cancer research 66:646-648.

Woo HY, Park H, Ki CS, Park YL, Bae WG (2006) Relationships among serum leptin, leptin receptor gene polymorphisms, and breast cancer in Korea. Cancer letters 237:137-142.

Xie D, Shu XO, Deng Z, Wen WQ, Creek KE, Dai Q, Gao YT, Jin F, Zheng W (2000) Population-based, case-control study of HER2 genetic polymorphism and breast cancer risk. Journal of the National Cancer Institute 92:412-417.

Yaich L, Dupont WD, Cavener DR, Parl FF (1992) Analysis of the PvuII restriction fragment-length polymorphism and exon structure of the estrogen receptor gene in breast cancer and peripheral blood. Cancer research 52:77-83.

Yu H, Li BD, Smith M, Shi R, Berkel HJ, Kato I (2001) Polymorphic CA repeats in the IGF-I gene and breast cancer. Breast cancer research and treatment 70:117-122.

Yu Y, Morimoto T, Sasa M, Okazaki K, Harada Y, Fujiwara T, Irie Y, Takahashi E, Tanigami A, Izumi K (1999) HPV33 DNA in premalignant and malignant breast lesions in Chinese and Japanese populations. Anticancer research 19:5057-5061.

Zheng T, Holford TR, Mayne ST, Owens PH, Zhang B, Boyle P, Carter D, Ward B, Zhang Y, Zahm SH (2000) Exposure to electromagnetic fields from use of electric blankets and other in-home electrical appliances and breast cancer risk. American journal of epidemiology 151:1103-1111.

Zheng W, Long J, Gao YT, Li C, Zheng Y, Xiang YB, Wen W, Levy S, Deming SL, Haines JL, Gu K, Fair AM, Cai Q, Lu W, Shu XO (2009) Genome-wide association study identifies a new breast cancer susceptibility locus at 6q25.1. Nature genetics 41:324-328.

Zheng W, Xie D, Cerhan JR, Sellers TA, Wen W, Folsom AR (2001) Sulfotransferase 1A1 polymorphism, endogenous estrogen exposure, well-done meat intake, and breast cancer risk. Cancer epidemiology, biomarkers & prevention : a publication of the American Association for Cancer Research, cosponsored by the American Society of Preventive Oncology 10:89-94.

Zhong S, Wyllie AH, Barnes D, Wolf CR, Spurr NK (1993) Relationship between the GSTM1 genetic polymorphism and susceptibility to bladder, breast and colon cancer. Carcinogenesis 14:1821-1824.

Ziv E, Cauley J, Morin PA, Saiz R, Browner WS (2001) Association between the T29-->C polymorphism in the transforming growth factor beta1 gene and breast cancer among elderly white women: The Study of Osteoporotic Fractures. JAMA : the journal of the American Medical Association 285:2859-2863.

**Glaucoma**

Alward WL, Fingert JH, Coote MA, Johnson AT, Lerner SF, Junqua D, Durcan FJ, McCartney PJ, Mackey DA, Sheffield VC, Stone EM (1998) Clinical features associated with mutations in the chromosome 1 open-angle glaucoma gene (GLC1A). The New England journal of medicine 338:1022-1027.

Aung T, Ocaka L, Ebenezer ND, Morris AG, Krawczak M, Thiselton DL, Alexander C, Votruba M, Brice G, Child AH, Francis PJ, Hitchings RA, Lehmann OJ, Bhattacharya SS (2002) A major marker for normal tension glaucoma: association with polymorphisms in the OPA1 gene. Human genetics 110:52-56.

Bleich S, Junemann A, von Ahsen N, Lausen B, Ritter K, Beck G, Naumann GO, Kornhuber J (2002) Homocysteine and risk of open-angle glaucoma. Journal of neural transmission (Vienna, Austria : 1996) 109:1499-1504.

Fingert JH, Heon E, Liebmann JM, Yamamoto T, Craig JE, Rait J, Kawase K, Hoh ST, Buys YM, Dickinson J, Hockey RR, Williams-Lyn D, Trope G, Kitazawa Y, Ritch R, Mackey DA, Alward WL, Sheffield VC, Stone EM (1999) Analysis of myocilin mutations in 1703 glaucoma patients from five different populations. Human molecular genetics 8:899-905.

Lin HJ, Chen WC, Tsai FJ, Tsai SW (2002) Distributions of p53 codon 72 polymorphism in primary open angle glaucoma. The British journal of ophthalmology 86:767-770.

Lin HJ, Tsai FJ, Chen WC, Shi YR, Hsu Y, Tsai SW (2003) Association of tumour necrosis factor alpha -308 gene polymorphism with primary open-angle glaucoma in Chinese. Eye (London, England) 17:31-34.

Mitchell P, Hourihan F, Sandbach J, Wang JJ (1999) The relationship between glaucoma and myopia: the Blue Mountains Eye Study. Ophthalmology 106:2010-2015.

Ponte F, Giuffre G, Giammanco R, Dardanoni G (1994) Risk factors of ocular hypertension and glaucoma. The Casteldaccia Eye Study. Documenta ophthalmologica Advances in ophthalmology 85:203-210.

Rezaie T, Child A, Hitchings R, Brice G, Miller L, Coca-Prados M, Heon E, Krupin T, Ritch R, Kreutzer D, Crick RP, Sarfarazi M (2002) Adult-onset primary open-angle glaucoma caused by mutations in optineurin. Science (New York, NY) 295:1077-1079.

Tang S, Toda Y, Kashiwagi K, Mabuchi F, Iijima H, Tsukahara S, Yamagata Z (2003) The association between Japanese primary open-angle glaucoma and normal tension glaucoma patients and the optineurin gene. Human genetics 113:276-279.

Thorleifsson G, Magnusson KP, Sulem P, Walters GB, Gudbjartsson DF, Stefansson H, Jonsson T, Jonasdottir A, Jonasdottir A, Stefansdottir G, Masson G, Hardarson GA, Petursson H, Arnarsson A, Motallebipour M, Wallerman O, Wadelius C, Gulcher JR, Thorsteinsdottir U, Kong A, Jonasson F, Stefansson K (2007) Common sequence variants in the LOXL1 gene confer susceptibility to exfoliation glaucoma. Science (New York, NY) 317:1397-1400.

Yao W, Jiao X, Hejtmancik JF, Leske MC, Hennis A, Nemesure B (2006) Evaluation of the association between OPA1 polymorphisms and primary open-angle glaucoma in Barbados families. Molecular vision 12:649-654.

**Psoriasis**

Brenner W, Gschnait F, Mayr WR (1978) HLA B13, B17, B37 and Cw6 in psoriasis vulgaris: association with the age of onset. Archives of dermatological research 262:337-339.

Capon F, Di Meglio P, Szaub J, Prescott NJ, Dunster C, Baumber L, Timms K, Gutin A, Abkevic V, Burden AD, Lanchbury J, Barker JN, Trembath RC, Nestle FO (2007) Sequence variants in the genes for the interleukin-23 receptor (IL23R) and its ligand (IL12B) confer protection against psoriasis. Human genetics 122:201-206.

Cargill M, Schrodi SJ, Chang M, Garcia VE, Brandon R, Callis KP, Matsunami N, Ardlie KG, Civello D, Catanese JJ, Leong DU, Panko JM, McAllister LB, Hansen CB, Papenfuss J, Prescott SM, White TJ, Leppert MF, Krueger GG, Begovich AB (2007) A large-scale genetic association study confirms IL12B and leads to the identification of IL23R as psoriasis-risk genes. American journal of human genetics 80:273-290.

Chaput JC, Poynard T, Naveau S, Penso D, Durrmeyer O, Suplisson D (1985) Psoriasis, alcohol, and liver disease. British medical journal (Clinical research ed) 291:25.

Criswell LA, Pfeiffer KA, Lum RF, Gonzales B, Novitzke J, Kern M, Moser KL, Begovich AB, Carlton VE, Li W, Lee AT, Ortmann W, Behrens TW, Gregersen PK (2005) Analysis of families in the multiple autoimmune disease genetics consortium (MADGC) collection: the PTPN22 620W allele associates with multiple autoimmune phenotypes. American journal of human genetics 76:561-571.

Furumoto H, Nakamura K, Imamura T, Hamamoto Y, Shimizu T, Muto M, Asagami C (1997) Association of apolipoprotein allele epsilon 2 with psoriasis vulgaris in Japanese population. Archives of dermatological research 289:497-500.

Kontula K, Valimaki S, Kainulainen K, Viitanen AM, Keski-Oja J (1997) Vitamin D receptor polymorphism and treatment of psoriasis with calcipotriol. The British journal of dermatology 136:977-978.

Mills CM, Srivastava ED, Harvey IM, Swift GL, Newcombe RG, Holt PJ, Rhodes J (1992) Smoking habits in psoriasis: a case control study. The British journal of dermatology 127:18-21.

Okita H, Ohtsuka T, Yamakage A, Yamazaki S (2002) Polymorphism of the vitamin D(3) receptor in patients with psoriasis. Archives of dermatological research 294:159-162.

Park BS, Park JS, Lee DY, Youn JI, Kim IG (1999) Vitamin D receptor polymorphism is associated with psoriasis. The Journal of investigative dermatology 112:113-116.

Young HS, Summers AM, Bhushan M, Brenchley PE, Griffiths CE (2004) Single-nucleotide polymorphisms of vascular endothelial growth factor in psoriasis of early onset. The Journal of investigative dermatology 122:209-215.

Zhang X, Wang H, Te-Shao H, Yang S, Wang F (2002) Frequent use of tobacco and alcohol in Chinese psoriasis patients. International journal of dermatology 41:659-662.

**Rheumatoid Arthritis**

Barton A, Jury F, Eyre S, Bowes J, Hinks A, Ward D, Worthington J (2004) Haplotype analysis in simplex families and novel analytic approaches in a case-control cohort reveal no evidence of association of the CTLA-4 gene with rheumatoid arthritis. Arthritis and rheumatism 50:748-752.

Begovich AB, Carlton VE, Honigberg LA, Schrodi SJ, Chokkalingam AP, Alexander HC, Ardlie KG, Huang Q, Smith AM, Spoerke JM, Conn MT, Chang M, Chang SY, Saiki RK, Catanese JJ, Leong DU, Garcia VE, McAllister LB, Jeffery DA, Lee AT, Batliwalla F, Remmers E, Criswell LA, Seldin MF, Kastner DL, Amos CI, Sninsky JJ, Gregersen PK (2004) A missense single-nucleotide polymorphism in a gene encoding a protein tyrosine phosphatase (PTPN22) is associated with rheumatoid arthritis. American journal of human genetics 75:330-337.

Breunis WB, van Mirre E, Geissler J, Laddach N, Wolbink G, van der Schoot E, de Haas M, de Boer M, Roos D, Kuijpers TW (2009) Copy number variation at the FCGR locus includes FCGR3A, FCGR2C and FCGR3B but not FCGR2A and FCGR2B. Human mutation 30:E640-650.

Cantagrel A, Navaux F, Loubet-Lescoulie P, Nourhashemi F, Enault G, Abbal M, Constantin A, Laroche M, Mazieres B (1999) Interleukin-1beta, interleukin-1 receptor antagonist, interleukin-4, and interleukin-10 gene polymorphisms: relationship to occurrence and severity of rheumatoid arthritis. Arthritis and rheumatism 42:1093-1100.

Coakley G, Mok CC, Hajeer AH, Ollier WE, Turner D, Sinnott PJ, Hutchinson IV, Panayi GS, Lanchbury JS (1998) Interleukin-10 promoter polymorphisms in rheumatoid arthritis and Felty's syndrome. British journal of rheumatology 37:988-991.

Cooke SP, Forrest G, Venables PJ, Hajeer A (1998) The delta32 deletion of CCR5 receptor in rheumatoid arthritis. Arthritis and rheumatism 41:1135-1136.

Kochi Y, Yamada R, Suzuki A, Harley JB, Shirasawa S, Sawada T, Bae SC, Tokuhiro S, Chang X, Sekine A, Takahashi A, Tsunoda T, Ohnishi Y, Kaufman KM, Kang CP, Kang C, Otsubo S, Yumura W, Mimori A, Koike T, Nakamura Y, Sasazuki T, Yamamoto K (2005) A functional variant in FCRL3, encoding Fc receptor-like 3, is associated with rheumatoid arthritis and several autoimmunities. Nature genetics 37:478-485.

Layton MA, Jones PW, Alldersea JE, Strange RC, Fryer AA, Dawes PT, Mattey DL (1999) The therapeutic response to D-penicillamine in rheumatoid arthritis: influence of glutathione S-transferase polymorphisms. Rheumatology (Oxford, England) 38:43-47.

Mattey DL, Hassell AB, Plant M, Dawes PT, Ollier WR, Jones PW, Fryer AA, Alldersea JE, Strange RC (1999) Association of polymorphism in glutathione S-transferase loci with susceptibility and outcome in rheumatoid arthritis: comparison with the shared epitope. Annals of the rheumatic diseases 58:164-168.

Nieto A, Caliz R, Pascual M, Mataran L, Garcia S, Martin J (2000) Involvement of Fcgamma receptor IIIA genotypes in susceptibility to rheumatoid arthritis. Arthritis and rheumatism 43:735-739.

Perrier S, Coussediere C, Dubost JJ, Albuisson E, Sauvezie B (1998) IL-1 receptor antagonist (IL-1RA) gene polymorphism in Sjogren's syndrome and rheumatoid arthritis. Clinical immunology and immunopathology 87:309-313.

Plenge b RM, Seielstad M, Padyukov L, Lee AT, Remmers EF, Ding B, Liew A, Khalili H, Chandrasekaran A, Davies LR, Li W, Tan AK, Bonnard C, Ong RT, Thalamuthu A, Pettersson S, Liu C, Tian C, Chen WV, Carulli JP, Beckman EM, Altshuler D, Alfredsson L, Criswell LA, Amos CI, Seldin MF, Kastner DL, Klareskog L, Gregersen PK (2007) TRAF1-C5 as a risk locus for rheumatoid arthritis--a genomewide study. The New England journal of medicine 357:1199-1209.

Plenge RM, Cotsapas C, Davies L, Price AL, de Bakker PI, Maller J, Pe'er I, Burtt NP, Blumenstiel B, DeFelice M, Parkin M, Barry R, Winslow W, Healy C, Graham RR, Neale BM, Izmailova E, Roubenoff R, Parker AN, Glass R, Karlson EW, Maher N, Hafler DA, Lee DM, Seldin MF, Remmers EF, Lee AT, Padyukov L, Alfredsson L, Coblyn J, Weinblatt ME, Gabriel SB, Purcell S, Klareskog L, Gregersen PK, Shadick NA, Daly MJ, Altshuler D (2007) Two independent alleles at 6q23 associated with risk of rheumatoid arthritis. Nature genetics 39:1477-1482.

Remmers EF, Plenge RM, Lee AT, Graham RR, Hom G, Behrens TW, de Bakker PI, Le JM, Lee HS, Batliwalla F, Li W, Masters SL, Booty MG, Carulli JP, Padyukov L, Alfredsson L, Klareskog L, Chen WV, Amos CI, Criswell LA, Seldin MF, Kastner DL, Gregersen PK (2007) STAT4 and the risk of rheumatoid arthritis and systemic lupus erythematosus. The New England journal of medicine 357:977-986.

Rueda B, Reddy MV, Gonzalez-Gay MA, Balsa A, Pascual-Salcedo D, Petersson IF, Eimon A, Paira S, Scherbarth HR, Pons-Estel BA, Gonzalez-Escribano MF, Alarcon-Riquelme ME, Martin J (2006) Analysis of IRF5 gene functional polymorphisms in rheumatoid arthritis. Arthritis and rheumatism 54:3815-3819.

Seidl C, Donner H, Fischer B, Usadel KH, Seifried E, Kaltwasser JP, Badenhoop K (1998) CTLA4 codon 17 dimorphism in patients with rheumatoid arthritis. Tissue antigens 51:62-66.

Sigurdsson S, Padyukov L, Kurreeman FA, Liljedahl U, Wiman AC, Alfredsson L, Toes R, Ronnelid J, Klareskog L, Huizinga TW, Alm G, Syvanen AC, Ronnblom L (2007) Association of a haplotype in the promoter region of the interferon regulatory factor 5 gene with rheumatoid arthritis. Arthritis and rheumatism 56:2202-2210.

Sivalingam SP, Yoon KH, Koh DR, Fong KY (2003) Single-nucleotide polymorphisms of the interleukin-18 gene promoter region in rheumatoid arthritis patients: protective effect of AA genotype. Tissue antigens 62:498-504.

Sugiura Y, Niimi T, Sato S, Yoshinouchi T, Banno S, Naniwa T, Maeda H, Shimizu S, Ueda R (2002) Transforming growth factor beta1 gene polymorphism in rheumatoid arthritis. Annals of the rheumatic diseases 61:826-828.

Suzuki A, Yamada R, Chang X, Tokuhiro S, Sawada T, Suzuki M, Nagasaki M, Nakayama-Hamada M, Kawaida R, Ono M, Ohtsuki M, Furukawa H, Yoshino S, Yukioka M, Tohma S, Matsubara T, Wakitani S, Teshima R, Nishioka Y, Sekine A, Iida A, Takahashi A, Tsunoda T, Nakamura Y, Yamamoto K (2003) Functional haplotypes of PADI4, encoding citrullinating enzyme peptidylarginine deiminase 4, are associated with rheumatoid arthritis. Nature genetics 34:395-402.

Swanberg M, Lidman O, Padyukov L, Eriksson P, Akesson E, Jagodic M, Lobell A, Khademi M, Borjesson O, Lindgren CM, Lundman P, Brookes AJ, Kere J, Luthman H, Alfredsson L, Hillert J, Klareskog L, Hamsten A, Piehl F, Olsson T (2005) MHC2TA is associated with differential MHC molecule expression and susceptibility to rheumatoid arthritis, multiple sclerosis and myocardial infarction. Nature genetics 37:486-494.

The WTCCC-Burton PR (2007) Genome-wide association study of 14,000 cases of seven common diseases and 3,000 shared controls. Nature 447:661-678.

Thomson W, Barton A, Ke X, Eyre S, Hinks A, Bowes J, Donn R, Symmons D, Hider S, Bruce IN, Wilson AG, Marinou I, Morgan A, Emery P, Carter A, Steer S, Hocking L, Reid DM, Wordsworth P, Harrison P, Strachan D, Worthington J (2007) Rheumatoid arthritis association at 6q23. Nature genetics 39:1431-1433.

Tokuhiro S, Yamada R, Chang X, Suzuki A, Kochi Y, Sawada T, Suzuki M, Nagasaki M, Ohtsuki M, Ono M, Furukawa H, Nagashima M, Yoshino S, Mabuchi A, Sekine A, Saito S, Takahashi A, Tsunoda T, Nakamura Y, Yamamoto K (2003) An intronic SNP in a RUNX1 binding site of SLC22A4, encoding an organic cation transporter, is associated with rheumatoid arthritis. Nature genetics 35:341-348.

Vessey MP, Villard-Mackintosh L, Yeates D (1987) Oral contraceptives, cigarette smoking and other factors in relation to arthritis. Contraception 35:457-464.
