# Supplementary material for: Replication Validity of Initial Association Studies: A Comparison between Psychiatry, Neurology and Four Somatic Diseases
Source: PLoS One. 2016 Jun 23;11(6):e0158064. doi: 10.1371/journal.pone.0158064 (PMC4919034; doi:10.1371/journal.pone.0158064)
Supplement: S3 Text — (DOCX) [file pone.0158064.s005.docx]

**References for largest studies**

**ADHD**

Abikoff HB, Jensen PS, Arnold LL, Hoza B, Hechtman L, Pollack S, Martin D, Alvir J, March JS, Hinshaw S, Vitiello B, Newcorn J, Greiner A, Cantwell DP, Conners CK, Elliott G, Greenhill LL, Kraemer H, Pelham WE, Jr., Severe JB, Swanson JM, Wells K, Wigal T (2002) Observed classroom behavior of children with ADHD: relationship to gender and comorbidity. Journal of abnormal child psychology 30:349-359.

Arcia E, Conners CK (1998) Gender differences in ADHD? Journal of developmental and behavioral pediatrics : JDBP 19:77-83.

Bateman B, Warner JO, Hutchinson E, Dean T, Rowlandson P, Gant C, Grundy J, Fitzgerald C, Stevenson J (2004) The effects of a double blind, placebo controlled, artificial food colourings and benzoate preservative challenge on hyperactivity in a general population sample of preschool children. Archives of disease in childhood 89:506-511.

Brookes K, Xu X, Chen W, Zhou K, Neale B, Lowe N, Anney R, Franke B, Gill M, Ebstein R, Buitelaar J, Sham P, Campbell D, Knight J, Andreou P, Altink M, Arnold R, Boer F, Buschgens C, Butler L, Christiansen H, Feldman L, Fleischman K, Fliers E, Howe-Forbes R, Goldfarb A, Heise A, Gabriels I, Korn-Lubetzki I, Johansson L, Marco R, Medad S, Minderaa R, Mulas F, Muller U, Mulligan A, Rabin K, Rommelse N, Sethna V, Sorohan J, Uebel H, Psychogiou L, Weeks A, Barrett R, Craig I, Banaschewski T, Sonuga-Barke E, Eisenberg J, Kuntsi J, Manor I, McGuffin P, Miranda A, Oades RD, Plomin R, Roeyers H, Rothenberger A, Sergeant J, Steinhausen HC, Taylor E, Thompson M, Faraone SV, Asherson P (2006) The analysis of 51 genes in DSM-IV combined type attention deficit hyperactivity disorder: association signals in DRD4, DAT1 and 16 other genes. Molecular psychiatry 11:934-953.

Castellanos FX, Giedd JN, Berquin PC, Walter JM, Sharp W, Tran T, Vaituzis AC, Blumenthal JD, Nelson J, Bastain TM, Zijdenbos A, Evans AC, Rapoport JL (2001) Quantitative brain magnetic resonance imaging in girls with attention-deficit/hyperactivity disorder. Archives of general psychiatry 58:289-295.

Coutinho G, Mattos P, Malloy-Diniz LF (2009) Neuropsychological differences between attention deficit hyperactivity disorder and control children and adolescents referred for academic impairment. Revista brasileira de psiquiatria (Sao Paulo, Brazil : 1999) 31:141-144.

Donfrancesco R, Parisi P, Vanacore N, Martines F, Sargentini V, Cortese S (2013) Iron and ADHD: Time to Move Beyond Serum Ferritin Levels. Journal of attention disorders 17:347-357.

Dvorakova M, Jezova D, Blazicek P, Trebaticka J, Skodacek I, Suba J, Iveta W, Rohdewald P, Durackova Z (2007) Urinary catecholamines in children with attention deficit hyperactivity disorder (ADHD): modulation by a polyphenolic extract from pine bark (pycnogenol). Nutritional neuroscience 10:151-157.

Gau SS, Shang CY (2010) Executive functions as endophenotypes in ADHD: evidence from the Cambridge Neuropsychological Test Battery (CANTAB). Journal of child psychology and psychiatry, and allied disciplines 51:838-849.

Herrmann MJ, Mader K, Schreppel T, Jacob C, Heine M, Boreatti-Hummer A, Ehlis AC, Scheuerpflug P, Pauli P, Fallgatter AJ (2010) Neural correlates of performance monitoring in adult patients with attention deficit hyperactivity disorder (ADHD). The world journal of biological psychiatry : the official journal of the World Federation of Societies of Biological Psychiatry 11:457-464.

Huang YS, Chen NH, Li HY, Wu YY, Chao CC, Guilleminault C (2004) Sleep disorders in Taiwanese children with attention deficit/hyperactivity disorder. Journal of sleep research 13:269-277.

Kalff AC, De Sonneville LM, Hurks PP, Hendriksen JG, Kroes M, Feron FJ, Steyaert J, van Zeben TM, Vles JS, Jolles J (2005) Speed, speed variability, and accuracy of information processing in 5 to 6-year-old children at risk of ADHD. Journal of the International Neuropsychological Society : JINS 11:173-183.

Kalff AC, Hendriksen JG, Kroes M, Vles JS, Steyaert J, Feron FJ, van Zeben TM, Jolles J (2002) Neurocognitive performance of 5- and 6-year-old children who met criteria for attention deficit/hyperactivity disorder at 18 months follow-up: results from a prospective population study. Journal of abnormal child psychology 30:589-598.

Kusaga A, Yamashita Y, Koeda T, Hiratani M, Kaneko M, Yamada S, Matsuishi T (2002) Increased urine phenylethylamine after methylphenidate treatment in children with ADHD. Annals of neurology 52:372-374.

Kustanovich V, Ishii J, Crawford L, Yang M, McGough JJ, McCracken JT, Smalley SL, Nelson SF (2004) Transmission disequilibrium testing of dopamine-related candidate gene polymorphisms in ADHD: confirmation of association of ADHD with DRD4 and DRD5. Molecular psychiatry 9:711-717.

Kustanovich V, Merriman B, McGough J, McCracken JT, Smalley SL, Nelson SF (2003) Biased paternal transmission of SNAP-25 risk alleles in attention-deficit hyperactivity disorder. Molecular psychiatry 8:309-315.

Loo SK, Cho A, Hale TS, McGough J, McCracken J, Smalley SL (2013) Characterization of the theta to beta ratio in ADHD: identifying potential sources of heterogeneity. Journal of attention disorders 17:384-392.

Mahone EM, Mostofsky SH, Lasker AG, Zee D, Denckla MB (2009) Oculomotor anomalies in attention-deficit/hyperactivity disorder: evidence for deficits in response preparation and inhibition. Journal of the American Academy of Child and Adolescent Psychiatry 48:749-756.

Motion S, Northstone K, Emond A (2001) Persistent early feeding difficulties and subsequent growth and developmental outcomes. Ambulatory Child Health 7:231-237.

Rhodes SM, Coghill DR, Matthews K (2004) Methylphenidate restores visual memory, but not working memory function in attention deficit-hyperkinetic disorder. Psychopharmacology 175:319-330.

Schachar R, Chen S, Crosbie J, Goos L, Ickowicz A, Charach A (2007) Comparison of the predictive validity of hyperkinetic disorder and attention deficit hyperactivity disorder. Journal of the Canadian Academy of Child and Adolescent Psychiatry = Journal de l'Academie canadienne de psychiatrie de l'enfant et de l'adolescent 16:90-100.

Shen YC, Wang YF (1984) Urinary 3-methoxy-4-hydroxyphenylglycol sulfate excretion in seventy-three schoolchildren with minimal brain dysfunction syndrome. Biological psychiatry 19:861-870.

Uebel H, Albrecht B, Asherson P, Borger NA, Butler L, Chen W, Christiansen H, Heise A, Kuntsi J, Schafer U, Andreou P, Manor I, Marco R, Miranda A, Mulligan A, Oades RD, van der Meere J, Faraone SV, Rothenberger A, Banaschewski T (2010) Performance variability, impulsivity errors and the impact of incentives as gender-independent endophenotypes for ADHD. Journal of child psychology and psychiatry, and allied disciplines 51:210-218.

van West D, Claes S, Deboutte D (2009) Differences in hypothalamic-pituitary-adrenal axis functioning among children with ADHD predominantly inattentive and combined types. European child & adolescent psychiatry 18:543-553.

Volkow ND, Wang GJ, Kollins SH, Wigal TL, Newcorn JH, Telang F, Fowler JS, Zhu W, Logan J, Ma Y, Pradhan K, Wong C, Swanson JM (2009) Evaluating dopamine reward pathway in ADHD: clinical implications. JAMA 302:1084-1091.

von Stauffenberg C, Campbell SB (2007) Predicting the Early Developmental Course of Symptoms of Attention Deficit Hyperactivity Disorder. Journal of applied developmental psychology 28:536-552.

Xu X, Duman EA, Anney R, Brookes K, Franke B, Zhou K, Buschgens C, Chen W, Christiansen H, Eisenberg J, Gabriels I, Manor I, Marco R, Muller UC, Mulligan A, Rommelse N, Thompson M, Uebel H, Banaschewski T, Buitelaar J, Ebstein R, Gill M, Miranda A, Mulas F, Oades RD, Roeyers H, Rothenberger A, Sergeant J, Sonuga-Barke E, Steinhausen HC, Taylor E, Faraone SV, Asherson P (2008) No association between two polymorphisms of the serotonin transporter gene and combined type attention deficit hyperactivity disorder. American journal of medical genetics Part B, Neuropsychiatric genetics : the official publication of the International Society of Psychiatric Genetics 147B:1306-1309.

**Autism**

Buchmayer S, Johansson S, Johansson A, Hultman CM, Sparen P, Cnattingius S (2009) Can association between preterm birth and autism be explained by maternal or neonatal morbidity? Pediatrics 124:e817-825.

Carper RA, Courchesne E (2000) Inverse correlation between frontal lobe and cerebellum sizes in children with autism. Brain : a journal of neurology 123 ( Pt 4):836-844.

Courchesne E, Saitoh O, Yeung-Courchesne R, Press GA, Lincoln AJ, Haas RH, Schreibman L (1994) Abnormality of cerebellar vermian lobules VI and VII in patients with infantile autism: identification of hypoplastic and hyperplastic subgroups with MR imaging. AJR American journal of roentgenology 162:123-130.

Coutinho AM, Oliveira G, Morgadinho T, Fesel C, Macedo TR, Bento C, Marques C, Ataide A, Miguel T, Borges L, Vicente AM (2004) Variants of the serotonin transporter gene (SLC6A4) significantly contribute to hyperserotonemia in autism. Molecular psychiatry 9:264-271.

Devlin B, Bennett P, Cook EH, Jr., Dawson G, Gonen D, Grigorenko EL, McMahon W, Pauls D, Smith M, Spence MA, Schellenberg GD (2002) No evidence for linkage of liability to autism to HOXA1 in a sample from the CPEA network. American journal of medical genetics 114:667-672.

Egaas B, Courchesne E, Saitoh O (1995) Reduced size of corpus callosum in autism. Archives of neurology 52:794-801.

Gernsbacher MA, Sauer EA, Geye HM, Schweigert EK, Hill Goldsmith H (2008) Infant and toddler oral- and manual-motor skills predict later speech fluency in autism. Journal of child psychology and psychiatry, and allied disciplines 49:43-50.

Grether JK, Anderson MC, Croen LA, Smith D, Windham GC (2009) Risk of autism and increasing maternal and paternal age in a large north American population. American journal of epidemiology 170:1118-1126.

Hardan AY, Kilpatrick M, Keshavan MS, Minshew NJ (2003) Motor performance and anatomic magnetic resonance imaging (MRI) of the basal ganglia in autism. Journal of child neurology 18:317-324.

Hisaoka S, Harada M, Nishitani H, Mori K (2001) Regional magnetic resonance spectroscopy of the brain in autistic individuals. Neuroradiology 43:496-498.

James SJ, Melnyk S, Jernigan S, Cleves MA, Halsted CH, Wong DH, Cutler P, Bock K, Boris M, Bradstreet JJ, Baker SM, Gaylor DW (2006) Metabolic endophenotype and related genotypes are associated with oxidative stress in children with autism. American journal of medical genetics Part B, Neuropsychiatric genetics : the official publication of the International Society of Psychiatric Genetics 141B:947-956.

Kleinhans NM, Richards T, Weaver KE, Liang O, Dawson G, Aylward E (2009) Brief report: biochemical correlates of clinical impairment in high functioning autism and Asperger's disorder. Journal of autism and developmental disorders 39:1079-1086.

Mori K (2010) [Psychopharmacological interventions in autism spectrum disorders]. No to hattatsu Brain and development 42:199-203.

Muscarella LA, Guarnieri V, Sacco R, Curatolo P, Manzi B, Alessandrelli R, Giana G, Militerni R, Bravaccio C, Lenti C, Saccani M, Schneider C, Melmed R, D'Agruma L, Persico AM (2010) Candidate gene study of HOXB1 in autism spectrum disorder. Molecular autism 1:9.

Oner O, Devrimci-Ozguven H, Oktem F, Yagmurlu B, Baskak B, Munir KM (2007) Proton MR spectroscopy: higher right anterior cingulate N-acetylaspartate/choline ratio in Asperger syndrome compared with healthy controls. AJNR American journal of neuroradiology 28:1494-1498.

Sparks BF, Friedman SD, Shaw DW, Aylward EH, Echelard D, Artru AA, Maravilla KR, Giedd JN, Munson J, Dawson G, Dager SR (2002) Brain structural abnormalities in young children with autism spectrum disorder. Neurology 59:184-192.

Tomchek SD, Dunn W (2007) Sensory processing in children with and without autism: a comparative study using the short sensory profile. The American journal of occupational therapy : official publication of the American Occupational Therapy Association 61:190-200.

Walker HA (1977) Incidence of minor physical anomaly in autism. Journal of autism and childhood schizophrenia 7:165-176.

**Major Depressive Disorder**

Almeida OP, McCaul K, Hankey GJ, Norman P, Jamrozik K, Flicker L (2008) Homocysteine and depression in later life. Archives of general psychiatry 65:1286-1294.

Baghai TC, Binder EB, Schule C, Salyakina D, Eser D, Lucae S, Zwanzger P, Haberger C, Zill P, Ising M, Deiml T, Uhr M, Illig T, Wichmann HE, Modell S, Nothdurfter C, Holsboer F, Muller-Myhsok B, Moller HJ, Rupprecht R, Bondy B (2006) Polymorphisms in the angiotensin-converting enzyme gene are associated with unipolar depression, ACE activity and hypercortisolism. Molecular psychiatry 11:1003-1015.

Blay SL, Andreoli SB, Fillenbaum GG, Gastal FL (2007) Depression morbidity in later life: prevalence and correlates in a developing country. The American journal of geriatric psychiatry : official journal of the American Association for Geriatric Psychiatry 15:790-799.

Eller T, Vasar V, Shlik J, Maron E (2008) Pro-inflammatory cytokines and treatment response to escitalopram in major depressive disorder. Progress in neuro-psychopharmacology & biological psychiatry 32:445-450.

Escribe-Aguir V, Gonzalez-Galarzo MC, Barona-Vilar C, Artazcoz L (2008) Factors related to depression during pregnancy: are there gender differences? Journal of epidemiology and community health 62:410-414.

Fan PL, Chen CD, Kao WT, Shu BC, Lung FW (2006) Protective effect of the apo epsilon2 allele in major depressive disorder in Taiwanese. Acta psychiatrica Scandinavica 113:48-53.

Frodl T, Jager M, Born C, Ritter S, Kraft E, Zetzsche T, Bottlender R, Leinsinger G, Reiser M, Moller HJ, Meisenzahl E (2008) Anterior cingulate cortex does not differ between patients with major depression and healthy controls, but relatively large anterior cingulate cortex predicts a good clinical course. Psychiatry research 163:76-83.

Frodl T, Meisenzahl EM, Zetzsche T, Born C, Jager M, Groll C, Bottlender R, Leinsinger G, Moller HJ (2003) Larger amygdala volumes in first depressive episode as compared to recurrent major depression and healthy control subjects. Biological psychiatry 53:338-344.

Gaysina D, Cohen S, Craddock N, Farmer A, Hoda F, Korszun A, Owen MJ, Craig IW, McGuffin P (2008) No association with the 5,10-methylenetetrahydrofolate reductase gene and major depressive disorder: results of the depression case control (DeCC) study and a meta-analysis. American journal of medical genetics Part B, Neuropsychiatric genetics : the official publication of the International Society of Psychiatric Genetics 147B:699-706.

Guze BH, Szuba MP (1992) Leukoencephalopathy and major depression: a preliminary report. Psychiatry research 45:169-175.

Haavind S, Bergin I, Brubakk AM (2007) [Mental health, school and leisure time of adolescents born "small for gestational age"]. Tidsskrift for den Norske laegeforening : tidsskrift for praktisk medicin, ny raekke 127:1933-1937.

Hale WW, 3rd (1998) Judgment of facial expressions and depression persistence. Psychiatry research 80:265-274.

Hannestad J, Taylor WD, McQuoid DR, Payne ME, Krishnan KR, Steffens DC, Macfall JR (2006) White matter lesion volumes and caudate volumes in late-life depression. International journal of geriatric psychiatry 21:1193-1198.

Hariri AR, Drabant EM, Munoz KE, Kolachana BS, Mattay VS, Egan MF, Weinberger DR (2005) A susceptibility gene for affective disorders and the response of the human amygdala. Archives of general psychiatry 62:146-152.

Hettema JM, An SS, van den Oord EJ, Neale MC, Kendler KS, Chen X (2008) Association study between the serotonin 1A receptor (HTR1A) gene and neuroticism, major depression, and anxiety disorders. American journal of medical genetics Part B, Neuropsychiatric genetics : the official publication of the International Society of Psychiatric Genetics 147B:661-666.

Hong ED, Taylor WD, McQuoid DR, Potter GG, Payne ME, Ashley-Koch A, Steffens DC (2009) Influence of the MTHFR C677T polymorphism on magnetic resonance imaging hyperintensity volume and cognition in geriatric depression. The American journal of geriatric psychiatry : official journal of the American Association for Geriatric Psychiatry 17:847-855.

Huang TL, Lee CT, Liu YL (2008) Serum brain-derived neurotrophic factor levels in patients with major depression: effects of antidepressants. Journal of psychiatric research 42:521-525.

MacMaster FP, Russell A, Mirza Y, Keshavan MS, Taormina SP, Bhandari R, Boyd C, Lynch M, Rose M, Ivey J, Moore GJ, Rosenberg DR (2006) Pituitary volume in treatment-naive pediatric major depressive disorder. Biological psychiatry 60:862-866.

Maes a M, Meltzer HY, Bosmans E, Bergmans R, Vandoolaeghe E, Ranjan R, Desnyder R (1995) Increased plasma concentrations of interleukin-6, soluble interleukin-6, soluble interleukin-2 and transferrin receptor in major depression. Journal of affective disorders 34:301-309.

Meisenzahl EM, Seifert D, Bottlender R, Teipel S, Zetzsche T, Jager M, Koutsouleris N, Schmitt G, Scheuerecker J, Burgermeister B, Hampel H, Rupprecht T, Born C, Reiser M, Moller HJ, Frodl T (2010) Differences in hippocampal volume between major depression and schizophrenia: a comparative neuroimaging study. European archives of psychiatry and clinical neuroscience 260:127-137.

Mezulis AH, Hyde JS, Clark R (2004) Father involvement moderates the effect of maternal depression during a child's infancy on child behavior problems in kindergarten. Journal of family psychology : JFP : journal of the Division of Family Psychology of the American Psychological Association (Division 43) 18:575-588.

Mufson L, Weissman MM, Warner V (1992) Depression and anxiety in parents and children: A direct interview study. Journal of Anxiety Disorders 6:1-13.

Nery FG, Stanley JA, Chen HH, Hatch JP, Nicoletti MA, Monkul ES, Matsuo K, Caetano SC, Peluso MA, Najt P, Soares JC (2009) Normal metabolite levels in the left dorsolateral prefrontal cortex of unmedicated major depressive disorder patients: a single voxel (1)H spectroscopy study. Psychiatry research 174:177-183.

Pan CC, McQuoid DR, Taylor WD, Payne ME, Ashley-Koch A, Steffens DC (2009) Association analysis of the COMT/MTHFR genes and geriatric depression: an MRI study of the putamen. International journal of geriatric psychiatry 24:847-855.

Parashos IA, Tupler LA, Blitchington T, Krishnan KR (1998) Magnetic-resonance morphometry in patients with major depression. Psychiatry research 84:7-15.

Parissis JT, Farmakis D, Nikolaou M, Birmpa D, Bistola V, Paraskevaidis I, Ikonomidis I, Gaitani S, Venetsanou K, Filippatos G, Kremastinos DT (2009) Plasma B-type natriuretic peptide and anti-inflammatory cytokine interleukin-10 levels predict adverse clinical outcome in chronic heart failure patients with depressive symptoms: a 1-year follow-up study. European journal of heart failure 11:967-972.

Pujol J, Cardoner N, Benlloch L, Urretavizcaya M, Deus J, Losilla JM, Capdevila A, Vallejo J (2002) CSF spaces of the Sylvian fissure region in severe melancholic depression. NeuroImage 15:103-106.

Ramchandani PG, Stein A, O'Connor TG, Heron J, Murray L, Evans J (2008) Depression in men in the postnatal period and later child psychopathology: a population cohort study. Journal of the American Academy of Child and Adolescent Psychiatry 47:390-398.

Rottenberg J, Kasch KL, Gross JJ, Gotlib IH (2002) Sadness and amusement reactivity differentially predict concurrent and prospective functioning in major depressive disorder. Emotion (Washington, DC) 2:135-146.

Schoevers RA, Beekman AT, Deeg DJ, Geerlings MI, Jonker C, Van Tilburg W (2000) Risk factors for depression in later life; results of a prospective community based study (AMSTEL). Journal of affective disorders 59:127-137.

Schumacher J, Jamra RA, Becker T, Ohlraun S, Klopp N, Binder EB, Schulze TG, Deschner M, Schmal C, Hofels S, Zobel A, Illig T, Propping P, Holsboer F, Rietschel M, Nothen MM, Cichon S (2005) Evidence for a relationship between genetic variants at the brain-derived neurotrophic factor (BDNF) locus and major depression. Biological psychiatry 58:307-314.

Serrano JP, Latorre JM, Gatz M (2007

) Autobiographical memory in older adults with and without depressive symptoms. International Journal of Clinical and Health Psychology 7:41-57.

Simon NM, McNamara K, Chow CW, Maser RS, Papakostas GI, Pollack MH, Nierenberg AA, Fava M, Wong KK (2008) A detailed examination of cytokine abnormalities in Major Depressive Disorder. European neuropsychopharmacology : the journal of the European College of Neuropsychopharmacology 18:230-233.

Spinhoven P, Bockting CL, Schene AH, Koeter MW, Wekking EM, Williams JM (2006) Autobiographical memory in the euthymic phase of recurrent depression. Journal of abnormal psychology 115:590-600.

Steingard R, Biederman J, Keenan K, Moore C (1990) Comorbidity in the interpretation of dexamethasone suppression test results in children: a review and report. Biological psychiatry 28:193-202.

Stordal KI, Lundervold AJ, Egeland J, Mykletun A, Asbjornsen A, Landro NI, Roness A, Rund BR, Sundet K, Oedegaard KJ, Lund A (2004) Impairment across executive functions in recurrent major depression. Nordic journal of psychiatry 58:41-47.

Surtees PG, Wainwright NW, Willis-Owen SA, Luben R, Day NE, Flint J (2006) Social adversity, the serotonin transporter (5-HTTLPR) polymorphism and major depressive disorder. Biological psychiatry 59:224-229.

Taylor WD, Macfall JR, Payne ME, McQuoid DR, Steffens DC, Provenzale JM, Krishnan KR (2007) Orbitofrontal cortex volume in late life depression: influence of hyperintense lesions and genetic polymorphisms. Psychological medicine 37:1763-1773.

Taylor WD, Steffens DC, Payne ME, MacFall JR, Marchuk DA, Svenson IK, Krishnan KR (2005) Influence of serotonin transporter promoter region polymorphisms on hippocampal volumes in late-life depression. Archives of general psychiatry 62:537-544.

Tupler LA, Krishnan KR, McDonald WM, Dombeck CB, D'Souza S, Steffens DC (2002) Anatomic location and laterality of MRI signal hyperintensities in late-life depression. Journal of psychosomatic research 53:665-676.

Vreeburg SA, Hoogendijk WJ, van Pelt J, Derijk RH, Verhagen JC, van Dyck R, Smit JH, Zitman FG, Penninx BW (2009) Major depressive disorder and hypothalamic-pituitary-adrenal axis activity: results from a large cohort study. Archives of general psychiatry 66:617-626.

Willis-Owen SA, Turri MG, Munafo MR, Surtees PG, Wainwright NW, Brixey RD, Flint J (2005) The serotonin transporter length polymorphism, neuroticism, and depression: a comprehensive assessment of association. Biological psychiatry 58:451-456.

**Schizophrenia**

Akyol O, Herken H, Uz E, Fadillioglu E, Unal S, Sogut S, Ozyurt H, Savas HA (2002) The indices of endogenous oxidative and antioxidative processes in plasma from schizophrenic patients. The possible role of oxidant/antioxidant imbalance. Progress in neuro-psychopharmacology & biological psychiatry 26:995-1005.

Albee GW, Lane EA, Reuter JM (1964) CHILDHOOD INTELLIGENCE OF FUTURE SCHIZOPHRENICS AND NEIGHBORHOOD PEERS. The Journal of psychology 58:141-144.

Auer DP, Wilke M, Grabner A, Heidenreich JO, Bronisch T, Wetter TC (2001) Reduced NAA in the thalamus and altered membrane and glial metabolism in schizophrenic patients detected by 1H-MRS and tissue segmentation. Schizophrenia research 52:87-99.

Baiano M, Perlini C, Rambaldelli G, Cerini R, Dusi N, Bellani M, Spezzapria G, Versace A, Balestrieri M, Mucelli RP, Tansella M, Brambilla P (2008) Decreased entorhinal cortex volumes in schizophrenia. Schizophrenia research 102:171-180.

Bentsen H, Solberg DK, Refsum H, Gran JM, Bohmer T, Torjesen PA, Halvorsen O, Lingjaerde O (2011) Bimodal distribution of polyunsaturated fatty acids in schizophrenia suggests two endophenotypes of the disorder. Biological psychiatry 70:97-105.

Bora E, Gokcen S, Kayahan B, Veznedaroglu B (2008) Deficits of social-cognitive and social-perceptual aspects of theory of mind in remitted patients with schizophrenia: effect of residual symptoms. The Journal of nervous and mental disease 196:95-99.

Bozikas VP, Andreou C, Giannakou M, Tonia T, Anezoulaki D, Karavatos A, Fokas K, Kosmidis MH (2005) Deficits in sustained attention in schizophrenia but not in bipolar disorder. Schizophrenia research 78:225-233.

Brown AS, Schaefer CA, Quesenberry CP, Jr., Shen L, Susser ES (2006) No evidence of relation between maternal exposure to herpes simplex virus type 2 and risk of schizophrenia? The American journal of psychiatry 163:2178-2180.

Brown KJ, Gonsalvez CJ, Harris AW, Williams LM, Gordon E (2002) Target and non-target ERP disturbances in first episode vs. chronic schizophrenia. Clinical neurophysiology : official journal of the International Federation of Clinical Neurophysiology 113:1754-1763.

Cantor-Graae E, Pedersen CB (2007) Risk of schizophrenia in second-generation immigrants: a Danish population-based cohort study. Psychological medicine 37:485-494.

Chen EY, Wong AW, Chen RY, Au JW (2001) Stroop interference and facilitation effects in first-episode schizophrenic patients. Schizophrenia research 48:29-44.

Crespo-Facorro B, Roiz-Santianez R, Pelayo-Teran JM, Gonzalez-Blanch C, Perez-Iglesias R, Gutierrez A, de Lucas EM, Tordesillas D, Vazquez-Barquero JL (2007) Caudate nucleus volume and its clinical and cognitive correlations in first episode schizophrenia. Schizophrenia research 91:87-96.

Crespo-Facorro B, Roiz-Santianez R, Perez-Iglesias R, Tordesillas-Gutierrez D, Mata I, Rodriguez-Sanchez JM, de Lucas EM, Vazquez-Barquero JL (2009) Specific brain structural abnormalities in first-episode schizophrenia. A comparative study with patients with schizophreniform disorder, non-schizophrenic non-affective psychoses and healthy volunteers. Schizophrenia research 115:191-201.

Crespo-Facorro B, Roiz-Santianez R, Quintero C, Perez-Iglesias R, Tordesillas-Gutierrez D, Mata I, Rodriguez-Sanchez JM, Gutierrez A, Vazquez-Barquero JL (2010) Insular cortex morphometry in first-episode schizophrenia-spectrum patients: Diagnostic specificity and clinical correlations. Journal of psychiatric research 44:314-320.

Cuesta MJ, Peralta V, Zarzuela A (2007) Empirical validation of competing definitions of schizophrenia: a poly-diagnostic study of cognitive impairment in non-affective psychosis. Schizophrenia research 95:39-47.

Deicken RF, Zhou L, Schuff N, Fein G, Weiner MW (1998) Hippocampal neuronal dysfunction in schizophrenia as measured by proton magnetic resonance spectroscopy. Biological psychiatry 43:483-488.

Depp CA, Moore DJ, Sitzer D, Palmer BW, Eyler LT, Roesch S, Lebowitz BD, Jeste DV (2007) Neurocognitive impairment in middle-aged and older adults with bipolar disorder: comparison to schizophrenia and normal comparison subjects. Journal of affective disorders 101:201-209.

Dickinson D, Iannone VN, Wilk CM, Gold JM (2004) General and specific cognitive deficits in schizophrenia. Biological psychiatry 55:826-833.

Egan MF, Hyde TM, Bonomo JB, Mattay VS, Bigelow LB, Goldberg TE, Weinberger DR (2001) Relative risk of neurological signs in siblings of patients with schizophrenia. The American journal of psychiatry 158:1827-1834.

Elkashef AM, Doudet D, Bryant T, Cohen RM, Li SH, Wyatt RJ (2000) 6-(18)F-DOPA PET study in patients with schizophrenia. Positron emission tomography. Psychiatry research 100:1-11.

Fan H, Zhang F, Xu Y, Huang X, Sun G, Song Y, Long H, Liu P (2010) An association study of DRD2 gene polymorphisms with schizophrenia in a Chinese Han population. Neuroscience letters 477:53-56.

Fisher M, McCoy K, Poole JH, Vinogradov S (2008) Self and other in schizophrenia: a cognitive neuroscience perspective. The American journal of psychiatry 165:1465-1472.

Friedman JI, Tang C, Carpenter D, Buchsbaum M, Schmeidler J, Flanagan L, Golembo S, Kanellopoulou I, Ng J, Hof PR, Harvey PD, Tsopelas ND, Stewart D, Davis KL (2008) Diffusion tensor imaging findings in first-episode and chronic schizophrenia patients. The American journal of psychiatry 165:1024-1032.

Frith CD, Leary J, Cahill C, Johnstone EC (1991) Performance on psychological tests. Demographic and clinical correlates of the results of these tests. The British journal of psychiatry Supplement 26-29, 44-26.

Galinska B, Szulc A, Tarasow E, Kubas B, Dzienis W, Czernikiewicz A, Walecki J (2009) Duration of untreated psychosis and proton magnetic resonance spectroscopy (1H-MRS) findings in first-episode schizophrenia. Medical science monitor : international medical journal of experimental and clinical research 15:CR82-88.

Glaser B, Schumacher J, Williams HJ, Jamra RA, Ianakiev N, Milev R, Ohlraun S, Schulze TG, Czerski PM, Hauser J, Jonsson EG, Sedvall GC, Klopp N, Illig T, Becker T, Propping P, Williams NM, Cichon S, Kirov G, Rietschel M, Murphy KC, O'Donovan MC, Nothen MM, Owen MJ (2005) No association between the putative functional ZDHHC8 single nucleotide polymorphism rs175174 and schizophrenia in large European samples. Biological psychiatry 58:78-80.

Good KP, Martzke JS, Honer WG, Kopala LC (1998) Left nostril olfactory identification impairment in a subgroup of male patients with schizophrenia. Schizophrenia research 33:35-43.

Green MF, Bearden CE, Cannon TD, Fiske AP, Hellemann GS, Horan WP, Kee K, Kern RS, Lee J, Sergi MJ, Subotnik KL, Sugar CA, Ventura J, Yee CM, Nuechterlein KH (2012) Social cognition in schizophrenia, Part 1: performance across phase of illness. Schizophrenia bulletin 38:854-864.

Gur RE, Turetsky BI, Bilker WB, Gur RC (1999) Reduced gray matter volume in schizophrenia. Archives of general psychiatry 56:905-911.

Haack M, Hinze-Selch D, Fenzel T, Kraus T, Kuhn M, Schuld A, Pollmacher T (1999) Plasma levels of cytokines and soluble cytokine receptors in psychiatric patients upon hospital admission: effects of confounding factors and diagnosis. Journal of psychiatric research 33:407-418.

Habel U, Gur RC, Mandal MK, Salloum JB, Gur RE, Schneider F (2000) Emotional processing in schizophrenia across cultures: standardized measures of discrimination and experience. Schizophrenia research 42:57-66.

Ho BC, Wassink TH, O'Leary DS, Sheffield VC, Andreasen NC (2005) Catechol-O-methyl transferase Val158Met gene polymorphism in schizophrenia: working memory, frontal lobe MRI morphology and frontal cerebral blood flow. Molecular psychiatry 10:229, 287-298.

Hoff AL, Wieneke M, Faustman WO, Horon R, Sakuma M, Blankfeld H, Espinoza S, DeLisi LE (1998) Sex differences in neuropsychological functioning of first-episode and chronically ill schizophrenic patients. The American journal of psychiatry 155:1437-1439.

Holmen A, Juuhl-Langseth M, Thormodsen R, Melle I, Rund BR (2010) Neuropsychological profile in early-onset schizophrenia-spectrum disorders: measured with the MATRICS battery. Schizophrenia bulletin 36:852-859.

Hulshoff Pol HE, Schnack HG, Bertens MG, van Haren NE, van der Tweel I, Staal WG, Baare WF, Kahn RS (2002) Volume changes in gray matter in patients with schizophrenia. The American journal of psychiatry 159:244-250.

Jabben N, Arts B, van Os J, Krabbendam L (2010) Neurocognitive functioning as intermediary phenotype and predictor of psychosocial functioning across the psychosis continuum: studies in schizophrenia and bipolar disorder. The Journal of clinical psychiatry 71:764-774.

Jamra RA, Becker T, Klopp N, Dahdouh F, Schulze TG, Gross M, Deschner M, Schmal C, Illig T, Rietschel M, Propping P, Cichon S, Nothen MM, Schumacher J (2007) No evidence for an association between variants at the gamma-amino-n-butyric acid type A receptor beta2 locus and schizophrenia. Psychiatric genetics 17:43-45.

Joo EJ, Jeong SH, Ahn YM, Lee KY, Chang Yoon S, Kim EJ, Kim SU, Cho SC, Sik Kim Y (2005) No association found between 158 Val/Met polymorphism of the COMT gene and schizophrenia with minor physical anomalies. Psychiatry research 136:83-91.

Joyce E, Hutton S, Mutsatsa S, Gibbins H, Webb E, Paul S, Robbins T, Barnes T (2002) Executive dysfunction in first-episode schizophrenia and relationship to duration of untreated psychosis: the West London Study. The British journal of psychiatry Supplement 43:s38-44.

Kaiser R, Tremblay PB, Schmider J, Henneken M, Dettling M, Muller-Oerlinghausen B, Uebelhack R, Roots I, Brockmoller J (2001) Serotonin transporter polymorphisms: no association with response to antipsychotic treatment, but associations with the schizoparanoid and residual subtypes of schizophrenia. Molecular psychiatry 6:179-185.

Kawashima K, Ikeda M, Kishi T, Kitajima T, Yamanouchi Y, Kinoshita Y, Okochi T, Aleksic B, Tomita M, Okada T, Kunugi H, Inada T, Ozaki N, Iwata N (2009) BDNF is not associated with schizophrenia: data from a Japanese population study and meta-analysis. Schizophrenia research 112:72-79.

Kemali D, Polani N, Polani PE, Amati A (1976) A dermatoglyphic study of 219 Italian schizophrenic males. Clinical genetics 9:51-60.

Kern RS, Gold JM, Dickinson D, Green MF, Nuechterlein KH, Baade LE, Keefe RS, Mesholam-Gately RI, Seidman LJ, Lee C, Sugar CA, Marder SR (2011) The MCCB impairment profile for schizophrenia outpatients: results from the MATRICS psychometric and standardization study. Schizophrenia research 126:124-131.

Koolschijn PC, van Haren NE, Cahn W, Schnack HG, Janssen J, Klumpers F, Hulshoff Pol HE, Kahn RS (2010) Hippocampal volume change in schizophrenia. The Journal of clinical psychiatry 71:737-744.

Kopala LC, Good KP, Honer WG (1994) Olfactory hallucinations and olfactory identification ability in patients with schizophrenia and other psychiatric disorders. Schizophrenia research 12:205-211.

Kostova M, Passerieux C, Laurent JP, Hardy-Bayle MC (2005) N400 anomalies in schizophrenia are correlated with the severity of formal thought disorder. Schizophrenia research 78:285-291.

Kucharska-Pietura K, David AS, Masiak M, Phillips ML (2005) Perception of facial and vocal affect by people with schizophrenia in early and late stages of illness. The British journal of psychiatry : the journal of mental science 187:523-528.

Kudoh A, Ishihara H, Matsuki A (2000) Current perception thresholds and postoperative pain in schizophrenic patients. Regional anesthesia and pain medicine 25:475-479.

Laursen TM, Munk-Olsen T, Nordentoft M, Bo Mortensen P (2007) A comparison of selected risk factors for unipolar depressive disorder, bipolar affective disorder, schizoaffective disorder, and schizophrenia from a danish population-based cohort. The Journal of clinical psychiatry 68:1673-1681.

Lavalaye J, Linszen DH, Booij J, Dingemans PM, Reneman L, Habraken JB, Gersons BP, van Royen EA (2001) Dopamine transporter density in young patients with schizophrenia assessed with [123]FP-CIT SPECT. Schizophrenia research 47:59-67.

Lawyer G, Nesvag R, Varnas K, Frigessi A, Agartz I (2008) Investigating possible subtypes of schizophrenia patients and controls based on brain cortical thickness. Psychiatry research 164:254-264.

Leeson VC, McKenna PJ, Murray G, Kondel TK, Laws KR (2005) What happens to semantic memory when formal thought disorder remits? Revisiting a case study. Cognitive neuropsychiatry 10:57-71.

Lung FW, Chen N, Shu BC (2006) Dopamine D4 receptor gene and the -521C>T polymorphism of the upstream region of the dopamine D4 receptor gene in schizophrenia. Psychiatric genetics 16:139-143.

Mamah D, Wang L, Barch D, de Erausquin GA, Gado M, Csernansky JG (2007) Structural analysis of the basal ganglia in schizophrenia. Schizophrenia research 89:59-71.

Martin Reyes M, Mendoza Quinonez R, Diaz de Villalvilla T, Lomba P, Padron Fernando A, Valdes Sosa M (2004) [Family transmission of positive and negative symptoms in familial and sporadic schizophrenia]. Actas espanolas de psiquiatria 32:353-357.

Martorell L, Virgos C, Valero J, Coll G, Figuera L, Joven J, Pocovi M, Labad A, Vilella E (2001) Schizophrenic women with the APOE epsilon 4 allele have a worse prognosis than those without it. Molecular psychiatry 6:307-310.

Mathalon DH, Pfefferbaum A, Lim KO, Rosenbloom MJ, Sullivan EV (2003) Compounded brain volume deficits in schizophrenia-alcoholism comorbidity. Archives of general psychiatry 60:245-252.

McCabe KL, Maloney EA, Stain HJ, Loughland CM, Carr VJ (2012) Relationship between childhood adversity and clinical and cognitive features in schizophrenia. Journal of psychiatric research 46:600-607.

Monakhov M, Golimbet V, Abramova L, Kaleda V, Karpov V (2008) Association study of three polymorphisms in the dopamine D2 receptor gene and schizophrenia in the Russian population. Schizophrenia research 100:302-307.

Mortensen PB, Norgaard-Pedersen B, Waltoft BL, Sorensen TL, Hougaard D, Torrey EF, Yolken RH (2007) Toxoplasma gondii as a risk factor for early-onset schizophrenia: analysis of filter paper blood samples obtained at birth. Biological psychiatry 61:688-693.

Muir WJ, St Clair DM, Blackwood DH (1991) Long-latency auditory event-related potentials in schizophrenia and in bipolar and unipolar affective disorder. Psychological medicine 21:867-879.

Muntjewerff JW, Ophoff RA, Buizer-Voskamp JE, Strengman E, den Heijer M (2011) Effects of season of birth and a common MTHFR gene variant on the risk of schizophrenia. European neuropsychopharmacology : the journal of the European College of Neuropsychopharmacology 21:300-305.

Nielsen AS, Mortensen PB, O'Callaghan E, Mors O, Ewald H (2002) Is head injury a risk factor for schizophrenia? Schizophrenia research 55:93-98.

Norton N, Williams HJ, Dwyer S, Carroll L, Peirce T, Moskvina V, Segurado R, Nikolov I, Williams NM, Ikeda M, Iwata N, Owen MJ, O'Donovan MC (2007) Association analysis of AKT1 and schizophrenia in a UK case control sample. Schizophrenia research 93:58-65.

O'Donovan MC, Craddock N, Norton N, Williams H, Peirce T, Moskvina V, Nikolov I, Hamshere M, Carroll L, Georgieva L, Dwyer S, Holmans P, Marchini JL, Spencer CC, Howie B, Leung HT, Hartmann AM, Moller HJ, Morris DW, Shi Y, Feng G, Hoffmann P, Propping P, Vasilescu C, Maier W, Rietschel M, Zammit S, Schumacher J, Quinn EM, Schulze TG, Williams NM, Giegling I, Iwata N, Ikeda M, Darvasi A, Shifman S, He L, Duan J, Sanders AR, Levinson DF, Gejman PV, Cichon S, Nothen MM, Gill M, Corvin A, Rujescu D, Kirov G, Owen MJ, Buccola NG, Mowry BJ, Freedman R, Amin F, Black DW, Silverman JM, Byerley WF, Cloninger CR (2008) Identification of loci associated with schizophrenia by genome-wide association and follow-up. Nature genetics 40:1053-1055.

Ohi K, Hashimoto R, Yasuda Y, Yoshida T, Takahashi H, Iike N, Iwase M, Kamino K, Ishii R, Kazui H, Fukumoto M, Takamura H, Yamamori H, Azechi M, Ikezawa K, Tanimukai H, Tagami S, Morihara T, Okochi M, Yamada K, Numata S, Ikeda M, Tanaka T, Kudo T, Ueno S, Yoshikawa T, Ohmori T, Iwata N, Ozaki N, Takeda M (2010) The chitinase 3-like 1 gene and schizophrenia: evidence from a multi-center case-control study and meta-analysis. Schizophrenia research 116:126-132.

Ohrmann P, Kugel H, Bauer J, Siegmund A, Kolkebeck K, Suslow T, Wiedl KH, Rothermundt M, Arolt V, Pedersen A (2008) Learning potential on the WCST in schizophrenia is related to the neuronal integrity of the anterior cingulate cortex as measured by proton magnetic resonance spectroscopy. Schizophrenia research 106:156-163.

Okugawa G, Tamagaki C, Agartz I (2007) Frontal and temporal volume size of grey and white matter in patients with schizophrenia: an MRI parcellation study. European archives of psychiatry and clinical neuroscience 257:304-307.

Palaniyappan L, Mallikarjun P, Joseph V, White TP, Liddle PF (2011) Reality distortion is related to the structure of the salience network in schizophrenia. Psychological medicine 41:1701-1708.

Perlstein WM, Carter CS, Barch DM, Baird JW (1998) The Stroop task and attention deficits in schizophrenia: a critical evaluation of card and single-trial Stroop methodologies. Neuropsychology 12:414-425.

Petryshen TL, Middleton FA, Tahl AR, Rockwell GN, Purcell S, Aldinger KA, Kirby A, Morley CP, McGann L, Gentile KL, Waggoner SG, Medeiros HM, Carvalho C, Macedo A, Albus M, Maier W, Trixler M, Eichhammer P, Schwab SG, Wildenauer DB, Azevedo MH, Pato MT, Pato CN, Daly MJ, Sklar P (2005) Genetic investigation of chromosome 5q GABAA receptor subunit genes in schizophrenia. Molecular psychiatry 10:1074-1088, 1057.

Pousa E, Duno R, Brebion G, David AS, Ruiz AI, Obiols JE (2008) Theory of mind deficits in chronic schizophrenia: evidence for state dependence. Psychiatry research 158:1-10.

Premkumar P, Kumari V, Corr PJ, Fannon D, Sharma T (2008) Neuropsychological function-brain structure relationships and stage of illness: an investigation into chronic and first-episode schizophrenia. Psychiatry research 162:195-204.

Rhinewine JP, Lencz T, Thaden EP, Cervellione KL, Burdick KE, Henderson I, Bhaskar S, Keehlisen L, Kane J, Kohn N, Fisch GS, Bilder RM, Kumra S (2005) Neurocognitive profile in adolescents with early-onset schizophrenia: clinical correlates. Biological psychiatry 58:705-712.

Rosa PG, Schaufelberger MS, Uchida RR, Duran FL, Lappin JM, Menezes PR, Scazufca M, McGuire PK, Murray RM, Busatto GF (2010) Lateral ventricle differences between first-episode schizophrenia and first-episode psychotic bipolar disorder: A population-based morphometric MRI study. The world journal of biological psychiatry : the official journal of the World Federation of Societies of Biological Psychiatry 11:873-887.

Rossell SL, David AS (2006) Are semantic deficits in schizophrenia due to problems with access or storage? Schizophrenia research 82:121-134.

Rothermundt M, Ponath G, Glaser T, Hetzel G, Arolt V (2004) S100B serum levels and long-term improvement of negative symptoms in patients with schizophrenia. Neuropsychopharmacology : official publication of the American College of Neuropsychopharmacology 29:1004-1011.

Saetre P, Lundmark P, Wang A, Hansen T, Rasmussen HB, Djurovic S, Melle I, Andreassen OA, Werge T, Agartz I, Hall H, Terenius L, Jonsson EG (2010) The tryptophan hydroxylase 1 (TPH1) gene, schizophrenia susceptibility, and suicidal behavior: a multi-centre case-control study and meta-analysis. American journal of medical genetics Part B, Neuropsychiatric genetics : the official publication of the International Society of Psychiatric Genetics 153B:387-396.

Sanders AR, Duan J, Levinson DF, Shi J, He D, Hou C, Burrell GJ, Rice JP, Nertney DA, Olincy A, Rozic P, Vinogradov S, Buccola NG, Mowry BJ, Freedman R, Amin F, Black DW, Silverman JM, Byerley WF, Crowe RR, Cloninger CR, Martinez M, Gejman PV (2008) No significant association of 14 candidate genes with schizophrenia in a large European ancestry sample: implications for psychiatric genetics. The American journal of psychiatry 165:497-506.

Schumacher J, Laje G, Abou Jamra R, Becker T, Muhleisen TW, Vasilescu C, Mattheisen M, Herms S, Hoffmann P, Hillmer AM, Georgi A, Herold C, Schulze TG, Propping P, Rietschel M, McMahon FJ, Nothen MM, Cichon S (2009) The DISC locus and schizophrenia: evidence from an association study in a central European sample and from a meta-analysis across different European populations. Human molecular genetics 18:2719-2727.

Selten JP, Slaets JP, Kahn RS (1997) Schizophrenia in Surinamese and Dutch Antillean immigrants to The Netherlands: evidence of an increased incidence. Psychological medicine 27:807-811.

Shapleske J, Rossell SL, Simmons A, David AS, Woodruff PW (2001) Are auditory hallucinations the consequence of abnormal cerebral lateralization? A morphometric MRI study of the sylvian fissure and planum temporale. Biological psychiatry 49:685-693.

Shimizu A, Kurachi M, Yamaguchi N, Torii H, Isaki K (1988) Does family history of schizophrenia influence age at onset of schizophrenia? Acta psychiatrica Scandinavica 78:716-719.

Shintani F, Kanba S, Maruo N, Nakaki T, Nibuya M, Suzuki E, Kinoshita N, Yagi G (1991) Serum interleukin-6 in schizophrenic patients. Life sciences 49:661-664.

Shirts BH, Wood J, Yolken RH, Nimgaonkar VL (2006) Association study of IL10, IL1beta, and IL1RN and schizophrenia using tag SNPs from a comprehensive database: suggestive association with rs16944 at IL1beta. Schizophrenia research 88:235-244.

Spitzer M, Weisker I, Winter M, Maier S, Hermle L, Maher BA (1994) Semantic and phonological priming in schizophrenia. Journal of abnormal psychology 103:485-494.

Stevens A, Schwarz J, Schwarz B, Ruf I, Kolter T, Czekalla J (2002) Implicit and explicit learning in schizophrenics treated with olanzapine and with classic neuroleptics. Psychopharmacology 160:299-306.

Szeszko PR, Goldberg E, Gunduz-Bruce H, Ashtari M, Robinson D, Malhotra AK, Lencz T, Bates J, Crandall DT, Kane JM, Bilder RM (2003) Smaller anterior hippocampal formation volume in antipsychotic-naive patients with first-episode schizophrenia. The American journal of psychiatry 160:2190-2197.

Takahashi T, Suzuki M, Zhou SY, Tanino R, Hagino H, Kawasaki Y, Matsui M, Seto H, Kurachi M (2006) Morphologic alterations of the parcellated superior temporal gyrus in schizophrenia spectrum. Schizophrenia research 83:131-143.

Takahashi T, Yucel M, Yung AR, Wood SJ, Phillips LJ, Berger GE, Ang A, Soulsby B, McGorry PD, Suzuki M, Velakoulis D, Pantelis C (2008) Adhesio interthalamica in individuals at high-risk for developing psychosis and patients with psychotic disorders. Progress in neuro-psychopharmacology & biological psychiatry 32:1708-1714.

Tayoshi S, Sumitani S, Taniguchi K, Shibuya-Tayoshi S, Numata S, Iga J, Nakataki M, Ueno S, Harada M, Ohmori T (2009) Metabolite changes and gender differences in schizophrenia using 3-Tesla proton magnetic resonance spectroscopy (1H-MRS). Schizophrenia research 108:69-77.

Tomasino B, Bellani M, Perlini C, Rambaldelli G, Cerini R, Isola M, Balestrieri M, Cali S, Versace A, Pozzi Mucelli R, Gasparini A, Tansella M, Brambilla P (2011) Altered microstructure integrity of the amygdala in schizophrenia: a bimodal MRI and DWI study. Psychological medicine 41:301-311.

Torrey EF, Yolken RH, Winfrey CJ (1982) Cytomegalovirus antibody in cerebrospinal fluid of schizophrenic patients detected by enzyme immunoassay. Science (New York, NY) 216:892-894.

Tune LE, Wong DF, Pearlson G, Strauss M, Young T, Shaya EK, Dannals RF, Wilson AA, Ravert HT, Sapp J, et al. (1993) Dopamine D2 receptor density estimates in schizophrenia: a positron emission tomography study with 11C-N-methylspiperone. Psychiatry research 49:219-237.

Turek S (1990) Dermatoglyphics and schizophrenia—analysis of quantitative traits. Collegium Antropologicum 14:137-150.

Utsunomiya K, Shinkai T, De Luca V, Hwang R, Sakata S, Fukunaka Y, Chen HI, Ohmori O, Nakamura J (2008) Genetic association between the dopamine D3 gene polymorphism (Ser9Gly) and schizophrenia in Japanese populations: evidence from a case-control study and meta-analysis. Neuroscience letters 444:161-165.

van Haren NE, Hulshoff Pol HE, Schnack HG, Cahn W, Brans R, Carati I, Rais M, Kahn RS (2008) Progressive brain volume loss in schizophrenia over the course of the illness: evidence of maturational abnormalities in early adulthood. Biological psychiatry 63:106-113.

Vilella E, Costas J, Sanjuan J, Guitart M, De Diego Y, Carracedo A, Martorell L, Valero J, Labad A, De Frutos R, Najera C, Molto MD, Toirac I, Guillamat R, Brunet A, Valles V, Perez L, Leon M, de Fonseca FR, Phillips C, Torres M (2008) Association of schizophrenia with DTNBP1 but not with DAO, DAOA, NRG1 and RGS4 nor their genetic interaction. Journal of psychiatric research 42:278-288.

Wang L, Hosakere M, Trein JC, Miller A, Ratnanather JT, Barch DM, Thompson PA, Qiu A, Gado MH, Miller MI, Csernansky JG (2007) Abnormalities of cingulate gyrus neuroanatomy in schizophrenia. Schizophrenia research 93:66-78.

Watanabe Y, Nunokawa A, Kaneko N, Shibuya M, Egawa J, Someya T (2012) Supportive evidence for the association between the Gln2Pro polymorphism in the SIGMAR1 gene and schizophrenia in the Japanese population: a case-control study and an updated meta-analysis. Schizophrenia research 141:279-280.

Wilk CM, Gold JM, Bartko JJ, Dickerson F, Fenton WS, Knable M, Randolph C, Buchanan RW (2002) Test-retest stability of the Repeatable Battery for the Assessment of Neuropsychological Status in schizophrenia. The American journal of psychiatry 159:838-844.

Williams NM, Green EK, Macgregor S, Dwyer S, Norton N, Williams H, Raybould R, Grozeva D, Hamshere M, Zammit S, Jones L, Cardno A, Kirov G, Jones I, O'Donovan MC, Owen MJ, Craddock N (2006) Variation at the DAOA/G30 locus influences susceptibility to major mood episodes but not psychosis in schizophrenia and bipolar disorder. Archives of general psychiatry 63:366-373.

Williams NM, Preece A, Spurlock G, Norton N, Williams HJ, Zammit S, O'Donovan MC, Owen MJ (2003) Support for genetic variation in neuregulin 1 and susceptibility to schizophrenia. Molecular psychiatry 8:485-487.

Xiu MH, Hui L, Dang YF, Hou TD, Zhang CX, Zheng YL, Chen da C, Kosten TR, Zhang XY (2009) Decreased serum BDNF levels in chronic institutionalized schizophrenia on long-term treatment with typical and atypical antipsychotics. Progress in neuro-psychopharmacology & biological psychiatry 33:1508-1512.

Yamada K, Ohnishi T, Hashimoto K, Ohba H, Iwayama-Shigeno Y, Toyoshima M, Okuno A, Takao H, Toyota T, Minabe Y, Nakamura K, Shimizu E, Itokawa M, Mori N, Iyo M, Yoshikawa T (2005) Identification of multiple serine racemase (SRR) mRNA isoforms and genetic analyses of SRR and DAO in schizophrenia and D-serine levels. Biological psychiatry 57:1493-1503.

Yamaguchi K, Sawada T, Naraki T, Igata-Yi R, Shiraki H, Horii Y, Ishii T, Ikeda K, Asou N, Okabe H, Mochizuki M, Takahashi K, Yamada S, Kubo K, Yashiki S, Waltrip RW, 2nd, Carbone KM (1999) Detection of borna disease virus-reactive antibodies from patients with psychiatric disorders and from horses by electrochemiluminescence immunoassay. Clinical and diagnostic laboratory immunology 6:696-700.

Yu L, Li T, Robertson Z, Dean J, Gu NF, Feng GY, Yates P, Sinclair M, Crombie C, Collier DA, Walker N, He L, St Clair D (2004) No association between polymorphisms of methylenetetrahydrofolate reductase gene and schizophrenia in both Chinese and Scottish populations. Molecular psychiatry 9:1063-1065.

Zhang XY, Tan YL, Cao LY, Wu GY, Xu Q, Shen Y, Zhou DF (2006) Antioxidant enzymes and lipid peroxidation in different forms of schizophrenia treated with typical and atypical antipsychotics. Schizophrenia research 81:291-300.

**Alzheimer’s disease**

Araria-Goumidi L, Lambert JC, Mann DM, Lendon C, Frigard B, Iwatsubo T, Cottel D, Amouyel P, Chartier-Harlin MC (2002) Association study of three polymorphisms of TGF-beta1 gene with Alzheimer's disease. Journal of neurology, neurosurgery, and psychiatry 73:62-64.

Arvanitakis Z, Lucas JA, Younkin LH, Younkin SG, Graff-Radford NR (2002) Serum creatinine levels correlate with plasma amyloid Beta protein. Alzheimer disease and associated disorders 16:187-190.

Belbin O, Carrasquillo MM, Crump M, Culley OJ, Hunter TA, Ma L, Bisceglio G, Zou F, Allen M, Dickson DW, Graff-Radford NR, Petersen RC, Morgan K, Younkin SG (2011) Investigation of 15 of the top candidate genes for late-onset Alzheimer's disease. Human genetics 129:273-282.

Bi XH, Zhao HL, Zhang ZX, Zhang JW (2009) Association of RFC1 A80G and MTHFR C677T polymorphisms with Alzheimer's disease. Neurobiology of aging 30:1601-1607.

Blennow K, Wallin A, Fredman P, Karlsson I, Gottfries CG, Svennerholm L (1990) Blood-brain barrier disturbance in patients with Alzheimer's disease is related to vascular factors. Acta neurologica Scandinavica 81:323-326.

Chapuis J, Moisan F, Mellick G, Elbaz A, Silburn P, Pasquier F, Hannequin D, Lendon C, Campion D, Amouyel P, Lambert JC (2008) Association study of the NEDD9 gene with the risk of developing Alzheimer's and Parkinson's disease. Human molecular genetics 17:2863-2867.

Cornett CR, Markesbery WR, Ehmann WD (1998) Imbalances of trace elements related to oxidative damage in Alzheimer's disease brain. Neurotoxicology 19:339-345.

Cousin E, Mace S, Rocher C, Dib C, Muzard G, Hannequin D, Pradier L, Deleuze JF, Genin E, Brice A, Campion D (2011) No replication of genetic association between candidate polymorphisms and Alzheimer's disease. Neurobiology of aging 32:1443-1451.

Davidson Y, Gibbons L, Pritchard A, Hardicre J, Wren J, Tian J, Shi J, Stopford C, Julien C, Thompson J, Payton A, Thaker U, Hayes AJ, Iwatsubo T, Pickering-Brown SM, Pendleton N, Horan MA, Burns A, Purandare N, Lendon CL, Neary D, Snowden JS, Mann DM (2006) Genetic associations between cathepsin D exon 2 C-->T polymorphism and Alzheimer's disease, and pathological correlations with genotype. Journal of neurology, neurosurgery, and psychiatry 77:515-517.

Depboylu C, Du Y, Muller U, Kurz A, Zimmer R, Riemenschneider M, Gasser T, Oertel WH, Klockgether T, Dodel RC (2003) Lack of association of interleukin-10 promoter region polymorphisms with Alzheimer's disease. Neuroscience letters 342:132-134.

Desai P, Nebes R, DeKosky ST, Kamboh MI (2005) Investigation of the effect of brain-derived neurotrophic factor (BDNF) polymorphisms on the risk of late-onset Alzheimer's disease (AD) and quantitative measures of AD progression. Neuroscience letters 379:229-234.

Engelhart MJ, Geerlings MI, Meijer J, Kiliaan A, Ruitenberg A, van Swieten JC, Stijnen T, Hofman A, Witteman JC, Breteler MM (2004) Inflammatory proteins in plasma and the risk of dementia: the rotterdam study. Archives of neurology 61:668-672.

Finckh U, von der Kammer H, Velden J, Michel T, Andresen B, Deng A, Zhang J, Muller-Thomsen T, Zuchowski K, Menzer G, Mann U, Papassotiropoulos A, Heun R, Zurdel J, Holst F, Benussi L, Stoppe G, Reiss J, Miserez AR, Staehelin HB, Rebeck GW, Hyman BT, Binetti G, Hock C, Growdon JH, Nitsch RM (2000) Genetic association of a cystatin C gene polymorphism with late-onset Alzheimer disease. Archives of neurology 57:1579-1583.

Fleischman DA, Gabrieli JD, Gilley DW, Hauser JD, Lange KL, Dwornik LM, Bennett DA, Wilson RS (1999) Word-stem completion priming in healthy aging and Alzheimer's disease: the effects of age, cognitive status, and encoding. Neuropsychology 13:22-30.

Grupe A, Abraham R, Li Y, Rowland C, Hollingworth P, Morgan A, Jehu L, Segurado R, Stone D, Schadt E, Karnoub M, Nowotny P, Tacey K, Catanese J, Sninsky J, Brayne C, Rubinsztein D, Gill M, Lawlor B, Lovestone S, Holmans P, O'Donovan M, Morris JC, Thal L, Goate A, Owen MJ, Williams J (2007) Evidence for novel susceptibility genes for late-onset Alzheimer's disease from a genome-wide association study of putative functional variants. Human molecular genetics 16:865-873.

Haines JL, Pritchard ML, Saunders AM, Schildkraut JM, Growdon JH, Gaskell PC, Farrer LA, Auerbach SA, Gusella JF, Locke PA, Rosi BL, Yamaoka L, Small GW, Conneally PM, Roses AD, Pericak-Vance MA (1996) No genetic effect of alpha1-antichymotrypsin in Alzheimer disease. Genomics 33:53-56.

Harold D, Abraham R, Hollingworth P, Sims R, Gerrish A, Hamshere ML, Pahwa JS, Moskvina V, Dowzell K, Williams A, Jones N, Thomas C, Stretton A, Morgan AR, Lovestone S, Powell J, Proitsi P, Lupton MK, Brayne C, Rubinsztein DC, Gill M, Lawlor B, Lynch A, Morgan K, Brown KS, Passmore PA, Craig D, McGuinness B, Todd S, Holmes C, Mann D, Smith AD, Love S, Kehoe PG, Hardy J, Mead S, Fox N, Rossor M, Collinge J, Maier W, Jessen F, Schurmann B, Heun R, van den Bussche H, Heuser I, Kornhuber J, Wiltfang J, Dichgans M, Frolich L, Hampel H, Hull M, Rujescu D, Goate AM, Kauwe JS, Cruchaga C, Nowotny P, Morris JC, Mayo K, Sleegers K, Bettens K, Engelborghs S, De Deyn PP, Van Broeckhoven C, Livingston G, Bass NJ, Gurling H, McQuillin A, Gwilliam R, Deloukas P, Al-Chalabi A, Shaw CE, Tsolaki M, Singleton AB, Guerreiro R, Muhleisen TW, Nothen MM, Moebus S, Jockel KH, Klopp N, Wichmann HE, Carrasquillo MM, Pankratz VS, Younkin SG, Holmans PA, O'Donovan M, Owen MJ, Williams J (2009) Genome-wide association study identifies variants at CLU and PICALM associated with Alzheimer's disease. Nature genetics 41:1088-1093.

Hogervorst E, Ribeiro HM, Molyneux A, Budge M, Smith AD (2002) Plasma homocysteine levels, cerebrovascular risk factors, and cerebral white matter changes (leukoaraiosis) in patients with Alzheimer disease. Archives of neurology 59:787-793.

in't Veld BA, Ruitenberg A, Hofman A, Stricker BH, Breteler MM (2001) Antihypertensive drugs and incidence of dementia: the Rotterdam Study. Neurobiology of aging 22:407-412.

Johansson A, Katzov H, Zetterberg H, Feuk L, Johansson B, Bogdanovic N, Andreasen N, Lenhard B, Brookes AJ, Pedersen NL, Blennow K, Prince JA (2004) Variants of CYP46A1 may interact with age and APOE to influence CSF Abeta42 levels in Alzheimer's disease. Human genetics 114:581-587.

Jungwirth S, Zehetmayer S, Bauer P, Weissgram S, Tragl KH, Fischer P (2009) Screening for Alzheimer's dementia at age 78 with short psychometric instruments. International psychogeriatrics / IPA 21:548-559.

Klimkowicz-Mrowiec A, Marona M, Wolkow P, Maruszak A, Styczynska M, Barcikowska M, Zekanowski C, Szczudlik A, Slowik A (2009) Interleukin-1 gene -511 CT polymorphism and the risk of Alzheimer's disease in a Polish population. Dementia and geriatric cognitive disorders 28:461-464.

Kokmen E, Beard CM, Chandra V, Offord KP, Schoenberg BS, Ballard DJ (1991) Clinical risk factors for Alzheimer's disease: a population-based case-control study. Neurology 41:1393-1397.

Lambert JC, Berr C, Pasquier F, Delacourte A, Frigard B, Cottel D, Perez-Tur J, Mouroux V, Mohr M, Cecyre D, Galasko D, Lendon C, Poirier J, Hardy J, Mann D, Amouyel P, Chartier-Harlin MC (1998) Pronounced impact of Th1/E47cs mutation compared with -491 AT mutation on neural APOE gene expression and risk of developing Alzheimer's disease. Human molecular genetics 7:1511-1516.

Laws SM, Perneczky R, Wagenpfeil S, Muller U, Forstl H, Martins RN, Kurz A, Riemenschneider M (2005) TNF polymorphisms in Alzheimer disease and functional implications on CSF beta-amyloid levels. Human mutation 26:29-35.

Lehmann DJ, Schuur M, Warden DR, Hammond N, Belbin O, Kolsch H, Lehmann MG, Wilcock GK, Brown K, Kehoe PG, Morris CM, Barker R, Coto E, Alvarez V, Deloukas P, Mateo I, Gwilliam R, Combarros O, Arias-Vasquez A, Aulchenko YS, Ikram MA, Breteler MM, van Duijn CM, Oulhaj A, Heun R, Cortina-Borja M, Morgan K, Robson K, Smith AD (2012) Transferrin and HFE genes interact in Alzheimer's disease risk: the Epistasis Project. Neurobiology of aging 33:202 e201-213.

Li H, Wetten S, Li L, St Jean PL, Upmanyu R, Surh L, Hosford D, Barnes MR, Briley JD, Borrie M, Coletta N, Delisle R, Dhalla D, Ehm MG, Feldman HH, Fornazzari L, Gauthier S, Goodgame N, Guzman D, Hammond S, Hollingworth P, Hsiung GY, Johnson J, Kelly DD, Keren R, Kertesz A, King KS, Lovestone S, Loy-English I, Matthews PM, Owen MJ, Plumpton M, Pryse-Phillips W, Prinjha RK, Richardson JC, Saunders A, Slater AJ, St George-Hyslop PH, Stinnett SW, Swartz JE, Taylor RL, Wherrett J, Williams J, Yarnall DP, Gibson RA, Irizarry MC, Middleton LT, Roses AD (2008) Candidate single-nucleotide polymorphisms from a genomewide association study of Alzheimer disease. Archives of neurology 65:45-53.

Li Y, Tacey K, Doil L, van Luchene R, Garcia V, Rowland C, Schrodi S, Leong D, Lau K, Catanese J, Sninsky J, Nowotny P, Holmans P, Hardy J, Powell J, Lovestone S, Thal L, Owen M, Williams J, Goate A, Grupe A (2004) Association of ABCA1 with late-onset Alzheimer's disease is not observed in a case-control study. Neuroscience letters 366:268-271.

Lindsay J, Laurin D, Verreault R, Hebert R, Helliwell B, Hill GB, McDowell I (2002) Risk factors for Alzheimer's disease: a prospective analysis from the Canadian Study of Health and Aging. American journal of epidemiology 156:445-453.

Lovati C, Galimberti D, Albani D, Bertora P, Venturelli E, Cislaghi G, Guidi I, Fenoglio C, Cortini F, Clerici F, Finazzi D, Forloni G, Scarpini E, Mariani C (2010) APOE epsilon2 and epsilon4 influence the susceptibility for Alzheimer's disease but not other dementias. International journal of molecular epidemiology and genetics 1:193-200.

Nordin S, Monsch AU, Murphy C (1995) Unawareness of smell loss in normal aging and Alzheimer's disease: discrepancy between self-reported and diagnosed smell sensitivity. The journals of gerontology Series B, Psychological sciences and social sciences 50:P187-192.

Nowotny P, Bertelsen S, Hinrichs AL, Kauwe JS, Mayo K, Jacquart S, Morris JC, Goate A (2007) Association studies between common variants in prolyl isomerase Pin1 and the risk for late-onset Alzheimer's disease. Neuroscience letters 419:15-17.

Nowotny P, Kwon JM, Chakraverty S, Nowotny V, Morris JC, Goate AM (2001) Association studies using novel polymorphisms in BACE1 and BACE2. Neuroreport 12:1799-1802.

Reiman EM, Webster JA, Myers AJ, Hardy J, Dunckley T, Zismann VL, Joshipura KD, Pearson JV, Hu-Lince D, Huentelman MJ, Craig DW, Coon KD, Liang WS, Herbert RH, Beach T, Rohrer KC, Zhao AS, Leung D, Bryden L, Marlowe L, Kaleem M, Mastroeni D, Grover A, Heward CB, Ravid R, Rogers J, Hutton ML, Melquist S, Petersen RC, Alexander GE, Caselli RJ, Kukull W, Papassotiropoulos A, Stephan DA (2007) GAB2 alleles modify Alzheimer's risk in APOE epsilon4 carriers. Neuron 54:713-720.

Reynolds CA, Hong MG, Eriksson UK, Blennow K, Johansson B, Malmberg B, Berg S, Gatz M, Pedersen NL, Bennet AM, Prince JA (2010) Sequence variation in SORL1 and dementia risk in Swedes. Neurogenetics 11:139-142.

Sevim S, Unal O, Tamer L, Dogu O, Ozge A (2007) Can serum levels of copper and zinc distinguish Alzheimer's patients from normal subjects? J Neurol Sci (turkish) 24:197-205.

van Oijen M, Arp PP, de Jong FJ, Hofman A, Koudstaal PJ, Uitterlinden AG, Breteler MM (2006) Polymorphisms in the interleukin 6 and transforming growth factor beta1 gene and risk of dementia. The Rotterdam Study. Neuroscience letters 402:113-117.

Vicenzini E, Ricciardi MC, Altieri M, Puccinelli F, Bonaffini N, Di Piero V, Lenzi GL (2007) Cerebrovascular reactivity in degenerative and vascular dementia: a transcranial Doppler study. European neurology 58:84-89.

Wahrle SE, Shah AR, Fagan AM, Smemo S, Kauwe JS, Grupe A, Hinrichs A, Mayo K, Jiang H, Thal LJ, Goate AM, Holtzman DM (2007) Apolipoprotein E levels in cerebrospinal fluid and the effects of ABCA1 polymorphisms. Molecular neurodegeneration 2:7.

Ward NI, Mason JA (1987) Neutron activation analysis techniques for identifying elemental status in Alzheimer's disease Journal of radioanalytical and nuclear chemistry 113:515-526.

Wingo TS, Rosen A, Cutler DJ, Lah JJ, Levey AI (2012) Paraoxonase-1 polymorphisms in Alzheimer's disease, Parkinson's disease, and AD-PD spectrum diseases. Neurobiology of aging 33:204 e213-205.

Zhou YT, Zhang ZX, Zhang JW, He XM, Xu T (2006) [Association between interleukin-1 alpha-889 C/T polymorphism and Alzheimer's disease in Chinese Han population]. Zhongguo yi xue ke xue yuan xue bao Acta Academiae Medicinae Sinicae 28:186-190.

**Epilepsy**

Badawy RA, Macdonell RA, Berkovic SF, Newton MR, Jackson GD (2010) Predicting seizure control: cortical excitability and antiepileptic medication. Annals of neurology 67:64-73.

Cavalleri GL, Lynch JM, Depondt C, Burley MW, Wood NW, Sisodiya SM, Goldstein DB (2005) Failure to replicate previously reported genetic associations with sporadic temporal lobe epilepsy: where to from here? Brain : a journal of neurology 128:1832-1840.

Diehl B, Busch RM, Duncan JS, Piao Z, Tkach J, Luders HO (2008) Abnormalities in diffusion tensor imaging of the uncinate fasciculus relate to reduced memory in temporal lobe epilepsy. Epilepsia 49:1409-1418.

Hallioglu O, Okuyaz C, Mert E, Makharoblidze K (2008) Effects of antiepileptic drug therapy on heart rate variability in children with epilepsy. Epilepsy research 79:49-54.

Jacobson JW, Janicki MP (1983) Observed prevalence of multiple developmental disabilities. Mental retardation 21:87-94.

Kinirons P, Cavalleri GL, Shahwan A, Wood NW, Goldstein DB, Sisodiya SM, Delanty N, Doherty CP (2006) Examining the role of common genetic variation in the gamma2 subunit of the GABA(A) receptor in epilepsy using tagging SNPs. Epilepsy research 70:229-238.

Kwan P, Baum L, Wong V, Ng PW, Lui CH, Sin NC, Hui AC, Yu E, Wong LK (2007) Association between ABCB1 C3435T polymorphism and drug-resistant epilepsy in Han Chinese. Epilepsy & behavior : E&B 11:112-117.

Leone M, Bottacchi E, Beghi E, Morgando E, Mutani R, Amedeo G, Cremo R, Gianelli M, Ravagli Ceroni L (1997) Alcohol use is a risk factor for a first generalized tonic-clonic seizure. The ALC.E. (Alcohol and Epilepsy) Study Group. Neurology 48:614-620.

Liimatainen S, Fallah M, Kharazmi E, Peltola M, Peltola J (2009) Interleukin-6 levels are increased in temporal lobe epilepsy but not in extra-temporal lobe epilepsy. Journal of neurology 256:796-802.

Nicoletti A, Sofia V, Mantella A, Vitale G, Contrafatto D, Sorbello V, Biondi R, Preux PM, Garcia HH, Zappia M, Bartoloni A (2008) Epilepsy and toxocariasis: a case-control study in Italy. Epilepsia 49:594-599.

Nsengiyumva G, Druet-Cabanac M, Ramanankandrasana B, Bouteille B, Nsizabira L, Preux PM (2003) Cysticercosis as a major risk factor for epilepsy in Burundi, east Africa. Epilepsia 44:950-955.

Ogunniyi A, Osuntokun BO, Bademosi O, Adeuja AO, Schoenberg BS (1987) Risk factors for epilepsy: case-control study in Nigerians. Epilepsia 28:280-285.

Tuchman RF, Rapin I, Shinnar S (1991) Autistic and dysphasic children. II: Epilepsy. Pediatrics 88:1219-1225.

**Multiple sclerosis**

Alvarez-Lafuente R, Garcia-Montojo M, De Las Heras V, Dominguez-Mozo MI, Bartolome M, Benito-Martin MS, Arroyo R (2008) Herpesviruses and human endogenous retroviral sequences in the cerebrospinal fluid of multiple sclerosis patients. Multiple sclerosis (Houndmills, Basingstoke, England) 14:595-601.

ANZGene (2009) Genome-wide association study identifies new multiple sclerosis susceptibility loci on chromosomes 12 and 20. Nature genetics 41:824-828.

Bray PF, Bloomer LC, Salmon VC, Bagley MH, Larsen PD (1983) Epstein-Barr virus infection and antibody synthesis in patients with multiple sclerosis. Archives of neurology 40:406-408.

Cocco E, Sotgiu A, Costa G, Murru MR, Mancosu C, Murru R, Lai M, Contu P, Marrosu MG (2005) HLA-DR,DQ and APOE genotypes and gender influence in Sardinian primary progressive MS. Neurology 64:564-566.

Cox MB, Ban M, Bowden NA, Baker A, Scott RJ, Lechner-Scott J (2012) Potential association of vitamin D receptor polymorphism Taq1 with multiple sclerosis. Multiple sclerosis (Houndmills, Basingstoke, England) 18:16-22.

de Jong BA, Westendorp RG, Eskdale J, Uitdehaag BM, Huizinga TW (2002) Frequency of functional interleukin-10 promoter polymorphism is different between relapse-onset and primary progressive multiple sclerosis. Human immunology 63:281-285.

DeStefano F, Verstraeten T, Jackson LA, Okoro CA, Benson P, Black SB, Shinefield HR, Mullooly JP, Likosky W, Chen RT (2003) Vaccinations and risk of central nervous system demyelinating diseases in adults. Archives of neurology 60:504-509.

Edwards LJ, Constantinescu CS (2004) A prospective study of conditions associated with multiple sclerosis in a cohort of 658 consecutive outpatients attending a multiple sclerosis clinic. Multiple sclerosis (Houndmills, Basingstoke, England) 10:575-581.

Fernandez-Arquero M, Arroyo R, Rubio A, Martin C, Vigil P, Conejero L, Figueredo MA, de la Concha EG (1999) Primary association of a TNF gene polymorphism with susceptibility to multiple sclerosis. Neurology 53:1361-1363.

Hafler DA, Compston A, Sawcer S, Lander ES, Daly MJ, De Jager PL, de Bakker PI, Gabriel SB, Mirel DB, Ivinson AJ, Pericak-Vance MA, Gregory SG, Rioux JD, McCauley JL, Haines JL, Barcellos LF, Cree B, Oksenberg JR, Hauser SL (2007) Risk alleles for multiple sclerosis identified by a genomewide study. The New England journal of medicine 357:851-862.

Hedstrom AK, Baarnhielm M, Olsson T, Alfredsson L (2009) Tobacco smoking, but not Swedish snuff use, increases the risk of multiple sclerosis. Neurology 73:696-701.

Hooper-van Veen T, Schrijver HM, Zwiers A, Crusius JB, Knol DL, Kalkers NF, Laine ML, Barkhof F, Pena AS, Polman CH, Uitdehaag BM (2003) The interleukin-1 gene family in multiple sclerosis susceptibility and disease course. Multiple sclerosis (Houndmills, Basingstoke, England) 9:535-539.

Kister I, Munger KL, Herbert J, Ascherio A (2012) Increased risk of multiple sclerosis among women with migraine in the Nurses' Health Study II. Multiple sclerosis (Houndmills, Basingstoke, England) 18:90-97.

Link J, Lorentzen AR, Kockum I, Duvefelt K, Lie BA, Celius EG, Harbo HF, Hillert J, Brynedal B (2010) Two HLA class I genes independently associated with multiple sclerosis. Journal of neuroimmunology 226:172-176.

Maier LM, Lowe CE, Cooper J, Downes K, Anderson DE, Severson C, Clark PM, Healy B, Walker N, Aubin C, Oksenberg JR, Hauser SL, Compston A, Sawcer S, De Jager PL, Wicker LS, Todd JA, Hafler DA (2009) IL2RA genetic heterogeneity in multiple sclerosis and type 1 diabetes susceptibility and soluble interleukin-2 receptor production. PLoS genetics 5:e1000322.

Marrosu MG, Lai M, Cocco E, Loi V, Spinicci G, Pischedda MP, Massole S, Marrosu G, Contu P (2002) Genetic factors and the founder effect explain familial MS in Sardinia. Neurology 58:283-288.

Pei J, Xu WZ, Sun SX, Mi XY, Hou ZC, Wang LH (1987) HLA and multiple sclerosis in Chinese. Proceedings of the Chinese Academy of Medical Sciences and the Peking Union Medical College = Chung-kuo i hsueh k'o hsueh yuan, Chung-kuo hsieh ho i k'o ta hsueh hsueh pao 2:111-115.

Ramagopalan SV, Valdar W, Dyment DA, DeLuca GC, Yee IM, Giovannoni G, Ebers GC, Sadovnick AD (2009) Association of infectious mononucleosis with multiple sclerosis. A population-based study. Neuroepidemiology 32:257-262.

Riise T, Kirkeleit J, Aarseth JH, Farbu E, Midgard R, Mygland A, Eikeland R, Morland TJ, Telstad W, Forland PT, Myhr KM (2011) Risk of MS is not associated with exposure to crude oil, but increases with low level of education. Multiple sclerosis (Houndmills, Basingstoke, England) 17:780-787.

Ristic S, Lovrecic L, Starcevic-Cizmarevic N, Brajenovic-Milic B, Sega Jazbec S, Sepcic J, Kapovic M, Peterlin B (2007) Tumor necrosis factor-alpha-308 gene polymorphism in Croatian and Slovenian multiple sclerosis patients. European neurology 57:203-207.

Russo C, Morabito F, Luise F, Piromalli A, Battaglia L, Vinci A, Trapani Lombardo V, de Marco V, Morabito P, Condino F, Quattrone A, Aguglia U (2008) Hyperhomocysteinemia is associated with cognitive impairment in multiple sclerosis. Journal of neurology 255:64-69.

Sadovnick AD, Baird PA (1988) The familial nature of multiple sclerosis: age-corrected empiric recurrence risks for children and siblings of patients. Neurology 38:990-991.

Sundstrom P, Juto P, Wadell G, Hallmans G, Svenningsson A, Nystrom L, Dillner J, Forsgren L (2004) An altered immune response to Epstein-Barr virus in multiple sclerosis: a prospective study. Neurology 62:2277-2282.

Teunissen CE, Killestein J, Kragt JJ, Polman CH, Dijkstra CD, Blom HJ (2008) Serum homocysteine levels in relation to clinical progression in multiple sclerosis. Journal of neurology, neurosurgery, and psychiatry 79:1349-1353.

Zorzon M, Zivadinov R, Nasuelli D, Dolfini P, Bosco A, Bratina A, Tommasi MA, Locatelli L, Cazzato G (2003) Risk factors of multiple sclerosis: a case-control study. Neurological sciences : official journal of the Italian Neurological Society and of the Italian Society of Clinical Neurophysiology 24:242-247.

**Parkinson’s Disease**

Ascherio A, Weisskopf MG, O'Reilly EJ, McCullough ML, Calle EE, Rodriguez C, Thun MJ (2004) Coffee consumption, gender, and Parkinson's disease mortality in the cancer prevention study II cohort: the modifying effects of estrogen. American journal of epidemiology 160:977-984.

Becker C, Jick SS, Meier CR (2008) Use of statins and the risk of Parkinson's disease: a retrospective case-control study in the UK. Drug safety : an international journal of medical toxicology and drug experience 31:399-407.

Bialecka M, Kurzawski M, Klodowska-Duda G, Opala G, Tan EK, Drozdzik M (2008) The association of functional catechol-O-methyltransferase haplotypes with risk of Parkinson's disease, levodopa treatment response, and complications. Pharmacogenetics and genomics 18:815-821.

Bocca B, Alimonti A, Senofonte O, Pino A, Violante N, Petrucci F, Sancesario G, Forte G (2006) Metal changes in CSF and peripheral compartments of parkinsonian patients. Journal of the neurological sciences 248:23-30.

Boesveldt S, Verbaan D, Knol DL, Visser M, van Rooden SM, van Hilten JJ, Berendse HW (2008) A comparative study of odor identification and odor discrimination deficits in Parkinson's disease. Movement disorders : official journal of the Movement Disorder Society 23:1984-1990.

Brandt-Christensen M, Kvist K, Nilsson FM, Andersen PK, Kessing LV (2006) Treatment with antidepressants and lithium is associated with increased risk of treatment with antiparkinson drugs: a pharmacoepidemiological study. Journal of neurology, neurosurgery, and psychiatry 77:781-783.

Brighina L, Frigerio R, Schneider NK, Lesnick TG, de Andrade M, Cunningham JM, Farrer MJ, Lincoln SJ, Checkoway H, Rocca WA, Maraganore DM (2008) Alpha-synuclein, pesticides, and Parkinson disease: a case-control study. Neurology 70:1461-1469.

Brighina L, Okubadejo NU, Schneider NK, Lesnick TG, de Andrade M, Cunningham JM, Farrer MJ, Lincoln SJ, Rocca WA, Maraganore DM (2007) Beta-synuclein gene variants and Parkinson's disease: a preliminary case-control study. Neuroscience letters 420:229-234.

Dick b FD, De Palma G, Ahmadi A, Scott NW, Prescott GJ, Bennett J, Semple S, Dick S, Counsell C, Mozzoni P, Haites N, Wettinger SB, Mutti A, Otelea M, Seaton A, Soderkvist P, Felice A (2007) Environmental risk factors for Parkinson's disease and parkinsonism: the Geoparkinson study. Occupational and environmental medicine 64:666-672.

Dick FD, De Palma G, Ahmadi A, Osborne A, Scott NW, Prescott GJ, Bennett J, Semple S, Dick S, Mozzoni P, Haites N, Wettinger SB, Mutti A, Otelea M, Seaton A, Soderkvist P, Felice A (2007) Gene-environment interactions in parkinsonism and Parkinson's disease: the Geoparkinson study. Occupational and environmental medicine 64:673-680.

Do CB, Tung JY, Dorfman E, Kiefer AK, Drabant EM, Francke U, Mountain JL, Goldman SM, Tanner CM, Langston JW, Wojcicki A, Eriksson N (2011) Web-based genome-wide association study identifies two novel loci and a substantial genetic component for Parkinson's disease. PLoS genetics 7:e1002141.

Eerola J, Luoma PT, Peuralinna T, Scholz S, Paisan-Ruiz C, Suomalainen A, Singleton AB, Tienari PJ (2010) POLG1 polyglutamine tract variants associated with Parkinson's disease. Neuroscience letters 477:1-5.

Etminan M, Carleton BC, Samii A (2008) Non-steroidal anti-inflammatory drug use and the risk of Parkinson disease: a retrospective cohort study. Journal of clinical neuroscience : official journal of the Neurosurgical Society of Australasia 15:576-577.

Fuchs J, Mueller JC, Lichtner P, Schulte C, Munz M, Berg D, Wullner U, Illig T, Sharma M, Gasser T (2009) The transcription factor PITX3 is associated with sporadic Parkinson's disease. Neurobiology of aging 30:731-738.

Hamza TH, Zabetian CP, Tenesa A, Laederach A, Montimurro J, Yearout D, Kay DM, Doheny KF, Paschall J, Pugh E, Kusel VI, Collura R, Roberts J, Griffith A, Samii A, Scott WK, Nutt J, Factor SA, Payami H (2010) Common genetic variation in the HLA region is associated with late-onset sporadic Parkinson's disease. Nature genetics 42:781-785.

Hernan MA, Logroscino G, Rodriguez LA (2004) A prospective study of alcoholism and the risk of Parkinson's disease. Journal of neurology 251 Suppl 7:vII14-17.

Hutter CM, Samii A, Factor SA, Nutt JG, Higgins DS, Bird TD, Griffith A, Roberts JW, Leis BC, Montimurro JS, Kay DM, Edwards KL, Payami H, Zabetian CP (2008) Lack of evidence for an association between UCHL1 S18Y and Parkinson's disease. European journal of neurology : the official journal of the European Federation of Neurological Societies 15:134-139.

IPDGC (2011) A two-stage meta-analysis identifies several new loci for Parkinson's disease. PLoS genetics 7:e1002142.

Kay DM, Zabetian CP, Factor SA, Nutt JG, Samii A, Griffith A, Bird TD, Kramer P, Higgins DS, Payami H (2006) Parkinson's disease and LRRK2: frequency of a common mutation in U.S. movement disorder clinics. Movement disorders : official journal of the Movement Disorder Society 21:519-523.

Klebe S, Thier S, Lorenz D, Nothnagel M, Schreiber S, Klein C, Hagenah J, Kasten M, Berg D, Srulijes K, Gasser T, Deuschl G, Kuhlenbaumer G (2010) LINGO1 is not associated with Parkinson's disease in German patients. American journal of medical genetics Part B, Neuropsychiatric genetics : the official publication of the International Society of Psychiatric Genetics 153B:1173-1178.

Lincoln SJ, Maraganore DM, Lesnick TG, Bounds R, de Andrade M, Bower JH, Hardy JA, Farrer MJ (2003) Parkin variants in North American Parkinson's disease: cases and controls. Movement disorders : official journal of the Movement Disorder Society 18:1306-1311.

Logroscino G, Marder K, Graziano J, Freyer G, Slavkovich V, LoIacono N, Cote L, Mayeux R (1997) Altered systemic iron metabolism in Parkinson's disease. Neurology 49:714-717.

Olsen JH, Friis S, Frederiksen K (2006) Malignant melanoma and other types of cancer preceding Parkinson disease. Epidemiology (Cambridge, Mass) 17:582-587.

Paganini-Hill A (2001) Risk factors for parkinson's disease: the leisure world cohort study. Neuroepidemiology 20:118-124.

Quinn NP, Rossor MN, Marsden CD (1987) Olfactory threshold in Parkinson's disease. Journal of neurology, neurosurgery, and psychiatry 50:88-89.

Rosen AR, Steenland NK, Hanfelt J, Factor SA, Lah JJ, Levey AI (2007) Evidence of shared risk for Alzheimer's disease and Parkinson's disease using family history. Neurogenetics 8:263-270.

Ross OA, Soto-Ortolaza AI, Heckman MG, Aasly JO, Abahuni N, Annesi G, Bacon JA, Bardien S, Bozi M, Brice A, Brighina L, Van Broeckhoven C, Carr J, Chartier-Harlin MC, Dardiotis E, Dickson DW, Diehl NN, Elbaz A, Ferrarese C, Ferraris A, Fiske B, Gibson JM, Gibson R, Hadjigeorgiou GM, Hattori N, Ioannidis JP, Jasinska-Myga B, Jeon BS, Kim YJ, Klein C, Kruger R, Kyratzi E, Lesage S, Lin CH, Lynch T, Maraganore DM, Mellick GD, Mutez E, Nilsson C, Opala G, Park SS, Puschmann A, Quattrone A, Sharma M, Silburn PA, Sohn YH, Stefanis L, Tadic V, Theuns J, Tomiyama H, Uitti RJ, Valente EM, van de Loo S, Vassilatis DK, Vilarino-Guell C, White LR, Wirdefeldt K, Wszolek ZK, Wu RM, Farrer MJ (2011) Association of LRRK2 exonic variants with susceptibility to Parkinson's disease: a case-control study. Lancet neurology 10:898-908.

Rugbjerg K, Friis S, Ritz B, Schernhammer ES, Korbo L, Olsen JH (2009) Autoimmune disease and risk for Parkinson disease: a population-based case-control study. Neurology 73:1462-1468.

Rugbjerg K, Ritz B, Korbo L, Martinussen N, Olsen JH (2008) Risk of Parkinson's disease after hospital contact for head injury: population based case-control study. BMJ (Clinical research ed) 337:a2494.

Samii A, Carleton BC, Etminan M (2008) Statin use and the risk of Parkinson disease: a nested case control study. Journal of clinical neuroscience : official journal of the Neurosurgical Society of Australasia 15:1272-1273.

Shino MY, McGuire V, Van Den Eeden SK, Tanner CM, Popat R, Leimpeter A, Bernstein AL, Nelson LM (2010) Familial aggregation of Parkinson's disease in a multiethnic community-based case-control study. Movement disorders : official journal of the Movement Disorder Society 25:2587-2594.

Simon-Sanchez J, Schulte C, Bras JM, Sharma M, Gibbs JR, Berg D, Paisan-Ruiz C, Lichtner P, Scholz SW, Hernandez DG, Kruger R, Federoff M, Klein C, Goate A, Perlmutter J, Bonin M, Nalls MA, Illig T, Gieger C, Houlden H, Steffens M, Okun MS, Racette BA, Cookson MR, Foote KD, Fernandez HH, Traynor BJ, Schreiber S, Arepalli S, Zonozi R, Gwinn K, van der Brug M, Lopez G, Chanock SJ, Schatzkin A, Park Y, Hollenbeck A, Gao J, Huang X, Wood NW, Lorenz D, Deuschl G, Chen H, Riess O, Hardy JA, Singleton AB, Gasser T (2009) Genome-wide association study reveals genetic risk underlying Parkinson's disease. Nature genetics 41:1308-1312.

Uitti RJ, Baba Y, Whaley NR, Wszolek ZK, Putzke JD (2005) Parkinson disease: handedness predicts asymmetry. Neurology 64:1925-1930.

Williams-Gray CH, Goris A, Saiki M, Foltynie T, Compston DA, Sawcer SJ, Barker RA (2009) Apolipoprotein E genotype as a risk factor for susceptibility to and dementia in Parkinson's disease. Journal of neurology 256:493-498.

Wu YR, Chen CM, Hwang JC, Chen ST, Feng IH, Hsu HC, Liu CN, Liu YT, Lai YY, Huang HJ, Lee-Chen GJ (2007) Interleukin-1 alpha polymorphism has influence on late-onset sporadic Parkinson's disease in Taiwan. Journal of neural transmission (Vienna, Austria : 1996) 114:1173-1177.

Wu YR, Wu CH, Chao CY, Kuan CC, Zhang WL, Wang CK, Chang CY, Chang YC, Lee-Chen GJ, Chen CM (2010) Genetic analysis of Parkin in early onset Parkinson's disease (PD): Novel intron 9 g > a single nucleotide polymorphism and risk of Taiwanese PD. American journal of medical genetics Part B, Neuropsychiatric genetics : the official publication of the International Society of Psychiatric Genetics 153B:229-234.

Yip JT, Lee TM, Ho SL, Tsang KL, Li LS (2003) Emotion recognition in patients with idiopathic Parkinson's disease. Movement disorders : official journal of the Movement Disorder Society 18:1115-1122.

Zhou YT, Yang JF, Zhang YL, Wang XY, Chan P (2008) Protective role of interlekin-1 alpha gene polymorphism in Chinese Han population with sporadic Parkinson's disease. Neuroscience letters 445:23-25.

**Breast cancer**

Abbas S, Linseisen J, Slanger T, Kropp S, Mutschelknauss EJ, Flesch-Janys D, Chang-Claude J (2008) Serum 25-hydroxyvitamin D and risk of post-menopausal breast cancer--results of a large case-control study. Carcinogenesis 29:93-99.

Ahlgren M, Melbye M, Wohlfahrt J, Sorensen TI (2004) Growth patterns and the risk of breast cancer in women. The New England journal of medicine 351:1619-1626.

Ahmed S, Thomas G, Ghoussaini M, Healey CS, Humphreys MK, Platte R, Morrison J, Maranian M, Pooley KA, Luben R, Eccles D, Evans DG, Fletcher O, Johnson N, dos Santos Silva I, Peto J, Stratton MR, Rahman N, Jacobs K, Prentice R, Anderson GL, Rajkovic A, Curb JD, Ziegler RG, Berg CD, Buys SS, McCarty CA, Feigelson HS, Calle EE, Thun MJ, Diver WR, Bojesen S, Nordestgaard BG, Flyger H, Dork T, Schurmann P, Hillemanns P, Karstens JH, Bogdanova NV, Antonenkova NN, Zalutsky IV, Bermisheva M, Fedorova S, Khusnutdinova E, Kang D, Yoo KY, Noh DY, Ahn SH, Devilee P, van Asperen CJ, Tollenaar RA, Seynaeve C, Garcia-Closas M, Lissowska J, Brinton L, Peplonska B, Nevanlinna H, Heikkinen T, Aittomaki K, Blomqvist C, Hopper JL, Southey MC, Smith L, Spurdle AB, Schmidt MK, Broeks A, van Hien RR, Cornelissen S, Milne RL, Ribas G, Gonzalez-Neira A, Benitez J, Schmutzler RK, Burwinkel B, Bartram CR, Meindl A, Brauch H, Justenhoven C, Hamann U, Chang-Claude J, Hein R, Wang-Gohrke S, Lindblom A, Margolin S, Mannermaa A, Kosma VM, Kataja V, Olson JE, Wang X, Fredericksen Z, Giles GG, Severi G, Baglietto L, English DR, Hankinson SE, Cox DG, Kraft P, Vatten LJ, Hveem K, Kumle M, Sigurdson A, Doody M, Bhatti P, Alexander BH, Hooning MJ, van den Ouweland AM, Oldenburg RA, Schutte M, Hall P, Czene K, Liu J, Li Y, Cox A, Elliott G, Brock I, Reed MW, Shen CY, Yu JC, Hsu GC, Chen ST, Anton-Culver H, Ziogas A, Andrulis IL, Knight JA, Beesley J, Goode EL, Couch F, Chenevix-Trench G, Hoover RN, Ponder BA, Hunter DJ, Pharoah PD, Dunning AM, Chanock SJ, Easton DF (2009) Newly discovered breast cancer susceptibility loci on 3p24 and 17q23.2. Nature genetics 41:585-590.

Al-Zahrani A, Sandhu MS, Luben RN, Thompson D, Baynes C, Pooley KA, Luccarini C, Munday H, Perkins B, Smith P, Pharoah PD, Wareham NJ, Easton DF, Ponder BA, Dunning AM (2006) IGF1 and IGFBP3 tagging polymorphisms are associated with circulating levels of IGF1, IGFBP3 and risk of breast cancer. Human molecular genetics 15:1-10.

Antoniou AC, Sinilnikova OM, Simard J, Leone M, Dumont M, Neuhausen SL, Struewing JP, Stoppa-Lyonnet D, Barjhoux L, Hughes DJ, Coupier I, Belotti M, Lasset C, Bonadona V, Bignon YJ, Rebbeck TR, Wagner T, Lynch HT, Domchek SM, Nathanson KL, Garber JE, Weitzel J, Narod SA, Tomlinson G, Olopade OI, Godwin A, Isaacs C, Jakubowska A, Lubinski J, Gronwald J, Gorski B, Byrski T, Huzarski T, Peock S, Cook M, Baynes C, Murray A, Rogers M, Daly PA, Dorkins H, Schmutzler RK, Versmold B, Engel C, Meindl A, Arnold N, Niederacher D, Deissler H, Spurdle AB, Chen X, Waddell N, Cloonan N, Kirchhoff T, Offit K, Friedman E, Kaufmann B, Laitman Y, Galore G, Rennert G, Lejbkowicz F, Raskin L, Andrulis IL, Ilyushik E, Ozcelik H, Devilee P, Vreeswijk MP, Greene MH, Prindiville SA, Osorio A, Benitez J, Zikan M, Szabo CI, Kilpivaara O, Nevanlinna H, Hamann U, Durocher F, Arason A, Couch FJ, Easton DF, Chenevix-Trench G (2007) RAD51 135G-->C modifies breast cancer risk among BRCA2 mutation carriers: results from a combined analysis of 19 studies. American journal of human genetics 81:1186-1200.

Askling J, Fored CM, Brandt L, Baecklund E, Bertilsson L, Feltelius N, Coster L, Geborek P, Jacobsson LT, Lindblad S, Lysholm J, Rantapaa-Dahlqvist S, Saxne T, Klareskog L (2005) Risks of solid cancers in patients with rheumatoid arthritis and after treatment with tumour necrosis factor antagonists. Annals of the rheumatic diseases 64:1421-1426.

BCAC (2006) Commonly studied single-nucleotide polymorphisms and breast cancer: results from the Breast Cancer Association Consortium. Journal of the National Cancer Institute 98:1382-1396.

Bernstein JL, Teraoka SN, John EM, Andrulis IL, Knight JA, Lapinski R, Olson ER, Wolitzer AL, Seminara D, Whittemore AS, Concannon P (2006) The CHEK2*1100delC allelic variant and risk of breast cancer: screening results from the Breast Cancer Family Registry. Cancer epidemiology, biomarkers & prevention : a publication of the American Association for Cancer Research, cosponsored by the American Society of Preventive Oncology 15:348-352.

Chang JH, Gertig DM, Chen X, Dite GS, Jenkins MA, Milne RL, Southey MC, McCredie MR, Giles GG, Chenevix-Trench G, Hopper JL, Spurdle AB (2005) CYP17 genetic polymorphism, breast cancer, and breast cancer risk factors: Australian Breast Cancer Family Study. Breast cancer research : BCR 7:R513-521.

CHECK2-BCC (2004) CHEK2*1100delC and susceptibility to breast cancer: a collaborative analysis involving 10,860 breast cancer cases and 9,065 controls from 10 studies. American journal of human genetics 74:1175-1182.

Colditz GA, Rosner BA, Chen WY, Holmes MD, Hankinson SE (2004) Risk factors for breast cancer according to estrogen and progesterone receptor status. Journal of the National Cancer Institute 96:218-228.

Cotterchio M, Boucher BA, Kreiger N, Mills CA, Thompson LU (2008) Dietary phytoestrogen intake--lignans and isoflavones--and breast cancer risk (Canada). Cancer causes & control : CCC 19:259-272.

Cox DG, Penney K, Guo Q, Hankinson SE, Hunter DJ (2007) TGFB1 and TGFBR1 polymorphisms and breast cancer risk in the Nurses' Health Study. BMC cancer 7:175.

Cronin-Fenton DP, Pedersen L, Lash TL, Friis S, Baron JA, Sorensen HT (2010) Prescriptions for selective cyclooxygenase-2 inhibitors, non-selective non-steroidal anti-inflammatory drugs, and risk of breast cancer in a population-based case-control study. Breast cancer research : BCR 12:R15.

Cui Y, Shikany JM, Liu S, Shagufta Y, Rohan TE (2008) Selected antioxidants and risk of hormone receptor-defined invasive breast cancers among postmenopausal women in the Women's Health Initiative Observational Study. The American journal of clinical nutrition 87:1009-1018.

Cybulski C, Gorski B, Huzarski T, Byrski T, Gronwald J, Debniak T, Wokolorczyk D, Jakubowska A, Serrano-Fernandez P, Dork T, Narod SA, Lubinski J (2009) Effect of CHEK2 missense variant I157T on the risk of breast cancer in carriers of other CHEK2 or BRCA1 mutations. Journal of medical genetics 46:132-135.

Dai Q, Shu XO, Jin F, Gao YT, Ruan ZX, Zheng W (2002) Consumption of animal foods, cooking methods, and risk of breast cancer. Cancer epidemiology, biomarkers & prevention : a publication of the American Association for Cancer Research, cosponsored by the American Society of Preventive Oncology 11:801-808.

Debniak T, Scott RJ, Huzarski T, Byrski T, Masojc B, van de Wetering T, Serrano-Fernandez P, Gorski B, Cybulski C, Gronwald J, Debniak B, Maleszka R, Kladny J, Bieniek A, Nagay L, Haus O, Grzybowska E, Wandzel P, Niepsuj S, Narod SA, Lubinski J (2006) XPD common variants and their association with melanoma and breast cancer risk. Breast cancer research and treatment 98:209-215.

Dunning AM, Ellis PD, McBride S, Kirschenlohr HL, Healey CS, Kemp PR, Luben RN, Chang-Claude J, Mannermaa A, Kataja V, Pharoah PD, Easton DF, Ponder BA, Metcalfe JC (2003) A transforming growth factorbeta1 signal peptide variant increases secretion in vitro and is associated with increased incidence of invasive breast cancer. Cancer research 63:2610-2615.

Dunning AM, Healey CS, Baynes C, Maia AT, Scollen S, Vega A, Rodriguez R, Barbosa-Morais NL, Ponder BA, Low YL, Bingham S, Haiman CA, Le Marchand L, Broeks A, Schmidt MK, Hopper J, Southey M, Beckmann MW, Fasching PA, Peto J, Johnson N, Bojesen SE, Nordestgaard B, Milne RL, Benitez J, Hamann U, Ko Y, Schmutzler RK, Burwinkel B, Schurmann P, Dork T, Heikkinen T, Nevanlinna H, Lindblom A, Margolin S, Mannermaa A, Kosma VM, Chen X, Spurdle A, Change-Claude J, Flesch-Janys D, Couch FJ, Olson JE, Severi G, Baglietto L, Borresen-Dale AL, Kristensen V, Hunter DJ, Hankinson SE, Devilee P, Vreeswijk M, Lissowska J, Brinton L, Liu J, Hall P, Kang D, Yoo KY, Shen CY, Yu JC, Anton-Culver H, Ziogoas A, Sigurdson A, Struewing J, Easton DF, Garcia-Closas M, Humphreys MK, Morrison J, Pharoah PD, Pooley KA, Chenevix-Trench G (2009) Association of ESR1 gene tagging SNPs with breast cancer risk. Human molecular genetics 18:1131-1139.

Fina F, Romain S, Ouafik L, Palmari J, Ben Ayed F, Benharkat S, Bonnier P, Spyratos F, Foekens JA, Rose C, Buisson M, Gerard H, Reymond MO, Seigneurin JM, Martin PM (2001) Frequency and genome load of Epstein-Barr virus in 509 breast cancers from different geographical areas. British journal of cancer 84:783-790.

Fletcher O, Johnson N, Orr N, Hosking FJ, Gibson LJ, Walker K, Zelenika D, Gut I, Heath S, Palles C, Coupland B, Broderick P, Schoemaker M, Jones M, Williamson J, Chilcott-Burns S, Tomczyk K, Simpson G, Jacobs KB, Chanock SJ, Hunter DJ, Tomlinson IP, Swerdlow A, Ashworth A, Ross G, dos Santos Silva I, Lathrop M, Houlston RS, Peto J (2011) Novel breast cancer susceptibility locus at 9q31.2: results of a genome-wide association study. Journal of the National Cancer Institute 103:425-435.

Forssen UM, Rutqvist LE, Ahlbom A, Feychting M (2005) Occupational magnetic fields and female breast cancer: a case-control study using Swedish population registers and new exposure data. American journal of epidemiology 161:250-259.

Fowke JH, Shu XO, Dai Q, Jin F, Cai Q, Gao YT, Zheng W (2004) Oral contraceptive use and breast cancer risk: modification by NAD(P)H:quinone oxoreductase (NQO1) genetic polymorphisms. Cancer epidemiology, biomarkers & prevention : a publication of the American Association for Cancer Research, cosponsored by the American Society of Preventive Oncology 13:1308-1315.

Gapska P, Scott RJ, Serrano-Fernandez P, Huzarski T, Byrski T, Kladny J, Gronwald J, Gorski B, Cybulski C, Lubinski J, Debniak T (2009) Vitamin D receptor variants and breast cancer risk in the Polish population. Breast cancer research and treatment 115:629-633.

Garcia-Closas M, Hall P, Nevanlinna H, Pooley K, Morrison J, Richesson DA, Bojesen SE, Nordestgaard BG, Axelsson CK, Arias JI, Milne RL, Ribas G, Gonzalez-Neira A, Benitez J, Zamora P, Brauch H, Justenhoven C, Hamann U, Ko YD, Bruening T, Haas S, Dork T, Schurmann P, Hillemanns P, Bogdanova N, Bremer M, Karstens JH, Fagerholm R, Aaltonen K, Aittomaki K, von Smitten K, Blomqvist C, Mannermaa A, Uusitupa M, Eskelinen M, Tengstrom M, Kosma VM, Kataja V, Chenevix-Trench G, Spurdle AB, Beesley J, Chen X, Devilee P, van Asperen CJ, Jacobi CE, Tollenaar RA, Huijts PE, Klijn JG, Chang-Claude J, Kropp S, Slanger T, Flesch-Janys D, Mutschelknauss E, Salazar R, Wang-Gohrke S, Couch F, Goode EL, Olson JE, Vachon C, Fredericksen ZS, Giles GG, Baglietto L, Severi G, Hopper JL, English DR, Southey MC, Haiman CA, Henderson BE, Kolonel LN, Le Marchand L, Stram DO, Hunter DJ, Hankinson SE, Cox DG, Tamimi R, Kraft P, Sherman ME, Chanock SJ, Lissowska J, Brinton LA, Peplonska B, Klijn JG, Hooning MJ, Meijers-Heijboer H, Collee JM, van den Ouweland A, Uitterlinden AG, Liu J, Lin LY, Yuqing L, Humphreys K, Czene K, Cox A, Balasubramanian SP, Cross SS, Reed MW, Blows F, Driver K, Dunning A, Tyrer J, Ponder BA, Sangrajrang S, Brennan P, McKay J, Odefrey F, Gabrieau V, Sigurdson A, Doody M, Struewing JP, Alexander B, Easton DF, Pharoah PD (2008) Heterogeneity of breast cancer associations with five susceptibility loci by clinical and pathological characteristics. PLoS genetics 4:e1000054.

Garcia-Closas M, Kristensen V, Langerod A, Qi Y, Yeager M, Burdett L, Welch R, Lissowska J, Peplonska B, Brinton L, Gerhard DS, Gram IT, Perou CM, Borresen-Dale AL, Chanock S (2007) Common genetic variation in TP53 and its flanking genes, WDR79 and ATP1B2, and susceptibility to breast cancer. International journal of cancer Journal international du cancer 121:2532-2538.

Gaudet MM, Egan KM, Lissowska J, Newcomb PA, Brinton LA, Titus-Ernstoff L, Yeager M, Chanock S, Welch R, Peplonska B, Trentham-Dietz A, Garcia-Closas M (2007) Genetic variation in tumor necrosis factor and lymphotoxin-alpha (TNF-LTA) and breast cancer risk. Human genetics 121:483-490.

Gaudet MM, Gammon MD, Bensen JT, Sagiv SK, Shantakumar S, Teitelbaum SL, Eng SM, Neugut AI, Santella RM (2008) Genetic variation of TP53, polycyclic aromatic hydrocarbon-related exposures, and breast cancer risk among women on Long Island, New York. Breast cancer research and treatment 108:93-99.

George SM, Park Y, Leitzmann MF, Freedman ND, Dowling EC, Reedy J, Schatzkin A, Hollenbeck A, Subar AF (2009) Fruit and vegetable intake and risk of cancer: a prospective cohort study. The American journal of clinical nutrition 89:347-353.

Gorski B, Cybulski C, Huzarski T, Byrski T, Gronwald J, Jakubowska A, Stawicka M, Gozdecka-Grodecka S, Szwiec M, Urbanski K, Mitus J, Marczyk E, Dziuba J, Wandzel P, Surdyka D, Haus O, Janiszewska H, Debniak T, Toloczko-Grabarek A, Medrek K, Masojc B, Mierzejewski M, Kowalska E, Narod SA, Lubinski J (2005) Breast cancer predisposing alleles in Poland. Breast cancer research and treatment 92:19-24.

Haiman CA, Henderson SO, Bretsky P, Kolonel LN, Henderson BE (2003) Genetic variation in angiotensin I-converting enzyme (ACE) and breast cancer risk: the multiethnic cohort. Cancer research 63:6984-6987.

Hankinson SE, Colditz GA, Manson JE, Willett WC, Hunter DJ, Stampfer MJ, Speizer FE (1997) A prospective study of oral contraceptive use and risk of breast cancer (Nurses' Health Study, United States). Cancer causes & control : CCC 8:65-72.

Haukka J, Sankila R, Klaukka T, Lonnqvist J, Niskanen L, Tanskanen A, Wahlbeck K, Tiihonen J (2010) Incidence of cancer and antidepressant medication: record linkage study. International journal of cancer Journal international du cancer 126:285-296.

Hedelin M, Lof M, Olsson M, Adlercreutz H, Sandin S, Weiderpass E (2008) Dietary phytoestrogens are not associated with risk of overall breast cancer but diets rich in coumestrol are inversely associated with risk of estrogen receptor and progesterone receptor negative breast tumors in Swedish women. The Journal of nutrition 138:938-945.

Higginbotham KS, Breyer JP, Bradley KM, Schuyler PA, Plummer WD, Jr., Freudenthal ME, Trentham-Dietz A, Newcomb PA, Sanders ME, Page DL, Parl FF, Egan KM, Dupont WD, Smith JR (2011) A multistage association study identifies a breast cancer genetic locus at NCOA7. Cancer research 71:3881-3888.

Hippisley-Cox J, Coupland C (2010) Unintended effects of statins in men and women in England and Wales: population based cohort study using the QResearch database. BMJ (Clinical research ed) 340:c2197.

Jakubowska A, Rozkrut D, Antoniou A, Hamann U, Scott RJ, McGuffog L, Healy S, Sinilnikova OM, Rennert G, Lejbkowicz F, Flugelman A, Andrulis IL, Glendon G, Ozcelik H, Thomassen M, Paligo M, Aretini P, Kantala J, Aroer B, von Wachenfeldt A, Liljegren A, Loman N, Herbst K, Kristoffersson U, Rosenquist R, Karlsson P, Stenmark-Askmalm M, Melin B, Nathanson KL, Domchek SM, Byrski T, Huzarski T, Gronwald J, Menkiszak J, Cybulski C, Serrano P, Osorio A, Cajal TR, Tsitlaidou M, Benitez J, Gilbert M, Rookus M, Aalfs CM, Kluijt I, Boessenkool-Pape JL, Meijers-Heijboer HE, Oosterwijk JC, van Asperen CJ, Blok MJ, Nelen MR, van den Ouweland AM, Seynaeve C, van der Luijt RB, Devilee P, Easton DF, Peock S, Frost D, Platte R, Ellis SD, Fineberg E, Evans DG, Lalloo F, Eeles R, Jacobs C, Adlard J, Davidson R, Eccles D, Cole T, Cook J, Godwin A, Bove B, Stoppa-Lyonnet D, Caux-Moncoutier V, Belotti M, Tirapo C, Mazoyer S, Barjhoux L, Boutry-Kryza N, Pujol P, Coupier I, Peyrat JP, Vennin P, Muller D, Fricker JP, Venat-Bouvet L, Johannsson OT, Isaacs C, Schmutzler R, Wappenschmidt B, Meindl A, Arnold N, Varon-Mateeva R, Niederacher D, Sutter C, Deissler H, Preisler-Adams S, Simard J, Soucy P, Durocher F, Chenevix-Trench G, Beesley J, Chen X, Rebbeck T, Couch F, Wang X, Lindor N, Fredericksen Z, Pankratz VS, Peterlongo P, Bonanni B, Fortuzzi S, Peissel B, Szabo C, Mai PL, Loud JT, Lubinski J (2012) Association of PHB 1630 C>T and MTHFR 677 C>T polymorphisms with breast and ovarian cancer risk in BRCA1/2 mutation carriers: results from a multicenter study. British journal of cancer 106:2016-2024.

Kerlikowske K, Miglioretti DL, Ballard-Barbash R, Weaver DL, Buist DS, Barlow WE, Cutter G, Geller BM, Yankaskas B, Taplin SH, Carney PA (2003) Prognostic characteristics of breast cancer among postmenopausal hormone users in a screened population. Journal of clinical oncology : official journal of the American Society of Clinical Oncology 21:4314-4321.

Khan NA, Castillo A, Koriyama C, Kijima Y, Umekita Y, Ohi Y, Higashi M, Sagara Y, Yoshinaka H, Tsuji T, Natsugoe S, Douchi T, Eizuru Y, Akiba S (2008) Human papillomavirus detected in female breast carcinomas in Japan. British journal of cancer 99:408-414.

Kuschel B, Chenevix-Trench G, Spurdle AB, Chen X, Hopper JL, Giles GG, McCredie M, Chang-Claude J, Gregory CS, Day NE, Easton DF, Ponder BA, Dunning AM, Pharoah PD (2005) Common polymorphisms in ERCC2 (Xeroderma pigmentosum D) are not associated with breast cancer risk. Cancer epidemiology, biomarkers & prevention : a publication of the American Association for Cancer Research, cosponsored by the American Society of Preventive Oncology 14:1828-1831.

Lambe M, Wigertz A, Garmo H, Walldius G, Jungner I, Hammar N (2011) Impaired glucose metabolism and diabetes and the risk of breast, endometrial, and ovarian cancer. Cancer causes & control : CCC 22:1163-1171.

Lee KM, Choi JY, Lee JE, Noh DY, Ahn SH, Han W, Yoo KY, Hayes RB, Kang D (2007) Genetic polymorphisms of NOS3 are associated with the risk of invasive breast cancer with lymph node involvement. Breast cancer research and treatment 106:433-438.

Lee SA, Fowke JH, Lu W, Ye C, Zheng Y, Cai Q, Gu K, Gao YT, Shu XO, Zheng W (2008) Cruciferous vegetables, the GSTP1 Ile105Val genetic polymorphism, and breast cancer risk. The American journal of clinical nutrition 87:753-760.

Lissowska J, Brinton LA, Zatonski W, Blair A, Bardin-Mikolajczak A, Peplonska B, Sherman ME, Szeszenia-Dabrowska N, Chanock S, Garcia-Closas M (2006) Tobacco smoking, NAT2 acetylation genotype and breast cancer risk. International journal of cancer Journal international du cancer 119:1961-1969.

Lissowska J, Gaudet MM, Brinton LA, Chanock SJ, Peplonska B, Welch R, Zatonski W, Szeszenia-Dabrowska N, Park S, Sherman M, Garcia-Closas M (2007) Genetic polymorphisms in the one-carbon metabolism pathway and breast cancer risk: a population-based case-control study and meta-analyses. International journal of cancer Journal international du cancer 120:2696-2703.

MARIE-GENICA (2010) Polymorphisms in the BRCA1 and ABCB1 genes modulate menopausal hormone therapy associated breast cancer risk in postmenopausal women. Breast cancer research and treatment 120:727-736.

MARIE-GENICA b (2010) Genetic polymorphisms in phase I and phase II enzymes and breast cancer risk associated with menopausal hormone therapy in postmenopausal women. Breast cancer research and treatment 119:463-474.

McDonald JA, Mandel MG, Marchbanks PA, Folger SG, Daling JR, Ursin G, Simon MS, Bernstein L, Strom BL, Norman SA, Malone KE, Weiss LK, Burkman RT, Weber AL, Spirtas R (2004) Alcohol exposure and breast cancer: results of the women's contraceptive and reproductive experiences study. Cancer epidemiology, biomarkers & prevention : a publication of the American Association for Cancer Research, cosponsored by the American Society of Preventive Oncology 13:2106-2116.

Mechanic LE, Millikan RC, Player J, de Cotret AR, Winkel S, Worley K, Heard K, Heard K, Tse CK, Keku T (2006) Polymorphisms in nucleotide excision repair genes, smoking and breast cancer in African Americans and whites: a population-based case-control study. Carcinogenesis 27:1377-1385.

Michels KB, Terry KL, Willett WC (2006) Longitudinal study on the role of body size in premenopausal breast cancer. Archives of internal medicine 166:2395-2402.

Mignone LI, Giovannucci E, Newcomb PA, Titus-Ernstoff L, Trentham-Dietz A, Hampton JM, Willett WC, Egan KM (2009) Dietary carotenoids and the risk of invasive breast cancer. International journal of cancer Journal international du cancer 124:2929-2937.

Millikan RC, Player J, de Cotret AR, Moorman P, Pittman G, Vannappagari V, Tse CK, Keku T (2004) Manganese superoxide dismutase Ala-9Val polymorphism and risk of breast cancer in a population-based case-control study of African Americans and whites. Breast cancer research : BCR 6:R264-274.

Millikan RC, Player JS, Decotret AR, Tse CK, Keku T (2005) Polymorphisms in DNA repair genes, medical exposure to ionizing radiation, and breast cancer risk. Cancer epidemiology, biomarkers & prevention : a publication of the American Association for Cancer Research, cosponsored by the American Society of Preventive Oncology 14:2326-2334.

Milne RL, Gaudet MM, Spurdle AB, Fasching PA, Couch FJ, Benitez J, Arias Perez JI, Zamora MP, Malats N, Dos Santos Silva I, Gibson LJ, Fletcher O, Johnson N, Anton-Culver H, Ziogas A, Figueroa J, Brinton L, Sherman ME, Lissowska J, Hopper JL, Dite GS, Apicella C, Southey MC, Sigurdson AJ, Linet MS, Schonfeld SJ, Freedman DM, Mannermaa A, Kosma VM, Kataja V, Auvinen P, Andrulis IL, Glendon G, Knight JA, Weerasooriya N, Cox A, Reed MW, Cross SS, Dunning AM, Ahmed S, Shah M, Brauch H, Ko YD, Bruning T, Lambrechts D, Reumers J, Smeets A, Wang-Gohrke S, Hall P, Czene K, Liu J, Irwanto AK, Chenevix-Trench G, Holland H, Giles GG, Baglietto L, Severi G, Bojensen SE, Nordestgaard BG, Flyger H, John EM, West DW, Whittemore AS, Vachon C, Olson JE, Fredericksen Z, Kosel M, Hein R, Vrieling A, Flesch-Janys D, Heinz J, Beckmann MW, Heusinger K, Ekici AB, Haeberle L, Humphreys MK, Morrison J, Easton DF, Pharoah PD, Garcia-Closas M, Goode EL, Chang-Claude J (2010) Assessing interactions between the associations of common genetic susceptibility variants, reproductive history and body mass index with breast cancer risk in the breast cancer association consortium: a combined case-control study. Breast cancer research : BCR 12:R110.

Montroni I, Santini D, Zucchini G, Fiacchi M, Zanotti S, Ugolini G, Manaresi A, Taffurelli M (2010) Nipple discharge: is its significance as a risk factor for breast cancer fully understood? Observational study including 915 consecutive patients who underwent selective duct excision. Breast cancer research and treatment 123:895-900.

Nagel G, Linseisen J, van Gils CH, Peeters PH, Boutron-Ruault MC, Clavel-Chapelon F, Romieu I, Tjonneland A, Olsen A, Roswall N, Witt PM, Overvad K, Rohrmann S, Kaaks R, Drogan D, Boeing H, Trichopoulou A, Stratigakou V, Zylis D, Engeset D, Lund E, Skeie G, Berrino F, Grioni S, Mattiello A, Masala G, Tumino R, Zanetti R, Ros MM, Bueno-de-Mesquita HB, Ardanaz E, Sanchez MJ, Huerta JM, Amiano P, Rodriguez L, Manjer J, Wirfalt E, Lenner P, Hallmans G, Spencer EA, Key TJ, Bingham S, Khaw KT, Rinaldi S, Slimani N, Boffetta P, Gallo V, Norat T, Riboli E (2010) Dietary beta-carotene, vitamin C and E intake and breast cancer risk in the European Prospective Investigation into Cancer and Nutrition (EPIC). Breast cancer research and treatment 119:753-765.

Nyante SJ, Gammon MD, Kaufman JS, Bensen JT, Lin DY, Barnholtz-Sloan JS, Hu Y, He Q, Luo J, Millikan RC (2011) Common genetic variation in adiponectin, leptin, and leptin receptor and association with breast cancer subtypes. Breast cancer research and treatment 129:593-606.

Onay UV, Aaltonen K, Briollais L, Knight JA, Pabalan N, Kilpivaara O, Andrulis IL, Blomqvist C, Nevanlinna H, Ozcelik H (2008) Combined effect of CCND1 and COMT polymorphisms and increased breast cancer risk. BMC cancer 8:6.

Pala V, Krogh V, Berrino F, Sieri S, Grioni S, Tjonneland A, Olsen A, Jakobsen MU, Overvad K, Clavel-Chapelon F, Boutron-Ruault MC, Romieu I, Linseisen J, Rohrmann S, Boeing H, Steffen A, Trichopoulou A, Benetou V, Naska A, Vineis P, Tumino R, Panico S, Masala G, Agnoli C, Engeset D, Skeie G, Lund E, Ardanaz E, Navarro C, Sanchez MJ, Amiano P, Svatetz CA, Rodriguez L, Wirfalt E, Manjer J, Lenner P, Hallmans G, Peeters PH, van Gils CH, Bueno-de-Mesquita HB, van Duijnhoven FJ, Key TJ, Spencer E, Bingham S, Khaw KT, Ferrari P, Byrnes G, Rinaldi S, Norat T, Michaud DS, Riboli E (2009) Meat, eggs, dairy products, and risk of breast cancer in the European Prospective Investigation into Cancer and Nutrition (EPIC) cohort. The American journal of clinical nutrition 90:602-612.

Park Y, Leitzmann MF, Subar AF, Hollenbeck A, Schatzkin A (2009) Dairy food, calcium, and risk of cancer in the NIH-AARP Diet and Health Study. Archives of internal medicine 169:391-401.

Pharoah PD, Tyrer J, Dunning AM, Easton DF, Ponder BA (2007) Association between common variation in 120 candidate genes and breast cancer risk. PLoS genetics 3:e42.

Pooley KA, Healey CS, Smith PL, Pharoah PD, Thompson D, Tee L, West J, Jordan C, Easton DF, Ponder BA, Dunning AM (2006) Association of the progesterone receptor gene with breast cancer risk: a single-nucleotide polymorphism tagging approach. Cancer epidemiology, biomarkers & prevention : a publication of the American Association for Cancer Research, cosponsored by the American Society of Preventive Oncology 15:675-682.

Raish M, Dhillon VS, Ahmad A, Ansari MA, Mudassar S, Shahid M, Batra V, Gupta P, Das BC, Shukla N, Husain SA (2009) Promoter Hypermethylation in Tumor Suppressing Genes p16 and FHIT and Their Relationship with Estrogen Receptor and Progesterone Receptor Status in Breast Cancer Patients from Northern India. Translational oncology 2:264-270.

Ralph DA, Zhao LP, Aston CE, Manjeshwar S, Pugh TW, DeFreese DC, Gramling BA, Shimasaki CD, Jupe ER (2007) Age-specific association of steroid hormone pathway gene polymorphisms with breast cancer risk. Cancer 109:1940-1948.

Reeves GK, Pirie K, Beral V, Green J, Spencer E, Bull D (2007) Cancer incidence and mortality in relation to body mass index in the Million Women Study: cohort study. BMJ (Clinical research ed) 335:1134.

Rintala P, Pukkala E, Paakkulainen HT, Vihko VJ (2002) Self-experienced physical workload and risk of breast cancer. Scandinavian journal of work, environment & health 28:158-162.

Rosato V, Bosetti C, Talamini R, Levi F, Montella M, Giacosa A, Negri E, La Vecchia C (2011) Metabolic syndrome and the risk of breast cancer in postmenopausal women. Annals of oncology : official journal of the European Society for Medical Oncology / ESMO 22:2687-2692.

Schmidt MK, Reincke S, Broeks A, Braaf LM, Hogervorst FB, Tollenaar RA, Johnson N, Fletcher O, Peto J, Tommiska J, Blomqvist C, Nevanlinna HA, Healey CS, Dunning AM, Pharoah PD, Easton DF, Dork T, Van't Veer LJ (2007) Do MDM2 SNP309 and TP53 R72P interact in breast cancer susceptibility? A large pooled series from the breast cancer association consortium. Cancer research 67:9584-9590.

Shikany JM, Redden DT, Neuhouser ML, Chlebowski RT, Rohan TE, Simon MS, Liu S, Lane DS, Tinker L (2011) Dietary glycemic load, glycemic index, and carbohydrate and risk of breast cancer in the Women's Health Initiative. Nutrition and cancer 63:899-907.

Shin MH, Holmes MD, Hankinson SE, Wu K, Colditz GA, Willett WC (2002) Intake of dairy products, calcium, and vitamin d and risk of breast cancer. Journal of the National Cancer Institute 94:1301-1311.

Shrubsole MJ, Gao YT, Cai Q, Shu XO, Dai Q, Jin F, Zheng W (2006) MTR and MTRR polymorphisms, dietary intake, and breast cancer risk. Cancer epidemiology, biomarkers & prevention : a publication of the American Association for Cancer Research, cosponsored by the American Society of Preventive Oncology 15:586-588.

Simon MS, Tang MT, Bernstein L, Norman SA, Weiss L, Burkman RT, Daling JR, Deapen D, Folger SG, Malone K, Marchbanks PA, McDonald JA, Strom BL, Wilson HG, Spirtas R (2002) Do thyroid disorders increase the risk of breast cancer? Cancer epidemiology, biomarkers & prevention : a publication of the American Association for Cancer Research, cosponsored by the American Society of Preventive Oncology 11:1574-1578.

Slattery ML, Sweeney C, Herrick J, Wolff R, Baumgartner K, Giuliano A, Byers T (2007) ESR1, AR, body size, and breast cancer risk in Hispanic and non-Hispanic white women living in the Southwestern United States. Breast cancer research and treatment 105:327-335.

Smyth PP, Shering SG, Kilbane MT, Murray MJ, McDermott EW, Smith DF, O'Higgins NJ (1998) Serum thyroid peroxidase autoantibodies, thyroid volume, and outcome in breast carcinoma. The Journal of clinical endocrinology and metabolism 83:2711-2716.

Spurdle AB, Chang JH, Byrnes GB, Chen X, Dite GS, McCredie MR, Giles GG, Southey MC, Chenevix-Trench G, Hopper JL (2007) A systematic approach to analysing gene-gene interactions: polymorphisms at the microsomal epoxide hydrolase EPHX and glutathione S-transferase GSTM1, GSTT1, and GSTP1 loci and breast cancer risk. Cancer epidemiology, biomarkers & prevention : a publication of the American Association for Cancer Research, cosponsored by the American Society of Preventive Oncology 16:769-774.

Tamimi RM, Hankinson SE, Campos H, Spiegelman D, Zhang S, Colditz GA, Willett WC, Hunter DJ (2005) Plasma carotenoids, retinol, and tocopherols and risk of breast cancer. American journal of epidemiology 161:153-160.

Tamimi RM, Hankinson SE, Ding S, Gagalang V, Larson GP, Spiegelman D, Colditz GA, Krontiris TG, Hunter DJ (2003) The HRAS1 variable number of tandem repeats and risk of breast cancer. Cancer epidemiology, biomarkers & prevention : a publication of the American Association for Cancer Research, cosponsored by the American Society of Preventive Oncology 12:1528-1530.

Tavani A, Pregnolato A, La Vecchia C, Favero A, Franceschi S (1998) Coffee consumption and the risk of breast cancer. European journal of cancer prevention : the official journal of the European Cancer Prevention Organisation (ECP) 7:77-82.

Thompson WD, Jacobson HI, Negrini B, Janerich DT (1989) Hypertension, pregnancy, and risk of breast cancer. Journal of the National Cancer Institute 81:1571-1574.

Tommiska J, Jansen L, Kilpivaara O, Edvardsen H, Kristensen V, Tamminen A, Aittomaki K, Blomqvist C, Borresen-Dale AL, Nevanlinna H (2006) ATM variants and cancer risk in breast cancer patients from Southern Finland. BMC cancer 6:209.

Vatten LJ, Forman MR, Nilsen TI, Barrett JC, Romundstad PR (2007) The negative association between pre-eclampsia and breast cancer risk may depend on the offspring's gender. British journal of cancer 96:1436-1438.

Wen W, Gao YT, Shu XO, Yu H, Cai Q, Smith JR, Zheng W (2005) Insulin-like growth factor-I gene polymorphism and breast cancer risk in Chinese women. International journal of cancer Journal international du cancer 113:307-311.

Zhang Y, Coogan PF, Palmer JR, Strom BL, Rosenberg L (2005) Use of nonsteroidal antiinflammatory drugs and risk of breast cancer: the Case-Control Surveillance Study revisited. American journal of epidemiology 162:165-170.

Zhang Y, Newcomb PA, Egan KM, Titus-Ernstoff L, Chanock S, Welch R, Brinton LA, Lissowska J, Bardin-Mikolajczak A, Peplonska B, Szeszenia-Dabrowska N, Zatonski W, Garcia-Closas M (2006) Genetic polymorphisms in base-excision repair pathway genes and risk of breast cancer. Cancer epidemiology, biomarkers & prevention : a publication of the American Association for Cancer Research, cosponsored by the American Society of Preventive Oncology 15:353-358.

Zheng W, Long J, Gao YT, Li C, Zheng Y, Xiang YB, Wen W, Levy S, Deming SL, Haines JL, Gu K, Fair AM, Cai Q, Lu W, Shu XO (2009) Genome-wide association study identifies a new breast cancer susceptibility locus at 6q25.1. Nature genetics 41:324-328.

**Glaucoma**

Alward WL, Kwon YH, Kawase K, Craig JE, Hayreh SS, Johnson AT, Khanna CL, Yamamoto T, Mackey DA, Roos BR, Affatigato LM, Sheffield VC, Stone EM (2003) Evaluation of optineurin sequence variations in 1,048 patients with open-angle glaucoma. American journal of ophthalmology 136:904-910.

Dimasi DP, Hewitt AW, Green CM, Mackey DA, Craig JE (2005) Lack of association of p53 polymorphisms and haplotypes in high and normal tension open angle glaucoma. Journal of medical genetics 42:e55.

Fan BJ, Pasquale L, Grosskreutz CL, Rhee D, Chen T, DeAngelis MM, Kim I, del Bono E, Miller JW, Li T, Haines JL, Wiggs JL (2008) DNA sequence variants in the LOXL1 gene are associated with pseudoexfoliation glaucoma in a U.S. clinic-based population with broad ethnic diversity. BMC medical genetics 9:5.

Fingert JH, Heon E, Liebmann JM, Yamamoto T, Craig JE, Rait J, Kawase K, Hoh ST, Buys YM, Dickinson J, Hockey RR, Williams-Lyn D, Trope G, Kitazawa Y, Ritch R, Mackey DA, Alward WL, Sheffield VC, Stone EM (1999) Analysis of myocilin mutations in 1703 glaucoma patients from five different populations. Human molecular genetics 8:899-905.

Funayama T, Ishikawa K, Ohtake Y, Tanino T, Kurosaka D, Kimura I, Suzuki K, Ideta H, Nakamoto K, Yasuda N, Fujimaki T, Murakami A, Asaoka R, Hotta Y, Tanihara H, Kanamoto T, Mishima H, Fukuchi T, Abe H, Iwata T, Shimada N, Kudoh J, Shimizu N, Mashima Y (2004) Variants in optineurin gene and their association with tumor necrosis factor-alpha polymorphisms in Japanese patients with glaucoma. Investigative ophthalmology & visual science 45:4359-4367.

Kuzin AA, Varma R, Reddy HS, Torres M, Azen SP (2010) Ocular biometry and open-angle glaucoma: the Los Angeles Latino Eye Study. Ophthalmology 117:1713-1719.

Liu Y, Schmidt S, Qin X, Gibson J, Munro D, Wiggs JL, Hauser MA, Allingham RR (2007) No association between OPA1 polymorphisms and primary open-angle glaucoma in three different populations. Molecular vision 13:2137-2141.

Mabuchi F, Tang S, Kashiwagi K, Yamagata Z, Iijima H, Tsukahara S (2007) The OPA1 gene polymorphism is associated with normal tension and high tension glaucoma. American journal of ophthalmology 143:125-130.

Micheal S, Qamar R, Akhtar F, Khan MI, Khan WA, Ahmed A (2009) MTHFR gene C677T and A1298C polymorphisms and homocysteine levels in primary open angle and primary closed angle glaucoma. Molecular vision 15:2268-2278.

Pasutto F, Krumbiegel M, Mardin CY, Paoli D, Lammer R, Weber BH, Kruse FE, Schlotzer-Schrehardt U, Reis A (2008) Association of LOXL1 common sequence variants in German and Italian patients with pseudoexfoliation syndrome and pseudoexfoliation glaucoma. Investigative ophthalmology & visual science 49:1459-1463.

Wu SY, Nemesure B, Leske MC (1999) Refractive errors in a black adult population: the Barbados Eye Study. Investigative ophthalmology & visual science 40:2179-2184.

Zetterberg M, Tasa G, Palmer MS, Juronen E, Toover E, Blennow K, Zetterberg H (2007) Methylenetetrahydrofolate reductase genetic polymorphisms in patients with primary open-angle glaucoma. Ophthalmic genetics 28:47-50.

**Psoriasis**

Butt C, Lim S, Greenwood C, Rahman P (2007) VEGF, FGF1, FGF2 and EGF gene polymorphisms and psoriatic arthritis. BMC musculoskeletal disorders 8:1.

Cohen AD, Weitzman D, Dreiher J (2010) Psoriasis and hypertension: a case-control study. Acta dermato-venereologica 90:23-26.

Coto-Segura P, Coto E, Alvarez V, Morales B, Soto-Sanchez J, Corao AI, Santos-Juanes J (2010) Apolipoprotein epsilon4 allele is associated with psoriasis severity. Archives of dermatological research 302:145-149.

Gudjonsson JE, Karason A, Antonsdottir A, Runarsdottir EH, Hauksson VB, Upmanyu R, Gulcher J, Stefansson K, Valdimarsson H (2003) Psoriasis patients who are homozygous for the HLA-Cw*0602 allele have a 2.5-fold increased risk of developing psoriasis compared with Cw6 heterozygotes. The British journal of dermatology 148:233-235.

Halsall JA, Osborne JE, Pringle JH, Hutchinson PE (2005) Vitamin D receptor gene polymorphisms, particularly the novel A-1012G promoter polymorphism, are associated with vitamin D3 responsiveness and non-familial susceptibility in psoriasis. Pharmacogenetics and genomics 15:349-355.

Huerta C, Rivero E, Rodriguez LA (2007) Incidence and risk factors for psoriasis in the general population. Archives of dermatology 143:1559-1565.

Nair RP, Ruether A, Stuart PE, Jenisch S, Tejasvi T, Hiremagalore R, Schreiber S, Kabelitz D, Lim HW, Voorhees JJ, Christophers E, Elder JT, Weichenthal M (2008) Polymorphisms of the IL12B and IL23R genes are associated with psoriasis. The Journal of investigative dermatology 128:1653-1661.

Prodanovich S, Kirsner RS, Kravetz JD, Ma F, Martinez L, Federman DG (2009) Association of psoriasis with coronary artery, cerebrovascular, and peripheral vascular diseases and mortality. Archives of dermatology 145:700-703.

Rucevic I, Stefanic M, Tokic S, Vuksic M, Glavas-Obrovac L, Barisic-Drusko V (2012) Lack of association of vitamin D receptor gene 3'-haplotypes with psoriasis in Croatian patients. The Journal of dermatology 39:58-62.

Smith RL, Warren RB, Eyre S, Ke X, Young HS, Allen M, Strachan D, McArdle W, Gittins MP, Barker JN, Griffiths CE, Worthington J (2008) Polymorphisms in the PTPN22 region are associated with psoriasis of early onset. The British journal of dermatology 158:962-968.

Sun LD, Cheng H, Wang ZX, Zhang AP, Wang PG, Xu JH, Zhu QX, Zhou HS, Ellinghaus E, Zhang FR, Pu XM, Yang XQ, Zhang JZ, Xu AE, Wu RN, Xu LM, Peng L, Helms CA, Ren YQ, Zhang C, Zhang SM, Nair RP, Wang HY, Lin GS, Stuart PE, Fan X, Chen G, Tejasvi T, Li P, Zhu J, Li ZM, Ge HM, Weichenthal M, Ye WZ, Zhang C, Shen SK, Yang BQ, Sun YY, Li SS, Lin Y, Jiang JH, Li CT, Chen RX, Cheng J, Jiang X, Zhang P, Song WM, Tang J, Zhang HQ, Sun L, Cui J, Zhang LJ, Tang B, Huang F, Qin Q, Pei XP, Zhou AM, Shao LM, Liu JL, Zhang FY, Du WD, Franke A, Bowcock AM, Elder JT, Liu JJ, Yang S, Zhang XJ (2010) Association analyses identify six new psoriasis susceptibility loci in the Chinese population. Nature genetics 42:1005-1009.

**Rheumatoid Arthritis**

Barton A, Thomson W, Ke X, Eyre S, Hinks A, Bowes J, Gibbons L, Plant D, Wilson AG, Marinou I, Morgan A, Emery P, Steer S, Hocking L, Reid DM, Wordsworth P, Harrison P, Worthington J (2008) Re-evaluation of putative rheumatoid arthritis susceptibility genes in the post-genome wide association study era and hypothesis of a key pathway underlying susceptibility. Human molecular genetics 17:2274-2279.

Barton b A, Bowes J, Eyre S, Spreckley K, Hinks A, John S, Worthington J (2004) A functional haplotype of the PADI4 gene associated with rheumatoid arthritis in a Japanese population is not associated in a United Kingdom population. Arthritis and rheumatism 50:1117-1121.

Burr ML, Naseem H, Hinks A, Eyre S, Gibbons LJ, Bowes J, Wilson AG, Maxwell J, Morgan AW, Emery P, Steer S, Hocking L, Reid DM, Wordsworth P, Harrison P, Thomson W, Worthington J, Barton A (2010) PADI4 genotype is not associated with rheumatoid arthritis in a large UK Caucasian population. Annals of the rheumatic diseases 69:666-670.

Carreira PE, Gonzalez-Crespo MR, Ciruelo E, Pablos JL, Santiago B, Gomez-Camara A, Gomez-Reino JJ (2005) Polymorphism of the interleukin-1 receptor antagonist gene: a factor in susceptibility to rheumatoid arthritis in a Spanish population. Arthritis and rheumatism 52:3015-3019.

Eyre S, Bowes J, Spreckley K, Potter C, Ring S, Strachan D, Worthington J, Barton A (2006) Investigation of the MHC2TA gene, associated with rheumatoid arthritis in a Swedish population, in a UK rheumatoid arthritis cohort. Arthritis and rheumatism 54:3417-3422.

Ikari K, Kuwahara M, Nakamura T, Momohara S, Hara M, Yamanaka H, Tomatsu T, Kamatani N (2005) Association between PADI4 and rheumatoid arthritis: a replication study. Arthritis and rheumatism 52:3054-3057.

Johnsen AK, Plenge RM, Butty V, Campbell C, Dieguez-Gonzalez R, Gomez-Reino JJ, Shadick N, Weinblatt M, Gonzalez A, Gregersen PK, Benoist C, Mathis D (2008) A broad analysis of IL1 polymorphism and rheumatoid arthritis. Arthritis and rheumatism 58:1947-1957.

Karlson EW, Lee IM, Cook NR, Manson JE, Buring JE, Hennekens CH (1999) A retrospective cohort study of cigarette smoking and risk of rheumatoid arthritis in female health professionals. Arthritis and rheumatism 42:910-917.

Kim YJ, Park JH, Kim I, Kim JO, Bae JS, Shin HD, Bae SC (2008) Putative role of functional interferon regulatory factor 5 (IRF5) polymorphism in rheumatoid arthritis in a Korean population. The Journal of rheumatology 35:2106-2118.

Kochi Y, Thabet MM, Suzuki A, Okada Y, Daha NA, Toes RE, Huizinga TW, Myouzen K, Kubo M, Yamada R, Nakamura Y, Yamamoto K (2011) PADI4 polymorphism predisposes male smokers to rheumatoid arthritis. Annals of the rheumatic diseases 70:512-515.

Lindner E, Nordang GB, Melum E, Flato B, Selvaag AM, Thorsby E, Kvien TK, Forre OT, Lie BA (2007) Lack of association between the chemokine receptor 5 polymorphism CCR5delta32 in rheumatoid arthritis and juvenile idiopathic arthritis. BMC medical genetics 8:33.

McKinney C, Fanciulli M, Merriman ME, Phipps-Green A, Alizadeh BZ, Koeleman BP, Dalbeth N, Gow PJ, Harrison AA, Highton J, Jones PB, Stamp LK, Steer S, Barrera P, Coenen MJ, Franke B, van Riel PL, Vyse TJ, Aitman TJ, Radstake TR, Merriman TR (2010) Association of variation in Fcgamma receptor 3B gene copy number with rheumatoid arthritis in Caucasian samples. Annals of the rheumatic diseases 69:1711-1716.

Mikuls TR, Levan T, Gould KA, Yu F, Thiele GM, Bynote KK, Conn D, Jonas BL, Callahan LF, Smith E, Brasington R, Moreland LW, Reynolds R, Gaffo A, Bridges SL, Jr. (2012) Impact of interactions of cigarette smoking with NAT2 polymorphisms on rheumatoid arthritis risk in African Americans. Arthritis and rheumatism 64:655-664.

Newman WG, Zhang Q, Liu X, Walker E, Ternan H, Owen J, Johnson B, Greer W, Mosher DP, Maksymowych WP, Bykerk VP, Keystone EC, Amos CI, Siminovitch KA (2006) Rheumatoid arthritis association with the FCRL3 -169C polymorphism is restricted to PTPN22 1858T-homozygous individuals in a Canadian population. Arthritis and rheumatism 54:3820-3827.

Padyukov b L, Hytonen AM, Smolnikova M, Hahn-Zoric M, Nilsson N, Hanson LA, Tarkowski A, Klareskog L (2004) Polymorphism in promoter region of IL10 gene is associated with rheumatoid arthritis in women. The Journal of rheumatology 31:422-425.

Padyukov L, Silva C, Stolt P, Alfredsson L, Klareskog L (2004) A gene-environment interaction between smoking and shared epitope genes in HLA-DR provides a high risk of seropositive rheumatoid arthritis. Arthritis and rheumatism 50:3085-3092.

Panoulas VF, Douglas KM, Smith JP, Stavropoulos-Kalinoglou A, Metsios GS, Nightingale P, Kitas GD (2009) Transforming growth factor-beta1 869T/C, but not interleukin-6 -174G/C, polymorphism associates with hypertension in rheumatoid arthritis. Rheumatology (Oxford, England) 48:113-118.

Paradowska-Gorycka A, Trefler J, Maciejewska-Stelmach J, Lacki JK (2010) Interleukin-10 gene promoter polymorphism in Polish rheumatoid arthritis patients. International journal of immunogenetics 37:225-231.

Plenge RM, Padyukov L, Remmers EF, Purcell S, Lee AT, Karlson EW, Wolfe F, Kastner DL, Alfredsson L, Altshuler D, Gregersen PK, Klareskog L, Rioux JD (2005) Replication of putative candidate-gene associations with rheumatoid arthritis in >4,000 samples from North America and Sweden: association of susceptibility with PTPN22, CTLA4, and PADI4. American journal of human genetics 77:1044-1060.

Potter C, Eyre S, Cope A, Worthington J, Barton A (2007) Investigation of association between the TRAF family genes and RA susceptibility. Annals of the rheumatic diseases 66:1322-1326.

Robinson JI, Barrett JH, Taylor JC, Naven M, Corscadden D, Barton A, Wilson AG, Emery P, Isaacs JD, Morgan AW (2010) Dissection of the FCGR3A association with RA: increased association in men and with autoantibody positive disease. Annals of the rheumatic diseases 69:1054-1057.

Seldin MF, Shigeta R, Laiho K, Li H, Saila H, Savolainen A, Leirisalo-Repo M, Aho K, Tuomilehto-Wolf E, Kaarela K, Kauppi M, Alexander HC, Begovich AB, Tuomilehto J (2005) Finnish case-control and family studies support PTPN22 R620W polymorphism as a risk factor in rheumatoid arthritis, but suggest only minimal or no effect in juvenile idiopathic arthritis. Genes and immunity 6:720-722.

Shimane K, Kochi Y, Horita T, Ikari K, Amano H, Hirakata M, Okamoto A, Yamada R, Myouzen K, Suzuki A, Kubo M, Atsumi T, Koike T, Takasaki Y, Momohara S, Yamanaka H, Nakamura Y, Yamamoto K (2010) The association of a nonsynonymous single-nucleotide polymorphism in TNFAIP3 with systemic lupus erythematosus and rheumatoid arthritis in the Japanese population. Arthritis and rheumatism 62:574-579.

Sigurdsson S, Padyukov L, Kurreeman FA, Liljedahl U, Wiman AC, Alfredsson L, Toes R, Ronnelid J, Klareskog L, Huizinga TW, Alm G, Syvanen AC, Ronnblom L (2007) Association of a haplotype in the promoter region of the interferon regulatory factor 5 gene with rheumatoid arthritis. Arthritis and rheumatism 56:2202-2210.

Sugiura T, Kawaguchi Y, Ikari K, Ichida H, Kawamoto M, Momohara S, Hara M, Yamanaka H (2011) Interleukin-18 promoter polymorphisms in Japanese patients with rheumatoid arthritis: protective effect of the T allele and T/T genotype at rs360722. Modern rheumatology / the Japan Rheumatism Association 21:359-364.

The WTCCC-Burton PR (2007) Genome-wide association study of 14,000 cases of seven common diseases and 3,000 shared controls. Nature 447:661-678.

Thomson W, Barton A, Ke X, Eyre S, Hinks A, Bowes J, Donn R, Symmons D, Hider S, Bruce IN, Wilson AG, Marinou I, Morgan A, Emery P, Carter A, Steer S, Hocking L, Reid DM, Wordsworth P, Harrison P, Strachan D, Worthington J (2007) Rheumatoid arthritis association at 6q23. Nature genetics 39:1431-1433.

Tsukahara S, Iwamoto T, Ikari K, Inoue E, Tomatsu T, Hara M, Yamanaka H, Kamatani N, Momohara S (2008) CTLA-4 CT60 polymorphism is not an independent genetic risk marker of rheumatoid arthritis in a Japanese population. Annals of the rheumatic diseases 67:428-429.
